# Correlation-based and feature-driven mutation signature analyses to identify genetic features associated with DNA mutagenic processes in cancer genomes

**DOI:** 10.5808/gi.21047

**Published:** 2021-12-31

**Authors:** Hye Young Jeong, Jinseon Yoo, Hyunwoo Kim, Tae-Min Kim

**Affiliations:** 1 Department of Medical Informatics, College of Medicine, The Catholic University of Korea, Seoul 06591, Korea; 2 Cancer Research Institute, College of Medicine, The Catholic University of Korea, Seoul 06591, Korea; 3 Department of Biomedicine and Health Sciences, Graduate School, The Catholic University of Korea, Seoul 06591, Korea

**Keywords:** DNA damage repair, mutation signature, tumor hypoxia, tumor mutation burden

## Abstract

Mutation signatures represent unique sequence footprints of somatic mutations resulting from specific DNA mutagenic and repair processes. However, their causal associations and the potential utility for genome research remain largely unknown. In this study, we performed PanCancer-scale correlative analyses to identify the genomic features associated with tumor mutation burdens (TMB) and individual mutation signatures. We observed that TMB was correlated with tumor purity, ploidy, and the level of aneuploidy, as well as with the expression of cell proliferation-related genes representing genomic covariates in evaluating TMB. Correlative analyses of mutation signature levels with genes belonging to specific DNA damage-repair processes revealed that deficiencies of *NHEJ1* and *ALKBH3* may contribute to mutations in the settings of APOBEC cytidine deaminase activation and DNA mismatch repair deficiency, respectively. We further employed a strategy to identify feature-driven, *de novo* mutation signatures and demonstrated that mutation signatures can be reconstructed using known causal features. Using the strategy, we further identified tumor hypoxia-related mutation signatures similar to the APOBEC-related mutation signatures, suggesting that APOBEC activity mediates hypoxia-related mutational consequences in cancer genomes. Our study advances the mechanistic insights into the TMB and signature-based DNA mutagenic and repair processes in cancer genomes. We also propose that feature-driven mutation signature analysis can further extend the categories of cancer-relevant mutation signatures and their causal relationships.

## Introduction

Recent advances in genomic sequencing technologies have yielded a huge catalog of somatic mutations in cancer genomes across diverse tumor types [1]. In addition to identifying cancer-driver mutations, including druggable targets [2,3], clinical benefits associated with the quantitative nature of somatic mutations, such as the tumor mutation burden (TMB), have been demonstrated to be predictive markers for immune checkpoint inhibitors [4-6]. The TMB of cancer genomes is highly variable within and between tumor types [7], with varying causal mechanisms that lead to hypermutation and mutator phenotypes [8]. To advance our understanding on the heterogeneity of TMB and its clinical relevance, it is essential to assess the roles of various exogenous and endogenous mutagenic agents and DNA repair-replication processes [9], as well as their relationship with genomic features.

Mutational processes are known to leave characteristic sequence features in the genomes [10]. Well-recognized examples include C:G>A:T transversions and C:G>T:A transitions associated with the mutagens of tobacco smoking in lung cancers [11] and ultraviolet radiation in skin cancers [12], respectively. The distinct sequence features of individual mutagenic processes indicate that the mutational processes that have been operative in cancer genomes can be inferred by sequence-based analyses. Accordingly, recently proposed trinucleotide context-based analysis of cancer genomes based on deconvolution techniques, such as non-negative matrix factorization (NMF), has revealed more than 30 mutations signatures across 7,000 cancer genomes (hereafter, Sig.#1 to Sig.#30 as annotated in the Sanger mutation signature database; https://cancer.sanger.ac.uk/cosmic/signatures) [10,13]. Signature-level mutation analysis enables the molecular dissection of TMB according to the distinct origins of the mutations because mutation signatures are often associated with causal genetic mechanisms or genes corresponding to endogenous mutagens and DNA repair-replication processes. For example, overactivity of APOBEC cytidine deaminase leads to the accumulation of mutations consistent with Sig.#2 and Sig.#13, with sequence preferences of C>G and C>T within the TpCpN trinucleotide contexts [14]. Genetic events responsible for the deficiency of DNA repair enzymes have also been shown for some mutation signatures, e.g., somatic DNA mismatch repair deficiency (MMRd) in colorectal and endometrial cancers are frequently associated with promoter hypermethylation and transcriptional downregulation of *MLH1* [7]. In addition, deregulation of DNA repair or the proofreading polymerase genes of *BRCA* - [15] and *POLE* -deficient genomes [16] leads to mutations consistent with Sig.#3 and Sig.#10, respectively [10]. However, the genetic mechanisms and potential gene markers for the majority of mutation signatures are still largely unknown.

It is also possible that the current list of mutation signatures is not yet complete. The missing mutation signatures may be found in somatic mutations with a unique presentation, e.g., a recent mutation signature analysis focusing on clustered mutations revealed a novel signature associated with an activity of a translesional polymerase of *POLH* [17]. We also reported a cisplatin treatment-related mutation signature that was exclusively observed in head and neck cancers with a history of chemotherapy, not in treatment-naïve cancer genomes [18]. Given that the current list of mutation signatures has been largely obtained by deconvolution-based methods, such as NMF, methods using differential nucleotide frequencies may lead to the identification of novel mutation signatures representing specific tumor phenotypes or genotypes.

Large-scaled multi-omics cancer genome data may serve as valuable resources to identify underlying genetic mechanisms and key DNA repair genes that contribute to somatic mutations and the TMB of cancer genomes. In this study, we performed PanCancer-scaled correlative analyses that linked TMB and mutation signatures with multi-omics datasets available from the PanCancer-scale Cancer Genome Atlas (TCGA) consortium. We first evaluated the correlation of TMBs with systematic genomic features, such as tumor purity, ploidy, and aneuploidy. Then, TMB was deconvoluted into the level of known mutation signatures (i.e., the extent of contribution of 30 mutation signatures in given cancer genomes), which were subject to multi-omics correlative analyses to discover known and novel relationships between mutation signatures and their potential genetic mechanisms. We also proposed a feature-driven mutation signature discovery method to identify *de novo* mutation signatures as differential trinucleotide frequencies based on tumor features of interests, such as homologous recombination (HR) deficiency or tumor hypoxia.

## Methods

### Mutation information

The TCGA cancer mutation profiles as well as from other multi-omics dataset encompassing 9,587 tumor specimens and >30 tumor types were downloaded from TCGA PanCancer Atlas (https://gdc.cancer.gov/about-data/publications/pancanatlas). Duplicate cases were removed and only mutations from primary tumor genomes, ignoring those from recurrent or metastatic genomes, were considered. TMB was defined as the sum of all types of somatic calls for single nucleotide variations and short indels. We also obtained tumor purity and ploidy data, as well as other copy number-related variables from the literature [19].

### Gene set enrichment analysis

To identify molecular functions associated with TMB, we calculated Pearson’s correlation coefficients for individual gene expressions (log-scaled RSEM) with TMB (log-scaled). Gene-level correlations were subjected to the pre-ranked version of gene set enrichment analysis (GSEA) with Gene Ontology (GO) terms [20] available in MSigDB (http://software.broadinstitute.org/gsea/msigdb/index.jsp; c5 category).

### Signature analysis

For known mutation signatures, we download 30 signatures as Sanger ver.2 mutation signatures (Sig.#1–Sig.#30, https://cancer.sanger.ac.uk/cosmic/signatures). For signature deconvolution, we used deconstructSigs R packages version 1.8.0 in R version 3.6.1 [21] to derive the relative contribution of individual mutation signatures to given cancer genomes. The estimated contribution or the number of mutations belonging to individual signatures were used as signature levels for the subsequent correlative analyses. For mutation signature analysis, we used 6,040 cancer genomes harboring no less than 50 mutations. For correlation, we selected 254 genes that belong to DNA damage and repair (DDR) processes available in a previous study [22].

### Supervised identification of mutation signatures

A mutation signature representing APOBEC overactivity was derived as the differentials of trinucleotide frequencies between the tumors with high and low expression of APOBEC3A (95th and 5th percentiles, respectively). In the case of the mutation signature representing *MLH1* deficiency, the differentials were calculated between the low and high expression of MLH1. Only positive values of differential trinucleotide frequencies were considered for mutation signatures, given that negative values have no biological significance for signature-based analysis. For the POLE-signature, the differential trinucleotide frequencies were obtained by comparison of the *POLE*-mutated and wild-type genomes. Such identified feature-driven mutation signatures were compared with known mutation signatures using hierarchical clustering of similarities in the 96 trinucleotide frequencies.

### Mutation signatures representing HR deficiency and tumor hypoxia

Three HR deficiency-representing scores of the number telomeric allelic imbalance (NtAI), large scale transition (LST), and loss of heterozygosity (HRD-LOH) were obtained from a previous publication [23]. Eight scores representing tumor hypoxia estimated from mRNA signatures were also obtained from the literature [24]. Two types of mutation signatures were acquired per score. For an example of a hypoxia score, positive and negative differential values of the trinucleotide frequencies between genomes with high and low hypoxia scores were obtained as hypoxia- and normoxia-representing mutation signatures. To estimate the mutation signature levels using two signatures for each genome score, we employed metagene projection, where the positive linear combination of the two mutation signatures, i.e., hypoxia and normoxia from a single genome score, were projected onto the normalized mutation frequencies across the genomes to be examined [25]. For metagene projection, we used Moore-Penrose generalized pseudoinverse with the ginv function of the R MASS library (version 7.3-51.4) as previously described [26].

## Results

### Genomic features associated with TMB across cancer genomes

**Fig. 1.**
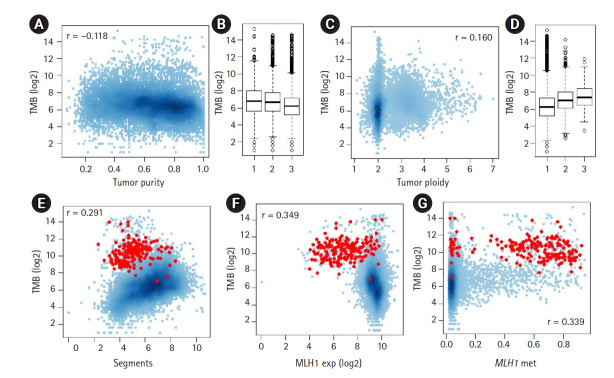
The relationship between tumor mutation burden (TMB) and other genomic features. (A) A scatter plot shows the inverse correlation between log2-transformed TMB and tumor purity. (B) TMB is shown for three equal-sized tumor bins (low-moderate-high tumor purity). (C, D) The positive correlation between TMB and tumor ploidy levels. (E) A scatter plot shows the positive correlation between TMB and the number of dosage-imbalance segments. Red dots indicate tumors with high microsatellite instability. (F, G) TMBs are shown against the expression level of MLH1 and the level of promoter methylation of *MLH1*.

To identify potential genomic covariates of TMB, we performed a PanCancer-scale correlative analysis with systematic genomic features, such as tumor purity and ploidy for 9,857 TCGA cases. First, we observed that the log-transformed TMB was inversely correlated with tumor purity (r = ‒0.118) (Fig. 1A and 1B). We previously showed that the tumor purity represents a surrogate marker for the level of tumor-infiltrating immune cells, including cytotoxic T cells [27], and the observed purity-TMB relationship may reflect the association between the level of tumor-infiltrating immune cells and TMB. We also observed that TMB was positively correlated with the level of aneuploidy, i.e., TMB was correlated with tumor ploidy (r = 0.161) (Fig. 1C and 1D) and also with the number of dosage-imbalanced copy number segments (r = 0.291) (Fig. 1E). These relationships have been previously reported [19,28], including the presence of a unique subset of tumors showing depletion of the copy number segments and an elevated mutation rate [7], a majority of which represented microsatellite instability-high or high microsatellite instability (MSI-H) genomes (red dots in Fig. 1E).

To assess the feasibility whereby the multi-omics dataset, including expression data, can be exploited to support the previously established relationship, we evaluated *MLH1*, whose promoter methylation, along with transcriptional downregulation are frequently observed in MSI-H genomes. Fig. 1F and 1G show the inverse and positive correlation of *MLH1* methylation and transcript levels with TMB (r = ‒0.349 and r = 0.339, respectively). This finding is consistent with a previous study that reported the DNA promoter methylation of *MLH1* was a major somatic mechanism and resulted in transcriptional downregulation leading to MMRd [29]. We also evaluated APOBEC3A, whose cytidine deaminase activity is associated with a substantial number of somatic mutations across multiple tumor types [14,30] (Supplementary Fig. 1). In contrast to *MLH1*, *APOBEC3A* showed a positive correlation with its transcript levels and TMB; however, the level of methylation of *APOBEC3A* was inversely correlated with TMB, suggesting that the transcript levels are controlled, at least to some extent, by promoter methylation levels as potential transcriptional regulators.

We also performed GSEA to identify molecular functions associated with the TMB (Supplementary Tables 1 and 2). The genes whose expression levels are positively or inversely correlated with TMB were enriched corresponded to cell-cycle and ion transport functions, respectively. Cell cycle-related replication stress is known to be a potential cause of mutations [31] and the transcriptional activation of cell-cycle-related genes may be related to the number of cell cycles of cancer stem cells. However, given that the TMB represents an admixture of mutations resulting from varying mutagenic or repair processes, the correlative analysis of TMB is limited in identifying the specific causal genes or mechanisms.

### Mutation signature correlative analyses for DNA DDR genes

**Fig. 2.**
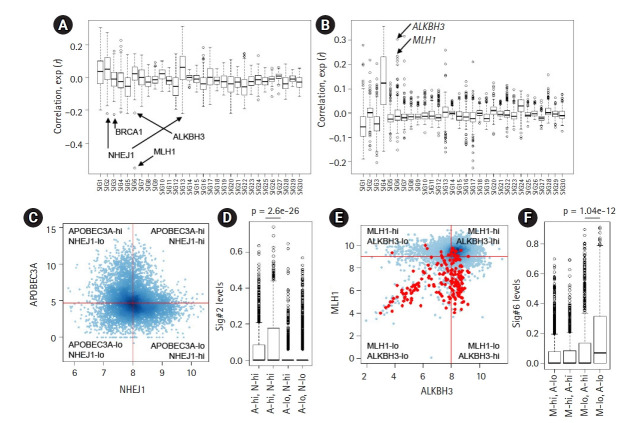
Correlative analysis of mutation levels with the damage and repair (DDR) gene. (A) The levels of 30 mutation signatures were correlated with the expression of 250 DDR genes. The *Y*-axiss shows the correlation, with arrows for selected genes (*ALKBH3*, *BRCA1*, *MLH1*, and *NHEJ1*). (B) The correlation between the 30 mutation signature levels and DDR gene promoter methylation levels. (C) A scatter plot shows the expression level of APOBEC3A and NHEJ1 (log-scaled). The cancer genomes were discriminated into four classes using the median APOBEC3A and NHEJ1 expression (shown by red lines). (D) A significant difference was observed in the Sig.#2 levels of those with or without *NHEJ1* deficiency only for APOBEC3A overexpression (p = 2.6e-26; t-test). (E) A scatter plot shows the distribution of ALKBH3 expression (*x*-axis; log2-scaled) and MLH1 expression (*y*-axis; log2-scaled). Red dots indicate the high microsatellite instability cases. (F) A significant difference was identified for the Sig.#6 levels between those with or without *ALKBH3* deficiency (p = 1.04e-12; t-test) only with *MLH1* deficiency.

The deconvolution-based, mutation signature level represents the relative contribution and activity levels of specific mutagenic or repair processes and may be a more appropriate resource for correlative analyses. For known mutation signatures, we obtained 30 mutation signatures (Sanger ver. 2 mutation signatures, annotated Sig.#1‒Sig.#30). For PanCancer tumor specimens, we estimated the relative contribution or signature levels of 30 known mutation signatures [21] and performed correlative analyses with the expression level of 254 genes belonging to the DDR pathway [22]. The distribution of PanCancer-scale correlation levels for 30 known mutation signatures are shown for gene expression and promoter methylation levels of the DDR genes, respectively (Fig. 2A and 2B). As expected, the lowest level of correlation with gene expression of MLH1 (r = ‒0.551) (arrow in Fig. 2A) and the second-highest level of correlation with promoter methylation of *MLH1* (r = 0.233) (arrow in Fig. 2B) were observed with Sig.#6 levels representing MMRd. The correlation levels for individual mutation signatures with DDR gene expression and promoter methylation are available in Supplementary Tables 3 and 4.

In addition to *MLH1*, a substantial level of inverse correlations were observed for certain DDR gene expression and mutation signature pairs, such as *NHEJ1*-Sig.#2 (r = ‒0.221, 1st ranked in Sig.#2) (Fig. 2A) and *BRCA1*-Sig.#3 (r = ‒0.224, 1st ranked in Sig.#3) (Fig. 2A), suggesting that their deficiency may give rise to mutations belonging to the corresponding mutation signatures. The association between *BRCA* loss and Sig.#3 representing HR deficiency has been well documented [15]; however, the association between *NHEJ1* that encodes essential DNA repair factors mediating non-homologous end-joining (NHEJ) with Sig.#2 is not well known. Furthermore, NHEJ1 expression was also negatively associated with the level of Sig.#13 (r = ‒0.212, 1st ranked in Sig.#13) (Fig. 2A), which is similar to Sig.#2 in potential causality and nucleotide composition [10]. Hierarchical clustering of joint profiles of mutation signature levels and DDR gene expression also highlights the association between *NHEJ1* and Sig.#2/Sig.#13 (Supplementary Fig. 2). Since no substantial level of correlation between NHEJ1 and APOBEC3A was noted, we classified the genomes into four classes according to the median expression of two genes (APOBEC3A-high/low and NHEJ1-high/low) and the Sig.#2 levels are shown against four classes (Fig. 2C). Genomes with low expression levels of APOBEC3A were almost devoid of Sig.#2 levels, regardless of NHEJ1 expression levels. However, the APOBEC3A-expressing genomes were further discriminated into two classes according to NHEJ1 expression levels with significant differences in the Sig.#2 levels (A-hi, N-hi vs. A-hi, N-lo, p = 2.6e-26; t-test) (Fig. 2D). These findings suggest that *NEHJ1* deficiency alone does not contribute to Sig.#2 mutations without APOBEC3A activity; however, an *NHEJ1* deficiency may potentiate the mutagenic activity of APOBEC cytidine deaminase. Given the roles of NHEJ1 in double-strand breakage (DSB) repair [32], it is assumed that an *NHEJ1* deficiency may pose DSB repair leaving the transient single strand terminus open as a substrate for APOBEC mutagenesis, but, this hypothesis requires further investigation.

For Sig.#6 associated with *MLH1* deficiency and MMRd, we also observed substantial correlation with ALKBH3 expression and *ALKHB3* promoter methylation (r = ‒0.215 and r = 0.304; 2nd and 1st ranked in Sig.#6, respectively) (Fig. 2A and 2B). The relationship between the methylation level of *MLH1* and *ALKBH3* and the level of Sig.#6 are illustrated in the hierarchical clustering of the joint methylation profiles and mutation signature levels (Supplementary Fig. 2), as well as those of the DDR gene expression profiles (Supplementary Fig. 3). The prevalent epigenetic modification of *ALKBH3* has been recently reported [22] but its functional significance is largely known. The similar regulatory relationship (i.e., an inverse correlation between the expression and methylation) of MLH1 and ALKBH3 is not simply explained by genomic adjacency (*ALKBH3* on 11p11.2 and *MLH1* on 3p22.2, respectively). We further classified the genomes into four classes according to the median expression of MLH1 and ALKBH3 (Fig. 2E; red dots for MSI-H cases) and the Sig.#6 levels are shown against four classes with significant difference (p = 1.04e-12, t-test) (Fig. 2F). This suggests that *ALKBH3* deficiency may accelerate the generation or accumulation of Sig.#6-consistent mutations in the context of MMRd induced by *MLH1* deficiency.

### Feature-driven discovery of mutation signatures

**Fig. 3.**
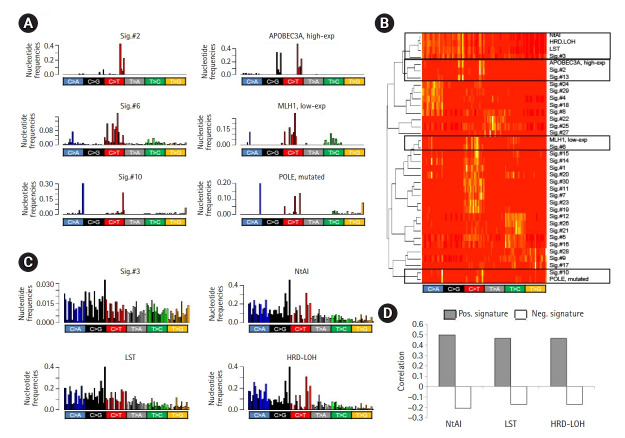
Feature-driven mutation signatures. (A) Bar plots show the trinucleotide frequencies of Sig.#2, Sig.#6, and Sig.#10 (left). The differentials of the trinucleotide frequencies are shown as potential mutation signatures. For example, the differential of trinucleotide frequencies between genomes with high and low expression of APOBEC3A is shown, along with those for MLH1 expression and *POLE* mutations with similar frequency distributions with their cognate mutation signatures (right). (B) A heatmap of trinucleotide frequency-based hierarchical clustering shows that Sig.#2, Sig.#6, and Sig.#10 are segregated with mutation signatures derived from APOBEC3A expression, MLH1 expression, and *POLE* mutations, respectively. Sig.#3 were also clustered with three mutation signatures derived from somatic copy number alterations (SCNA)‒based scores of HR deficiency. (C) Sig.#3 shows similar trinucleotide frequency distribution with differentials based on three homologous recombination (HR) deficiency scores of number of telomeric allelic imbalance (NtAI), large scale transition (LST), and homologous recombination deficiency–loss of heterozygosity (HRD-LOH). (D) The level of mutation signatures derived from HR deficiency scores shows a correlation with the HR deficiency scores. The signatures with positive and negative values were distinguished (Pos. and Neg. signatures, respectively) and separately analyzed for their correlation with HR deficiency scores (closed and open bars, respectively).

The correlation of gene expression or other genomic features, such as DNA promoter methylation levels with their attributed mutation signature levels, suggests that the corresponding mutation signature can be directly derived using gene-level features, such as mRNA expression. For example, *de novo* mutation signatures representing a deficiency in DDR genes can be derived as differential trinucleotide frequencies between the genomes with high and low expression of the gene. We tested the methods for three genes whose transcript levels or somatic mutations were associated with the cognate mutation signatures—Sig.#2/APOBEC3A (high-expression), Sig.#6/MLH1 (low-expression), and Sig.#10/*POLE* (somatic mutation) (Fig. 3). The mutation signature representing APOBEC overactivity was derived as differential trinucleotide frequencies between the genomes with high expression of APOBEC3A and those with low expression. The MMRd-representing signature was also inferred from the comparison of genomes with low MLH1 expression and those with high MLH1 expression. The genomes with *POLE* mutations were also compared to those without *POLE* mutations to derive *POLE* -related mutation signatures. Since the negative contribution of mutation profiles is not biologically meaningful in terms of mutation signatures, only positive differentials were taken into account. Fig. 3A shows three mutation signatures derived from 96 trinucleotide frequencies, along with their cognate mutation signatures (Sig.#2, Sig.#6, and Sig.#10, respectively). Fig. 3B also shows that feature-driven mutation signatures were segregated along with their cognate signatures in terms of trinucleotide frequencies.

In addition to single gene-based mutation signature discovery, we further tested whether the genomic features could be used for the discovery of *de novo* mutation signatures. We first obtained three somatic copy number alteration (SCNA)-based scores representing HR deficiency (NtAI, LST, and HRD-LOH) [23]. As expected, three HR deficiency score-driven mutation signatures were similar to Sig.#3 in terms of trinucleotide frequencies (Fig. 3C) and were also segregated along with Sig.#3 in hierarchical clustering (Fig. 3B). Next, we further explored whether the mutation signature levels estimated by metagene projection for each HR deficiency score were correlated with the original scores of HR deficiency. For each of HR deficiency scores, the positive and negative differential of the trinucleotide frequencies were collected as pairs of mutation signatures corresponding to the scores (Pos. and Neg. signatures, respectively). The mutation signature levels were estimated by metagene projection for each pair of signatures and then were correlated with the original scores. Fig. 3D shows the level of correlation. Positive (r = 0.465–0.499) and negative correlation (r = ‒0.171 to ‒0.208) were observed for the corresponding score pairs, suggesting that the SCNA-based HR deficiency scores could be reproduced to some extents, by the mutation signature levels (see Supplementary Fig. 4 for individual correlations).

### Mutation signatures representing tumor hypoxia

**Fig. 4.**
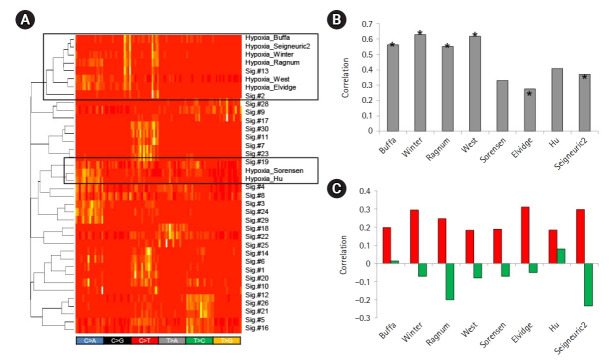
Mutation signatures of tumor hypoxia. (A) Eight mutation signatures were derived based on the mRNA-based tumor hypoxia scores with literature-based annotation as obtained. A heatmap is shown to demonstrate that six out of eight tumor hypoxia-representing mutation signatures are co-segregated with APOBEC-related Sig.#2 and Sig.#13 (open box; upper) for 96 trinucleotide contexts. Two hypoxia mutations were also co-segregated with Sig.#19. (B) A bar plot shows correlations between the level of APOBEC3A transcripts and tumor hypoxia scores. Asterisks identify six signatures co-segregated with Sig.#2 and Sig.#13 in (A). (C) Correlations are shown for the mutation signature levels derived from the tumor hypoxia scores and the hypoxia scores. Red and green represent the correlations for tumor hypoxia and normoxia scores, respectively.

To further test the feasibility of using feature-driven mutation signatures, we selected tumor hypoxia as one of the key tumor hallmarks associated with poor prognosis and treatment failure of various cancers [24]. We obtained eight mRNA signature-based tumor hypoxia scores from the literature and identified mutation signatures by the differential trinucleotide frequencies of mutations between hypoxic and normoxic tumors using the hypoxia scores. Of note, hierarchical clustering showed that the six of the eight hypoxia score-based mutation signatures showed similarities to those of APOBEC-related Sig.#2 and Sig.#13 (Fig. 4A). In addition, the hypoxia scores also showed a substantial correlation with the gene expression levels of APOBEC3A (Fig. 4B), suggesting that the genomic consequences of tumor hypoxia, at least for somatic mutations, were mainly attributed to APOBEC activity. For eight tumor hypoxia scores, the resulting mutation signatures and their levels measured by metagene projection were substantially correlated with the mRNA-based hypoxia scores (Fig. 4C). This suggests that the impact of tumor hypoxia in terms of somatic mutations may have specific nucleotide predisposition on the tumor genome and the level of impact may be predicted by the mutation profiles of cancer genomes.

## Discussion

In this study, we performed PanCancer-scaled correlative analyses for the TMB and their deconvoluted mutation signatures with various genomic features, including the expression of DDR genes. We further proposed an analytical framework to derive feature-driven mutation signatures representing the genotypic or phenotypic variables of interest.

TMB, a measure of the total number of mutations in a given cancer genome, has been recently highlighted as a biomarker for treatment with immune checkpoint inhibitors [5,33]. Thus, it is important to identify TMB-correlating genomic features. This study demonstrated that systematic genomic variables of cancer genomes, such as tumor purity, ploidy, and the level of aneuploidy, were correlated with TMB. But whether the observed correlation was due to a causal relationship or the identified genomic features represented confounding factors remains unknown. For the latter, the correlating features can be taken into account in TMB-centric correlative analysis, e.g., clinical benefits of high TMB for immunotherapy. We recently demonstrated that tumor purity was a confounding factor for cancer genome analyses, including the correlation between TMB and the abundance of tumor-infiltrating cells [27]. Given the overall positive correlation between TMB and the level of aneuploidy, it is still possible that an underlying mechanism in the cancer genome elevates both the genomic instability in terms of tumor ploidy and aneuploidy, along with the TMB. One main assumption is that the TMB of individual cancer genomes represents the aggregate of multiple mutagenic and DNA repair-replicative processes. GSEA only revealed the universal cellular functions of the cell cycles and chromosome-related genes were relatively overexpressed in high-TMB tumors. This is consistent with a previous assumption that the number of cell cycles and thus, elevated cell cycling in cancer stem cells, may be associated with elevated TMB [31]. However, TMB-based correlative analysis hardly points to specific DNA mutagenic or repair-replicative processes with potential biological or clinical relevance. To cope with this issue, we deconvoluted the TMB into known, multiple mutation signatures and used their levels for the correlative analyses. Here, we focused on the expression and promoter DNA methylation of 270 DDR genes belonging to nine DNA damage-repair-replicative processes [22]. Along with *MLH1*, whose promoter hypermethylation and the resulting transcriptional downregulation lead to somatic MSI-H genotypes and the generation of mutations belonging to Sig.#6, we observed an additional relationship between Sig.#2-vs.-*NHEJ1* deficiency and Sig.#6-vs.-*ALKBH3* deficiency. In both cases, deficiencies in *NHEJ1* and *ALKBH3* alone did not increase mutations corresponding to Sig.#2 or Sig.#6. Instead, their deficiency is effective only in the genome with APOBEC overactivity and MMRd, suggesting that their potential mutagenic activity requires specific conditions. In addition to further validation, the list of DDR genes showing high levels of correlation may serve as potential candidates in the search of hypermutated cancer genomes with clinical benefits [8].

Currently, available mutation signature discovery methods are classified into two categories, e.g., those for “*de novo* ” mutation signature extraction and the others for “signature refitting” using known mutation signatures [34]. For the former, unsupervised NMF or its derivatives have been proposed to extract the *de novo* mutation signatures whose lineage specificity and potential causal association are investigated *post hoc* . However, it has been rarely discussed whether the mutation signatures can be derived in a supervised manner directly from phenotypic or genotypic scores of interests and can serve as a proxy to infer original scores. To test the feature-driven discovery of mutation signatures, we first demonstrated that Sig.#2, Sig.#6, and Sig.#10, known to be associated with APOBEC overexpression, MLH1 under-expression, and *POLE* mutations, could be derived using the associated genetic features, e.g., the differential trinucleotide frequencies between genomes with or without the causal features. Next, we used three SCNA-based scores representing HR deficiency to derive three mutation signatures, which were similar to Sig.#3 associated with *BRCA* deficiency. Of note, the levels of mutation signatures representing HR deficiency showed a concordance with the original SCNA-based HR deficiency scores, suggesting that the mutation profiles could be used to infer the level of HR deficiency, previously done by SCNA profiles. The use of quantitative features of somatic mutations to assess the nature of cancer genomes has been largely limited to TMB. However, our study demonstrated that the somatic mutations identified as mutation signatures could serve as cancer markers. The genomic alterations associated with tumor hypoxia have been previously reported, such as HR deficiency [24] and the deficiency of *TP53* [35] and *RAD53* [36]. Tumor hypoxia has been proposed to increase the mutation rates of cancer genomes with the downregulation of MMR genes [37], however, the impact of tumor hypoxia on tumor mutations is not well understood. This study showed that the majority of tumor hypoxia-driven mutation signatures resembled those of the APOBEC-related signatures of Sig.#2 and Sig.#13 in terms of trinucleotide frequencies. Along with the correlation between the transcript levels of APOBEC and tumor hypoxia scores, this observation suggests that tumor hypoxia is associated with APOBEC activity and that the somatic mutations in hypoxic tumor genomes may be largely attributed to the APOBEC-mediated C-to-T transitions. As in the case of the SCNA-driven HR deficiency scores, mRNA-based tumor hypoxia scores were concordant with the mutation signatures levels inferred from the mutation profiles of cancer genomes. This indicates that the mutation profiles can be used as proxies to infer various cancer genome-related features for mutation signature-based analysis. Although our exploratory study requires further validation in an extended, validation cohort, the potential of mutation signatures to derive cancer hallmark features, such as HR deficiency and tumor hypoxia, may be extended to mutation signature-based tumor markers with potential clinical relevance.

## Supplementary Materials

Supplemental Fig. 1. Relationship between APOBEC3A regulation and tumor mutation burden (TMB). TMB showed positive and inverse correlation with the expression of APOBEC3A (A) and methylation of *APOBEC3A* (B). This relationship is the opposite to those of *MLH1*, suggestive of a potential transcriptional regulatory mode of APOBEC3A by DNA promoter methylation.

Supplemental Fig. 2. Clustering of joint profiles of DNA damage and repair (DDR) gene methylation and mutation signature levels. (A) The promoter methylation levels of 254 DDR genes were merged with those of mutation signature levels and were subject to hierarchical clustering. Inlet shows that the methylation levels of *MLH1* and *ALKBH3* were segregated with the level of Sig.#6. (B) Scatter plots showing the level of correlation of gene methylation (*x*-axis) and expression (*y*-axis) with the level of Sig.#6 highlighting the *MLH1* and *ALKBH3* deficiencies. (C) The correlation of MLH1 expression and methylation levels are shown in a scatter plot (red-dots for MLH-H cases). (D) Similarly shown for ALKBH3.

Supplemental Fig. 3. Clustering of joint profiles of DNA damage and repair (DDR) gene expression and mutation signature levels. (A) The expression level of 254 DDR genes was merged with those of mutation signature levels estimated from 5617 the Cancer Genome Atlas (TCGA) samples and wassubject to hierarchical clustering. Inlet shows that NHEJ1 expression was segregated with the level of Sig.#2 and Sig.#13 associated with APOBEC overactivity. (B) Scatter plots showing the level of NHEJ1 expression levels and the level of Sig.#2 and Sig.#13, respectively.

Supplemental Fig. 4. The relationship between the homologous recombination (HR) deficiency scores and levels of the corresponding mutation signatures. For three types of scores representing the HR deficiency (number of telomeric allelic imbalance [NtAI], large scale transition [LST], homologous recombination deficiency–loss of heterozygosity [HRD-LOH] in the top, middle and bottom panels, respectively), the HR deficiency scores (*x*-axis) are shown against the level of mutation signatures derived of the corresponding scores (*y*-axis). Overall positive and negative relationships are observed for mutation signatures corresponding to the positive and negative differentials (Pos and Neg, respectively; left and right). The correlation levels are shown in the main Fig. 3D.

Supplemental Table 1. Molecular functions enriched for high tumor mutation burden (TMB). Preranked gene set enrichment analysis (GSEA) results using the correlation levels of individual genes with TMB are shown. The number of genes in the Gene Ontology (SIZE) and other results are shown as output of GSEA. Enrichment score (ES) and normalized ES (NES) are shown with significance levels. The significance level of zero indicates < 0.001.

Supplemental Table 2. Molecular functions enriched for low tumor mutation burden (TMB). Preranked gene set enrichment analysis results using the correlation levels of individual genes with TMB. Similarly shown with Supplementary Table 1.

Supplemental Table 3. Correlation table of DNA damage and repair gene expression and mutation signature levels

Supplemental Table 4. Correlation table of DNA damage and repair gene methylation and mutation signature levels
