# Supplementary material for: Correlation-based and feature-driven mutation signature analyses to identify genetic features associated with DNA mutagenic processes in cancer genomes
Source: Genomics Inform. 2021 Dec 31;19(4):e40. doi: 10.5808/gi.21047 (PMC8752981; doi:10.5808/gi.21047)
Supplement: Supplemental Fig. 1. — Relationship between APOBEC3A regulation and tumor mutation burden (TMB). TMB showed positive and inverse correlation with the expression of APOBEC3A (A) and methylation of APOBEC3A (B). This relationship is the opposite to those of MLH1, suggestive of a potential transcriptional regulatory mode of APOBEC3A by DNA promoter methylation. [file gi-21047suppl1.pdf]

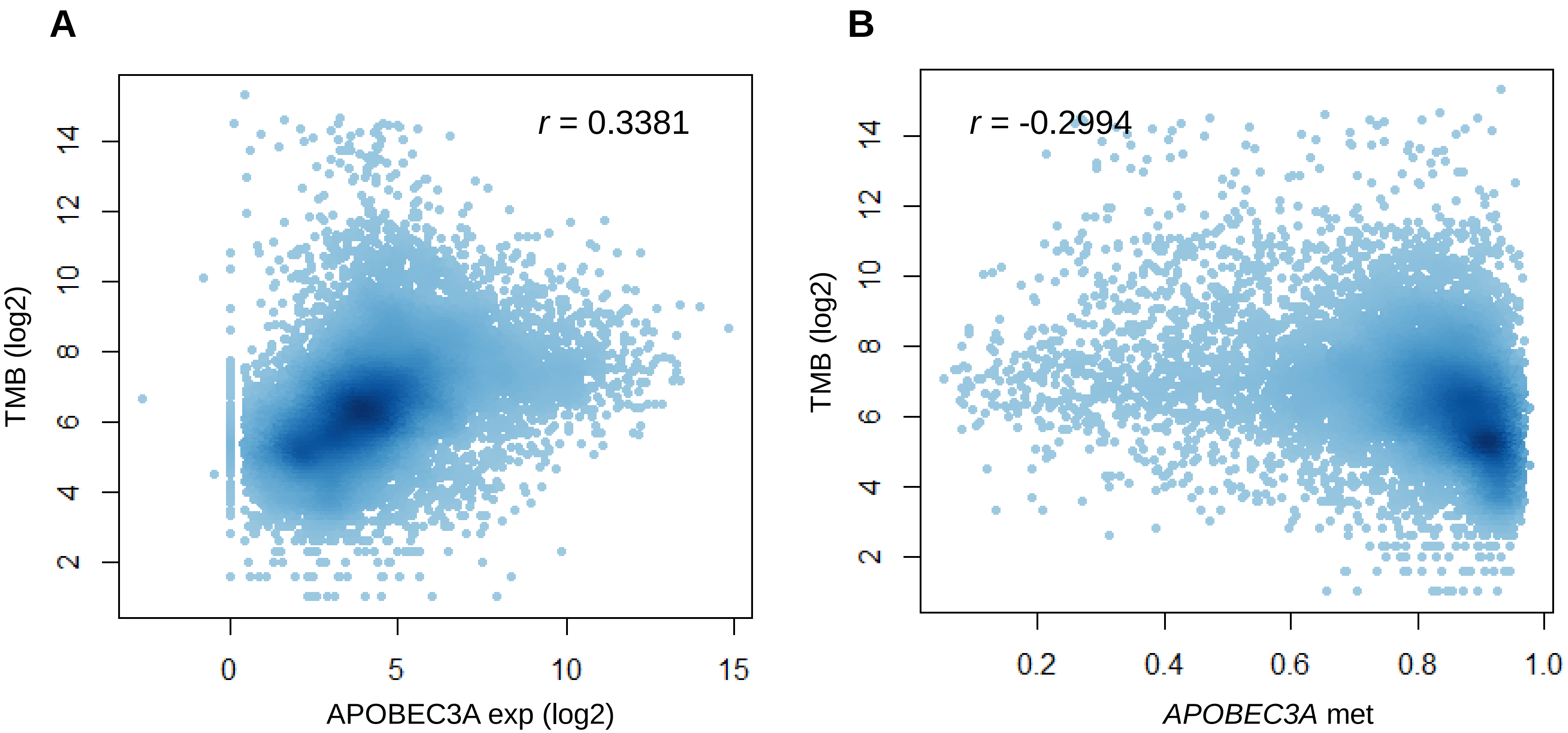

Supplementary Fig. 1. Relationship between APOBEC3A regulation and tumor mutation burden (TMB). TMB showed positive and inverse correlation with the expression of APOBEC3A (A) and methylation of APOBEC3A (B). This relationship is the opposite to those of MLH1, suggestive of a potential transcriptional regulatory mode of APOBEC3A by DNA promoter methylation.
