# Supplementary material for: Correlation-based and feature-driven mutation signature analyses to identify genetic features associated with DNA mutagenic processes in cancer genomes
Source: Genomics Inform. 2021 Dec 31;19(4):e40. doi: 10.5808/gi.21047 (PMC8752981; doi:10.5808/gi.21047)
Supplement: Supplemental Fig. 2. — Clustering of joint profiles of DNA damage and repair (DDR) gene methylation and mutation signature levels. (A) The promoter methylation levels of 254 DDR genes were merged with those of mutation signature levels and were subject to hierarchical clustering. Inlet shows that the methylation levels of MLH1 and ALKBH3 were segregated with the level of Sig.#6. (B) Scatter plots showing the level of correlation of gene methylation (x-axis) and expression (y-axis) with the level of Sig.#6 highlighting the MLH1 and ALKBH3 deficiencies. (C) The correlation of MLH1 expression and methylation levels are shown in a scatter plot (red-dots for MLH-H cases). (D) Similarly shown for ALKBH3. [file gi-21047suppl2.pdf]

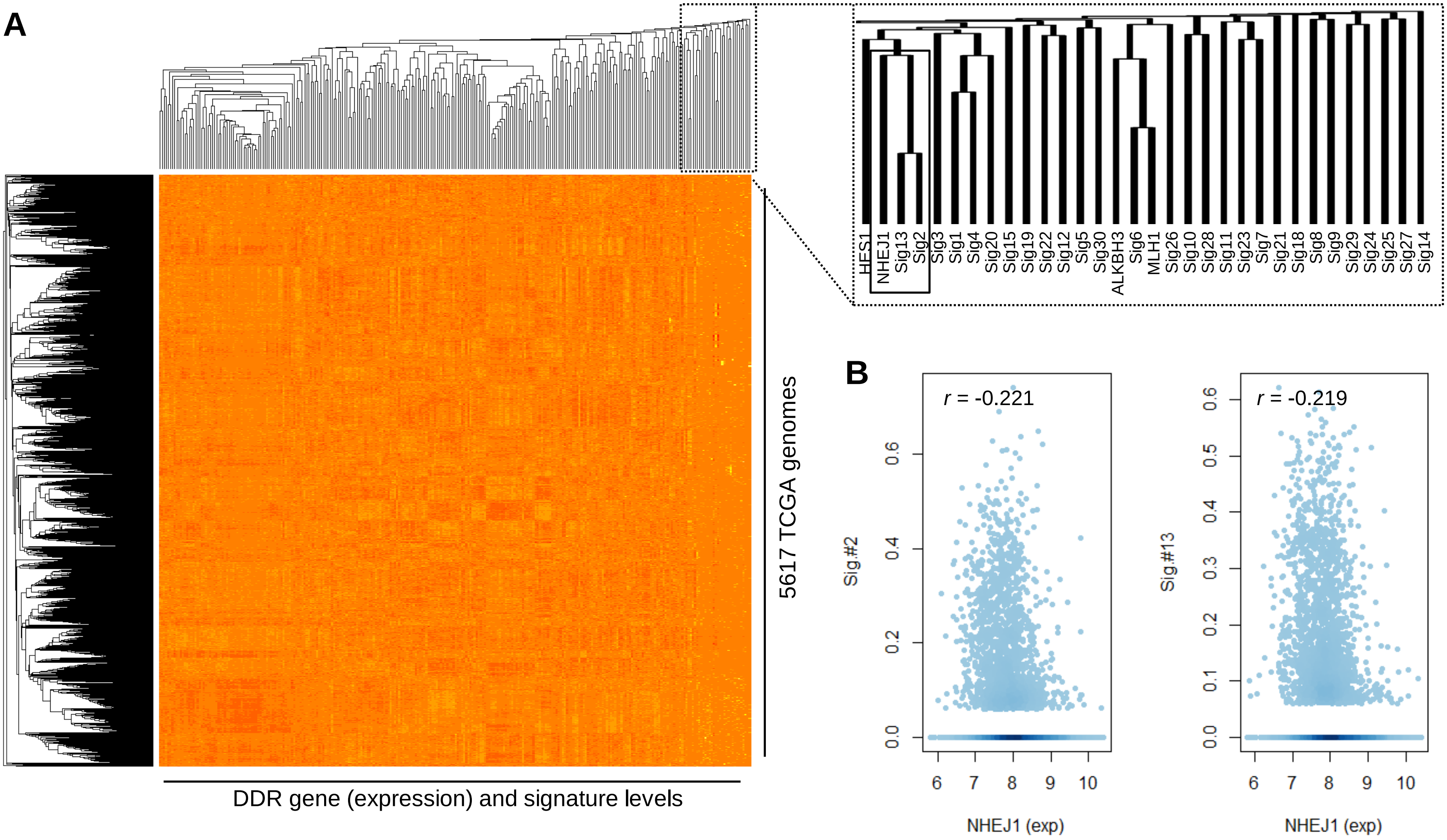

Supplementary Fig. 2. Clustering of joint profiles of DNA damage and repair (DDR) gene expression and mutation signature levels. (A) The expression level of 254 DDR genes was merged with those of mutation signature levels estimated from 5617 the Cancer Genome Atlas (TCGA) samples and wassubject to hierarchical clustering. Inlet shows that NHEJ1 expression was segregated with the level of Sig.#2 and Sig.#13 associated with APOBEC overactivity. (B) Scatter plots showing the level of NHEJ1 expression levels and the level of Sig.#2 and Sig.#13, respectively.
