# Supplementary material for: Correlation-based and feature-driven mutation signature analyses to identify genetic features associated with DNA mutagenic processes in cancer genomes
Source: Genomics Inform. 2021 Dec 31;19(4):e40. doi: 10.5808/gi.21047 (PMC8752981; doi:10.5808/gi.21047)
Supplement: Supplemental Fig. 4. — The relationship between the homologous recombination (HR) deficiency scores and levels of the corresponding mutation signatures. For three types of scores representing the HR deficiency (number of telomeric allelic imbalance [NtAI], large scale transition [LST], homologous recombination deficiency–loss of heterozygosity [HRD-LOH] in the top, middle and bottom panels, respectively), the HR deficiency scores (x-axis) are shown against the level of mutation signatures derived of the corresponding scores (y-axis). Overall positive and negative relationships are observed for mutation signatures corresponding to the positive and negative differentials (Pos and Neg, respectively; left and right). The correlation levels are shown in the main Fig. 3D. [file gi-21047suppl4.pdf]

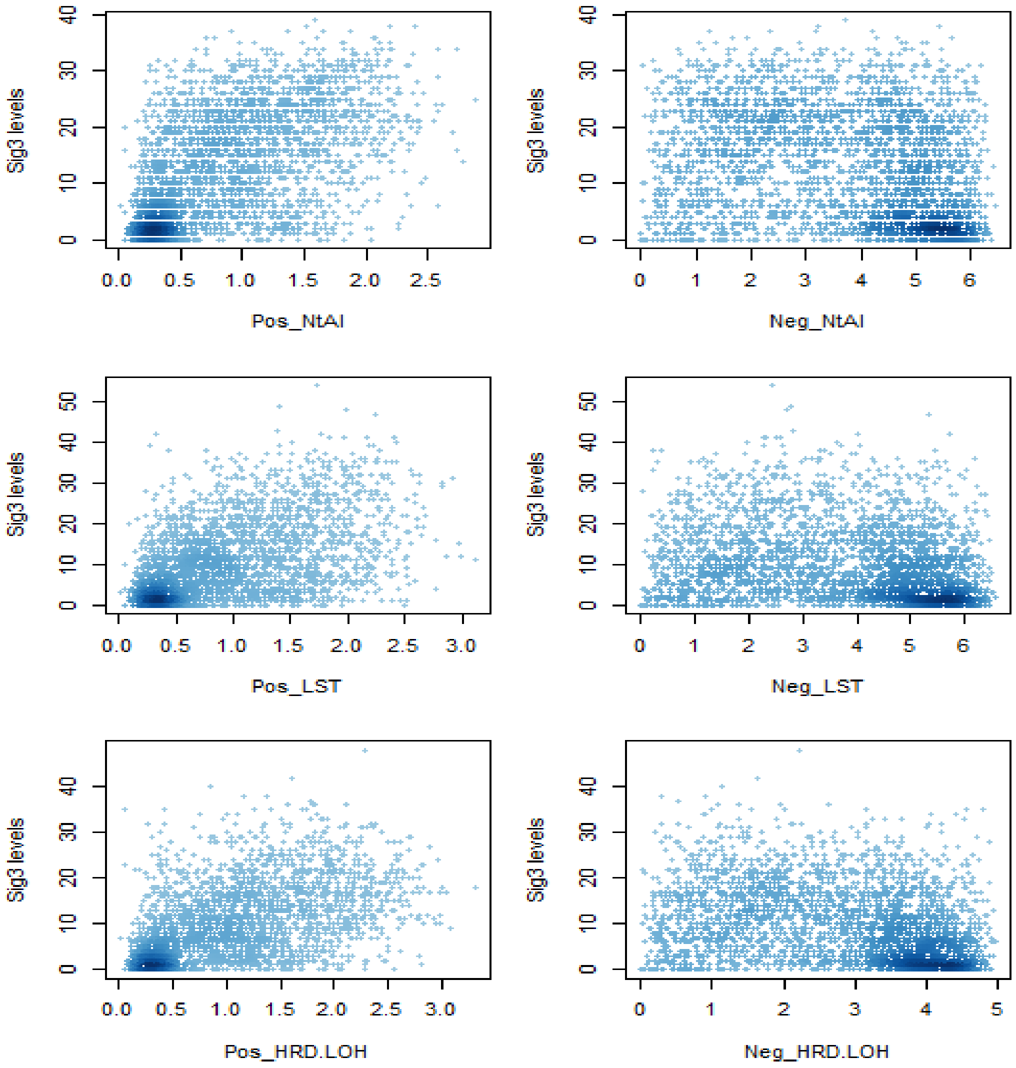

Supplementary Fig. 4. The relationship between the homologous recombination (HR) deficiency scores and levels of the corresponding mutation signatures. For three types of scores representing the HR deficiency (number of telomeric allelic imbalance [NtAI], large scale transition [LST], homologous recombination deficiency–loss of heterozygosity [HRD-LOH] in the top, middle and bottom panels, respectively), the HR deficiency scores (x-axis) are shown against the level of mutation signatures derived of the corresponding scores (y-axis). Overall positive and negative relationships are observed for mutation signatures corresponding to the positive and negative differentials (Pos and Neg, respectively; left and right). The correlation levels are shown in the main Fig. 3D.
