# Supplementary material for: Correlation-based and feature-driven mutation signature analyses to identify genetic features associated with DNA mutagenic processes in cancer genomes
Source: Genomics Inform. 2021 Dec 31;19(4):e40. doi: 10.5808/gi.21047 (PMC8752981; doi:10.5808/gi.21047)
Supplement: Supplemental Table 1. — Molecular functions enriched for high tumor mutation burden (TMB). Preranked gene set enrichment analysis (GSEA) results using the correlation levels of individual genes with TMB are shown. The number of genes in the Gene Ontology (SIZE) and other results are shown as output of GSEA. Enrichment score (ES) and normalized ES (NES) are shown with significance levels. The significance level of zero indicates < 0.001. [file gi-21047suppl5.pdf]

Supplementary Table 1. Molecular functions enriched for high tumor mutation burden (TMB)

Gene Ontology terms (MSigDB, c5)	SIZE	ES	NES	NOM p-val	FDR q-val	FWER p-va
GO_SISTER_CHROMATID_SEGREGATION	170	0.68	3.45	0	0	0
GO_CONDENSED_CHROMOSOME_CENTROMERIC_REGION	94	0.74	3.38	0	0	0
GO_SISTER_CHROMATID_COHESION	108	0.71	3.34	0	0	0
GO_NUCLEAR_CHROMOSOME_SEGREGATION	218	0.64	3.31	0	0	0
GO_CHROMOSOME_SEGREGATION	259	0.62	3.3	0	0	0
GO_CONDENSED_CHROMOSOME	183	0.64	3.24	0	0	0
GO_KINETOCHORE	111	0.68	3.24	0	0	0
GO_CHROMOSOME_CENTROMERIC_REGION	164	0.65	3.21	0	0	0
GO_MITOTIC_RECOMBINATION	39	0.81	3.15	0	0	0
GO_MITOTIC_SISTER_CHROMATID_SEGREGATION	87	0.69	3.12	0	0	0
GO_ANAPHASE_PROMOTING_COMPLEX_DEPENDENT_CATABOLIC_PROC	77	0.7	3.08	0	0	0
GO_MITOTIC_NUCLEAR_DIVISION	347	0.56	3.08	0	0	0
GO_DNA_DEPENDENT_DNA_REPLICATION	91	0.67	3.06	0	0	0
GO_CHROMOSOMAL_REGION	310	0.55	2.99	0	0	0
GO_CELL_CYCLE_G1_S_PHASE_TRANSITION	104	0.64	2.96	0	0	0
GO_TELOMERE_MAINTENANCE_VIA_RECOMBINATION	32	0.83	2.96	0	0	0
GO_DNA_REPLICATION_INITIATION	24	0.86	2.93	0	0	0
GO_CENTROMERE_COMPLEX_ASSEMBLY	45	0.73	2.91	0	0	0
GO_ANTIGEN_PROCESSING_AND_PRESENTATION_OF_EXOGENOUS_PEPTIDE_ANTIGEN_VIA_MHC_CLASS_I	66	0.69	2.91	0	0	0
GO_ORGANELLE_FISSION	474	0.51	2.89	0	0	0
GO_DNA_REPLICATION	195	0.56	2.88	0	0	0
GO_CELL_DIVISION	434	0.51	2.87	0	0	0
GO_TUMOR_NECROSIS_FACTOR_MEDIATED_SIGNALING_PATHWAY	118	0.61	2.85	0	0	0
GO_ANTIGEN_PROCESSING_AND_PRESENTATION_OF_PEPTIDE_ANTIGEN_VIA_MHC_CLASS_I	91	0.64	2.83	0	0	0
GO_REPLICATION_FORK	58	0.68	2.8	0	0	0
GO_DNA_STRAND_ELONGATION	30	0.78	2.75	0	0	0
GO_NIK_NF_KAPPAB_SIGNALING	83	0.61	2.75	0	0	0
GO_DNA_STRAND_ELONGATION_INVOLVED_IN_DNA_REPLICATION	25	0.8	2.74	0	0	0
GO_REGULATION_OF_CHROMOSOME_SEGREGATION	85	0.61	2.73	0	0	0
GO_CELL_CYCLE_CHECKPOINT	191	0.53	2.72	0	0	0
GO_PROTEASOME_COMPLEX	74	0.62	2.69	0	0	0
GO_KERATINOCYTE_DIFFERENTIATION	98	0.59	2.68	0	0	0
GO_POSITIVE_REGULATION_OF_LIGASE_ACTIVITY	109	0.57	2.67	0	0	0
GO_PROTEASOME_ACCESSORY_COMPLEX	24	0.78	2.66	0	0	0
GO_ACTIVATION_OF_INNATE_IMMUNE_RESPONSE	202	0.51	2.65	0	0	0
GO_REGULATION_OF_RNA_STABILITY	139	0.54	2.65	0	0	0
GO_CHROMOSOME_LOCALIZATION	59	0.62	2.64	0	0	0
GO_PRERIBOSOME	58	0.63	2.64	0	0	0
GO_RIBOSOME_BIOGENESIS	295	0.49	2.63	0	0	0
GO_CELL_CYCLE_PHASE_TRANSITION	242	0.5	2.63	0	0	0
GO_MITOTIC_SPINDLE_ORGANIZATION	68	0.61	2.63	0	0	0
GO_REGULATION_OF_PROTEIN_UBIQUITINATION_INVOLVED_IN_UBIQUITIN_DEPENDENT_PROTEIN_CATABOLIC_PROCESS	103	0.56	2.63	0	0	0
GO_POSITIVE_REGULATION_OF_INNATE_IMMUNE_RESPONSE	244	0.49	2.6	0	0	0
GO_KERATINIZATION	49	0.65	2.6	0	0	0
GO_DNA_HELICASE_ACTIVITY	52	0.63	2.6	0	0	0
GO_DNA_REPLICATION_INDEPENDENT_NUCLEOSOME_ORGANIZATION	50	0.64	2.6	0	0	0
GO_DNA_RECOMBINATION	196	0.51	2.59	0	0	0
GO_CONDENSED_NUCLEAR_CHROMOSOME_CENTROMERIC_REGION	16	0.85	2.58	0	0	0
GO_METAPHASE_PLATE_CONGRESSION	40	0.67	2.58	0	0	0
GO_SPINDLE_POLE	117	0.55	2.58	0	0	0
GO_CORNIFIED_ENVELOPE	43	0.68	2.58	0	0	0
GO_DNA_CONFORMATION_CHANGE	257	0.49	2.58	0	0	0
GO_REGULATION_OF_LIGASE_ACTIVITY	129	0.53	2.57	0	0	0
GO_NEGATIVE_REGULATION_OF_PROTEIN_MODIFICATION_BY_SMALL_PROTEIN_CONJUGATION_OR_REMOVAL	137	0.53	2.56	0	0	0
GO_CYTOKINE_MEDIATED_SIGNALING_PATHWAY	446	0.46	2.56	0	0	0
GO_REGULATION_OF_INNATE_IMMUNE_RESPONSE	351	0.47	2.55	0	0	0
GO_RRNA_METABOLIC_PROCESS	245	0.48	2.55	0	0	0
GO_RESPONSE_TO_INTERFERON_GAMMA	141	0.52	2.55	0	0	0
GO_ATP_DEPENDENT_CHROMATIN_REMODELING	71	0.59	2.54	0	0	0
GO_NCRNA_PROCESSING	372	0.46	2.54	0	0	0
GO_REGULATION_OF_TRANSCRIPTION_INVOLVED_IN_G1_S_TRANSITION_OF_MITOTIC_CELL_CYCLE	25	0.74	2.54	0	0	0
GO_SPINDLE	268	0.48	2.53	0	0	0
GO_TELOMERE_ORGANIZATION	98	0.55	2.53	0	0	0
GO_NUCLEOLAR_PART	62	0.6	2.53	0	0	0
GO_CELLULAR_RESPONSE_TO_INTERFERON_GAMMA	119	0.53	2.52	0	0	0
GO_RESPONSE_TO_TUMOR_NECROSIS_FACTOR	232	0.48	2.52	0	0	0
GO_EPIDERMAL_CELL_DIFFERENTIATION	138	0.51	2.52	0	0	0
GO_DNA_BIOSYNTHETIC_PROCESS	112	0.53	2.52	0	0	0
GO_CONDENSED_NUCLEAR_CHROMOSOME	80	0.57	2.51	0	0	0
GO_MITOTIC_CELL_CYCLE_CHECKPOINT	138	0.51	2.51	0	0	0
GO_MITOCHONDRIAL_TRANSLATION	105	0.53	2.5	0	0	0
GO_POSITIVE_REGULATION_OF_DEFENSE_RESPONSE	361	0.46	2.5	0	0	0
GO_HISTONE_EXCHANGE	50	0.63	2.5	0	0	0
GO_MULTI_ORGANISM_LOCALIZATION	67	0.58	2.49	0	0	0
GO_REGULATION_OF_DNA_DEPENDENT_DNA_REPLICATION	41	0.63	2.49	0	0	0

GO_REGULATION_OF_CELL_CYCLE_PHASE_TRANSITION	317	0.45	2.48	0	0	0
GO_RNA_PHOSPHODIESTER_BOND_HYDROLYSIS	112	0.53	2.48	0	0	0
GO_RIBONUCLEOPROTEIN_COMPLEX_LOCALIZATION	105	0.53	2.47	0	0	0
GO_RIBONUCLEOPROTEIN_COMPLEX_BIOGENESIS	415	0.44	2.46	0	0	0
GO_MEIOTIC_CELL_CYCLE_PROCESS	145	0.49	2.46	0	0	0
GO_RESPONSE_TO_IONIZING_RADIATION	140	0.5	2.46	0	0	0
GO_NUCLEAR_REPLICATION_FORK	37	0.65	2.45	0	0	0
GO_NUCLEAR_CHROMOSOME	494	0.43	2.44	0	0	0
GO_CHEMOKINE_RECEPTOR_BINDING	57	0.58	2.44	0	0	0
GO_MITOTIC_SPINDLE_ASSEMBLY	40	0.63	2.44	0	0	0
GO_REGULATION_OF_CELLULAR_AMINO_ACID_METABOLIC_PROCESS	66	0.57	2.43	0	0	0
GO_CHRONIC_INFLAMMATORY_RESPONSE	15	0.8	2.43	0	0	0
GO_INNATE_IMMUNE_RESPONSE_ACTIVATING_CELL_SURFACE_RECEPTOR_SIGNALING_PATHWAY	105	0.52	2.43	0	0	0
GO_DNA_PACKAGING	179	0.48	2.42	0	0	0
GO_SPINDLE_MIDZONE	27	0.7	2.42	0	0	0
GO_MATURATION_OF_5_8S_RRNA	28	0.68	2.42	0	0	0
GO_DNA_INTEGRITY_CHECKPOINT	143	0.49	2.41	0	0	0
GO_MEMBRANE_DISASSEMBLY	46	0.61	2.41	0	0	0
GO_DNA_PACKAGING_COMPLEX	98	0.51	2.4	0	0	0
GO_90S_PRERIBOSOME	23	0.73	2.4	0	0	0
GO_REGULATION_OF_NUCLEAR_DIVISION	159	0.48	2.39	0	0	0
GO_SPINDLE_CHECKPOINT	25	0.7	2.39	0	0	0
GO_TRNA_TRANSPORT	34	0.64	2.38	0	0	0.001
GO_EPIDERMIS_DEVELOPMENT	249	0.45	2.38	0	0	0.001
GO_DESMOSOME	25	0.7	2.38	0	0	0.001
GO_SKIN_DEVELOPMENT	208	0.46	2.38	0	0	0.001
GO_NUCLEAR_PORE	76	0.55	2.38	0	0	0.001
GO_REGULATION_OF_SISTER_CHROMATID_SEGREGATION	67	0.56	2.38	0	0	0.001
GO_REGULATION_OF_INTERFERON_GAMMA_PRODUCTION	93	0.52	2.37	0	0	0.001
GO_CHEMOKINE_ACTIVITY	48	0.6	2.37	0	0	0.001
GO_DNA_DEPENDENT_ATPASE_ACTIVITY	76	0.55	2.37	0	0	0.001
GO_RESPONSE_TO_TYPE_I_INTERFERON	68	0.55	2.37	0	0	0.001
GO_ORGANELLAR_RIBOSOME	72	0.55	2.37	0	0	0.001
GO_PEPTIDE_CROSS_LINKING	54	0.58	2.36	0	0	0.001
GO_ANTIGEN_PROCESSING_AND_PRESENTATION	209	0.46	2.36	0	0	0.001
GO_INTERSTRAND_CROSS_LINK_REPAIR	39	0.62	2.36	0	0	0.001
GO_DNA_GEOMETRIC_CHANGE	80	0.53	2.35	0	0	0.001
GO_ACTIVATION_OF_IMMUNE_RESPONSE	388	0.43	2.35	0	0	0.001
GO_REGULATION_OF_MITOTIC_CELL_CYCLE	459	0.42	2.35	0	0	0.001
GO_RIBOSOMAL_SMALL_SUBUNIT_BIOGENESIS	56	0.57	2.35	0	0	0.001
GO_REGULATION_OF_T_CELL_PROLIFERATION	144	0.48	2.34	0	0	0.001
GO_POSITIVE_REGULATION_OF_DNA_METABOLIC_PROCESS	179	0.45	2.34	0	0	0.001
GO_POSITIVE_REGULATION_OF_CELL_CYCLE_PROCESS	243	0.44	2.34	0	0	0.001
GO_ADAPTIVE_IMMUNE_RESPONSE	252	0.44	2.34	0	0	0.001
GO_HISTONE_KINASE_ACTIVITY	19	0.74	2.34	0	0	0.002
GO_RNA_PHOSPHODIESTER_BOND_HYDROLYSIS_ENDONUCLEOLYTIC	55	0.57	2.34	0	0	0.002
GO_T_CELL_RECEPTOR_SIGNALING_PATHWAY	141	0.48	2.33	0	0	0.002
GO_MATURATION_OF_5_8S_RRNA_FROM_TRICISTRONIC_RRNA_TRANSCRIPT_SSU_RRNA_5_8S_RRNA_LSU_RRNA_	19	0.74	2.33	0	0	0.002
GO_REGULATION_OF_INTERLEUKIN_12_PRODUCTION	51	0.57	2.33	0	0	0.002
GO_DNA_REPAIR	461	0.41	2.32	0	0	0.002
GO_ANTIGEN_PROCESSING_AND_PRESENTATION_OF_PEPTIDE_ANTIGE	173	0.46	2.32	0	0	0.002
GO_RESPONSE_TO_VIRUS	244	0.44	2.32	0	0	0.002
GO_G1_DNA_DAMAGE_CHECKPOINT	72	0.53	2.32	0	0	0.002
GO_LYMPHOCYTE_CHEMOTAXIS	38	0.61	2.32	0	0	0.002
GO_DNA_SYNTHESIS_INVOLVED_IN_DNA_REPAIR	71	0.54	2.32	0	0	0.002
GO_EXONUCLEASE_ACTIVITY_ACTIVE_WITH_EITHER_RIBO_OR_DEOXYRIBONUCLEIC_ACIDS_AND_PRODUCING_5_PHOSPHOMONOESTERS	46	0.59	2.32	0	0	0.002
GO_ANTIGEN_RECEPTOR_MEDIATED_SIGNALING_PATHWAY	170	0.46	2.31	0	0	0.002
GO_CLEAVAGE_INVOLVED_IN_RRNA_PROCESSING	18	0.73	2.31	0	0	0.002
GO_TRANSLATIONAL_TERMINATION	92	0.51	2.3	0	0	0.002
GO_REPLISOME	28	0.65	2.3	0	0	0.002
GO_SIGNAL_TRANSDUCTION_IN_RESPONSE_TO_DNA_DAMAGE	95	0.5	2.3	0	0	0.002
GO_SMALL_SUBUNIT_PROCESSOME	32	0.63	2.3	0	0	0.002
GO_ENDONUCLEASE_ACTIVITY_ACTIVE_WITH_EITHER_RIBO_OR_DEOXYRIBONUCLEIC_ACIDS_AND_PRODUCING_5_PHOSPHOMONOESTERS	29	0.63	2.3	0	0	0.002
GO_DEFENSE_RESPONSE_TO_OTHER_ORGANISM	465	0.4	2.3	0	0	0.003
GO_THREONINE_TYPE_PEPTIDASE_ACTIVITY	21	0.7	2.29	0	0	0.003
GO_VIRUS_RECEPTOR_ACTIVITY	70	0.52	2.29	0	0	0.003
GO_T_CELL_RECEPTOR_COMPLEX	19	0.71	2.29	0	0	0.004
GO_NUCLEAR_EXPORT	133	0.48	2.29	0	0	0.004
GO_MEIOTIC_CELL_CYCLE	179	0.45	2.29	0	0	0.004
GO_TRANSLATIONAL_ELONGATION	110	0.48	2.29	0	0	0.004
GO_GENE_SILENCING_BY_RNA	135	0.47	2.28	0	0	0.005
GO_KERATIN_FILAMENT	84	0.51	2.28	0	0	0.005
GO_GLYCERALDEHYDE_3_PHOSPHATE_METABOLIC_PROCESS	18	0.73	2.28	0	0	0.005
GO_PATTERN_RECOGNITION_RECEPTOR_SIGNALING_PATHWAY	109	0.48	2.28	0	0	0.005
GO_NEGATIVE_REGULATION_OF_CELL_CYCLE_PHASE_TRANSITION	145	0.46	2.28	0	0	0.005
GO_NUCLEAR_CHROMOSOME_TELOMERIC_REGION	121	0.47	2.28	0	0	0.006
GO_NUCLEIC_ACID_PHOSPHODIESTER_BOND_HYDROLYSIS	242	0.43	2.28	0	0	0.009
GO_SPINDLE_ASSEMBLY	68	0.52	2.28	0	0	0.009
GO_PROTEIN_LOCALIZATION_TO_CHROMOSOME	44	0.58	2.27	0	0	0.01
GO_STRAND_DISPLACEMENT	23	0.67	2.27	0	0	0.012
GO_CELL_DIFFERENTIATION_INVOLVED_IN_EMBRYONIC_PLACENTA_DEVELOPMENT	25	0.66	2.26	0	0	0.013
GO_MATURATION_OF_SSU_RRNA	40	0.6	2.26	0	0	0.013

GO_REGULATION_OF_TYPE_I_INTERFERON_PRODUCTION	109	0.48	2.26	0	0	0.014
GO_REGULATION_OF_ESTABLISHMENT_OF_PLANAR_POLARITY	110	0.48	2.26	0	0	0.014
GO_REGULATION_OF_CYTOKINESIS	62	0.53	2.26	0	0	0.014
GO_PRONUCLEUS	15	0.77	2.26	0	0	0.014
GO_PRERIBOSOME_LARGE_SUBUNIT_PRECURSOR	20	0.69	2.26	0	0	0.016
GO_REGULATION_OF_CENTROSOME_CYCLE	36	0.61	2.26	0	0	0.016
GO_EXONUCLEASE_ACTIVITY	79	0.51	2.26	0	0	0.018
GO_POSITIVE_REGULATION_OF_T_CELL_PROLIFERATION	95	0.5	2.26	0	0	0.018
GO_CENTROSOME_CYCLE	44	0.57	2.25	0	0	0.019
GO_POSITIVE_REGULATION_OF_CYTOKINESIS	35	0.6	2.25	0	0	0.022
GO_NEGATIVE_REGULATION_OF_EXTRINSIC_APOPTOTIC_SIGNALING_PATHWAY_VIA_DEATH_DOMAIN_RECEPTORS	34	0.6	2.25	0	0	0.022
GO_POSITIVE_REGULATION_OF_PROTEIN_MODIFICATION_BY_SMALL_PROTEIN_CONJUGATION_OR_REMOVAL	194	0.44	2.25	0	0	0.022
GO_MITOTIC_DNA_INTEGRITY_CHECKPOINT	99	0.49	2.25	0	0	0.022
GO_TRNA_METABOLIC_PROCESS	171	0.46	2.25	0	0	0.022
GO_MIDBODY	124	0.47	2.25	0	0	0.022
GO_REGULATION_OF_DNA_REPLICATION	157	0.46	2.25	0	0	0.022
GO_NUCLEOTIDE_EXCISION_REPAIR_DNA_GAP_FILLING	24	0.66	2.24	0	0	0.023
GO_DOUBLE_STRAND_BREAK_REPAIR	153	0.46	2.24	0	0	0.023
GO_REGULATION_OF_CELL_DIVISION	266	0.42	2.24	0	0	0.023
GO_DEOXYRIBONUCLEASE_ACTIVITY	62	0.52	2.24	0	0	0.023
GO_TERMINATION_OF_RNA_POLYMERASE_II_TRANSCRIPTION	50	0.55	2.24	0	0	0.023
GO_RECOMBINATIONAL_REPAIR	70	0.52	2.23	0	0	0.024
GO_DNA_TEMPLATED_TRANSCRIPTION_TERMINATION	89	0.49	2.23	0	0	0.024
GO_POSITIVE_REGULATION_OF_LEUKOCYTE_MIGRATION	109	0.48	2.23	0	0	0.024
GO_POSITIVE_REGULATION_OF_TYPE_I_INTERFERON_PRODUCTION	69	0.52	2.23	0	0	0.024
GO_ATP_DEPENDENT_DNA_HELICASE_ACTIVITY	33	0.61	2.23	0	0	0.024
GO_POSITIVE_REGULATION_OF_INTERLEUKIN_1_PRODUCTION	36	0.58	2.23	0	0	0.024
GO_CHROMOSOME_CONDENSATION	31	0.62	2.23	0	0	0.024
GO_POSITIVE_REGULATION_OF_CYTOKINE_PRODUCTION	365	0.4	2.22	0	0	0.024
GO_POSITIVE_REGULATION_OF_CELL_CELL_ADHESION	235	0.42	2.22	0	0	0.026
GO_DNA_DAMAGE_RESPONSE_DETECTION_OF_DNA_DAMAGE	36	0.59	2.21	0	0	0.034
GO_SNORNA_BINDING	25	0.64	2.21	0	0	0.035
GO_POSITIVE_REGULATION_OF_INTERFERON_GAMMA_PRODUCTION	64	0.52	2.21	0	0	0.037
GO_INFLAMMATORY_RESPONSE	445	0.39	2.21	0	0	0.037
GO_TRNA_PROCESSING	110	0.47	2.2	0	0	0.042
GO_REGULATION_OF_HOMOTYPIC_CELL_CELL_ADHESION	299	0.41	2.2	0	0	0.042
GO_MITOTIC_SPINDLE	54	0.53	2.2	0	0	0.043
GO_NADP_METABOLIC_PROCESS	28	0.63	2.2	0	0	0.043
GO_ENDORIBONUCLEASE_ACTIVITY_PRODUCING_5_PHOSPHOMONOEST	25	0.64	2.19	0	0	0.045
GO_DEFENSE_RESPONSE_TO_BACTERIUM	202	0.42	2.19	0	0	0.046
GO_EXODEOXYRIBONUCLEASE_ACTIVITY	18	0.71	2.19	0	0	0.046
GO_POSITIVE_REGULATION_OF_PROTEOLYSIS	359	0.4	2.19	0	0	0.047
GO_LYMPHOCYTE_MEDIATED_IMMUNITY	112	0.46	2.19	0	0	0.047
GO_SPINDLE_MICROTUBULE	58	0.52	2.18	0	0	0.048
GO_REGULATION_OF_DNA_METABOLIC_PROCESS	330	0.4	2.18	0	0	0.048
GO_NEGATIVE_REGULATION_OF_CELL_DIVISION	60	0.52	2.18	0	0	0.048
GO_DEFENSE_RESPONSE_TO_VIRUS	161	0.44	2.18	0	0	0.048
GO_TUMOR_NECROSIS_FACTOR_RECEPTOR_BINDING	30	0.61	2.18	0	0	0.048
GO_NUCLEOID	45	0.55	2.18	0	0	0.048
GO_REGULATION_OF_CYSTEINE_TYPE_ENDOPEPTIDASE_ACTIVITY_INVOLVED_IN_APOPTOTIC_SIGNALING_PATHWAY	22	0.66	2.18	0	0	0.051
GO_MATURATION_OF_SSU_RRNA_FROM_TRICISTRONIC_RRNA_TRANSCRIPT_SSU_RRNA_5_8S_RRNA_LSU_RRNA_	31	0.61	2.18	0	0	0.051
GO_DNA_REPLICATION_DEPENDENT_NUCLEOSOME_ORGANIZATION	31	0.6	2.18	0	0	0.051
GO_MICROTUBULE_ORGANIZING_CENTER_ORGANIZATION	83	0.48	2.18	0	0	0.051
GO_GLUCOSE_6_PHOSPHATE_METABOLIC_PROCESS	22	0.65	2.17	0	0	0.059
GO_ENDORIBONUCLEASE_COMPLEX	20	0.67	2.17	0	0	0.059
GO_MHC_PROTEIN_BINDING	24	0.64	2.17	0	0	0.06
GO_MONOCYTE_CHEMOTAXIS	41	0.57	2.17	0	0	0.06
GO_RNA_LOCALIZATION	167	0.44	2.17	0	0	0.06
GO_RIBONUCLEASE_ACTIVITY	93	0.48	2.17	0	0	0.061
GO_NEGATIVE_REGULATION_OF_CELL_CYCLE_PROCESS	212	0.42	2.17	0	0	0.061
GO_PROTEIN_DNA_COMPLEX	162	0.44	2.17	0	0	0.063
GO_CELLULAR_RESPONSE_TO_INTERLEUKIN_1	87	0.48	2.16	0	0	0.065
GO_VIRAL_LIFE_CYCLE	287	0.4	2.16	0	0	0.065
GO_LEUKOCYTE_APOPTOTIC_PROCESS	22	0.65	2.16	0	0	0.066
GO_MOVEMENT_IN_ENVIRONMENT_OF_OTHER_ORGANISM_INVOLVED_IN_SYMBIOTIC_INTERACTION	87	0.48	2.16	0	0	0.066
GO_REGULATION_OF_SYMBIOSIS_ENCOMPASSING_MUTUALISM_THROUGH_PARASITISM	203	0.42	2.16	0	0	0.066
GO_CHROMATIN_ASSEMBLY_OR_DISASSEMBLY	162	0.43	2.16	0	0	0.066
GO_POSITIVE_REGULATION_OF_CYSTEINE_TYPE_ENDOPEPTIDASE_ACTIVITY_INVOLVED_IN_APOPTOTIC_SIGNALING_PATHWAY	17	0.69	2.16	0	0	0.066
GO_ORGANELLAR_SMALL_RIBOSOMAL_SUBUNIT	25	0.63	2.16	0	0	0.069
GO_REGULATION_OF_LEUKOCYTE_PROLIFERATION	202	0.42	2.15	0	0	0.07
GO__DE_NOVO_PROTEIN_FOLDING	18	0.68	2.15	0	0	0.07
GO_NUCLEOBASE_BIOSYNTHETIC_PROCESS	18	0.67	2.15	0	0	0.071
GO_REGULATION_OF_ADAPTIVE_IMMUNE_RESPONSE	123	0.45	2.15	0	0	0.071
GO_CHROMOSOME_TELOMERIC_REGION	151	0.44	2.15	0	0	0.075
GO_HELICASE_ACTIVITY	150	0.44	2.15	0	0	0.075
GO_CYTOSKELETON_DEPENDENT_CYTOKINESIS	39	0.56	2.15	0	0	0.075
GO_REGULATION_OF_ALPHA_BETA_T_CELL_PROLIFERATION	23	0.65	2.15	0	0	0.079
GO_CHEMOKINE_MEDIATED_SIGNALING_PATHWAY	72	0.5	2.15	0	0	0.08
GO_POSITIVE_REGULATION_OF_TRANSLATIONAL_INITIATION	22	0.65	2.15	0	0	0.08
GO_REGULATION_OF_INTERLEUKIN_1_BETA_PRODUCTION	47	0.53	2.14	0	0	0.082

GO_NEGATIVE_REGULATION_OF_CHROMOSOME_SEGREGATION	28	0.6	2.14	0	0	0.082
GO_OXIDOREDUCTION_COENZYME_METABOLIC_PROCESS	104	0.46	2.14	0	0	0.082
GO_RESPONSE_TO_INTERFERON_BETA	21	0.67	2.14	0	0.001	0.088
GO_REGULATION_OF_MICROTUBULE_BASED_PROCESS	234	0.41	2.14	0	0.001	0.09
GO_ANTIGEN_BINDING	79	0.48	2.13	0	0.001	0.09
GO_REGULATION_OF_TELOMERASE_RNA_LOCALIZATION_TO_CAJAL_BO	15	0.74	2.13	0	0.001	0.09
GO_CYTOKINESIS	80	0.48	2.13	0	0.001	0.098
GO_NUCLEASE_ACTIVITY	195	0.42	2.13	0	0.001	0.098
GO_REGULATION_OF_CELL_CELL_ADHESION	371	0.39	2.13	0	0.001	0.1
GO_REGULATION_OF_INTERLEUKIN_1_PRODUCTION	57	0.51	2.13	0	0.001	0.1
GO_NUCLEAR_ENVELOPE_ORGANIZATION	80	0.49	2.13	0	0.001	0.101
GO_REGULATION_OF_MICROTUBULE_POLYMERIZATION_OR_DEPOLYMERIZATION	173	0.43	2.13	0	0.001	0.105
GO_TUMOR_NECROSIS_FACTOR_RECEPTOR_SUPERFAMILY_BINDING	46	0.54	2.13	0	0.001	0.105
GO_REGULATION_OF_KERATINOCYTE_DIFFERENTIATION	26	0.63	2.12	0	0.001	0.109
GO_RESPONSE_TO_BACTERIUM	489	0.37	2.12	0	0.001	0.112
GO_SPINDLE_LOCALIZATION	38	0.56	2.12	0	0.001	0.112
GO_RESPONSE_TO_GAMMA_RADIATION	50	0.52	2.12	0	0.001	0.115
GO_ENDORIBONUCLEASE_ACTIVITY	47	0.53	2.12	0	0.001	0.116
GO_CYTOKINE_ACTIVITY	214	0.41	2.12	0	0.001	0.118
GO_POSITIVE_REGULATION_OF_IMMUNE_EFFECTOR_PROCESS	153	0.42	2.11	0	0.001	0.12
GO_POSITIVE_REGULATION_OF_LEUKOCYTE_MEDIATED_IMMUNITY	84	0.46	2.11	0	0.001	0.125
GO_LEUKOCYTE_CHEMOTAXIS	117	0.44	2.11	0	0.001	0.125
GO_REGULATION_OF_T_CELL_MIGRATION	24	0.63	2.11	0	0.001	0.126
GO_PROTEIN_SUMOYLATION	115	0.44	2.11	0	0.001	0.128
GO_POSITIVE_REGULATION_OF_LYMPHOCYTE_MIGRATION	26	0.61	2.11	0	0.001	0.132
GO_RESPONSE_TO_MOLECULE_OF_BACTERIAL_ORIGIN	318	0.39	2.11	0	0.001	0.134
GO_REGULATION_OF_LYMPHOCYTE_MEDIATED_IMMUNITY	113	0.45	2.11	0	0.001	0.138
GO_POSITIVE_REGULATION_OF_PEPTIDASE_ACTIVITY	152	0.42	2.1	0	0.001	0.142
GO_NEGATIVE_REGULATION_OF_VIRAL_PROCESS	88	0.47	2.1	0	0.001	0.142
GO_POSITIVE_REGULATION_OF_ADAPTIVE_IMMUNE_RESPONSE	73	0.48	2.1	0	0.001	0.142
GO_LYMPHOCYTE_MIGRATION	49	0.53	2.1	0	0.001	0.142
GO_REGULATION_OF_DNA_RECOMBINATION	58	0.5	2.1	0	0.001	0.143
GO_BLASTOCYST_DEVELOPMENT	61	0.5	2.1	0	0.001	0.148
GO_CYSTEINE_TYPE_ENDOPEPTIDASE_ACTIVITY_INVOLVED_IN_APOPTOTIC_PROCESS	15	0.7	2.1	0	0.001	0.148
GO_POSITIVE_REGULATION_OF_CELL_ACTIVATION	284	0.39	2.1	0	0.001	0.148
GO_ENDODEOXYRIBONUCLEASE_ACTIVITY	43	0.54	2.1	0	0.001	0.148
GO_MHC_PROTEIN_COMPLEX	25	0.61	2.1	0	0.001	0.156
GO_CXCR_CHEMOKINE_RECEPTOR_BINDING	16	0.69	2.1	0	0.001	0.161
GO_REGULATION_OF_PEPTIDASE_ACTIVITY	384	0.38	2.1	0	0.001	0.162
GO_POSITIVE_REGULATION_OF_LEUKOCYTE_CHEMOTAXIS	81	0.47	2.09	0	0.001	0.163
GO_IMMUNE_EFFECTOR_PROCESS	446	0.37	2.09	0	0.001	0.169
GO_INTERFERON_GAMMA_MEDIATED_SIGNALING_PATHWAY	68	0.49	2.09	0	0.001	0.169
GO_POSITIVE_REGULATION_OF_ALPHA_BETA_T_CELL_PROLIFERATION	19	0.65	2.09	0	0.001	0.17
GO_POSITIVE_REGULATION_OF_IMMUNOGLOBULIN_PRODUCTION	30	0.57	2.09	0	0.001	0.174
GO_CELLULAR_PROTEIN_COMPLEX_DISASSEMBLY	122	0.44	2.09	0	0.001	0.174
GO_REGULATION_OF_T_CELL_DIFFERENTIATION	106	0.45	2.09	0	0.001	0.174
GO_TRNA_SPECIFIC_RIBONUCLEASE_ACTIVITY	16	0.69	2.09	0	0.001	0.175
GO_REGULATION_OF_TRANSLATIONAL_INITIATION	80	0.46	2.09	0	0.001	0.175
GO_REGULATION_OF_WATER_LOSS_VIA_SKIN	19	0.67	2.09	0	0.001	0.179
GO_NEGATIVE_REGULATION_OF_PROTEIN_COMPLEX_DISASSEMBLY	168	0.42	2.08	0	0.001	0.182
GO_FC_RECEPTOR_SIGNALING_PATHWAY	183	0.41	2.08	0	0.001	0.182
GO_POSITIVE_REGULATION_OF_INTERLEUKIN_1_BETA_PRODUCTION	30	0.57	2.08	0	0.001	0.182
GO_FC_EPSILON_RECEPTOR_SIGNALING_PATHWAY	124	0.43	2.08	0	0.001	0.185
GO_POSITIVE_REGULATION_OF_CELL_CYCLE_ARREST	84	0.47	2.08	0	0.001	0.186
GO_GENE_SILENCING	201	0.41	2.08	0	0.001	0.188
GO_REGULATION_OF_PROTEIN_MODIFICATION_BY_SMALL_PROTEIN_CONJUGATION_OR_REMOVAL	277	0.39	2.08	0	0.001	0.188
GO_POSITIVE_REGULATION_OF_CYTOKINE_BIOSYNTHETIC_PROCESS	58	0.5	2.08	0	0.001	0.188
GO_POSITIVE_REGULATION_OF_CHROMOSOME_SEGREGATION	25	0.61	2.08	0	0.001	0.19
GO_REGULATION_OF_LEUKOCYTE_MIGRATION	148	0.42	2.08	0	0.001	0.19
GO_RNA_POLYMERASE_ACTIVITY	43	0.54	2.08	0	0.001	0.193
GO_NUCLEOTIDYLTRANSFERASE_ACTIVITY	122	0.43	2.08	0	0.001	0.194
GO_TELOMERASE_HOLOENZYME_COMPLEX	19	0.64	2.07	0	0.001	0.203
GO_INTERACTION_WITH_HOST	134	0.43	2.07	0	0.001	0.206
GO_ERROR_FREE_TRANSLESION_SYNTHESIS	19	0.66	2.07	0	0.001	0.206
GO_REGULATION_OF_CD4_POSITIVE_ALPHA_BETA_T_CELL_ACTIVATION	38	0.55	2.07	0	0.001	0.208
GO_REGULATION_OF_PROTEIN_COMPLEX_DISASSEMBLY	212	0.4	2.07	0	0.001	0.212
GO_POSITIVE_REGULATION_OF_GENE_EXPRESSION_EPIGENETIC	75	0.47	2.07	0	0.001	0.218
GO_POSITIVE_REGULATION_OF_CD4_POSITIVE_ALPHA_BETA_T_CELL_ACTIVATION	27	0.59	2.07	0	0.001	0.222
GO_ORGANELLAR_LARGE_RIBOSOMAL_SUBUNIT	32	0.55	2.07	0	0.001	0.222
GO_ZYMOGEN_ACTIVATION	110	0.45	2.07	0	0.001	0.225
GO_RNA_PHOSPHODIESTER_BOND_HYDROLYSIS_EXONUCLEOLYTIC	35	0.55	2.07	0	0.001	0.225
GO_MITOTIC_CYTOKINESIS	31	0.57	2.07	0	0.001	0.225
GO_POSITIVE_REGULATION_OF_DNA_REPLICATION	85	0.45	2.07	0	0.001	0.23
GO_REGULATION_OF_INTERLEUKIN_8_SECRETION	19	0.65	2.07	0	0.001	0.232
GO_REGULATION_OF_CELLULAR_AMINE_METABOLIC_PROCESS	88	0.47	2.06	0	0.001	0.244
GO_POSTTRANSCRIPTIONAL_REGULATION_OF_GENE_EXPRESSION	441	0.37	2.06	0	0.001	0.246
GO_NEGATIVE_REGULATION_OF_MITOTIC_CELL_CYCLE	198	0.4	2.06	0	0.001	0.249
GO_POSITIVE_REGULATION_OF_INTERLEUKIN_1_SECRETION	24	0.62	2.06	0	0.001	0.249
GO_REGULATION_OF_SIGNAL_TRANSDUCTION_BY_P53_CLASS_MEDIAT	159	0.41	2.06	0	0.001	0.25
GO_CELL_REDOX_HOMEOSTASIS	65	0.48	2.06	0	0.001	0.251
GO_REGULATION_OF_TOLERANCE_INDUCTION	17	0.66	2.06	0	0.001	0.257
GO_REGULATION_OF_EPIDERMIS_DEVELOPMENT	62	0.49	2.06	0	0.001	0.266
GO_POSITIVE_REGULATION_OF_ALPHA_BETA_T_CELL_ACTIVATION	51	0.5	2.05	0	0.001	0.27

GO_REGULATION_OF_CELL_CYCLE_CHECKPOINT	28	0.58	2.05	0.002	0.001	0.274
GO_U12_TYPE_SPLICEOSOMAL_COMPLEX	26	0.6	2.05	0	0.001	0.276
GO_SMALL_NUCLEOLAR_RIBONUCLEOPROTEIN_COMPLEX	20	0.64	2.05	0	0.001	0.277
GO_NEGATIVE_REGULATION_OF_INNATE_IMMUNE_RESPONSE	37	0.54	2.05	0	0.001	0.277
GO_T_CELL_APOPTOTIC_PROCESS	15	0.7	2.05	0	0.001	0.277
GO_3_5_EXONUCLEASE_ACTIVITY	50	0.51	2.05	0	0.001	0.28
GO_CAJAL_BODY	52	0.51	2.05	0	0.001	0.285
GO_LIPOPOLYSACCHARIDE_MEDIATED_SIGNALING_PATHWAY	31	0.57	2.05	0	0.001	0.286
GO_SMN_SM_PROTEIN_COMPLEX	16	0.67	2.05	0	0.001	0.286
GO_TRANSLESION_SYNTHESIS	41	0.53	2.05	0	0.001	0.288
GO_RIBOSOMAL_LARGE_SUBUNIT_BIOGENESIS	49	0.51	2.05	0	0.001	0.288
GO_REGULATION_OF_CELL_CYCLE_G1_S_PHASE_TRANSITION	145	0.42	2.04	0	0.001	0.303
GO_REGULATION_OF_RESPONSE_TO_INTERFERON_GAMMA	22	0.6	2.04	0	0.001	0.305
GO_POSITIVE_REGULATION_OF_EPIDERMIS_DEVELOPMENT	32	0.57	2.04	0.002	0.001	0.307
GO_REGULATION_OF_T_HELPER_1_TYPE_IMMUNE_RESPONSE	22	0.62	2.04	0	0.001	0.307
GO_MEIOTIC_CHROMOSOME_SEGREGATION	61	0.48	2.04	0	0.001	0.307
GO_POSITIVE_REGULATION_OF_DNA_BIOSYNTHETIC_PROCESS	58	0.49	2.04	0	0.002	0.309
GO_REGULATION_OF_ALPHA_BETA_T_CELL_ACTIVATION	68	0.48	2.04	0	0.002	0.309
GO_NUCLEOBASE_CONTAINING_COMPOUND_TRANSPORT	181	0.4	2.04	0	0.002	0.312
GO_SPLICEOSOMAL_COMPLEX	163	0.41	2.04	0	0.002	0.32
GO_PROTEIN_DNA_COMPLEX_SUBUNIT_ORGANIZATION	211	0.39	2.04	0	0.002	0.32
GO_POSITIVE_REGULATION_OF_CELL_CYCLE	327	0.37	2.04	0	0.002	0.32
GO_REGULATION_OF_CYSTEINE_TYPE_ENDOPEPTIDASE_ACTIVITY	211	0.39	2.04	0	0.002	0.321
GO_REGULATION_OF_LEUKOCYTE_MEDIATED_IMMUNITY	155	0.41	2.03	0	0.002	0.327
GO_POSITIVE_REGULATION_OF_CELLULAR_PROTEIN_CATABOLIC_PROC	192	0.4	2.03	0	0.002	0.327
GO_CYTOKINE_RECEPTOR_BINDING	265	0.38	2.03	0	0.002	0.328
GO_NUCLEAR_NUCLEOSOME	37	0.53	2.03	0	0.002	0.329
GO_REGULATION_OF_SUBSTRATE_ADHESION_DEPENDENT_CELL_SPREADING	43	0.52	2.03	0	0.002	0.33
GO_GRANULOCYTE_MIGRATION	75	0.45	2.03	0	0.002	0.332
GO_IMMUNE_RESPONSE_REGULATING_CELL_SURFACE_RECEPTOR_SIGNALING_PATHWAY	286	0.38	2.03	0	0.002	0.338
GO_ADAPTIVE_IMMUNE_RESPONSE_BASED_ON_SOMATIC_RECOMBINATION_OF_IMMUNE_RECEPTORS_BUILT_FROM_IMMUNOGLOBULIN_SUPERFAMILY_DOMAINS	121	0.43	2.03	0	0.002	0.342
GO_MITOCHONDRIAL_RNA_METABOLIC_PROCESS	29	0.57	2.03	0	0.002	0.345
GO_POSTREPLICATION_REPAIR	54	0.5	2.03	0	0.002	0.355
GO_NEGATIVE_REGULATION_OF_IMMUNE_RESPONSE	119	0.43	2.03	0	0.002	0.364
GO_REGULATION_OF_CYTOKINE_BIOSYNTHETIC_PROCESS	94	0.45	2.03	0	0.002	0.365
GO_REGULATION_OF_DOUBLE_STRAND_BREAK_REPAIR_VIA_HOMOLOGOUS_RECOMBINATION	19	0.64	2.03	0.002	0.002	0.365
GO_MEIOSIS_I	81	0.45	2.02	0	0.002	0.377
GO_POSITIVE_REGULATION_OF_PRODUCTION_OF_MOLECULAR_MEDIATOR_OF_IMMUNE_RESPONSE	62	0.48	2.02	0	0.002	0.379
GO_REGULATION_OF_LYMPHOCYTE_MIGRATION	37	0.54	2.02	0	0.002	0.38
GO_RNA_3_END_PROCESSING	86	0.45	2.02	0	0.002	0.383
GO_HISTONE_PHOSPHORYLATION	25	0.59	2.02	0	0.002	0.39
GO_NEGATIVE_REGULATION_OF_INTERLEUKIN_12_PRODUCTION	15	0.67	2.02	0.002	0.002	0.39
GO_ESTABLISHMENT_OF_MITOTIC_SPINDLE_LOCALIZATION	24	0.59	2.02	0	0.002	0.396
GO_POSITIVE_REGULATION_OF_MITOTIC_NUCLEAR_DIVISION	51	0.5	2.01	0	0.002	0.404
GO_CCR_CHEMOKINE_RECEPTOR_BINDING	35	0.55	2.01	0	0.002	0.405
GO_POSITIVE_REGULATION_OF_MITOTIC_CELL_CYCLE	122	0.42	2.01	0	0.002	0.41
GO_POSITIVE_REGULATION_OF_INTERLEUKIN_12_PRODUCTION	33	0.54	2.01	0	0.002	0.41
GO_POSITIVE_REGULATION_OF_LYMPHOCYTE_MEDIATED_IMMUNITY	68	0.46	2.01	0	0.002	0.417
GO_LEUKOCYTE_MEDIATED_IMMUNITY	153	0.41	2.01	0	0.002	0.421
GO_NUCLEAR_IMPORT	128	0.41	2.01	0	0.002	0.424
GO_NEGATIVE_REGULATION_OF_MULTI_ORGANISM_PROCESS	148	0.41	2.01	0	0.002	0.429
GO_CHROMATIN_REMODELING	146	0.41	2.01	0	0.002	0.437
GO_NUCLEOSOME_BINDING	44	0.51	2.01	0	0.002	0.442
GO_POSITIVE_REGULATION_OF_INTERLEUKIN_6_PRODUCTION	65	0.48	2	0	0.002	0.446
GO_MACROMOLECULAR_COMPLEX_DISASSEMBLY	180	0.4	2	0	0.002	0.446
GO_ACTIVATION_OF_ANAPHASE_PROMOTING_COMPLEX_ACTIVITY	15	0.69	2	0.002	0.002	0.448
GO_REGULATION_OF_UBIQUITIN_PROTEIN_LIGASE_ACTIVITY	18	0.64	2	0.002	0.002	0.451
GO_CELLULAR_DEFENSE_RESPONSE	59	0.48	2	0	0.002	0.465
GO_POSITIVE_REGULATION_OF_INFLAMMATORY_RESPONSE	113	0.43	2	0	0.002	0.47
GO_REGULATION_OF_T_HELPER_CELL_DIFFERENTIATION	26	0.58	2	0	0.002	0.47
GO_ISOMERASE_ACTIVITY	156	0.4	2	0	0.002	0.471
GO_KINESIN_COMPLEX	53	0.49	2	0	0.002	0.476
GO_SMALL_NUCLEAR_RIBONUCLEOPROTEIN_COMPLEX	59	0.47	1.99	0	0.002	0.476
GO_ACTIVATION_OF_CYSTEINE_TYPE_ENDOPEPTIDASE_ACTIVITY	94	0.44	1.99	0	0.002	0.489
GO_NEGATIVE_REGULATION_OF_T_CELL_PROLIFERATION	49	0.5	1.99	0	0.002	0.489
GO_LYMPHOCYTE_COSTIMULATION	72	0.46	1.99	0	0.002	0.489
GO_REGULATION_OF_ANTIGEN_PROCESSING_AND_PRESENTATION	23	0.6	1.99	0	0.002	0.497
GO_TOLL_LIKE_RECEPTOR_SIGNALING_PATHWAY	85	0.45	1.99	0	0.003	0.506
GO_DNA_REPLICATION_CHECKPOINT	15	0.66	1.99	0.002	0.003	0.516
GO_REGULATION_OF_TRANSLATION_IN_RESPONSE_TO_STRESS	19	0.63	1.98	0	0.003	0.521
GO_NEGATIVE_REGULATION_OF_VIRAL_GENOME_REPLICATION	49	0.49	1.98	0	0.003	0.527
GO_NEGATIVE_REGULATION_OF_LEUKOCYTE_PROLIFERATION	66	0.47	1.98	0	0.003	0.527
GO_RNA_SPLICING	335	0.36	1.98	0	0.003	0.531
GO_ENDOPEPTIDASE_ACTIVITY	408	0.36	1.98	0	0.003	0.537
GO_CYTOLYSIS	23	0.59	1.98	0	0.003	0.54
GO_POSITIVE_REGULATION_OF_T_HELPER_1_TYPE_IMMUNE_RESPONS	15	0.66	1.98	0	0.003	0.547
GO_REGULATION_OF_IMMUNE_EFFECTOR_PROCESS	416	0.36	1.98	0	0.003	0.547
GO_MONOSACCHARIDE_CATABOLIC_PROCESS	59	0.47	1.98	0	0.003	0.551
GO_CELL_CHEMOTAXIS	162	0.4	1.98	0	0.003	0.551
GO_NEGATIVE_REGULATION_OF_CELL_CYCLE_G1_S_PHASE_TRANSITIO	97	0.43	1.97	0	0.003	0.554
GO_ENDONUCLEASE_ACTIVITY	116	0.42	1.97	0	0.003	0.564

GO_ACUTE_PHASE_RESPONSE	43	0.5	1.97	0	0.003	0.567
GO_NATURAL_KILLER_CELL_MEDIATED_IMMUNITY	24	0.58	1.97	0	0.003	0.572
GO_REGULATION_OF_GENE_SILENCING	52	0.49	1.97	0	0.003	0.581
GO_RESPONSE_TO_XENOBIOTIC_STIMULUS	104	0.42	1.97	0	0.003	0.583
GO_STRUCTURE_SPECIFIC_DNA_BINDING	114	0.42	1.97	0	0.003	0.585
GO_NEGATIVE_REGULATION_OF_HOMOTYPIC_CELL_CELL_ADHESION	99	0.43	1.97	0	0.003	0.585
GO_REGULATION_OF_CELLULAR_PROTEIN_CATABOLIC_PROCESS	272	0.37	1.97	0	0.003	0.585
GO_REGULATION_OF_CYTOKINE_SECRETION	144	0.4	1.97	0	0.003	0.591
GO_PROTEIN_HETEROTETRAMERIZATION	37	0.53	1.97	0	0.003	0.596
GO_REGULATION_OF_CELL_ACTIVATION	454	0.35	1.96	0	0.003	0.604
GO_CHROMATIN	425	0.35	1.96	0	0.003	0.607
GO_DNA_DEPENDENT_DNA_REPLICATION_MAINTENANCE_OF_FIDELITY	22	0.57	1.96	0.002	0.003	0.608
GO_REGULATION_OF_LYMPHOCYTE_CHEMOTAXIS	19	0.61	1.96	0.002	0.003	0.608
GO_SIGNAL_TRANSDUCTION_BY_P53_CLASS_MEDIATOR	123	0.41	1.96	0	0.003	0.61
GO_NEGATIVE_REGULATION_OF_MITOTIC_NUCLEAR_DIVISION	34	0.52	1.96	0	0.003	0.616
GO_PEPTIDE_ANTIGEN_BINDING	27	0.57	1.96	0.005	0.003	0.616
GO_POSITIVE_REGULATION_OF_CANONICAL_WNT_SIGNALING_PATHWA	118	0.41	1.96	0	0.003	0.617
GO_NON_CANONICAL_WNT_SIGNALING_PATHWAY	140	0.4	1.96	0	0.003	0.621
GO_PSEUDOURIDINE_SYNTHESIS	17	0.63	1.96	0.011	0.003	0.621
GO_NUCLEUS_ORGANIZATION	134	0.4	1.96	0	0.003	0.621
GO_NAD_METABOLIC_PROCESS	54	0.48	1.96	0	0.003	0.625
GO_PLACENTA_DEVELOPMENT	138	0.4	1.96	0	0.003	0.625
GO_REGULATION_OF_TYPE_I_INTERFERON_MEDIATED_SIGNALING_PAT HWAY	39	0.51	1.95	0	0.003	0.649
GO_LYMPHOCYTE_APOPTOTIC_PROCESS	18	0.63	1.95	0	0.004	0.665
GO_REGULATION_OF_INTERLEUKIN_6_PRODUCTION	100	0.42	1.95	0	0.004	0.671
GO_DAMAGED_DNA_BINDING	62	0.46	1.95	0	0.004	0.675
GO_REGULATION_OF_EPIDERMAL_CELL_DIFFERENTIATION	44	0.5	1.95	0	0.004	0.68
GO_MICROTUBULE_CYTOSKELETON_ORGANIZATION	335	0.36	1.94	0	0.004	0.688
GO_BLASTOCYST_GROWTH	16	0.64	1.94	0.004	0.004	0.691
GO_POSITIVE_REGULATION_OF_EPIDERMAL_CELL_DIFFERENTIATION	20	0.6	1.94	0.005	0.004	0.696
GO_RNA_SPLICING_VIA_ENDONUCLEOLYTIC_CLEAVAGE_AND_LIGATION	16	0.64	1.94	0	0.004	0.701
GO_CYTOPLASMIC_PATTERN_RECOGNITION_RECEPTOR_SIGNALING_PA THWAY	33	0.53	1.94	0.002	0.004	0.708
GO_AMINO_ACID_ACTIVATION	51	0.48	1.94	0	0.004	0.708
GO_DISULFIDE_OXIDOREDUCTASE_ACTIVITY	30	0.54	1.94	0	0.004	0.711
GO_RIBONUCLEOPROTEIN_COMPLEX_BINDING	93	0.42	1.94	0	0.004	0.711
GO_POSITIVE_REGULATION_OF_NEUTROPHIL_MIGRATION	27	0.56	1.94	0.009	0.004	0.712
GO_POSITIVE_REGULATION_OF_LEUKOCYTE_PROLIFERATION	136	0.39	1.94	0	0.004	0.722
GO_GLUTAMINE_METABOLIC_PROCESS	23	0.57	1.93	0.002	0.004	0.73
GO_POSITIVE_REGULATION_OF_NF_KAPPAB_TRANSCRIPTION_FACTOR_ ACTIVITY	129	0.4	1.93	0	0.004	0.731
GO_REGULATION_OF_LEUKOCYTE_CHEMOTAXIS	95	0.43	1.93	0	0.004	0.74
GO_OXIDOREDUCTASE_ACTIVITY_ACTING_ON_CH_OH_GROUP_OF_DON	133	0.4	1.93	0	0.004	0.74
GO_RESPONSE_TO_INTERLEUKIN_1	114	0.41	1.93	0	0.004	0.744
GO_REGULATION_OF_MULTI_ORGANISM_PROCESS	462	0.34	1.92	0	0.004	0.759
GO_CELLULAR_PROTEIN_COMPLEX_LOCALIZATION	20	0.6	1.92	0	0.004	0.759
GO_PURINE_NTP_DEPENDENT_HELICASE_ACTIVITY	95	0.42	1.92	0	0.004	0.76
GO_RESPONSE_TO_X_RAY	30	0.54	1.92	0	0.005	0.764
GO_REGULATION_OF_ACTIVATED_T_CELL_PROLIFERATION	40	0.5	1.92	0.002	0.005	0.773
GO_REGULATION_OF_VIRAL_GENOME_REPLICATION	75	0.43	1.92	0	0.005	0.774
GO_CATALYTIC_STEP_2_SPLICEOSOME	88	0.43	1.92	0	0.005	0.781
GO_NEGATIVE_REGULATION_OF_EXTRINSIC_APOPTOTIC_SIGNALING_PA THWAY	98	0.41	1.92	0	0.005	0.792
GO_POSITIVE_REGULATION_OF_NUCLEAR_DIVISION	62	0.46	1.91	0	0.005	0.802
GO_POSITIVE_REGULATION_OF_B_CELL_MEDIATED_IMMUNITY	26	0.54	1.91	0.002	0.005	0.809
GO_REGULATION_OF_CENTROSOME_DUPLICATION	30	0.54	1.91	0	0.005	0.809
GO_REGULATION_OF_INTERLEUKIN_1_SECRETION	32	0.52	1.91	0.002	0.005	0.812
GO_NEGATIVE_REGULATION_OF_IMMUNE_EFFECTOR_PROCESS	102	0.41	1.91	0	0.005	0.813
GO_SINGLE_STRANDED_DNA_BINDING	84	0.43	1.91	0	0.005	0.815
GO_NUCLEOBASE_METABOLIC_PROCESS	39	0.51	1.91	0.002	0.005	0.818
GO_PROSTANOID_METABOLIC_PROCESS	27	0.53	1.91	0	0.005	0.823
GO_SEX_CHROMOSOME	27	0.56	1.91	0	0.005	0.824
GO_SOMATIC_DIVERSIFICATION_OF_IMMUNE_RECEPTORS	40	0.5	1.91	0	0.005	0.824
GO_SPLICEOSOMAL_TRI_SNRNP_COMPLEX	26	0.55	1.91	0	0.005	0.83
GO_RIBOSOME	220	0.37	1.91	0	0.005	0.83
GO_FLAVONOID_METABOLIC_PROCESS	27	0.55	1.9	0	0.005	0.833
GO_REGULATION_OF_B_CELL_MEDIATED_IMMUNITY	41	0.5	1.9	0	0.005	0.84
GO_FIBRINOLYSIS	21	0.58	1.9	0	0.005	0.843
GO_REGULATION_OF_CELLULAR_KETONE_METABOLIC_PROCESS	170	0.38	1.9	0	0.005	0.847
GO_MHC_CLASS_I_PROTEIN_BINDING	17	0.61	1.9	0.004	0.005	0.847
GO_DNA_POLYMERASE_ACTIVITY	34	0.52	1.9	0	0.006	0.849
GO_RRNA_MODIFICATION	22	0.57	1.9	0.002	0.006	0.851
GO_CENTRIOLE_ASSEMBLY	18	0.61	1.9	0.002	0.006	0.853
GO_SPLICEOSOMAL_SNRNP_ASSEMBLY	35	0.51	1.9	0	0.006	0.856
GO_REGULATION_OF_CELL_CYCLE_ARREST	107	0.4	1.89	0	0.006	0.863
GO_LEUKOCYTE_MIGRATION	259	0.36	1.89	0	0.006	0.866
GO_ORGAN_REGENERATION	82	0.43	1.89	0	0.006	0.867
GO_MYD88_INDEPENDENT_TOLL_LIKE_RECEPTOR_SIGNALING_PATHWA	30	0.52	1.89	0	0.006	0.869
GO_RNA_SPLICING_VIA_TRANSESTERIFICATION_REACTIONS	248	0.36	1.89	0	0.006	0.869
GO_NUCLEOBASE_CONTAINING_SMALL_MOLECULE_INTERCONVERSION	21	0.58	1.89	0	0.006	0.869
GO_POSITIVE_REGULATION_OF_I_KAPPAB_KINASE_NF_KAPPAB_SIGNALI	179	0.37	1.89	0	0.006	0.869
GO_REGULATION_OF_T_CELL_MEDIATED_IMMUNITY	52	0.46	1.89	0	0.006	0.871
GO_O_GLYCAN_PROCESSING	54	0.46	1.89	0	0.006	0.871
GO_DOUBLE_STRANDED_RNA_BINDING	62	0.45	1.89	0	0.006	0.874
GO_POSITIVE_REGULATION_OF_PROTEIN_CATABOLIC_PROCESS	262	0.36	1.88	0	0.006	0.88

GO_POSITIVE_REGULATION_OF_SUBSTRATE_ADHESION_DEPENDENT_CELL_SPREADING	28	0.53	1.88	0	0.006	0.887
GO_EXTRACELLULAR_MATRIX_DISASSEMBLY	74	0.44	1.88	0	0.006	0.887
GO_ATP_DEPENDENT_MICROTUBULE_MOTOR_ACTIVITY	18	0.6	1.88	0.002	0.006	0.892
GO_BASE_EXCISION_REPAIR	39	0.5	1.88	0	0.006	0.892
GO_REGULATION_OF_EXTRINSIC_APOPTOTIC_SIGNALING_PATHWAY_VIA_DEATH_DOMAIN_RECEPTORS	54	0.46	1.88	0	0.006	0.892
GO_STEROL_BIOSYNTHETIC_PROCESS	42	0.48	1.88	0	0.007	0.896
GO_POSITIVE_REGULATION_OF_CYTOKINE_SECRETION	96	0.4	1.88	0	0.007	0.897
GO_REGULATION_OF_INTERLEUKIN_10_PRODUCTION	44	0.47	1.88	0	0.007	0.897
GO_IMMUNOGLOBULIN_PRODUCTION	42	0.48	1.88	0	0.007	0.897
GO_REGULATION_OF_MITOCHONDRIAL_OUTER_MEMBRANE_PERMEABILIZATION_INVOLVED_IN_APOPTOTIC_SIGNALING_PATHWAY	43	0.48	1.87	0.002	0.007	0.904
GO_INTRAMOLECULAR_TRANSFERASE_ACTIVITY	27	0.53	1.87	0	0.007	0.906
GO_REGULATION_OF_INTERFERON_BETA_PRODUCTION	44	0.48	1.87	0	0.007	0.91
GO_CELL_CYCLE_G2_M_PHASE_TRANSITION	132	0.39	1.87	0	0.007	0.91
GO_REGULATION_OF_INFLAMMATORY_RESPONSE	290	0.35	1.87	0	0.007	0.91
GO_NF_KAPPAB_BINDING	30	0.52	1.87	0.002	0.007	0.91
GO_GLUCOSYLTRANSFERASE_ACTIVITY	15	0.64	1.87	0.002	0.007	0.911
GO_POSITIVE_REGULATION_OF_CELL_CYCLE_PHASE_TRANSITION	67	0.43	1.87	0	0.007	0.913
GO_RIBOSOME_ASSEMBLY	53	0.46	1.87	0	0.007	0.916
GO_RESPONSE_TO_UV	125	0.39	1.87	0	0.007	0.916
GO_NUCLEAR_CHROMATIN	282	0.35	1.87	0	0.007	0.916
GO_REGULATION_OF_INTERLEUKIN_8_PRODUCTION	61	0.45	1.87	0	0.007	0.916
GO_POSITIVE_REGULATION_OF_TRANSCRIPTION_INITIATION_FROM_RNA_POLYMERASE_II_PROMOTER	17	0.61	1.87	0.008	0.007	0.916
GO_REGULATION_OF_NIK_NF_KAPPAB_SIGNALING	42	0.49	1.87	0.002	0.007	0.916
GO_G2_DNA_DAMAGE_CHECKPOINT	31	0.52	1.87	0.002	0.007	0.919
GO_HISTONE_MRNA_METABOLIC_PROCESS	28	0.53	1.87	0	0.007	0.919
GO_MRNA_3_END_PROCESSING	60	0.44	1.86	0	0.007	0.92
GO_POSITIVE_REGULATION_OF_PROTEIN_LOCALIZATION_TO_NUCLEUS	128	0.38	1.86	0	0.007	0.921
GO_INTERMEDIATE_FILAMENT	183	0.37	1.86	0	0.007	0.927
GO_NEGATIVE_REGULATION_OF_NUCLEAR_DIVISION	46	0.47	1.86	0	0.007	0.927
GO_PIGMENT_GRANULE	102	0.4	1.86	0	0.007	0.927
GO_CELLULAR_GLUCURONIDATION	21	0.57	1.86	0	0.007	0.927
GO_HEXOSE_CATABOLIC_PROCESS	49	0.46	1.86	0	0.007	0.929
GO_CELLULAR_RESPONSE_TO_BIOTIC_STIMULUS	160	0.38	1.86	0	0.007	0.929
GO_T_CELL_MEDIATED_IMMUNITY	28	0.53	1.86	0	0.007	0.929
GO_POSITIVE_REGULATION_OF_MITOCHONDRIAL_OUTER_MEMBRANE_PERMEABILIZATION_INVOLVED_IN_APOPTOTIC_SIGNALING_PATHWAY	36	0.5	1.86	0.002	0.007	0.929
GO_REGULATION_OF_PROTEIN_INSERTION_INTO_MITOCHONDRIAL_MEMBRANE_INVOLVED_IN_APOPTOTIC_SIGNALING_PATHWAY	29	0.52	1.86	0.002	0.007	0.932
GO_REGULATION_OF_ALPHA_BETA_T_CELL_DIFFERENTIATION	46	0.47	1.86	0.002	0.008	0.936
GO_ORGANELLE_ENVELOPE_LUMEN	79	0.42	1.86	0	0.008	0.939
GO_NECROTIC_CELL_DEATH	28	0.52	1.86	0.004	0.008	0.939
GO_URONIC_ACID_METABOLIC_PROCESS	26	0.53	1.85	0.007	0.008	0.94
GO_POSITIVE_REGULATION_OF_CELL_DIVISION	131	0.38	1.85	0	0.008	0.943
GO_REGULATION_OF_EXIT_FROM_MITOSIS	16	0.6	1.85	0.002	0.008	0.943
GO_NUCLEOSOMAL_DNA_BINDING	30	0.52	1.85	0	0.008	0.947
GO_EXECUTION_PHASE_OF_APOPTOSIS	55	0.45	1.85	0	0.008	0.949
GO_LEUKOCYTE_CELL_CELL_ADHESION	255	0.35	1.85	0	0.008	0.95
GO_REGULATION_OF_GENE_EXPRESSION_EPIGENETIC	218	0.36	1.85	0	0.008	0.95
GO_CELLULAR_COMPONENT_DISASSEMBLY_INVOLVED_IN_EXECUTION_PHASE_OF_APOPTOSIS	43	0.47	1.85	0	0.008	0.95
GO_B_CELL_MEDIATED_IMMUNITY	66	0.43	1.85	0	0.008	0.955
GO_ISOPRENOID_BIOSYNTHETIC_PROCESS	25	0.54	1.85	0	0.008	0.955
GO_ESTABLISHMENT_OF_SPINDLE_ORIENTATION	26	0.53	1.85	0.009	0.008	0.955
GO_OXIDOREDUCTASE_ACTIVITY_ACTING_ON_THE_CH_OH_GROUP_OF_DONORS_NAD_OR_NADP_AS_ACCEPTOR	112	0.4	1.85	0	0.008	0.956
GO_REGULATION_OF_ORGAN_MORPHOGENESIS	241	0.35	1.84	0	0.008	0.958
GO_OXIDOREDUCTASE_ACTIVITY_ACTING_ON_A_SULFUR_GROUP_OF_DONORS	48	0.46	1.84	0	0.009	0.958
GO_POSITIVE_REGULATION_OF_ALPHA_BETA_T_CELL_DIFFERENTIATIO	37	0.49	1.84	0.002	0.009	0.959
GO_RESPONSE_TO_MURAMYL_DIPEPTIDE	15	0.63	1.84	0.002	0.009	0.959
GO_NUCLEAR_PORE_ORGANIZATION	15	0.61	1.84	0.007	0.009	0.962
GO_POSITIVE_REGULATION_OF_CELL_ADHESION	366	0.34	1.84	0	0.009	0.962
GO_REGULATION_OF_DNA_REPAIR	74	0.42	1.84	0	0.009	0.966
GO_POSITIVE_REGULATION_OF_CHEMOKINE_PRODUCTION	49	0.45	1.84	0	0.009	0.966
GO_PRECATALYTIC_SPLICEOSOME	22	0.56	1.83	0.004	0.009	0.969
GO_CYSTEINE_TYPE_ENDOPEPTIDASE_ACTIVITY	86	0.41	1.83	0	0.009	0.972
GO_STEROID_DEHYDROGENASE_ACTIVITY	27	0.53	1.83	0.002	0.009	0.972
GO_NUCLEOTIDE_BINDING_DOMAIN_LEUCINE_RICH_REPEAT_CONTAINING_RECEPTOR_SIGNALING_PATHWAY	28	0.52	1.83	0.002	0.009	0.972
GO_AMIDE_BIOSYNTHETIC_PROCESS	488	0.32	1.83	0	0.01	0.972
GO_MISMATCH_REPAIR	28	0.5	1.83	0.005	0.01	0.972
GO_CELLULAR_MODIFIED_AMINO_ACID_BIOSYNTHETIC_PROCESS	51	0.46	1.83	0	0.01	0.972
GO_POSITIVE_REGULATION_OF_VIRAL_PROCESS	91	0.4	1.83	0	0.01	0.974
GO_LUMENAL_SIDE_OF_MEMBRANE	31	0.5	1.83	0.005	0.01	0.974
GO_NEGATIVE_REGULATION_OF_CYTOSKELETON_ORGANIZATION	219	0.35	1.83	0	0.01	0.974
GO_NEGATIVE_REGULATION_OF_LYMPHOCYTE_MEDIATED_IMMUNITY	36	0.49	1.83	0.002	0.01	0.974
GO_PROTEIN_DISULFIDE_OXIDOREDUCTASE_ACTIVITY	23	0.54	1.83	0	0.01	0.975
GO_DENDRITIC_CELL_CHEMOTAXIS	16	0.6	1.82	0.011	0.01	0.978
GO_ARACHIDONIC_ACID_MONOOXYGENASE_ACTIVITY	15	0.63	1.82	0.007	0.01	0.978
GO_POSITIVE_REGULATION_OF_ACTIN_FILAMENT_POLYMERIZATION	67	0.43	1.82	0	0.01	0.978
GO_TRNA_MODIFICATION	56	0.44	1.82	0	0.01	0.98
GO_CYSTEINE_TYPE_ENDOPEPTIDASE_INHIBITOR_ACTIVITY	55	0.44	1.82	0	0.01	0.98
GO_SOMATIC_DIVERSIFICATION_OF_IMMUNOGLOBULINS	27	0.54	1.82	0.002	0.01	0.983

GO_ERROR_PRONE_TRANSLESION_SYNTHESIS	19	0.58	1.82	0.002	0.01	0.983
GO_REGULATION_OF_PROTEIN_CATABOLIC_PROCESS	391	0.33	1.81	0	0.01	0.983
GO_CENTROSOME_DUPLICATION	31	0.5	1.81	0	0.011	0.983
GO_REGULATION_OF_VIRAL_ENTRY_INTO_HOST_CELL	28	0.51	1.81	0.002	0.011	0.984
GO_REGULATION_OF_CELL_KILLING	62	0.43	1.81	0	0.011	0.984
GO_REGULATION_OF_PRODUCTION_OF_MOLECULAR_MEDIATOR_OF_IMMUNE_RESPONSE	99	0.39	1.81	0	0.011	0.986
GO_REGULATION_OF_GRANULOCYTE_CHEMOTAXIS	39	0.48	1.81	0	0.011	0.986
GO_REGULATION_OF_T_CELL_RECEPTOR_SIGNALING_PATHWAY	28	0.52	1.81	0	0.011	0.987
GO_LIGASE_ACTIVITY_FORMING_CARBON_OXYGEN_BONDS	44	0.47	1.81	0	0.011	0.987
GO_NUCLEAR_TRANSPORT	337	0.33	1.81	0	0.011	0.987
GO_MATERNAL_PROCESS_INVOLVED_IN_FEMALE_PREGNANCY	60	0.43	1.81	0	0.011	0.989
GO_MYELOID_DENDRITIC_CELL_ACTIVATION	26	0.52	1.81	0.004	0.011	0.989
GO_RNA_MODIFICATION	110	0.39	1.8	0	0.011	0.991
GO_MYELOID_LEUKOCYTE_MIGRATION	99	0.4	1.8	0	0.011	0.991
GO_RESPONSE_TO_VITAMIN	98	0.39	1.8	0	0.012	0.992
GO_REGULATION_OF_LYMPHOCYTE_DIFFERENTIATION	131	0.38	1.8	0	0.012	0.992
GO_ACTIVATION_OF_NF_KAPPAB_INDUCING_KINASE_ACTIVITY	17	0.58	1.8	0.005	0.012	0.992
GO_REGULATION_OF_MEMBRANE_PERMEABILITY	70	0.41	1.8	0.003	0.012	0.992
GO_POSITIVE_REGULATION_OF_T_HELPER_CELL_DIFFERENTIATION	18	0.58	1.8	0.002	0.012	0.992
GO_POSITIVE_REGULATION_OF_TELOMERE_MAINTENANCE_VIA_TELOMERE_LENGTHENING	33	0.5	1.8	0.002	0.012	0.992
GO_LEUKOCYTE_MEDIATED_CYTOTOXICITY	33	0.49	1.8	0	0.012	0.992
GO_SNRNA_METABOLIC_PROCESS	82	0.41	1.8	0	0.012	0.992
GO_NECROPTOTIC_PROCESS	21	0.55	1.8	0.009	0.012	0.992
GO_MULTICELLULAR_ORGANISMAL_MACROMOLECULE_METABOLIC_PROCESS	77	0.41	1.8	0.005	0.012	0.993
GO_REGULATION_OF_IMMUNOGLOBULIN_PRODUCTION	46	0.45	1.8	0	0.012	0.994
GO_REGULATION_OF_DNA_BIOSYNTHETIC_PROCESS	90	0.39	1.8	0	0.012	0.994
GO_INTESTINAL_EPITHELIAL_CELL_DIFFERENTIATION	17	0.58	1.8	0.004	0.012	0.994
GO_REGULATION_OF_TRANSCRIPTION_FROM_RNA_POLYMERASE_I_PROMOTER	23	0.54	1.8	0	0.012	0.995
GO_SOMATIC_RECOMBINATION_OF_IMMUNOGLOBULIN_GENE_SEGMENT	21	0.55	1.79	0.011	0.012	0.995
GO_POSITIVE_REGULATION_OF_LYMPHOCYTE_DIFFERENTIATION	79	0.41	1.79	0.003	0.012	0.995
GO_DRUG_METABOLIC_PROCESS	39	0.46	1.79	0	0.012	0.995
GO_ACUTE_INFLAMMATORY_RESPONSE	73	0.42	1.79	0	0.012	0.996
GO_SOMATIC_CELL_DNA_RECOMBINATION	33	0.49	1.79	0	0.012	0.996
GO_REGULATION_OF_INTERLEUKIN_13_PRODUCTION	18	0.56	1.79	0	0.012	0.996
GO_REGULATION_OF_LEUKOCYTE_MEDIATED_CYTOTOXICITY	52	0.44	1.79	0	0.012	0.996
GO_RESPONSE_TO_PROTOZOAN	20	0.56	1.79	0.002	0.012	0.996
GO_REGULATION_OF_RESPONSE_TO_DNA_DAMAGE_STIMULUS	143	0.37	1.79	0	0.012	0.996
GO_NUCLEAR_PERIPHERY	117	0.38	1.79	0	0.013	0.997
GO_NCRNA_3_END_PROCESSING	21	0.55	1.79	0.002	0.013	0.997
GO_POSITIVE_REGULATION_OF_T_CELL_MEDIATED_IMMUNITY	32	0.5	1.79	0.002	0.013	0.997
GO_CELL_KILLING	52	0.44	1.79	0	0.013	0.997
GO_PROTEASOMAL_PROTEIN_CATABOLIC_PROCESS	269	0.34	1.78	0	0.013	0.997
GO_NEGATIVE_REGULATION_OF_CANONICAL_WNT_SIGNALING_PATHW	162	0.36	1.78	0	0.013	0.997
GO_ORGAN_OR_TISSUE_SPECIFIC_IMMUNE_RESPONSE	29	0.5	1.78	0	0.013	0.997
GO_NEGATIVE_REGULATION_OF_CELL_CELL_ADHESION	135	0.37	1.78	0	0.013	0.998
GO_DENDRITIC_CELL_MIGRATION	21	0.55	1.78	0.008	0.013	0.998
GO_REGULATION_OF_EXTRINSIC_APOPTOTIC_SIGNALING_PATHWAY	151	0.36	1.78	0	0.013	0.998
GO_ESTABLISHMENT_OF_MITOTIC_SPINDLE_ORIENTATION	20	0.56	1.78	0.002	0.013	0.998
GO_MRNA_PROCESSING	397	0.32	1.78	0	0.013	0.998
GO_MYELOID_LEUKOCYTE_DIFFERENTIATION	96	0.39	1.78	0	0.013	0.998
GO_REGULATION_OF_DOUBLE_STRAND_BREAK_REPAIR	38	0.48	1.78	0.002	0.013	0.998
GO_LYMPH_NODE_DEVELOPMENT	17	0.59	1.78	0.009	0.014	0.998
GO_NEGATIVE_REGULATION_OF_ORGANELLE_ORGANIZATION	384	0.32	1.78	0	0.014	0.998
GO_RIBOSOMAL_SUBUNIT	159	0.36	1.78	0	0.014	0.998
GO_DEVELOPMENTAL_PROGRAMMED_CELL_DEATH	26	0.51	1.78	0.005	0.014	0.998
GO_NUCLEAR_TRANSCRIBED_MRNA_CATABOLIC_PROCESS_DEADENYLATION_DEPENDENT_DECAY	57	0.43	1.78	0.002	0.014	0.998
GO_RAN_GTPASE_BINDING	31	0.5	1.78	0.004	0.014	0.998
GO_NEGATIVE_REGULATION_OF_PROTEOLYSIS	322	0.33	1.77	0	0.014	0.998
GO_PROTEIN_HYDROXYLATION	18	0.56	1.77	0.01	0.014	0.998
GO_NEGATIVE_REGULATION_OF_INTERFERON_GAMMA_PRODUCTION	32	0.49	1.77	0.009	0.014	0.998
GO_REGULATION_OF_TELOMERE_MAINTENANCE_VIA_TELOMERE_LENGTHENING	48	0.44	1.77	0.005	0.014	0.998
GO_PRODUCTION_OF_MOLECULAR_MEDIATOR_OF_IMMUNE_RESPONSE	59	0.42	1.77	0.002	0.014	0.998
GO_POSITIVE_REGULATION_OF_DNA_RECOMBINATION	18	0.57	1.77	0.006	0.014	0.998
GO_REGULATION_OF_RESPONSE_TO_CYTOKINE_STIMULUS	142	0.36	1.77	0	0.015	0.999
GO_RNA_STABILIZATION	31	0.49	1.77	0.007	0.015	0.999
GO_GLUCOSE_CATABOLIC_PROCESS	29	0.49	1.77	0.009	0.015	0.999
GO_CELLULAR_RESPONSE_TO_HEAT	36	0.47	1.77	0.002	0.015	0.999
GO_LEUKOCYTE_ACTIVATION	409	0.31	1.76	0	0.015	0.999
GO_REGULATION_OF_LYMPHOCYTE_APOPTOTIC_PROCESS	53	0.43	1.76	0.005	0.016	0.999
GO_DNA_SECONDARY_STRUCTURE_BINDING	22	0.52	1.76	0	0.016	0.999
GO_POSITIVE_REGULATION_OF_TISSUE_REMODELING	26	0.51	1.76	0.002	0.016	0.999
GO_FOLIC_ACID_CONTAINING_COMPOUND_METABOLIC_PROCESS	28	0.5	1.76	0.004	0.016	0.999
GO_ALCOHOL_DEHYDROGENASE_NADP_ACTIVITY	16	0.59	1.76	0.004	0.016	0.999
GO_MITOCHONDRIAL_MEMBRANE_ORGANIZATION	92	0.39	1.76	0	0.016	0.999
GO_NCRNA_TRANSCRIPTION	88	0.39	1.76	0	0.016	0.999
GO_RESPONSE_TO_GONADOTROPIN	28	0.5	1.75	0.005	0.016	0.999
GO_LYMPHOCYTE_ACTIVATION	338	0.32	1.75	0	0.016	0.999
GO_RIBONUCLEOPROTEIN_COMPLEX_SUBUNIT_ORGANIZATION	186	0.35	1.75	0	0.016	0.999
GO_MATERNAL_PLACENTA_DEVELOPMENT	31	0.49	1.75	0	0.016	0.999
GO_INTRAMOLECULAR_OXIDOREDUCTASE_ACTIVITY	51	0.43	1.75	0.005	0.017	0.999
GO_INTERCELLULAR_BRIDGE	43	0.45	1.75	0.004	0.017	0.999

GO_PERICENTRIC_HETEROCHROMATIN	15	0.58	1.75	0.007	0.017	0.999
GO_COLLAGEN_FIBRIL_ORGANIZATION	37	0.47	1.75	0.009	0.017	0.999
GO_REGULATION_OF_CYTOSKELETON_ORGANIZATION	490	0.31	1.75	0	0.017	0.999
GO_INTERLEUKIN_1_PRODUCTION	15	0.58	1.74	0.019	0.018	0.999
GO_PROSTANOID_BIOSYNTHETIC_PROCESS	19	0.55	1.74	0.007	0.018	0.999
GO_REGULATION_OF_I_KAPPAB_KINASE_NF_KAPPAB_SIGNALING	233	0.33	1.74	0	0.018	0.999
GO_NEGATIVE_REGULATION_OF_PEPTIDASE_ACTIVITY	239	0.33	1.74	0	0.018	0.999
GO_EXOSOME_RNASE_COMPLEX_	21	0.54	1.74	0.009	0.018	0.999
GO_REGULATION_OF_HUMORAL_IMMUNE_RESPONSE	49	0.43	1.74	0.007	0.018	0.999
GO_DNA_DIRECTED_DNA_POLYMERASE_ACTIVITY	27	0.5	1.74	0.005	0.018	0.999
GO_OXIDOREDUCTASE_ACTIVITY_ACTING_ON_SINGLE_DONORS_WITH_INCORPORATION_OF_MOLECULAR_OXYGEN	27	0.49	1.74	0.005	0.018	0.999
GO_NEGATIVE_REGULATION_OF_TRANSFERASE_ACTIVITY	346	0.32	1.74	0	0.018	0.999
GO_ANAPHASE_PROMOTING_COMPLEX	22	0.53	1.74	0.004	0.018	0.999
GO_POSITIVE_REGULATION_OF_INTERLEUKIN_8_PRODUCTION	45	0.44	1.74	0.007	0.018	0.999
GO_NUCLEOSIDE_MONOPHOSPHATE_BIOSYNTHETIC_PROCESS	82	0.39	1.74	0	0.019	0.999
GO_COENZYME_METABOLIC_PROCESS	261	0.33	1.73	0	0.019	0.999
GO_CELL_DIVISION_SITE	48	0.43	1.73	0.002	0.019	0.999
GO_REGULATION_OF_NATURAL_KILLER_CELL_MEDIATED_IMMUNITY	34	0.47	1.73	0.007	0.019	0.999
GO_EMBRYONIC_PLACENTA_DEVELOPMENT	83	0.39	1.73	0.003	0.019	1
GO_POSITIVE_REGULATION_OF_TELOMERE_MAINTENANCE	44	0.45	1.73	0.002	0.019	1
GO_DECIDUALIZATION	21	0.53	1.73	0.007	0.019	1
GO_POSITIVE_REGULATION_OF_WNT_SIGNALING_PATHWAY	151	0.35	1.73	0	0.019	1
GO_POSITIVE_REGULATION_OF_NIK_NF_KAPPAB_SIGNALING	29	0.5	1.73	0.002	0.019	1
GO_PURINE_NUCLEOBASE_METABOLIC_PROCESS	21	0.53	1.73	0.015	0.019	1
GO_ELECTRON_CARRIER_ACTIVITY	110	0.37	1.73	0	0.019	1
GO_RESPONSE_TO_REACTIVE_OXYGEN_SPECIES	191	0.34	1.73	0	0.02	1
GO_REGULATION_OF_CHROMOSOME_ORGANIZATION	271	0.33	1.73	0	0.02	1
GO_POSITIVE_REGULATION_OF_INTERLEUKIN_10_PRODUCTION	29	0.49	1.72	0.009	0.02	1
GO_DEFENSE_RESPONSE_TO_GRAM_POSITIVE_BACTERIUM	67	0.4	1.72	0	0.02	1
GO_MITOTIC_G2_M_TRANSITION_CHECKPOINT	19	0.55	1.72	0.008	0.02	1
GO_NUCLEOSIDE_PHOSPHATE_BIOSYNTHETIC_PROCESS	182	0.34	1.72	0	0.02	1
GO_DSRNA_FRAGMENTATION	21	0.52	1.72	0.013	0.02	1
GO_REGULATION_OF_DEFENSE_RESPONSE_TO_VIRUS	186	0.34	1.72	0	0.02	1
GO_PYRIDINE_NUCLEOTIDE_BIOSYNTHETIC_PROCESS	17	0.56	1.72	0.023	0.02	1
GO_REGULATION_OF_ANTIGEN_RECEPTOR_MEDIATED_SIGNALING_PATHWAY	41	0.44	1.72	0.002	0.02	1
GO_REGULATION_OF_TYROSINE_PHOSPHORYLATION_OF_STAT1_PROT	16	0.58	1.72	0.013	0.02	1
GO_REGULATION_OF_KERATINOCYTE_PROLIFERATION	27	0.49	1.72	0.007	0.021	1
GO_REGULATION_OF_NECROTIC_CELL_DEATH	26	0.5	1.72	0.011	0.02	1
GO_INTESTINAL_ABSORPTION	26	0.49	1.72	0.002	0.021	1
GO_REGULATION_OF_TELOMERASE_ACTIVITY	40	0.44	1.72	0.005	0.021	1
GO_NEGATIVE_REGULATION_OF_CELL_CYCLE	426	0.31	1.72	0	0.021	1
GO_NEGATIVE_REGULATION_OF_ALPHA_BETA_T_CELL_ACTIVATION	23	0.52	1.72	0.013	0.021	1
GO_NEGATIVE_REGULATION_OF_IMMUNE_SYSTEM_PROCESS	362	0.31	1.72	0	0.021	1
GO_REGULATION_OF_B_CELL_ACTIVATION	100	0.38	1.72	0	0.021	1
GO_REGULATION_OF_APOPTOTIC_SIGNALING_PATHWAY	359	0.31	1.71	0	0.021	1
GO_RNA_CATABOLIC_PROCESS	223	0.33	1.71	0	0.021	1
GO_POSITIVE_REGULATION_OF_APOPTOTIC_SIGNALING_PATHWAY	168	0.34	1.71	0	0.021	1
GO_NEGATIVE_REGULATION_OF_CYTOKINE_PRODUCTION	205	0.33	1.71	0	0.021	1
GO_POSITIVE_REGULATION_OF_STAT_CASCADE	73	0.39	1.71	0.002	0.022	1
GO_PEPTIDASE_REGULATOR_ACTIVITY	208	0.33	1.71	0	0.022	1
GO_MULTI_ORGANISM_METABOLIC_PROCESS	138	0.35	1.71	0	0.022	1
GO_REGULATION_OF_RESPONSE_TO_WOUNDING	407	0.31	1.71	0	0.022	1
GO_NUCLEOCYTOPLASMIC_TRANSPORTER_ACTIVITY	24	0.5	1.7	0.016	0.023	1
GO_POSITIVE_REGULATION_OF_TRANSFORMING_GROWTH_FACTOR_BETA_PRODUCTION	16	0.57	1.7	0.011	0.023	1
GO_U2_SNRNP	19	0.53	1.7	0.009	0.023	1
GO_POSITIVE_REGULATION_OF_LAMELLIPODIUM_ORGANIZATION	23	0.51	1.7	0.011	0.023	1
GO_REGULATION_OF_THYMOCYTE_AGGREGATION	26	0.5	1.7	0.004	0.024	1
GO_BRANCHING_INVOLVED_IN_MAMMARY_GLAND_DUCT_MORPHOGENE	20	0.52	1.7	0.011	0.024	1
GO_NEGATIVE_REGULATION_OF_DEFENSE_RESPONSE	141	0.35	1.7	0	0.024	1
GO_CELLULAR_MODIFIED_AMINO_ACID_METABOLIC_PROCESS	213	0.32	1.7	0	0.024	1
GO_FATTY_ACID_DERIVATIVE_METABOLIC_PROCESS	92	0.37	1.69	0.005	0.024	1
GO_CENTROSOME_LOCALIZATION	18	0.55	1.69	0.018	0.024	1
GO_NEGATIVE_REGULATION_OF_T_CELL_DIFFERENTIATION	32	0.46	1.69	0.007	0.024	1
GO_REGULATION_OF_CELL_CYCLE_G2_M_PHASE_TRANSITION	57	0.41	1.69	0.002	0.024	1
GO_CHROMATIN_SILENCING_AT_RDNA	35	0.45	1.69	0.01	0.024	1
GO_RESPONSE_TO_HEAT	89	0.37	1.69	0.005	0.025	1
GO_GERM_CELL_NUCLEUS	20	0.53	1.69	0.016	0.025	1
GO_POSITIVE_REGULATION_OF_TUMOR_NECROSIS_FACTOR_SUPERFAMILY_CYTOKINE_PRODUCTION	57	0.41	1.69	0.002	0.025	1
GO_HETEROCHROMATIN	66	0.4	1.69	0.002	0.025	1
GO_REGULATION_OF_TUMOR_NECROSIS_FACTOR_SUPERFAMILY_CYTOKINE_PRODUCTION	101	0.36	1.69	0	0.025	1
GO_NUCLEAR_TRANSCRIBED_MRNA_CATABOLIC_PROCESS_EXONUCLEOLYTIC	31	0.46	1.69	0.01	0.025	1
GO_MAMMARY_GLAND_EPITHELIUM_DEVELOPMENT	53	0.41	1.69	0.002	0.025	1
GO_REGULATION_OF_CELLULAR_RESPONSE_TO_HEAT	76	0.39	1.69	0	0.025	1
GO_REGULATION_OF_ISOTYPE_SWITCHING	24	0.5	1.69	0.009	0.025	1
GO_ENDODERMAL_CELL_DIFFERENTIATION	40	0.43	1.69	0.005	0.025	1
GO_REGULATION_OF_HAIR_FOLLICLE_DEVELOPMENT	15	0.58	1.69	0.02	0.025	1
GO_REGULATION_OF_TELOMERE_MAINTENANCE	62	0.4	1.69	0.002	0.025	1
GO_SITE_OF_DOUBLE_STRAND_BREAK	31	0.46	1.69	0.014	0.025	1
GO_NEGATIVE_REGULATION_OF_PROTEIN_MATURATION	35	0.46	1.69	0.005	0.025	1
GO_POSITIVE_REGULATION_OF_FIBROBLAST_PROLIFERATION	52	0.42	1.69	0.007	0.025	1
GO_NEGATIVE_REGULATION_OF_GENE_EXPRESSION_EPIGENETIC	103	0.36	1.69	0	0.025	1

GO_MICROTUBULE_MOTOR_ACTIVITY	75	0.39	1.68	0.005	0.026	1
GO_METALLOENDOPEPTIDASE_ACTIVITY	111	0.36	1.68	0	0.026	1
GO_POSITIVE_REGULATION_OF_PROTEIN_COMPLEX_ASSEMBLY	192	0.33	1.68	0	0.026	1
GO_MRNA_CLEAVAGE	21	0.52	1.68	0.011	0.026	1
GO_POSITIVE_REGULATION_OF_T_CELL_MEDIATED_CYTOTOXICITY	16	0.56	1.68	0.004	0.026	1
GO_PYRIDINE_CONTAINING_COMPOUND_BIOSYNTHETIC_PROCESS	21	0.52	1.68	0.016	0.026	1
GO_POSITIVE_REGULATION_OF_ANTIGEN_PROCESSING_AND_PRESENTATION	16	0.56	1.68	0.017	0.026	1
GO_MYELOID_CELL_DIFFERENTIATION	187	0.33	1.68	0	0.026	1
GO_POSITIVE_REGULATION_OF_RESPONSE_TO_WOUNDING	162	0.34	1.68	0	0.027	1
GO_DNA_LIGATION	16	0.57	1.68	0.016	0.027	1
GO_RESPIRATORY_BURST	15	0.57	1.68	0.009	0.027	1
GO_NADH_METABOLIC_PROCESS	35	0.45	1.68	0.009	0.027	1
GO_EPIBOLY	23	0.51	1.67	0.02	0.027	1
GO_DEATH_RECEPTOR_ACTIVITY	24	0.49	1.67	0.013	0.027	1
GO_CHROMATIN_DNA_BINDING	80	0.38	1.67	0	0.027	1
GO_CELL_SEPARATION_AFTER_CYTOKINESIS	16	0.56	1.67	0.01	0.027	1
GO_POSITIVE_REGULATION_OF_LEUKOCYTE_DIFFERENTIATION	129	0.35	1.67	0	0.027	1
GO_OOCYTE_MATURATION	18	0.54	1.67	0.007	0.027	1
GO_SIGNALING_PATTERN_RECOGNITION_RECEPTOR_ACTIVITY	17	0.55	1.67	0.011	0.028	1
GO_NEGATIVE_REGULATION_OF_EPIDERMIS_DEVELOPMENT	15	0.57	1.67	0.018	0.028	1
GO_NEGATIVE_REGULATION_OF_WNT_SIGNALING_PATHWAY	196	0.32	1.67	0	0.028	1
GO_ENDONUCLEASE_ACTIVITY_ACTIVE_WITH_EITHER_RIBO_OR_DEOXYRIBONUCLEIC_ACIDS_AND_PRODUCING_3_PHOSPHOMONOESTERS	17	0.54	1.67	0.014	0.028	1
GO_NEGATIVE_REGULATION_OF_LEUKOCYTE_MEDIATED_IMMUNITY	47	0.42	1.67	0.007	0.028	1
GO_CELLULAR_RESPONSE_TO_IONIZING_RADIATION	51	0.42	1.67	0.002	0.028	1
GO_POSITIVE_REGULATION_OF_INTERLEUKIN_6_SECRETION	16	0.56	1.67	0.007	0.028	1
GO_PROTEIN_EXPORT_FROM_NUCLEUS	30	0.46	1.67	0.01	0.028	1
GO_ATP_GENERATION_FROM_ADP	38	0.45	1.67	0.007	0.028	1
GO_PROTEIN_MATURATION	262	0.31	1.67	0	0.028	1
GO_REGULATION_OF_NEUTROPHIL_MIGRATION	32	0.45	1.67	0.01	0.028	1
GO_POSITIVE_REGULATION_OF_PROTEIN_OLIGOMERIZATION	21	0.52	1.67	0.002	0.028	1
GO_ACTIN_NUCLEATION	22	0.51	1.67	0.009	0.028	1
GO_LARGE_RIBOSOMAL_SUBUNIT	93	0.37	1.67	0	0.028	1
GO_TRNA_BINDING	42	0.43	1.67	0.009	0.028	1
GO_POSITIVE_REGULATION_OF_LYMPHOCYTE_APOPTOTIC_PROCESS	19	0.53	1.67	0.022	0.028	1
GO_ISOTYPE_SWITCHING	16	0.56	1.67	0.016	0.029	1
GO_COFACTOR_METABOLIC_PROCESS	329	0.31	1.67	0	0.029	1
GO_CYTOPLASMIC_MRNA_PROCESSING_BODY	70	0.38	1.67	0.01	0.029	1
GO_REGULATION_OF_T_CELL_APOPTOTIC_PROCESS	32	0.46	1.66	0.01	0.029	1
GO_INTERMEDIATE_FILAMENT_CYTOSKELETON	226	0.32	1.66	0	0.029	1
GO_ARP2_3_COMPLEX_MEDIATED_ACTIN_NUCLEATION	16	0.56	1.66	0.009	0.029	1
GO_MULTICELLULAR_ORGANISM_METABOLIC_PROCESS	91	0.38	1.66	0	0.029	1
GO_POSITIVE_REGULATION_OF_TYROSINE_PHOSPHORYLATION_OF_STAT3_PROTEIN	37	0.45	1.66	0.005	0.029	1
GO_OSTEOCLAST_DIFFERENTIATION	30	0.47	1.66	0.005	0.029	1
GO_METHYLTRANSFERASE_COMPLEX	89	0.36	1.66	0	0.029	1
GO_NUCLEOTIDE_SUGAR_METABOLIC_PROCESS	33	0.45	1.66	0.011	0.03	1
GO_REGULATION_OF_TOLL_LIKE_RECEPTOR_SIGNALING_PATHWAY	45	0.41	1.66	0.002	0.03	1
GO_DNA_BINDING_BENDING	20	0.52	1.66	0.011	0.03	1
GO_ATPASE_ACTIVITY	417	0.3	1.66	0	0.03	1
GO_REGULATION_OF_TYROSINE_PHOSPHORYLATION_OF_STAT_PROTEI	68	0.39	1.66	0.008	0.03	1
GO_STRUCTURAL_CONSTITUENT_OF_RIBOSOME	206	0.32	1.65	0	0.03	1
GO_INTRAMOLECULAR_OXIDOREDUCTASE_ACTIVITY_TRANSPOSING_S_S_BONDS	22	0.5	1.65	0.014	0.031	1
GO_MYELOID_DENDRITIC_CELL_DIFFERENTIATION	20	0.51	1.65	0.009	0.031	1
GO_ANTIGEN_PROCESSING_AND_PRESENTATION_OF_ENDOGENOUS_ANTIGEN	17	0.54	1.65	0.021	0.031	1
GO_NEGATIVE_REGULATION_OF_ADAPTIVE_IMMUNE_RESPONSE	37	0.44	1.65	0.007	0.031	1
GO_RIBONUCLEOPROTEIN_GRANULE	146	0.34	1.65	0	0.031	1
GO_TRANSLATION_INITIATION_FACTOR_BINDING	28	0.47	1.65	0.01	0.031	1
GO_CYTOKINE_SECRETION	37	0.44	1.65	0.009	0.031	1
GO_SNRNA_BINDING	33	0.44	1.65	0.016	0.032	1
GO_NUCLEAR_MATRIX	94	0.36	1.65	0.002	0.032	1
GO_POSITIVE_REGULATION_OF_NF_KAPPAB_IMPORT_INTO_NUCLEUS	27	0.47	1.65	0.005	0.032	1
GO_PYRIMIDINE_NUCLEOBASE_METABOLIC_PROCESS	19	0.52	1.65	0.025	0.032	1
GO_REGULATION_OF_INTERLEUKIN_2_BIOSYNTHETIC_PROCESS	18	0.52	1.65	0.022	0.032	1
GO_EPITHELIAL_CELL_DIFFERENTIATION	487	0.29	1.65	0	0.032	1
GO_REGULATION_OF_RESPONSE_TO_BIOTIC_STIMULUS	230	0.32	1.65	0	0.032	1
GO_ANATOMICAL_STRUCTURE_HOMEOSTASIS	272	0.31	1.65	0	0.032	1
GO_XY_BODY	15	0.55	1.65	0.019	0.032	1
GO_MONOCARBOXYLIC_ACID_BINDING	63	0.39	1.65	0	0.032	1
GO_PROTEIN_HETEROOLIGOMERIZATION	108	0.35	1.64	0	0.032	1
GO_NEGATIVE_REGULATION_OF_CELLULAR_PROTEIN_CATABOLIC_PRO	63	0.39	1.64	0.007	0.032	1
GO_ALPHA_BETA_T_CELL_ACTIVATION	54	0.41	1.64	0.007	0.032	1
GO_CELLULAR_RESPONSE_TO_INTERLEUKIN_4	26	0.49	1.64	0.007	0.033	1
GO_POSITIVE_REGULATION_OF_MULTI_ORGANISM_PROCESS	154	0.33	1.64	0.003	0.033	1
GO_REGULATION_OF_T_CELL_CYTOKINE_PRODUCTION	22	0.5	1.64	0.007	0.033	1
GO_REGULATION_OF_DEFENSE_RESPONSE_TO_VIRUS_BY_HOST	131	0.34	1.64	0	0.033	1
GO_NUCLEAR_UBIQUITIN_LIGASE_COMPLEX	42	0.43	1.64	0.009	0.033	1
GO_MONOSACCHARIDE_METABOLIC_PROCESS	198	0.32	1.64	0	0.033	1
GO_GLUTAMINE_FAMILY_AMINO_ACID_BIOSYNTHETIC_PROCESS	19	0.51	1.64	0.015	0.033	1
GO_PEPTIDASE_INHIBITOR_ACTIVITY	171	0.33	1.64	0	0.033	1
GO_RESPONSE_TO_INTERFERON_ALPHA	20	0.51	1.64	0.016	0.033	1
GO_REGULATION_OF_TRANSLATIONAL_FIDELITY	15	0.54	1.64	0.014	0.033	1
GO_OXIDOREDUCTASE_ACTIVITY_ACTING_ON_THE_CH_CH_GROUP_OF_DONORS_NAD_OR_NADP_AS_ACCEPTOR	24	0.49	1.64	0.017	0.033	1

GO_DNA_TEMPLATED_TRANSCRIPTION_ELONGATION	95	0.36	1.64	0	0.033	1
GO_REPRODUCTIVE_SYSTEM_DEVELOPMENT	406	0.29	1.64	0	0.033	1
GO_HOMOTYPIC_CELL_CELL_ADHESION	51	0.41	1.64	0.005	0.033	1
GO_NEGATIVE_REGULATION_OF_DEFENSE_RESPONSE_TO_VIRUS	18	0.52	1.64	0.019	0.034	1
GO_REGULATION_OF_VIRAL_TRANSCRIPTION	61	0.39	1.64	0.002	0.034	1
GO_TETRAHYDROFOLATE_METABOLIC_PROCESS	20	0.51	1.63	0.018	0.034	1
GO_CELLULAR_RESPONSE_TO_RADIATION	134	0.33	1.63	0	0.034	1
GO_POSITIVE_REGULATION_OF_PROTEIN_IMPORT	104	0.35	1.63	0	0.034	1
GO_NUCLEAR_ENVELOPE_REASSEMBLY	17	0.54	1.63	0.02	0.035	1
GO_RESPONSE_TO_VITAMIN_E	15	0.55	1.63	0.023	0.035	1
GO_GLYCOSYL_COMPOUND_BIOSYNTHETIC_PROCESS	116	0.35	1.63	0.003	0.035	1
GO_CYTOPLASMIC_TRANSLATION	39	0.43	1.63	0.016	0.035	1
GO_GLAND_MORPHOGENESIS	97	0.35	1.63	0	0.035	1
GO_MACROPHAGE_DIFFERENTIATION	19	0.51	1.63	0.026	0.035	1
GO_NEGATIVE_REGULATION_OF_INTERLEUKIN_10_PRODUCTION	16	0.55	1.63	0.022	0.035	1
GO_NEGATIVE_REGULATION_OF_CELL_ACTIVATION	154	0.33	1.63	0	0.035	1
GO_REGULATION_OF_TYROSINE_PHOSPHORYLATION_OF_STAT3_PROT	44	0.42	1.63	0.007	0.035	1
GO_DETECTION_OF_BIOTIC_STIMULUS	24	0.48	1.63	0.02	0.035	1
GO_OXIDOREDUCTASE_ACTIVITY_ACTING_ON_PAIRED_DONORS_WITH_INCORPORATION_OR_REDUCTION_OF_MOLECULAR_OXYGEN_NAD_P_H_AS_ONE_DONOR_AND_INCORPORATION_OF_ONE_ATOM_OF_OXYGEN	36	0.44	1.63	0.012	0.035	1
GO_NEGATIVE_REGULATION_OF_CHROMOSOME_ORGANIZATION	96	0.36	1.63	0.002	0.035	1
GO_CYTOPLASMIC_EXOSOME_RNASE_COMPLEX_	15	0.54	1.63	0.026	0.035	1
GO_REGULATION_OF_CHEMOKINE_PRODUCTION	65	0.39	1.63	0.005	0.036	1
GO_POSITIVE_REGULATION_OF_ACUTE_INFLAMMATORY_RESPONSE	28	0.46	1.63	0.004	0.036	1
GO_LAMIN_BINDING	17	0.52	1.63	0.017	0.036	1
GO_PROTEIN_POLYUBIQUITINATION	239	0.31	1.63	0	0.036	1
GO_NUCLEOTIDE_PHOSPHORYLATION	57	0.39	1.62	0.01	0.036	1
GO_GALACTOSYLTRANSFERASE_ACTIVITY	32	0.45	1.62	0.012	0.036	1
GO_NEGATIVE_REGULATION_OF_APOPTOTIC_SIGNALING_PATHWAY	200	0.32	1.62	0	0.037	1
GO_PYRIMIDINE_NUCLEOSIDE_TRIPHOSPHATE_BIOSYNTHETIC_PROCES	19	0.5	1.62	0.021	0.037	1
GO_HOMOLOGOUS_CHROMOSOME_SEGREGATION	44	0.42	1.62	0.01	0.037	1
GO_POSITIVE_REGULATION_OF_NUCLEASE_ACTIVITY	15	0.55	1.62	0.019	0.037	1
GO_CELLULAR_RESPONSE_TO_UV	65	0.38	1.62	0.005	0.037	1
GO_PURINE_NUCLEOSIDE_MONOPHOSPHATE_BIOSYNTHETIC_PROCESS	61	0.38	1.62	0.007	0.037	1
GO_NEGATIVE_REGULATION_OF_CD4_POSITIVE_ALPHA_BETA_T_CELL_ACTIVATION	15	0.54	1.62	0.035	0.038	1
GO_POSITIVE_REGULATION_OF_HEMOPOIESIS	160	0.33	1.62	0	0.038	1
GO_EPITHELIAL_CELL_APOPTOTIC_PROCESS	25	0.49	1.62	0.022	0.038	1
GO_NUCLEAR_ENVELOPE	406	0.29	1.62	0	0.038	1
GO_RESPONSE_TO_NUTRIENT	191	0.32	1.62	0	0.038	1
GO_CALCIUM_DEPENDENT_CYSTEINE_TYPE_ENDOPEPTIDASE_ACTIVITY	21	0.49	1.61	0.018	0.038	1
GO_NUCLEAR_HETEROCHROMATIN	32	0.44	1.61	0.011	0.039	1
GO_PORE_COMPLEX_ASSEMBLY	16	0.54	1.61	0.017	0.039	1
GO_WIDE_PORE_CHANNEL_ACTIVITY	23	0.49	1.61	0.023	0.039	1
GO_REGULATION_OF_RELEASE_OF_CYTOCHROME_C_FROM_MITOCHON	43	0.42	1.61	0.009	0.039	1
GO_PHOSPHATIDYLGLYCEROL_ACYL_CHAIN_REMODELING	16	0.54	1.61	0.033	0.039	1
GO_REGULATION_OF_NUCLEASE_ACTIVITY	23	0.48	1.61	0.017	0.039	1
GO_RESPONSE_TO_HYDROGEN_PEROXIDE	109	0.34	1.61	0	0.039	1
GO_RNA_HELICASE_ACTIVITY	65	0.38	1.61	0.003	0.039	1
GO_RESPONSE_TO_RADIATION	406	0.29	1.61	0	0.04	1
GO_NEGATIVE_REGULATION_OF_TYPE_I_INTERFERON_PRODUCTION	39	0.43	1.61	0.019	0.04	1
GO_REGULATION_OF_MYOBLAST_FUSION	19	0.52	1.61	0.018	0.04	1
GO_MONOSACCHARIDE_BIOSYNTHETIC_PROCESS	54	0.4	1.61	0.005	0.04	1
GO_CYTOKINE_PRODUCTION	119	0.34	1.61	0.003	0.04	1
GO_IMMUNOGLOBULIN_PRODUCTION_INVOLVED_IN_IMMUNOGLOBULIN_MEDIATED_IMMUNE_RESPONSE	22	0.48	1.6	0.02	0.041	1
GO_NEGATIVE_REGULATION_OF_DNA_REPLICATION	52	0.4	1.6	0.011	0.041	1
GO_MITOCHONDRIAL_MATRIX	406	0.29	1.6	0	0.041	1
GO_HUMORAL_IMMUNE_RESPONSE_MEDIATED_BY_CIRCULATING_IMMUNOGLOBULIN	36	0.43	1.6	0.007	0.041	1
GO_TRANSCRIPTION_COUPLED_NUCLEOTIDE_EXCISION_REPAIR	73	0.37	1.6	0.002	0.041	1
GO_RESPONSE_TO_ENDOPLASMIC_RETICULUM_STRESS	229	0.3	1.6	0	0.041	1
GO_HUMORAL_IMMUNE_RESPONSE	149	0.33	1.6	0	0.041	1
GO_PYRIMIDINE_NUCLEOSIDE_TRIPHOSPHATE_METABOLIC_PROCESS	21	0.49	1.6	0.024	0.041	1
GO_RRNA_CATABOLIC_PROCESS	16	0.54	1.6	0.031	0.041	1
GO_REGULATION_OF_T_CELL_MEDIATED_CYTOTOXICITY	23	0.49	1.6	0.026	0.041	1
GO_RNA_POLYMERASE_COMPLEX	120	0.34	1.6	0	0.041	1
GO_NEGATIVE_REGULATION_OF_RESPONSE_TO_BIOTIC_STIMULUS	29	0.46	1.6	0.009	0.042	1
GO_CELLULAR_RESPONSE_TO_MECHANICAL_STIMULUS	79	0.36	1.6	0.005	0.042	1
GO_NEGATIVE_REGULATION_OF_STEROID_METABOLIC_PROCESS	23	0.49	1.6	0.035	0.042	1
GO_TRANSCRIPTION_ELONGATION_FROM_RNA_POLYMERASE_II_PROM	80	0.36	1.6	0	0.042	1
GO_APOPTOTIC_DNA_FRAGMENTATION	15	0.53	1.6	0.026	0.042	1
GO_DEOXYRIBONUCLEOSIDE_TRIPHOSPHATE_METABOLIC_PROCESS	17	0.52	1.6	0.03	0.042	1
GO_EUCHROMATIN	31	0.44	1.6	0.025	0.042	1
GO_REGULATION_OF_RETINOIC_ACID_RECEPTOR_SIGNALING_PATHWA	29	0.45	1.6	0.017	0.042	1
GO_REGULATION_OF_LAMELLIPODIUM_ORGANIZATION	36	0.42	1.6	0.013	0.042	1
GO_ISOPRENOID_METABOLIC_PROCESS	125	0.33	1.6	0	0.042	1
GO_MAMMARY_GLAND_DEVELOPMENT	117	0.34	1.6	0.003	0.042	1
GO_UDP_GALACTOSYLTRANSFERASE_ACTIVITY	28	0.45	1.6	0.029	0.042	1
GO_EXTRINSIC_APOPTOTIC_SIGNALING_PATHWAY	99	0.35	1.6	0	0.042	1
GO_POSITIVE_REGULATION_OF_B_CELL_ACTIVATION	66	0.37	1.6	0.007	0.042	1
GO_ALDO_KETO_REDUCTASE_NADP_ACTIVITY	26	0.47	1.59	0.016	0.042	1
GO_POSITIVE_REGULATION_OF_TELOMERASE_ACTIVITY	28	0.46	1.59	0.022	0.043	1
GO_NUCLEOTIDE_SUGAR_BIOSYNTHETIC_PROCESS	19	0.5	1.59	0.024	0.043	1
GO_REGULATION_OF_NEUTROPHIL_CHEMOTAXIS	27	0.45	1.59	0.016	0.043	1
GO_HEPATICOBILIARY_SYSTEM_DEVELOPMENT	124	0.33	1.59	0	0.043	1

GO_REGULATION_OF_LEUKOCYTE_DIFFERENTIATION	230	0.31	1.59	0	0.043	1
GO_SNRNA_PROCESSING	22	0.49	1.59	0.02	0.043	1
GO_POSITIVE_REGULATION_OF_MONOCYTE_CHEMOTAXIS	15	0.53	1.59	0.03	0.043	1
GO_POSITIVE_REGULATION_OF_TOLL_LIKE_RECEPTOR_SIGNALING_PATHWAY	19	0.5	1.59	0.018	0.043	1
GO_RESPONSE_TO_FUNGUS	49	0.39	1.59	0.011	0.043	1
GO_RIBOSOME_BINDING	45	0.4	1.59	0	0.043	1
GO_TRANSFERASE_ACTIVITY_TRANSFERRING_HEXOSYL_GROUPS	197	0.31	1.59	0	0.043	1
GO_RRNA_BINDING	56	0.39	1.59	0.005	0.044	1
GO_ALDITOL_METABOLIC_PROCESS	20	0.49	1.59	0.019	0.044	1
GO_POSITIVE_REGULATION_OF_MEMBRANE_PROTEIN_ECTODOMAIN_PROTEOLYSIS	15	0.53	1.59	0.02	0.044	1
GO_PEPTIDYL_LYSINE_MODIFICATION	298	0.3	1.59	0	0.045	1
GO_ENDODERM_FORMATION	50	0.39	1.59	0.002	0.045	1
GO_SERINE_TYPE_ENDOPEPTIDASE_INHIBITOR_ACTIVITY	91	0.35	1.59	0.002	0.045	1
GO_CHROMATIN_SILENCING	86	0.35	1.59	0	0.045	1
GO_RETINOL_DEHYDROGENASE_ACTIVITY	18	0.51	1.58	0.027	0.045	1
GO_REGULATION_OF_LEUKOCYTE_APOPTOTIC_PROCESS	77	0.36	1.58	0.002	0.045	1
GO_PROTEIN_LOCALIZATION_TO_CYTOSKELETON	29	0.45	1.58	0.024	0.045	1
GO_TRANSLATION_INITIATION_FACTOR_ACTIVITY	49	0.4	1.58	0.019	0.045	1
GO_METALLOPEPTIDASE_ACTIVITY	184	0.31	1.58	0	0.045	1
GO_REGULATION_OF_INTERFERON_ALPHA_PRODUCTION	19	0.51	1.58	0.04	0.046	1
GO_RESPONSE_TO_LIPOPROTEIN_PARTICLE	20	0.5	1.58	0.017	0.046	1
GO_INTRINSIC_APOPTOTIC_SIGNALING_PATHWAY_IN_RESPONSE_TO_DNA_DAMAGE	70	0.36	1.58	0.005	0.046	1
GO_NUCLEAR_EXOSOME_RNASE_COMPLEX_	15	0.54	1.58	0.036	0.046	1
GO_EXTRACELLULAR_STRUCTURE_ORGANIZATION	299	0.29	1.58	0	0.046	1
GO_DEATH_RECEPTOR_BINDING	17	0.53	1.58	0.028	0.046	1
GO_NON_RECOMBINATIONAL_REPAIR	65	0.37	1.58	0.005	0.046	1
GO_SERINE_HYDROLASE_ACTIVITY	210	0.31	1.58	0	0.046	1
GO_BILE_ACID_AND_BILE_SALT_TRANSPORT	31	0.45	1.58	0.018	0.046	1
GO_NEGATIVE_REGULATION_OF_CELL_ADHESION	219	0.31	1.58	0	0.046	1
GO_AGING	261	0.3	1.58	0.003	0.046	1
GO_GLAND_DEVELOPMENT	390	0.28	1.58	0	0.046	1
GO_DNA_DAMAGE_RESPONSE_SIGNAL_TRANSDUCTION_RESULTING_IN_TRANSCRIPTION	15	0.53	1.58	0.027	0.046	1
GO_DETECTION_OF_OTHER_ORGANISM	17	0.51	1.58	0.034	0.046	1
GO_INTERMEDIATE_FILAMENT_BASED_PROCESS	43	0.4	1.58	0.017	0.047	1
GO_REGULATION_OF_TRANSLATIONAL_ELONGATION	23	0.47	1.58	0.038	0.047	1
GO_HYDROLASE_ACTIVITY_ACTING_ON_CARBON_NITROGEN_BUT_NOT_PEPTIDE_BONDS_IN_CYCLIC_AMIDINES	34	0.43	1.58	0.013	0.047	1
GO_PROTEIN_FOLDING	217	0.3	1.58	0	0.047	1
GO_EMBRYONIC_PLACENTA_MORPHOGENESIS	22	0.48	1.57	0.023	0.048	1
GO_T_CELL_LINEAGE_COMMITMENT	15	0.53	1.57	0.031	0.048	1
GO_NON_MEMBRANE_SPANNING_PROTEIN_TYROSINE_KINASE_ACTIVITY	45	0.4	1.57	0	0.048	1
GO_REGULATION_OF_INTERLEUKIN_17_PRODUCTION	21	0.48	1.57	0.016	0.048	1
GO_OXIDOREDUCTASE_ACTIVITY_OXIDIZING_METAL_IONS	17	0.51	1.57	0.042	0.048	1
GO_CYTOKINE_RECEPTOR_ACTIVITY	89	0.35	1.57	0.005	0.049	1
GO_REGULATION_OF_CYCLIN_DEPENDENT_PROTEIN_KINASE_ACTIVITY	94	0.35	1.57	0.005	0.049	1
GO_RESPONSE_TO_TESTOSTERONE	38	0.41	1.57	0.016	0.05	1
GO_CYTOSOLIC_PART	217	0.3	1.57	0	0.05	1
GO_CARBOHYDRATE_CATABOLIC_PROCESS	112	0.33	1.57	0.005	0.05	1
GO_NONRIBOSOMAL_PEPTIDE_BIOSYNTHETIC_PROCESS	16	0.52	1.56	0.034	0.05	1
GO_CYTOKINE_METABOLIC_PROCESS	17	0.51	1.56	0.039	0.051	1
GO_FEMALE_MEIOTIC_DIVISION	25	0.46	1.56	0.02	0.052	1
GO_LEUKOCYTE_DIFFERENTIATION	292	0.29	1.56	0	0.052	1
GO_RESPONSE_TO_OXIDATIVE_STRESS	352	0.28	1.56	0	0.052	1
GO_POSITIVE_REGULATION_OF_CELL_KILLING	38	0.41	1.56	0.031	0.052	1
GO_LIGASE_ACTIVITY_FORMING_CARBON_NITROGEN_BONDS	55	0.38	1.56	0.017	0.052	1
GO_XENOPHAGY	102	0.33	1.56	0.011	0.052	1
GO_RESPONSE_TO_DEXAMETHASONE	33	0.43	1.56	0.023	0.052	1
GO_POSITIVE_REGULATION_OF_RELEASE_OF_CYTOCHROME_C_FROM_MITOCHONDRIA	27	0.44	1.56	0.018	0.052	1
GO_NEGATIVE_REGULATION_OF_T_CELL_MEDIATED_IMMUNITY	16	0.51	1.56	0.031	0.052	1
GO_POSITIVE_REGULATION_OF_CHROMOSOME_ORGANIZATION	145	0.32	1.56	0.002	0.053	1
GO_NCRNA_CATABOLIC_PROCESS	21	0.49	1.56	0.03	0.053	1
GO_BICELLULAR_TIGHT_JUNCTION_ASSEMBLY	31	0.43	1.56	0.021	0.053	1
GO_REGULATION_OF_INTRINSIC_APOPTOTIC_SIGNALING_PATHWAY	144	0.32	1.56	0.002	0.053	1
GO_NUCLEOTIDE_EXCISION_REPAIR_DNA_INCISION	38	0.41	1.55	0.025	0.054	1
GO_MIRNA_BINDING	16	0.52	1.55	0.04	0.054	1
GO_POSITIVE_REGULATION_OF_VIRAL_GENOME_REPLICATION	30	0.44	1.55	0.017	0.054	1
GO_RECIPROCAL_DNA_RECOMBINATION	37	0.41	1.55	0.021	0.054	1
GO_APOPTOTIC_SIGNALING_PATHWAY	286	0.29	1.55	0	0.054	1
GO_REGULATION_OF_IMMUNOGLOBULIN_SECRETION	17	0.51	1.55	0.041	0.054	1
GO_CHROMOSOME_ORGANIZATION_INVOLVED_IN_MEIOTIC_CELL_CYCL	47	0.39	1.55	0.012	0.055	1
GO_APOPTOTIC_NUCLEAR_CHANGES	25	0.46	1.55	0.023	0.055	1
GO_LYMPHOCYTE_HOMEOSTASIS	50	0.38	1.55	0.009	0.055	1
GO_T_CELL_HOMEOSTASIS	34	0.42	1.55	0.023	0.055	1
GO_POSITIVE_REGULATION_OF_ACTIVATED_T_CELL_PROLIFERATION	28	0.45	1.55	0.041	0.055	1
GO_RIBOSOMAL_SMALL_SUBUNIT_ASSEMBLY	17	0.51	1.55	0.044	0.055	1
GO_NEGATIVE_REGULATION_OF_VIRAL_ENTRY_INTO_HOST_CELL	19	0.49	1.55	0.033	0.056	1
GO_REGULATION_OF_HAIR_CYCLE	22	0.47	1.55	0.034	0.056	1
GO_REGULATION_OF_SISTER_CHROMATID_COHESION	17	0.49	1.54	0.032	0.057	1
GO_STEROID_BIOSYNTHETIC_PROCESS	112	0.33	1.54	0	0.057	1
GO_ATPASE_ACTIVITY_COUPLED	308	0.29	1.54	0	0.056	1
GO_PROTEASE_BINDING	101	0.34	1.54	0	0.057	1
GO_UNSATURATED_FATTY_ACID_METABOLIC_PROCESS	104	0.33	1.54	0.002	0.057	1

GO_TRANSLATIONAL_INITIATION	144	0.32	1.54	0.003	0.057	1
GO_BLASTOCYST_FORMATION	30	0.43	1.54	0.02	0.057	1
GO_EXTERNAL_SIDE_OF_PLASMA_MEMBRANE	212	0.3	1.54	0	0.057	1
GO_PEPTIDYL_CYSTEINE_MODIFICATION	20	0.48	1.54	0.048	0.057	1
GO_NEGATIVE_REGULATION_OF_CYSTEINE_TYPE_ENDOPEPTIDASE_ACTIVITY	88	0.34	1.54	0.007	0.057	1
GO_TRANSLATION_FACTOR_ACTIVITY_RNA_BINDING	85	0.34	1.54	0.008	0.057	1
GO_PROTEIN_IMPORT_INTO_NUCLEUS_TRANSLOCATION	29	0.43	1.54	0.015	0.057	1
GO_MOLTING_CYCLE	83	0.35	1.54	0.007	0.057	1
GO_RECEPTOR_REGULATOR_ACTIVITY	44	0.4	1.54	0.014	0.058	1
GO_NEGATIVE_REGULATION_OF_RELEASE_OF_CYTOCHROME_C_FROM_MITOCHONDRIA	17	0.5	1.54	0.044	0.058	1
GO_REGULATION_OF_SPINDLE_ORGANIZATION	18	0.49	1.54	0.035	0.058	1
GO_REGULATION_OF_PROTEASOMAL_UBIQUITIN_DEPENDENT_PROTEIN_CATABOLIC_PROCESS	146	0.31	1.54	0.003	0.058	1
GO_GLUTAMINE_FAMILY_AMINO_ACID_METABOLIC_PROCESS	64	0.36	1.54	0.018	0.058	1
GO_ADP_METABOLIC_PROCESS	45	0.39	1.54	0.021	0.059	1
GO_REGULATION_OF_FIBROBLAST_PROLIFERATION	80	0.34	1.54	0.002	0.059	1
GO_REGULATION_OF_TRANSFORMING_GROWTH_FACTOR_BETA_PRODUCTION	25	0.44	1.53	0.037	0.059	1
GO_REGULATION_OF_MEIOTIC_NUCLEAR_DIVISION	29	0.43	1.53	0.021	0.06	1
GO_AMIDE_TRANSMEMBRANE_TRANSPORTER_ACTIVITY	19	0.49	1.53	0.036	0.06	1
GO_REGULATION_OF_CHROMATIN_SILENCING	21	0.48	1.53	0.02	0.061	1
GO_CENTRIOLE	93	0.33	1.53	0.012	0.061	1
GO_FUCOSE_METABOLIC_PROCESS	16	0.5	1.53	0.043	0.061	1
GO_REGULATION_OF_GRANULOCYTE_MACROPHAGE_COLONY_STIMULATING_FACTOR_PRODUCTION	15	0.51	1.53	0.056	0.061	1
GO_I_KAPPAB_KINASE_NF_KAPPAB_SIGNALING	70	0.36	1.53	0.012	0.061	1
GO_CELL_SUBSTRATE_JUNCTION	395	0.28	1.53	0	0.062	1
GO_MODULATION_BY_SYMBIONT_OF_HOST_CELLULAR_PROCESS	28	0.44	1.53	0.034	0.062	1
GO_NUCLEOSIDE_TRIPHOSPHATE_METABOLIC_PROCESS	221	0.29	1.53	0.003	0.062	1
GO_POSITIVE_REGULATION_OF_COAGULATION	25	0.45	1.53	0.024	0.063	1
GO_IRE1_MEDIATED_UNFOLDED_PROTEIN_RESPONSE	55	0.37	1.53	0.014	0.063	1
GO_REGULATION_OF_MONONUCLEAR_CELL_MIGRATION	16	0.51	1.52	0.056	0.063	1
GO_RESPONSE_TO_INCREASED_OXYGEN_LEVELS	23	0.45	1.52	0.043	0.063	1
GO_T_HELPER_1_TYPE_IMMUNE_RESPONSE	19	0.48	1.52	0.037	0.063	1
GO_T_CELL_SELECTION	36	0.4	1.52	0.026	0.063	1
GO_PROTEIN_UBIQUITINATION_INVOLVED_IN_UBIQUITIN_DEPENDENT_PROTEIN_CATABOLIC_PROCESS	133	0.32	1.52	0.005	0.064	1
GO_ANCHORING_JUNCTION	484	0.27	1.52	0	0.064	1
GO_PYRIMIDINE_CONTAINING_COMPOUND_BIOSYNTHETIC_PROCESS	40	0.4	1.52	0.017	0.064	1
GO_PYRIMIDINE_NUCLEOSIDE_BIOSYNTHETIC_PROCESS	30	0.42	1.52	0.034	0.064	1
GO_POSITIVE_REGULATION_OF_SEQUENCE_SPECIFIC_DNA_BINDING_TRANSCRIPTION_FACTOR_ACTIVITY	225	0.29	1.52	0	0.064	1
GO_MEMBRANE_RAFT_ORGANIZATION	17	0.5	1.52	0.05	0.064	1
GO_REGULATION_OF_COAGULATION	88	0.34	1.52	0.015	0.065	1
GO_ER_NUCLEUS_SIGNALING_PATHWAY	34	0.42	1.52	0.023	0.065	1
GO_TELOMERE_MAINTENANCE_VIA_TELOMERE_LENGTHENING	25	0.44	1.52	0.035	0.065	1
GO_REGULATION_OF_INTERLEUKIN_2_PRODUCTION	48	0.38	1.52	0.024	0.065	1
GO_REGULATION_OF_HEMOPOIESIS	308	0.28	1.52	0	0.066	1
GO_PROTEIN_TRIMERIZATION	39	0.39	1.52	0.025	0.066	1
GO_MAGNESIUM_ION_BINDING	195	0.3	1.52	0	0.066	1
GO_ENDOPLASMIC_RETICULUM_LUMEN	198	0.3	1.52	0.003	0.066	1
GO_U1_SNRNP	17	0.5	1.52	0.04	0.066	1
GO_IN_UTERO_EMBRYONIC_DEVELOPMENT	308	0.28	1.52	0	0.066	1
GO_VITAMIN_TRANSPORTER_ACTIVITY	24	0.45	1.52	0.027	0.066	1
GO_CELLULAR_RESPONSE_TO_ABIOTIC_STIMULUS	259	0.29	1.51	0	0.066	1
GO_NAD_BIOSYNTHETIC_PROCESS	15	0.51	1.51	0.04	0.067	1
GO_DEOXYRIBONUCLEOTIDE_METABOLIC_PROCESS	34	0.41	1.51	0.029	0.067	1
GO_CELLULAR_RESPONSE_TO_VIRUS	22	0.46	1.51	0.035	0.067	1
GO_RESPONSE_TO_TOPOLOGICALLY_INCORRECT_PROTEIN	162	0.3	1.51	0	0.067	1
GO_U4_U6_X_U5_TRI_SNRNP_COMPLEX	21	0.47	1.51	0.039	0.067	1
GO_CELLULAR_RESPONSE_TO_DSRNA	36	0.41	1.51	0.038	0.067	1
GO_REGULATION_OF_CELL_AGING	32	0.42	1.51	0.028	0.068	1
GO_COPPER_ION_TRANSPORT	19	0.47	1.51	0.045	0.068	1
GO_U5_SNRNP	16	0.5	1.51	0.055	0.068	1
GO_POSITIVE_REGULATION_OF_PROTEIN_POLYMERIZATION	87	0.34	1.51	0.009	0.069	1
GO_OXIDOREDUCTASE_ACTIVITY_ACTING_ON_THE_CH_CH_GROUP_OF_DONORS	57	0.36	1.51	0.019	0.069	1
GO_NEGATIVE_REGULATION_OF_CELL_KILLING	18	0.49	1.51	0.038	0.069	1
GO_CELLULAR_RESPONSE_TO_TOPOLOGICALLY_INCORRECT_PROTEIN	121	0.32	1.51	0.005	0.069	1
GO_LAMININ_BINDING	30	0.42	1.51	0.025	0.069	1
GO_LEUKOCYTE_HOMEOSTASIS	59	0.36	1.51	0.012	0.069	1
GO_REGULATION_OF_INFLAMMATORY_RESPONSE_TO_ANTIGENIC_STIM	18	0.48	1.51	0.045	0.069	1
GO_POSITIVE_REGULATION_OF_INTERFERON_BETA_PRODUCTION	30	0.42	1.5	0.031	0.07	1
GO_POSITIVE_REGULATION_OF_RESPONSE_TO_EXTERNAL_STIMULUS	294	0.28	1.5	0	0.07	1
GO_UBIQUITIN_LIKE_PROTEIN_BINDING	119	0.31	1.5	0.005	0.07	1
GO_RETROGRADE_VESICLE_MEDIATED_TRANSPORT_GOLGI_TO_ER	77	0.34	1.5	0.021	0.07	1
GO_REGULATION_OF_TUMOR_NECROSIS_FACTOR_MEDIATED_SIGNALING_PATHWAY	49	0.37	1.5	0.02	0.07	1
GO_POSITIVE_REGULATION_OF_CHEMOTAXIS	120	0.32	1.5	0.016	0.07	1
GO_MICROTUBULE_ASSOCIATED_COMPLEX	143	0.31	1.5	0.003	0.07	1
GO_RNA_METHYLATION	48	0.38	1.5	0.017	0.07	1
GO_DNA_REPAIR_COMPLEX	39	0.39	1.5	0.025	0.071	1
GO_DNA_DIRECTED_RNA_POLYMERASE_III_COMPLEX	18	0.48	1.5	0.054	0.071	1
GO_POSITIVE_REGULATION_OF_CELLULAR_PROTEIN_LOCALIZATION	356	0.27	1.5	0	0.071	1
GO_FATTY_ACID_DERIVATIVE_BIOSYNTHETIC_PROCESS	43	0.39	1.5	0.026	0.071	1

GO_TRNA_METHYLATION	22	0.45	1.5	0.058	0.072	1
GO_PEPTIDASE_ACTIVATOR_ACTIVITY	38	0.4	1.5	0.035	0.072	1
GO_REGULATION_OF_NF_KAPPAB_IMPORT_INTO_NUCLEUS	48	0.37	1.5	0.023	0.072	1
GO_FC_GAMMA_RECEPTOR_SIGNALING_PATHWAY	73	0.34	1.5	0.012	0.072	1
GO_CELLULAR_RESPONSE_TO_EXTERNAL_STIMULUS	261	0.28	1.5	0.003	0.072	1
GO_CENTROSOME	460	0.26	1.5	0	0.073	1
GO_REGULATION_OF_PROTEASOMAL_PROTEIN_CATABOLIC_PROCESS	179	0.3	1.5	0	0.073	1
GO_TOLL_LIKE_RECEPTOR_4_SIGNALING_PATHWAY	18	0.48	1.49	0.052	0.073	1
GO_REGULATION_OF_INTERLEUKIN_6_BIOSYNTHETIC_PROCESS	16	0.48	1.49	0.06	0.074	1
GO_MHC_PROTEIN_COMPLEX_BINDING	19	0.47	1.49	0.052	0.074	1
GO_POSITIVE_REGULATION_OF_VIRAL_TRANSCRIPTION	39	0.4	1.49	0.018	0.074	1
GO_MAMMARY_GLAND_DUCT_MORPHOGENESIS	28	0.42	1.49	0.044	0.074	1
GO_PYRIMIDINE_NUCLEOTIDE_METABOLIC_PROCESS	45	0.37	1.49	0.021	0.075	1
GO_REGULATION_OF_CELLULAR_AMIDE_METABOLIC_PROCESS	347	0.27	1.49	0	0.075	1
GO_RETINOIC_ACID_METABOLIC_PROCESS	21	0.45	1.49	0.052	0.075	1
GO_UNFOLDED_PROTEIN_BINDING	92	0.32	1.49	0.01	0.075	1
GO_RESPONSE_TO_CORTICOSTEROID	176	0.29	1.49	0.008	0.075	1
GO_CHROMOSOME_SEPARATION	20	0.46	1.49	0.055	0.075	1
GO_NEGATIVE_REGULATION_OF_LYMPHOCYTE_DIFFERENTIATION	40	0.39	1.49	0.031	0.075	1
GO_REGULATION_OF_MACROPHAGE_ACTIVATION	26	0.43	1.49	0.05	0.075	1
GO_MICROTUBULE_BINDING	195	0.29	1.49	0	0.076	1
GO_MALE_GERM_CELL_NUCLEUS	16	0.49	1.49	0.049	0.076	1
GO_REGULATION_OF_HETEROTYPIC_CELL_CELL_ADHESION	18	0.47	1.49	0.055	0.077	1
GO_POSITIVE_REGULATION_OF_LEUKOCYTE_DEGRANULATION	18	0.48	1.49	0.065	0.077	1
GO_PRIMARY_ALCOHOL_METABOLIC_PROCESS	47	0.37	1.48	0.019	0.077	1
GO_CONNEXON_COMPLEX	20	0.46	1.48	0.051	0.078	1
GO_POST_ANAL_TAIL_MORPHOGENESIS	18	0.47	1.48	0.056	0.078	1
GO_BRUSH_BORDER	102	0.32	1.48	0.005	0.078	1
GO_NEGATIVE_REGULATION_OF_DNA_DEPENDENT_DNA_REPLICATION	16	0.49	1.48	0.044	0.078	1
GO_APICAL_JUNCTION_ASSEMBLY	39	0.38	1.48	0.052	0.078	1
GO_CHAPERONE_MEDIATED_PROTEIN_FOLDING	47	0.38	1.48	0.034	0.078	1
GO_REGULATION_OF_CELL_ADHESION_MEDIATED_BY_INTEGRIN	38	0.4	1.48	0.03	0.078	1
GO_NEGATIVE_REGULATION_OF_CELL_CYCLE_G2_M_PHASE_TRANSITIO	25	0.43	1.48	0.057	0.078	1
GO_ARACHIDONIC_ACID_METABOLIC_PROCESS	49	0.36	1.48	0.035	0.078	1
GO_SMALL_MOLECULE_BIOSYNTHETIC_PROCESS	433	0.26	1.48	0	0.078	1
GO_NEGATIVE_REGULATION_OF_T_CELL_RECEPTOR_SIGNALING_PATH	16	0.49	1.48	0.082	0.079	1
GO_MRNA_CLEAVAGE_FACTOR_COMPLEX	17	0.48	1.48	0.056	0.079	1
GO_POSITIVE_REGULATION_OF_MYELOID_LEUKOCYTE_MEDIATED_IMMU	18	0.47	1.48	0.058	0.08	1
GO_NUCLEOSIDE_MONOPHOSPHATE_METABOLIC_PROCESS	236	0.28	1.48	0	0.08	1
GO_POSITIVE_REGULATION_OF_OSTEOCLAST_DIFFERENTIATION	23	0.44	1.48	0.04	0.08	1
GO_REGULATION_OF_ICOSANOID_SECRETION	20	0.47	1.48	0.043	0.081	1
GO_NUCLEOTIDE_EXCISION_REPAIR	109	0.31	1.48	0.007	0.081	1
GO_POSITIVE_REGULATION_OF_INTERLEUKIN_4_PRODUCTION	22	0.45	1.48	0.055	0.081	1
GO_RESPONSE_TO_ANTIBIOTIC	47	0.37	1.47	0.028	0.081	1
GO_REGULATION_OF_HISTONE_H3_K9_METHYLATION	17	0.48	1.47	0.059	0.081	1
GO_INFLAMMATORY_RESPONSE_TO_ANTIGENIC_STIMULUS	25	0.43	1.47	0.049	0.081	1
GO_MODULATION_BY_VIRUS_OF_HOST_MORPHOLOGY_OR_PHYSIOLOG	37	0.39	1.47	0.033	0.081	1
GO_NEGATIVE_REGULATION_OF_ANTIGEN_RECEPTOR_MEDIATED_SIGN ALING_PATHWAY	20	0.45	1.47	0.049	0.082	1
GO_RESPONSE_TO_ALCOHOL	361	0.27	1.47	0	0.082	1
GO_SINGLE_ORGANISM_CELL_ADHESION	454	0.26	1.47	0	0.082	1
GO_MONOSACCHARIDE_BINDING	70	0.34	1.47	0.012	0.082	1
GO_RESPONSE_TO_PROGESTERONE	49	0.36	1.47	0.028	0.082	1
GO_CELLULAR_RESPONSE_TO_REACTIVE_OXYGEN_SPECIES	104	0.31	1.47	0.007	0.083	1
GO_CELL_AGING	64	0.34	1.47	0.017	0.083	1
GO_CELLULAR_RESPONSE_TO_CORTICOSTEROID_STIMULUS	58	0.35	1.47	0.028	0.083	1
GO_NEGATIVE_REGULATION_OF_B_CELL_PROLIFERATION	15	0.5	1.47	0.053	0.083	1
GO_NEGATIVE_REGULATION_OF_CYTOKINE_SECRETION	43	0.38	1.47	0.041	0.083	1
GO_MODIFICATION_OF_MORPHOLOGY_OR_PHYSIOLOGY_OF_OTHER_OR GANISM	98	0.32	1.47	0.015	0.083	1
GO_POSITIVE_REGULATION_OF_INTERFERON_ALPHA_PRODUCTION	16	0.48	1.47	0.069	0.083	1
GO_RUFFLE_ORGANIZATION	20	0.46	1.47	0.048	0.083	1
GO_SMALL_RIBOSOMAL_SUBUNIT	66	0.34	1.47	0.022	0.083	1
GO_CHROMATIN_BINDING	430	0.26	1.47	0	0.084	1
GO_NEGATIVE_REGULATION_OF_LYMPHOCYTE_APOPTOTIC_PROCESS	27	0.43	1.47	0.051	0.084	1
GO_T_CELL_DIFFERENTIATION_INVOLVED_IN_IMMUNE_RESPONSE	29	0.41	1.47	0.055	0.084	1
GO_POSITIVE_REGULATION_OF_DNA_REPAIR	37	0.39	1.47	0.045	0.084	1
GO_POSITIVE_REGULATION_OF_CATABOLIC_PROCESS	393	0.26	1.47	0	0.084	1
GO_MORPHOGENESIS_OF_AN_EPITHELIAL_SHEET	43	0.38	1.47	0.029	0.084	1
GO_IMMUNOLOGICAL_SYNAPSE	33	0.4	1.47	0.041	0.084	1
GO_POSITIVE_REGULATION_OF_RESPONSE_TO_DNA_DAMAGE_STIMULU	63	0.34	1.46	0.021	0.085	1
GO_RESPONSE_TO_VITAMIN_D	33	0.39	1.46	0.026	0.085	1
GO_NEGATIVE_REGULATION_OF_INTERLEUKIN_2_PRODUCTION	16	0.48	1.46	0.05	0.085	1
GO_RACEMASE_AND_EPIMERASE_ACTIVITY	17	0.48	1.46	0.064	0.085	1
GO_INTERMEDIATE_FILAMENT_ORGANIZATION	20	0.45	1.46	0.042	0.085	1
GO_EPHRIN_RECEPTOR_SIGNALING_PATHWAY	85	0.33	1.46	0.012	0.086	1
GO_HYALURONAN_METABOLIC_PROCESS	30	0.41	1.46	0.042	0.086	1
GO_INTRINSIC_COMPONENT_OF_ENDOPLASMIC_RETICULUM_MEMBRAN	133	0.3	1.46	0.008	0.086	1
GO_DNA_CATABOLIC_PROCESS	27	0.41	1.46	0.05	0.086	1
GO_RESPONSE_TO_OSMOTIC_STRESS	63	0.34	1.46	0.019	0.086	1
GO_FOLIC_ACID_METABOLIC_PROCESS	17	0.47	1.46	0.069	0.086	1
GO_PROTEIN_LOCALIZATION_TO_NUCLEUS	155	0.29	1.46	0.012	0.087	1
GO_RESPONSE_TO_DSRNA	70	0.33	1.46	0.019	0.087	1
GO_CYSTEINE_TYPE_PEPTIDASE_ACTIVITY	166	0.3	1.46	0.005	0.087	1
GO_RRNA_TRANSCRIPTION	18	0.47	1.46	0.048	0.087	1
GO_POSITIVE_REGULATION_OF_LOCOMOTION	417	0.26	1.46	0	0.087	1
GO_REGULATION_OF_MAMMARY_GLAND_EPITHELIAL_CELL_PROLIFERAT	16	0.48	1.46	0.068	0.087	1

GO_STEROID_METABOLIC_PROCESS	232	0.28	1.46	0.006	0.087	1
GO_POSITIVE_REGULATION_OF_CELLULAR_AMIDE_METABOLIC_PROCES	109	0.31	1.46	0.01	0.087	1
GO_SYNAPTONEMAL_COMPLEX_ORGANIZATION	18	0.47	1.46	0.065	0.087	1
GO_REGULATION_OF_PROTEIN_TYROSINE_KINASE_ACTIVITY	61	0.35	1.46	0.02	0.088	1
GO_PROTEASOME_BINDING	15	0.5	1.46	0.071	0.088	1
GO_POSITIVE_T_CELL_SELECTION	21	0.44	1.46	0.061	0.088	1
GO_PIGMENT_METABOLIC_PROCESS	60	0.35	1.45	0.029	0.089	1
GO_REGULATION_OF_CYTOKINE_PRODUCTION_INVOLVED_IN_IMMUNE_RESPONSE	57	0.35	1.45	0.038	0.089	1
GO_MYOBLAST_DIFFERENTIATION	37	0.38	1.45	0.047	0.089	1
GO_DENDRITIC_CELL_DIFFERENTIATION	33	0.4	1.45	0.057	0.089	1
GO_REGULATION_OF_PEPTIDYL_TYROSINE_PHOSPHORYLATION	213	0.28	1.45	0.003	0.09	1
GO_LABYRINTHINE_LAYER_DEVELOPMENT	44	0.38	1.45	0.039	0.09	1
GO_ALPHA_AMINO_ACID_BIOSYNTHETIC_PROCESS	75	0.34	1.45	0.026	0.091	1
GO_CELLULAR_RESPONSE_TO_HYDROGEN_PEROXIDE	61	0.34	1.45	0.025	0.09	1
GO_REGULATION_OF_HISTONE_H3_K4_METHYLATION	27	0.41	1.45	0.05	0.091	1
GO_ORGANIC_CYCLIC_COMPOUND_CATABOLIC_PROCESS	422	0.26	1.45	0	0.091	1
GO_APOPTOTIC_PROCESS_INVOLVED_IN_MORPHOGENESIS	16	0.49	1.45	0.069	0.091	1
GO_HEAT_SHOCK_PROTEIN_BINDING	87	0.32	1.45	0.015	0.091	1
GO_REGULATION_OF_ACUTE_INFLAMMATORY_RESPONSE	73	0.33	1.45	0.021	0.091	1
GO_HETEROTYPIC_CELL_CELL_ADHESION	27	0.42	1.45	0.058	0.091	1
GO_CELLULAR_ALDEHYDE_METABOLIC_PROCESS	83	0.33	1.45	0.031	0.092	1
GO_GOLGI_LUMEN	87	0.32	1.45	0.013	0.092	1
GO_MICROTUBULE_POLYMERIZATION_OR_DEPOLYMERIZATION	41	0.38	1.45	0.04	0.092	1
GO_RNA_SECONDARY_STRUCTURE_UNWINDING	42	0.37	1.45	0.063	0.092	1
GO_CARBOHYDRATE_KINASE_ACTIVITY	20	0.45	1.45	0.062	0.092	1
GO_REGULATION_OF_MONOCYTE_CHEMOTAXIS	20	0.44	1.45	0.077	0.093	1
GO_LIPID_PARTICLE	62	0.34	1.44	0.032	0.093	1
GO_REGULATION_OF_CANONICAL_WNT_SIGNALING_PATHWAY	235	0.28	1.44	0	0.094	1
GO_T_CELL_ACTIVATION_INVOLVED_IN_IMMUNE_RESPONSE	60	0.35	1.44	0.047	0.094	1
GO_POSITIVE_REGULATION_OF_INTERLEUKIN_2_PRODUCTION	31	0.4	1.44	0.051	0.095	1
GO_LATERAL_PLASMA_MEMBRANE	49	0.37	1.44	0.021	0.095	1
GO_STRUCTURAL_CONSTITUENT_OF_CYTOSKELETON	98	0.31	1.44	0.015	0.095	1
GO_VITAMIN_TRANSPORT	34	0.39	1.44	0.046	0.095	1
GO_PHOSPHATIDYLSERINE_ACYL_CHAIN_REMODELING	16	0.48	1.44	0.064	0.096	1
GO_RNA_POLYMERASE_II_DISTAL_ENHANCER_SEQUENCE_SPECIFIC_DNA_BINDING	65	0.34	1.44	0.037	0.096	1
GO_INTRINSIC_APOPTOTIC_SIGNALING_PATHWAY_IN_RESPONSE_TO_DNA_DAMAGE_BY_P53_CLASS_MEDIATOR	29	0.4	1.44	0.061	0.096	1
GO_OLIGOSACCHARIDE_LIPID_INTERMEDIATE_BIOSYNTHETIC_PROCESS	20	0.45	1.44	0.074	0.096	1
GO_ENHANCER_BINDING	93	0.31	1.44	0.027	0.096	1
GO_AMINOPEPTIDASE_ACTIVITY	41	0.38	1.44	0.055	0.097	1
GO_POSITIVE_REGULATION_OF_MYELOID_CELL_DIFFERENTIATION	79	0.33	1.44	0.017	0.097	1
GO_MAINTENANCE_OF_GASTROINTESTINAL_EPITHELIUM	15	0.48	1.44	0.065	0.097	1
GO_TRANSFERASE_ACTIVITY_TRANSFERRING_AMINO_ACYL_GROUPS	23	0.43	1.44	0.059	0.097	1
GO_POSITIVE_REGULATION_OF_NUCLEOCYTOPLASMIC_TRANSPORT	121	0.3	1.43	0.008	0.098	1
GO_POSITIVE_REGULATION_OF_DNA_TEMPLATED_TRANSCRIPTION_INITIATION	24	0.43	1.43	0.05	0.098	1
GO_NEGATIVE_REGULATION_OF_TUMOR_NECROSIS_FACTOR_SUPERFAMILY_CYTOKINE_PRODUCTION	44	0.37	1.43	0.051	0.098	1
GO_ESTABLISHMENT_OF_EPITHELIAL_CELL_POLARITY	23	0.43	1.43	0.077	0.098	1
GO_PYRIMIDINE_RIBONUCLEOSIDE_METABOLIC_PROCESS	32	0.39	1.43	0.043	0.098	1
GO_POSITIVE_REGULATION_OF_MYELOID_LEUKOCYTE_DIFFERENTIATIO	49	0.35	1.43	0.032	0.098	1
GO_FUCOSYLATION	24	0.42	1.43	0.065	0.098	1
GO_MODIFICATION_BY_SYMBIONT_OF_HOST_MORPHOLOGY_OR_PHYSIOLOGY	45	0.37	1.43	0.057	0.098	1
GO_NEGATIVE_REGULATION_OF_VIRAL_TRANSCRIPTION	24	0.43	1.43	0.072	0.098	1
GO_PROTEIN_IMPORT	152	0.29	1.43	0.01	0.098	1
GO_MITOCHONDRIAL_TRANSPORT	171	0.29	1.43	0.005	0.099	1
GO_MAMMARY_GLAND_MORPHOGENESIS	40	0.38	1.43	0.044	0.1	1
GO_HISTONE_BINDING	171	0.28	1.43	0.006	0.1	1
GO_STAT_CASCADE	50	0.35	1.43	0.026	0.101	1
GO_POSTTRANSCRIPTIONAL_GENE_SILENCING	41	0.37	1.43	0.037	0.101	1
GO_POSITIVE_REGULATION_OF_LAMELLIPODIUM_ASSEMBLY	15	0.48	1.43	0.098	0.102	1
GO_GAP_JUNCTION	29	0.4	1.43	0.062	0.102	1
GO_PTERIDINE_CONTAINING_COMPOUND_METABOLIC_PROCESS	35	0.39	1.42	0.048	0.103	1
GO_UBIQUITIN_LIKE_PROTEIN_CONJUGATING_ENZYME_ACTIVITY	29	0.4	1.42	0.055	0.103	1
GO_INO80_TYPE_COMPLEX	21	0.43	1.42	0.06	0.103	1
GO_ADENYLYLTRANSFERASE_ACTIVITY	24	0.43	1.42	0.056	0.103	1
GO_QUINONE_METABOLIC_PROCESS	28	0.4	1.42	0.063	0.104	1
GO_INNATE_IMMUNE_RESPONSE_IN_MUCOSA	19	0.44	1.42	0.076	0.104	1
GO_COENZYME_BIOSYNTHETIC_PROCESS	124	0.3	1.42	0.026	0.104	1
GO_DNA_CATABOLIC_PROCESS_ENDONUCLEOLYTIC	19	0.44	1.42	0.088	0.105	1
GO_REGULATION_OF_INTERLEUKIN_5_PRODUCTION	19	0.45	1.42	0.103	0.105	1
GO_MICROVILLUS_MEMBRANE	19	0.45	1.42	0.072	0.105	1
GO_REGULATION_OF_PROTEIN_LOCALIZATION_TO_NUCLEUS	216	0.28	1.42	0	0.105	1
GO_PEPTIDYL_PROLINE_MODIFICATION	56	0.35	1.42	0.044	0.105	1
GO_CARBOHYDRATE_DERIVATIVE_TRANSPORT	48	0.36	1.42	0.051	0.105	1
GO_NEGATIVE_REGULATION_OF_RETINOIC_ACID_RECEPTOR_SIGNALING_PATHWAY	23	0.42	1.42	0.059	0.105	1
GO_ADP_BINDING	33	0.39	1.42	0.065	0.106	1
GO_DIGESTIVE_SYSTEM_PROCESS	61	0.34	1.42	0.031	0.106	1
GO_PYRUVATE_METABOLIC_PROCESS	63	0.33	1.42	0.03	0.106	1
GO_REGULATION_OF_B_CELL_PROLIFERATION	54	0.35	1.42	0.04	0.106	1
GO_CELLULAR_AMINO_ACID_METABOLIC_PROCESS	328	0.26	1.42	0.003	0.107	1
GO_MESODERMAL_CELL_DIFFERENTIATION	26	0.42	1.42	0.076	0.107	1
GO_TRANSFERASE_ACTIVITY_TRANSFERRING_PENTOSYL_GROUPS	54	0.34	1.42	0.035	0.107	1

GO_PROTEIN_TETRAMERIZATION	132	0.29	1.41	0.014	0.108	1
GO_SIDE_OF_MEMBRANE	399	0.25	1.41	0	0.108	1
GO_CD4_POSITIVE_ALPHA_BETA_T_CELL_ACTIVATION	34	0.38	1.41	0.053	0.108	1
GO_TRANSCRIPTION_FROM_RNA_POLYMERASE_I_PROMOTER	35	0.38	1.41	0.064	0.109	1
GO_REGULATION_OF_TOLL_LIKE_RECEPTOR_4_SIGNALING_PATHWAY	15	0.48	1.41	0.088	0.109	1
GO_NEGATIVE_REGULATION_OF_STEM_CELL_PROLIFERATION	17	0.45	1.41	0.059	0.109	1
GO_ESTABLISHMENT_OF_CELL_POLARITY	88	0.31	1.41	0.027	0.109	1
GO_WOUND_HEALING	465	0.25	1.41	0	0.109	1
GO_GUANOSINE_CONTAINING_COMPOUND_BIOSYNTHETIC_PROCESS	15	0.48	1.41	0.085	0.109	1
GO_RESPONSE_TO_SALT_STRESS	19	0.44	1.41	0.067	0.11	1
GO_RNA_CAPPING	37	0.38	1.41	0.074	0.111	1
GO_REGULATION_OF_CYTOKINE_PRODUCTION_INVOLVED_IN_INFLAMMATORY_RESPONSE	18	0.45	1.41	0.081	0.111	1
GO_CELL_JUNCTION_ORGANIZATION	182	0.28	1.41	0.011	0.111	1
GO_MALE_MEIOSIS_I	17	0.46	1.41	0.075	0.111	1
GO_PYRIMIDINE_NUCLEOSIDE_METABOLIC_PROCESS	49	0.35	1.41	0.038	0.111	1
GO_TRANSFERASE_COMPLEX_TRANSFERRING_PHOSPHORUS_CONTAINING_GROUPS	231	0.27	1.4	0.003	0.116	1
GO_NEGATIVE_REGULATION_OF_ALCOHOL_BIOSYNTHETIC_PROCESS	17	0.45	1.4	0.089	0.116	1
GO_SKIN_EPIDERMIS_DEVELOPMENT	71	0.32	1.4	0.043	0.116	1
GO_PLATELET_AGGREGATION	39	0.37	1.4	0.052	0.116	1
GO_EPOXYGENASE_P450_PATHWAY	18	0.44	1.4	0.087	0.116	1
GO_T_CELL_DIFFERENTIATION	123	0.29	1.4	0.005	0.116	1
GO_NEGATIVE_REGULATION_OF_CELL_AGING	16	0.46	1.4	0.082	0.116	1
GO_POSITIVE_REGULATION_OF_VASCULAR_ENDOTHELIAL_GROWTH_FACTOR_PRODUCTION	26	0.41	1.4	0.062	0.116	1
GO_REGULATION_OF_EXECUTION_PHASE_OF_APOPTOSIS	24	0.41	1.4	0.092	0.116	1
GO_ESTABLISHMENT_OR_MAINTENANCE_OF_MONOPOLAR_CELL_POLAR	16	0.46	1.4	0.095	0.116	1
GO_ALPHA_BETA_T_CELL_DIFFERENTIATION	45	0.36	1.4	0.043	0.117	1
GO_RNA_POLYADENYLATION	29	0.38	1.4	0.07	0.117	1
GO_NUCLEOSIDE_TRIPHOSPHATE_BIOSYNTHETIC_PROCESS	61	0.33	1.4	0.033	0.117	1
GO_EPITHELIAL_CELL_MATURATION	15	0.46	1.4	0.083	0.117	1
GO_CYTOPLASMIC_STRESS_GRANULE	31	0.39	1.4	0.05	0.117	1
GO_NEGATIVE_REGULATION_OF_COAGULATION	48	0.35	1.4	0.052	0.117	1
GO_REGENERATION	158	0.28	1.4	0.018	0.117	1
GO_MYELOID_LEUKOCYTE_ACTIVATION	97	0.3	1.4	0.023	0.117	1
GO_MICROTUBULE	397	0.25	1.4	0.003	0.117	1
GO_PHAGOCYTIC_VESICLE	84	0.31	1.4	0.036	0.118	1
GO_PYRIMIDINE_NUCLEOTIDE_BIOSYNTHETIC_PROCESS	30	0.39	1.4	0.076	0.118	1
GO_ALCOHOL_BIOSYNTHETIC_PROCESS	110	0.3	1.4	0.025	0.118	1
GO_SYNAPTONEMAL_COMPLEX	32	0.39	1.39	0.068	0.118	1
GO_GLYCOSYL_COMPOUND_METABOLIC_PROCESS	362	0.26	1.39	0	0.118	1
GO_REGULATION_OF_VASCULAR_ENDOTHELIAL_GROWTH_FACTOR_PRODUCTION	31	0.39	1.39	0.073	0.119	1
GO_EMBRYO_IMPLANTATION	37	0.37	1.39	0.065	0.119	1
GO_POSITIVE_REGULATION_OF_EXTRINSIC_APOPTOTIC_SIGNALING_PATHWAY	52	0.34	1.39	0.043	0.119	1
GO_TRANSFERASE_ACTIVITY_TRANSFERRING_GLYCOSYL_GROUPS	275	0.26	1.39	0.009	0.12	1
GO_FIBRONECTIN_BINDING	28	0.39	1.39	0.078	0.122	1
GO_ANTIMICROBIAL_HUMORAL_RESPONSE	43	0.35	1.39	0.049	0.122	1
GO_CARGO_RECEPTOR_ACTIVITY	65	0.32	1.39	0.028	0.122	1
GO_POSITIVE_REGULATION_OF_DNA_BINDING	42	0.36	1.39	0.063	0.123	1
GO_NUCLEAR_MEMBRANE	274	0.26	1.39	0.014	0.124	1
GO_BILE_ACID_TRANSMEMBRANE_TRANSPORTER_ACTIVITY	15	0.46	1.38	0.1	0.125	1
GO_SPLEEN_DEVELOPMENT	38	0.37	1.38	0.06	0.125	1
GO_PIGMENT_BIOSYNTHETIC_PROCESS	45	0.35	1.38	0.065	0.125	1
GO_REGULATION_OF_LEUKOCYTE_DEGRANULATION	41	0.36	1.38	0.048	0.125	1
GO_NEGATIVE_REGULATION_OF_RESPONSE_TO_WOUNDING	152	0.28	1.38	0.013	0.125	1
GO_FEMALE_SEX_DIFFERENTIATION	116	0.29	1.38	0.022	0.125	1
GO_PROTEIN_O_LINKED_GLYCOSYLATION	96	0.3	1.38	0.029	0.125	1
GO_REGULATION_OF_DNA_BINDING	93	0.3	1.38	0.018	0.126	1
GO_NEGATIVE_REGULATION_OF_NF_KAPPAB_TRANSCRIPTION_FACTOR_ACTIVITY	65	0.33	1.38	0.05	0.127	1
GO_RETINOIC_ACID_BINDING	22	0.42	1.38	0.074	0.127	1
GO_RNA_METHYLTRANSFERASE_ACTIVITY	39	0.36	1.38	0.073	0.127	1
GO_RIBOSOMAL_LARGE_SUBUNIT_ASSEMBLY	23	0.41	1.38	0.063	0.129	1
GO_POSITIVE_REGULATION_OF_ERBB_SIGNALING_PATHWAY	36	0.37	1.38	0.074	0.129	1
GO_CELLULAR_RESPONSE_TO_LIPID	451	0.24	1.38	0	0.13	1
GO_NEGATIVE_REGULATION_OF_FATTY_ACID_METABOLIC_PROCESS	28	0.39	1.38	0.088	0.13	1
GO_CARBOHYDRATE_BIOSYNTHETIC_PROCESS	121	0.29	1.38	0.018	0.13	1
GO_NUCLEAR_TRANSCRIBED_MRNA_POLY_A_TAIL_SHORTENING	25	0.4	1.37	0.069	0.131	1
GO_CELLULAR_RESPONSE_TO_GLUCOSE_STARVATION	30	0.39	1.37	0.071	0.131	1
GO_CELL_ACTIVATION_INVOLVED_IN_IMMUNE_RESPONSE	137	0.28	1.37	0.018	0.131	1
GO_UNSATURATED_FATTY_ACID_BIOSYNTHETIC_PROCESS	54	0.34	1.37	0.053	0.131	1
GO_POSITIVE_REGULATION_OF_PEPTIDYL_TYROSINE_PHOSPHORYLATI	162	0.27	1.37	0.005	0.132	1
GO_CELL_JUNCTION_ASSEMBLY	127	0.29	1.37	0.039	0.132	1
GO_NEGATIVE_REGULATION_OF_LEUKOCYTE_APOPTOTIC_PROCESS	43	0.35	1.37	0.058	0.133	1
GO_NEGATIVE_REGULATION_OF_INTERLEUKIN_1_PRODUCTION	18	0.44	1.37	0.095	0.133	1
GO_BETA_1_3_GALACTOSYLTRANSFERASE_ACTIVITY	15	0.46	1.37	0.092	0.133	1
GO_MICROTUBULE_END	22	0.42	1.37	0.088	0.133	1
GO_CYCLIN_DEPENDENT_PROTEIN_SERINE_THREONINE_KINASE_REGULATOR_ACTIVITY	27	0.39	1.37	0.074	0.135	1
GO_NEGATIVE_REGULATION_OF_GENE_SILENCING	19	0.43	1.37	0.112	0.135	1
GO_POLY_PURINE_TRACT_BINDING	19	0.43	1.37	0.097	0.135	1
GO_POSITIVE_REGULATION_OF_STRESS_ACTIVATED_PROTEIN_KINASE_SIGNALING_CASCADE	135	0.28	1.37	0.023	0.136	1
GO_DNA_MODIFICATION	79	0.31	1.37	0.028	0.136	1

GO_REGULATION_OF_PROTEIN_OLIGOMERIZATION	34	0.37	1.37	0.079	0.137	1
GO_TERPENOID_METABOLIC_PROCESS	105	0.3	1.36	0.048	0.137	1
GO_GLUTATHIONE_METABOLIC_PROCESS	57	0.32	1.36	0.043	0.137	1
GO_MEMBRANE_PROTEIN_ECTODOMAIN_PROTEOLYSIS	22	0.41	1.36	0.097	0.138	1
GO_FORMATION_OF_PRIMARY_GERM_LAYER	110	0.29	1.36	0.032	0.138	1
GO_NEGATIVE_REGULATION_OF_PROTEIN_CATABOLIC_PROCESS	108	0.29	1.36	0.035	0.138	1
GO_POSITIVE_REGULATION_OF_CELL_CYCLE_G1_S_PHASE_TRANSITION	29	0.39	1.36	0.072	0.138	1
GO_CELLULAR_KETONE_METABOLIC_PROCESS	67	0.32	1.36	0.035	0.138	1
GO_VITAMIN_METABOLIC_PROCESS	119	0.29	1.36	0.03	0.138	1
GO_REGULATION_OF_SUPEROXIDE_METABOLIC_PROCESS	21	0.42	1.36	0.1	0.138	1
GO_PYRIMIDINE_CONTAINING_COMPOUND_METABOLIC_PROCESS	75	0.32	1.36	0.044	0.139	1
GO_CELLULAR_RESPONSE_TO_OXYGEN_LEVELS	142	0.28	1.36	0.02	0.139	1
GO_OXIDOREDUCTASE_ACTIVITY_ACTING_ON_THE_ALDEHYDE_OR_OXO_GROUP_OF_DONORS_NAD_OR_NADP_AS_ACCEPTOR	36	0.36	1.36	0.071	0.139	1
GO_RESPONSE_TO_TEMPERATURE_STIMULUS	148	0.28	1.36	0.022	0.139	1
GO_RESPONSE_TO_COPPER_ION	28	0.38	1.36	0.078	0.139	1
GO_CELL_CELL_JUNCTION	376	0.25	1.36	0	0.139	1
GO_POSITIVE_REGULATION_OF_LEUKOCYTE_APOPTOTIC_PROCESS	26	0.39	1.36	0.075	0.139	1
GO_PHAGOCYTOSIS	158	0.27	1.36	0.011	0.139	1
GO_REGULATION_OF_TYPE_2_IMMUNE_RESPONSE	26	0.39	1.36	0.09	0.139	1
GO_GLUCURONOSYLTRANSFERASE_ACTIVITY	33	0.38	1.36	0.068	0.139	1
GO_G_PROTEIN_COUPLED_CHEMOATTRACTANT_RECEPTOR_ACTIVITY	25	0.39	1.36	0.085	0.14	1
GO_CARTILAGE_DEVELOPMENT_INVOLVED_IN_ENDOCHONDRAL_BONE_MORPHOGENESIS	19	0.43	1.36	0.087	0.14	1
GO_CELL_FATE_COMMITMENT_INVOLVED_IN_FORMATION_OF_PRIMARY_GERM_LAYER	28	0.39	1.36	0.086	0.14	1
GO_OXIDOREDUCTASE_ACTIVITY_ACTING_ON_PAIRED_DONORS_WITH_INCORPORATION_OR_REDUCTION_OF_MOLECULAR_OXYGEN_2_OXOGLUTARATE_AS_ONE_DONOR_AND_INCORPORATION_OF_ONE_ATOM_EACH_OF_OXYGEN_INTO_BOTH_DONORS	43	0.35	1.36	0.07	0.14	1
GO_RNA_DEPENDENT_DNA_BIOSYNTHETIC_PROCESS	21	0.41	1.36	0.11	0.141	1
GO_DOPAMINERGIC_NEURON_DIFFERENTIATION	28	0.39	1.36	0.091	0.141	1
GO_EMBRYONIC_SKELETAL_SYSTEM_MORPHOGENESIS	93	0.3	1.36	0.024	0.141	1
GO_NUCLEAR_BODY	332	0.25	1.36	0.006	0.141	1
GO_PROTEIN_DEPOLYMERIZATION	25	0.41	1.36	0.096	0.141	1
GO_POSITIVE_REGULATION_OF_PROTEIN_SECRETION	207	0.26	1.36	0.008	0.141	1
GO_CYCLIN_DEPENDENT_PROTEIN_KINASE_ACTIVITY	33	0.36	1.36	0.08	0.142	1
GO_PEPTIDYL_TYROSINE_AUTOPHOSPHORYLATION	39	0.35	1.36	0.073	0.141	1
GO_HEXOSE_METABOLIC_PROCESS	156	0.28	1.36	0.021	0.141	1
GO_REGULATION_OF_FATTY_ACID_TRANSPORT	26	0.39	1.36	0.094	0.142	1
GO_CELLULAR_RESPONSE_TO_DEXAMETHASONE_STIMULUS	27	0.39	1.36	0.107	0.142	1
GO_MORPHOGENESIS_OF_AN_EPITHELIUM	398	0.25	1.35	0	0.142	1
GO_PEPTIDYL_SERINE_MODIFICATION	146	0.27	1.35	0.026	0.143	1
GO_INTRINSIC_APOPTOTIC_SIGNALING_PATHWAY_IN_RESPONSE_TO_ENDOPLASMIC_RETICULUM_STRESS	31	0.38	1.35	0.098	0.144	1
GO_OSTEOBLAST_DIFFERENTIATION	124	0.28	1.35	0.036	0.144	1
GO_ORGANOPHOSPHATE_BIOSYNTHETIC_PROCESS	442	0.24	1.35	0.003	0.146	1
GO_COPI_COATED_VESICLE_MEMBRANE	17	0.45	1.35	0.096	0.146	1
GO_MALE_MEIOSIS	38	0.36	1.35	0.091	0.147	1
GO_CELLULAR_RESPONSE_TO_ALCOHOL	115	0.29	1.35	0.043	0.147	1
GO_TRNA_METHYLTRANSFERASE_ACTIVITY	19	0.43	1.35	0.103	0.147	1
GO_UBIQUITIN_LIKE_PROTEIN_LIGASE_BINDING	261	0.25	1.35	0.003	0.147	1
GO_SERINE_FAMILY_AMINO_ACID_METABOLIC_PROCESS	41	0.35	1.35	0.094	0.147	1
GO_DEOXYRIBOSE_PHOSPHATE_CATABOLIC_PROCESS	21	0.42	1.35	0.103	0.147	1
GO_REGULATION_OF_STEROID_BIOSYNTHETIC_PROCESS	48	0.34	1.35	0.082	0.148	1
GO_NEGATIVE_REGULATION_OF_HYDROLASE_ACTIVITY	382	0.24	1.35	0	0.148	1
GO_RESPONSE_TO_INTERLEUKIN_4	31	0.37	1.35	0.093	0.148	1
GO_RESPONSE_TO_ESTRADIOL	146	0.27	1.35	0.028	0.149	1
GO_CELLULAR_RESPONSE_TO_EXTRACELLULAR_STIMULUS	186	0.26	1.34	0.023	0.149	1
GO_NUCLEOSIDE_DIPHOSPHATE_METABOLIC_PROCESS	80	0.3	1.34	0.04	0.149	1
GO_MYOFILAMENT	24	0.4	1.34	0.102	0.15	1
GO_CELLULAR_HORMONE_METABOLIC_PROCESS	104	0.29	1.34	0.035	0.15	1
GO_POSITIVE_REGULATION_OF_RESPONSE_TO_BIOTIC_STIMULUS	38	0.35	1.34	0.083	0.15	1
GO_REGULATION_OF_B_CELL_APOPTOTIC_PROCESS	18	0.43	1.34	0.095	0.151	1
GO_REGULATION_OF_SEQUENCE_SPECIFIC_DNA_BINDING_TRANSCRIPTION_FACTOR_ACTIVITY	358	0.24	1.34	0.003	0.151	1
GO_COMPLEX_OF_COLLAGEN_TRIMERS	23	0.4	1.34	0.111	0.152	1
GO_ENDODERM_DEVELOPMENT	70	0.31	1.34	0.048	0.153	1
GO_REGULATION_OF_MEIOTIC_CELL_CYCLE	40	0.34	1.34	0.091	0.153	1
GO_INNER_MITOCHONDRIAL_MEMBRANE_ORGANIZATION	17	0.44	1.34	0.096	0.153	1
GO_WATER_SOLUBLE_VITAMIN_METABOLIC_PROCESS	87	0.3	1.34	0.039	0.154	1
GO_COA_HYDROLASE_ACTIVITY	21	0.41	1.34	0.114	0.154	1
GO_POSITIVE_REGULATION_OF_RESPONSE_TO_CYTOKINE_STIMULUS	33	0.36	1.34	0.091	0.154	1
GO_CHEMOKINE_BINDING	21	0.41	1.34	0.109	0.155	1
GO_NEGATIVE_REGULATION_OF_CYTOKINE_PRODUCTION_INVOLVED_IN_IMMUNE_RESPONSE	22	0.4	1.33	0.111	0.157	1
GO_CELLULAR_RESPONSE_TO_FATTY_ACID	51	0.33	1.33	0.075	0.157	1
GO_PODOSOME	23	0.4	1.33	0.114	0.158	1
GO_PROTEIN_AUTOPHOSPHORYLATION	191	0.26	1.33	0.028	0.158	1
GO_EXTRACELLULAR_MATRIX_BINDING	51	0.33	1.33	0.084	0.158	1
GO_RESPONSE_TO_DRUG	428	0.24	1.33	0	0.159	1
GO_LYSOPHOSPHOLIPID_ACYLTRANSFERASE_ACTIVITY	19	0.41	1.33	0.101	0.159	1
GO_MORPHOGENESIS_OF_A_BRANCHING_STRUCTURE	167	0.26	1.33	0.026	0.159	1
GO_REGULATION_OF_INTERFERON_GAMMA_BIOSYNTHETIC_PROCESS	16	0.44	1.33	0.115	0.161	1
GO_RNA_POLYMERASE_II_CARBOXY_TERMINAL_DOMAIN_KINASE_ACTIVI	16	0.43	1.33	0.12	0.161	1
GO_TRANSITION_METAL_ION_TRANSMEMBRANE_TRANSPORTER_ACTIVI	39	0.35	1.33	0.088	0.163	1
GO_CELLULAR_RESPONSE_TO_OXIDATIVE_STRESS	184	0.26	1.33	0.029	0.163	1

GO_NLS_BEARING_PROTEIN_IMPORT_INTO_NUCLEUS	22	0.41	1.33	0.111	0.164	1
GO_REGULATION_OF_LAMELLIPODIUM_ASSEMBLY	26	0.38	1.33	0.127	0.164	1
GO_REGULATION_OF_RUFFLE_ASSEMBLY	20	0.41	1.32	0.112	0.164	1
GO_RESPONSE_TO_EXTRACELLULAR_STIMULUS	438	0.24	1.32	0.003	0.165	1
GO_WNT_SIGNALING_PATHWAY	350	0.24	1.32	0.003	0.165	1
GO_REGULATION_OF_ANOIKIS	24	0.39	1.32	0.113	0.165	1
GO_INTRINSIC_APOPTOTIC_SIGNALING_PATHWAY	149	0.27	1.32	0.026	0.167	1
GO_BLOOD_MICROPARTICLE	114	0.28	1.32	0.049	0.168	1
GO_MACROMOLECULE_METHYLATION	191	0.26	1.32	0.019	0.167	1
GO_POSITIVE_REGULATION_OF_CYTOKINE_PRODUCTION_INVOLVED_IN_IMMUNE_RESPONSE	30	0.38	1.32	0.118	0.168	1
GO_MOTOR_ACTIVITY	129	0.27	1.32	0.027	0.168	1
GO_REGULATION_OF_CHEMOTAXIS	179	0.26	1.32	0.018	0.168	1
GO_ACYLGLYCEROL_O_ACYLTRANSFERASE_ACTIVITY	28	0.37	1.32	0.122	0.168	1
GO_ALPHA_AMINO_ACID_METABOLIC_PROCESS	226	0.25	1.32	0.008	0.168	1
GO_NEGATIVE_REGULATION_OF_INFLAMMATORY_RESPONSE	97	0.29	1.32	0.053	0.168	1
GO_EMBRYONIC_SKELETAL_SYSTEM_DEVELOPMENT	122	0.28	1.32	0.041	0.168	1
GO_SIGNAL_TRANSDUCTION_IN_ABSENCE_OF_LIGAND	33	0.37	1.32	0.108	0.17	1
GO_TOXIN_TRANSPORT	36	0.35	1.32	0.102	0.17	1
GO_ESTABLISHMENT_OR_MAINTENANCE_OF_CELL_POLARITY	140	0.27	1.32	0.021	0.17	1
GO_IMMUNOGLOBULIN_BINDING	23	0.39	1.32	0.121	0.172	1
GO_MULTI_MULTICELLULAR_ORGANISM_PROCESS	210	0.26	1.32	0.032	0.172	1
GO_POSITIVE_REGULATION_OF_OXIDOREDUCTASE_ACTIVITY	46	0.33	1.31	0.085	0.173	1
GO_V_D_J_RECOMBINATION	16	0.43	1.31	0.136	0.174	1
GO_LYMPHOCYTE_DIFFERENTIATION	209	0.25	1.31	0.025	0.174	1
GO_CELLULAR_RESPONSE_TO_LIGHT_STIMULUS	89	0.29	1.31	0.079	0.175	1
GO_CALCIUM_INDEPENDENT_CELL_CELL_ADHESION_VIA_PLASMA_MEMBRANE_CELL_ADHESION_MOLECULES	21	0.39	1.31	0.139	0.175	1
GO_AMELOGENESIS	20	0.41	1.31	0.157	0.175	1
GO_MHC_CLASS_II_PROTEIN_COMPLEX_BINDING	16	0.44	1.31	0.122	0.175	1
GO_AROMATIC_AMINO_ACID_FAMILY_CATABOLIC_PROCESS	20	0.42	1.31	0.14	0.175	1
GO_UDP_GLYCOSYLTRANSFERASE_ACTIVITY	136	0.27	1.31	0.027	0.175	1
GO_B_CELL_ACTIVATION	131	0.27	1.31	0.04	0.175	1
GO_MITOCHONDRIAL_GENOME_MAINTENANCE	24	0.39	1.31	0.118	0.176	1
GO_ER_TO_GOLGI_TRANSPORT_VESICLE_MEMBRANE	53	0.32	1.31	0.09	0.176	1
GO_COFACTOR_BIOSYNTHETIC_PROCESS	162	0.26	1.31	0.033	0.176	1
GO_TRANSFERASE_ACTIVITY_TRANSFERRING_ONE_CARBON_GROUPS	203	0.25	1.31	0.033	0.176	1
GO_PROTEIN_HOMOTRIMERIZATION	19	0.41	1.31	0.131	0.178	1
GO_TRANSCRIPTION_FROM_RNA_POLYMERASE_III_PROMOTER	40	0.34	1.31	0.111	0.179	1
GO_NEGATIVE_REGULATION_OF_B_CELL_ACTIVATION	30	0.36	1.31	0.119	0.18	1
GO_REGULATION_OF_WNT_SIGNALING_PATHWAY	306	0.24	1.3	0.012	0.18	1
GO_PLACENTA_BLOOD_VESSEL_DEVELOPMENT	28	0.38	1.3	0.125	0.18	1
GO_REGULATION_OF_MITOCHONDRION_ORGANIZATION	214	0.25	1.3	0.028	0.18	1
GO_OXIDOREDUCTASE_ACTIVITY_ACTING_ON_PAIRED_DONORS_WITH_INCORPORATION_OR_REDUCTION_OF_MOLECULAR_OXYGEN_REDUCED_FLAVIN_OR_FLAVOPROTEIN_AS_ONE_DONOR_AND_INCORPORATION_OF_ONE_ATOM_OF_OXYGEN	26	0.38	1.3	0.115	0.18	1
GO_LUNG_MORPHOGENESIS	45	0.34	1.3	0.095	0.181	1
GO_POSITIVE_REGULATION_OF_MONOOXYGENASE_ACTIVITY	28	0.37	1.3	0.108	0.182	1
GO_PROTEIN_EXIT_FROM_ENDOPLASMIC_RETICULUM	20	0.41	1.3	0.105	0.183	1
GO_CELLULAR_RESPONSE_TO_NUTRIENT	39	0.34	1.3	0.095	0.183	1
GO_TRANSCRIPTION_FACTOR_COMPLEX	296	0.24	1.3	0.018	0.184	1
GO_OVARIAN_FOLLICLE_DEVELOPMENT	61	0.31	1.3	0.096	0.184	1
GO_SERINE_FAMILY_AMINO_ACID_BIOSYNTHETIC_PROCESS	15	0.42	1.3	0.17	0.184	1
GO_RESPONSE_TO_TOXIC_SUBSTANCE	239	0.25	1.3	0.019	0.185	1
GO_EPIDERMIS_MORPHOGENESIS	29	0.36	1.3	0.127	0.185	1
GO_COVALENT_CHROMATIN_MODIFICATION	336	0.24	1.3	0.006	0.185	1
GO_LIPOPOLYSACCHARIDE_BINDING	18	0.41	1.3	0.13	0.185	1
GO_LEUKOTRIENE_METABOLIC_PROCESS	30	0.36	1.3	0.124	0.186	1
GO_PURINE_NUCLEOSIDE_BIOSYNTHETIC_PROCESS	87	0.29	1.3	0.062	0.186	1
GO_PTERIDINE_CONTAINING_COMPOUND_BIOSYNTHETIC_PROCESS	17	0.42	1.3	0.164	0.187	1
GO_NEGATIVE_REGULATION_OF_ALPHA_BETA_T_CELL_DIFFERENTIATIO	15	0.44	1.3	0.144	0.188	1
GO_TRANSLATION_ELONGATION_FACTOR_ACTIVITY	19	0.41	1.3	0.13	0.188	1
GO_NEGATIVE_REGULATION_OF_INTERLEUKIN_8_PRODUCTION	15	0.43	1.29	0.173	0.189	1
GO_POSITIVE_REGULATION_OF_INTRINSIC_APOPTOTIC_SIGNALING_PATHWAY	51	0.32	1.29	0.084	0.19	1
GO_TUBULIN_BINDING	263	0.24	1.29	0.024	0.191	1
GO_PYRIMIDINE_RIBONUCLEOSIDE_TRIPHOSPHATE_METABOLIC_PROCE	16	0.43	1.29	0.151	0.191	1
GO_QUINONE_BINDING	17	0.43	1.29	0.147	0.191	1
GO_REGULATION_OF_ERK1_AND_ERK2_CASCADE	236	0.24	1.29	0.023	0.192	1
GO_EPITHELIAL_TUBE_BRANCHING_INVOLVED_IN_LUNG_MORPHOGENE	25	0.38	1.29	0.119	0.192	1
GO_CELLULAR_RESPONSE_TO_GONADOTROPIN_STIMULUS	15	0.43	1.29	0.168	0.192	1
GO_CARBOHYDRATE_PHOSPHORYLATION	22	0.39	1.29	0.148	0.193	1
GO_FORMATION_OF_TRANSLATION_PREINITIATION_COMPLEX	20	0.4	1.29	0.137	0.194	1
GO_HEME_BIOSYNTHETIC_PROCESS	19	0.41	1.29	0.129	0.194	1
GO_NEUROBLAST_PROLIFERATION	29	0.36	1.29	0.142	0.194	1
GO_B_CELL_ACTIVATION_INVOLVED_IN_IMMUNE_RESPONSE	35	0.35	1.29	0.12	0.194	1
GO_REGULATION_OF_MONOOXYGENASE_ACTIVITY	59	0.31	1.29	0.085	0.195	1
GO_PHOSPHATIDYLETHANOLAMINE_ACYL_CHAIN_REMODELING	22	0.39	1.29	0.141	0.196	1
GO_PROTEIN_BINDING_INVOLVED_IN_CELL_ADHESION	17	0.42	1.29	0.139	0.196	1
GO_CARBOHYDRATE_DERIVATIVE_TRANSPORTER_ACTIVITY	33	0.35	1.29	0.124	0.197	1
GO_ER_TO_GOLGI_TRANSPORT_VESICLE	69	0.3	1.28	0.083	0.198	1
GO_OXIDOREDUCTASE_ACTIVITY_ACTING_ON_NAD_P_H_OXYGEN_AS_ACCEPTOR	16	0.43	1.28	0.17	0.198	1
GO_HSP90_PROTEIN_BINDING	27	0.37	1.28	0.14	0.198	1
GO_REGULATION_OF_DNA_TEMPLATED_TRANSCRIPTION_IN_RESPONSE_TO_STRESS	67	0.3	1.28	0.079	0.199	1

GO_RESPONSE_TO_KETONE	181	0.25	1.28	0.028	0.199	1
GO_REGULATION_OF_TUMOR_NECROSIS_FACTOR_BIOSYNTHETIC_PRO	18	0.41	1.28	0.153	0.2	1
GO_NEGATIVE_REGULATION_OF_PROTEIN_BINDING	79	0.29	1.28	0.083	0.2	1
GO_PEPTIDYL_ARGININE_MODIFICATION	19	0.4	1.28	0.131	0.2	1
GO_REGULATION_OF_ALCOHOL_BIOSYNTHETIC_PROCESS	45	0.32	1.28	0.118	0.201	1
GO_NUCLEAR_LOCALIZATION_SEQUENCE_BINDING	21	0.39	1.28	0.141	0.201	1
GO_REGULATION_OF_MEMBRANE_PROTEIN_ECTODOMAIN_PROTEOLYSI	21	0.39	1.28	0.139	0.201	1
GO_DNA_DEALKYLATION	20	0.4	1.28	0.154	0.201	1
GO_REGULATION_OF_MAST_CELL_ACTIVATION_INVOLVED_IN_IMMUNE_RESPONSE	31	0.35	1.28	0.118	0.201	1
GO_OXIDOREDUCTASE_ACTIVITY_ACTING_ON_PAIRED_DONORS_WITH_INCORPORATION_OR_REDUCTION_OF_MOLECULAR_OXYGEN	149	0.26	1.28	0.052	0.202	1
GO_REGULATION_OF_BONE_DEVELOPMENT	17	0.42	1.28	0.148	0.203	1
GO_OXIDOREDUCTASE_ACTIVITY_ACTING_ON_THE_ALDEHYDE_OR_OXO_GROUP_OF_DONORS	46	0.32	1.28	0.105	0.205	1
GO_RESPONSE_TO_FOOD	19	0.41	1.28	0.173	0.205	1
GO_RESPONSE_TO_VITAMIN_A	20	0.4	1.28	0.167	0.205	1
GO_FOREBRAIN_REGIONALIZATION	25	0.37	1.27	0.152	0.207	1
GO_MICROTUBULE_ORGANIZING_CENTER_PART	131	0.27	1.27	0.059	0.208	1
GO_REGULATION_OF_NUCLEOCYTOPLASMIC_TRANSPORT	219	0.25	1.27	0.03	0.209	1
GO_RNA_POLYMERASE_II_TRANSCRIPTION_FACTOR_COMPLEX	100	0.28	1.27	0.065	0.209	1
GO_PHOSPHOLIPASE_A2_ACTIVITY	30	0.35	1.27	0.149	0.21	1
GO_MICROVILLUS_ORGANIZATION	20	0.39	1.27	0.143	0.21	1
GO_DNA_N_GLYCOSYLASE_ACTIVITY	15	0.43	1.27	0.176	0.21	1
GO_OVULATION	18	0.4	1.27	0.153	0.211	1
GO_RIBONUCLEOSIDE_DIPHOSPHATE_METABOLIC_PROCESS	62	0.3	1.27	0.125	0.211	1
GO_NEGATIVE_REGULATION_OF_CYTOKINE_BIOSYNTHETIC_PROCESS	28	0.37	1.27	0.129	0.212	1
GO_NEGATIVE_REGULATION_OF_T_CELL_APOPTOTIC_PROCESS	16	0.42	1.27	0.164	0.213	1
GO_RESPONSE_TO_INORGANIC_SUBSTANCE	479	0.22	1.27	0.003	0.213	1
GO_NEGATIVE_REGULATION_OF_INTRINSIC_APOPTOTIC_SIGNALING_PATHWAY	90	0.28	1.27	0.084	0.213	1
GO_INTEGRIN_MEDIATED_SIGNALING_PATHWAY	82	0.28	1.27	0.073	0.213	1
GO_LYMPHOCYTE_ACTIVATION_INVOLVED_IN_IMMUNE_RESPONSE	97	0.28	1.27	0.1	0.215	1
GO_RIBONUCLEOSIDE_TRIPHOSPHATE_BIOSYNTHETIC_PROCESS	51	0.31	1.27	0.123	0.215	1
GO_DEVELOPMENT_OF_PRIMARY_SEXUAL_CHARACTERISTICS	214	0.24	1.26	0.027	0.215	1
GO_DEFENSE_RESPONSE_TO_FUNGUS	36	0.34	1.26	0.136	0.216	1
GO_NEGATIVE_REGULATION_OF_RESPONSE_TO_DNA_DAMAGE_STIMUL	51	0.31	1.26	0.102	0.216	1
GO_INTRINSIC_COMPONENT_OF_MITOCHONDRIAL_INNER_MEMBRANE	20	0.4	1.26	0.158	0.216	1
GO_PRE_MRNA_BINDING	22	0.38	1.26	0.143	0.216	1
GO_RETINOL_METABOLIC_PROCESS	29	0.35	1.26	0.132	0.216	1
GO_DIGESTION	132	0.26	1.26	0.07	0.217	1
GO_PYRIMIDINE_RIBONUCLEOTIDE_METABOLIC_PROCESS	24	0.37	1.26	0.155	0.217	1
GO_PHAGOCYTIC_VESICLE_MEMBRANE	57	0.31	1.26	0.1	0.219	1
GO_CYTOKINE_BINDING	91	0.28	1.26	0.079	0.219	1
GO_REGULATION_OF_PROTEIN_MATURATION	80	0.28	1.26	0.102	0.219	1
GO_METAL_CLUSTER_BINDING	62	0.3	1.26	0.11	0.22	1
GO_NUCLEAR_EUCHROMATIN	24	0.36	1.26	0.157	0.22	1
GO_N_TERMINAL_PROTEIN_AMINO_ACID_MODIFICATION	24	0.37	1.26	0.184	0.22	1
GO_GRANULOCYTE_ACTIVATION	21	0.39	1.26	0.166	0.221	1
GO_ORGANOPHOSPHATE_ESTER_TRANSMEMBRANE_TRANSPORTER_ACTIVITY	23	0.37	1.26	0.163	0.224	1
GO_REGULATION_OF_WOUND_HEALING	125	0.26	1.26	0.063	0.224	1
GO_PML_BODY	93	0.27	1.26	0.073	0.225	1
GO_CELL_SUBSTRATE_JUNCTION_ASSEMBLY	41	0.33	1.26	0.136	0.225	1
GO_NEURAL_TUBE_FORMATION	93	0.27	1.25	0.076	0.227	1
GO_EPIDERMAL_GROWTH_FACTOR_RECEPTOR_SIGNALING_PATHWAY	55	0.3	1.25	0.114	0.227	1
GO_CORTICAL_CYTOSKELETON_ORGANIZATION	36	0.34	1.25	0.152	0.227	1
GO_TISSUE_REMODELING	87	0.28	1.25	0.076	0.228	1
GO_NEGATIVE_REGULATION_OF_PROTEIN_TYROSINE_KINASE_ACTIVITY	20	0.4	1.25	0.164	0.228	1
GO_ROUGH_ENDOPLASMIC_RETICULUM_MEMBRANE	21	0.38	1.25	0.151	0.228	1
GO_GLYCOLIPID_BINDING	19	0.41	1.25	0.176	0.229	1
GO_REGULATION_OF_STRESS_ACTIVATED_PROTEIN_KINASE_SIGNALING_CASCADE	197	0.24	1.25	0.045	0.229	1
GO_MULTICELLULAR_ORGANISM_AGING	31	0.34	1.25	0.151	0.229	1
GO_REGULATION_OF_REACTIVE_OXYGEN_SPECIES_METABOLIC_PROCE	148	0.26	1.25	0.058	0.229	1
GO_REGULATION_OF_ESTABLISHMENT_OF_PROTEIN_LOCALIZATION_TO_MITOCHONDRION	126	0.26	1.25	0.082	0.229	1
GO_PROTEIN_LOCALIZATION_TO_CENTROSOME	17	0.41	1.25	0.205	0.229	1
GO_POLY_PYRIMIDINE_TRACT_BINDING	17	0.41	1.25	0.195	0.23	1
GO_COMPLEMENT_ACTIVATION	45	0.31	1.25	0.139	0.23	1
GO_COMPLEMENT_BINDING	18	0.4	1.25	0.194	0.23	1
GO_NEGATIVE_REGULATION_OF_TRANSLATIONAL_INITIATION	21	0.39	1.25	0.142	0.23	1
GO_CORE_PROMOTER_BINDING	152	0.25	1.25	0.043	0.231	1
GO_TRANSCRIPTIONAL_REPRESSOR_ACTIVITY_RNA_POLYMERASE_II_TRANSCRIPTION_REGULATORY_REGION_SEQUENCE_SPECIFIC_BINDING	167	0.25	1.25	0.05	0.231	1
GO_HISTONE_METHYLTRANSFERASE_COMPLEX	70	0.29	1.25	0.13	0.232	1
GO_NEURAL_PRECURSOR_CELL_PROLIFERATION	69	0.29	1.25	0.108	0.232	1
GO_NUCLEAR_TRANSCRIPTION_FACTOR_COMPLEX	126	0.26	1.25	0.079	0.232	1
GO_TRANSCRIPTION_FACTOR_ACTIVITY_RNA_POLYMERASE_II_CORE_PROMOTER_PROXIMAL_REGION_SEQUENCE_SPECIFIC_BINDING	328	0.23	1.25	0.02	0.234	1
GO_EXTRINSIC_APOPTOTIC_SIGNALING_PATHWAY_VIA_DEATH_DOMAIN_RECEPTORS	39	0.32	1.25	0.145	0.234	1
GO_REGULATION_OF_REPRODUCTIVE_PROCESS	129	0.26	1.25	0.083	0.234	1
GO_HYDROLASE_ACTIVITY_ACTING_ON_CARBON_NITROGEN_BUT_NOT_PEPTIDE_BONDS	143	0.25	1.25	0.059	0.234	1
GO_PROTEIN_PHOSPHATASE_BINDING	117	0.27	1.24	0.059	0.234	1
GO_REGULATION_OF_EXOSOMAL_SECRETION	16	0.41	1.24	0.167	0.234	1

GO_FRUCTOSE_METABOLIC_PROCESS	15	0.43	1.24	0.167	0.235	1
GO_NUCLEOSIDE_SALVAGE	16	0.41	1.24	0.184	0.235	1
GO_NEGATIVE_REGULATION_OF_WOUND_HEALING	58	0.3	1.24	0.106	0.236	1
GO_POSITIVE_REGULATION_OF_G1_S_TRANSITION_OF_MITOTIC_CELL_	24	0.37	1.24	0.195	0.238	1
GO_SINGLE_STRANDED_RNA_BINDING	67	0.29	1.24	0.131	0.24	1
GO_GLYCOSYLATION	259	0.23	1.24	0.026	0.24	1
GO_RELEASE_OF_CYTOCHROME_C_FROM_MITOCHONDRIA	22	0.37	1.24	0.167	0.24	1
GO_NEGATIVE_REGULATION_OF_LEUKOCYTE_DIFFERENTIATION	82	0.28	1.24	0.103	0.24	1
GO_CELL_DEATH_IN_RESPONSE_TO_OXIDATIVE_STRESS	18	0.4	1.24	0.178	0.24	1
GO_RECEPTOR_AGONIST_ACTIVITY	16	0.41	1.24	0.187	0.241	1
GO_ANDROGEN_METABOLIC_PROCESS	30	0.34	1.24	0.173	0.241	1
GO_RESPONSE_TO_ARSENIC_CONTAINING_SUBSTANCE	29	0.34	1.24	0.161	0.241	1
GO_NITROGEN_COMPOUND_TRANSPORT	487	0.22	1.24	0.013	0.241	1
GO_PHOSPHATIDYLCHOLINE_ACYL_CHAIN_REMODELING	25	0.37	1.24	0.169	0.242	1
GO_SH2_DOMAIN_BINDING	29	0.35	1.24	0.17	0.243	1
GO_NATURAL_KILLER_CELL_ACTIVATION	52	0.31	1.24	0.116	0.243	1
GO_POSITIVE_REGULATION_OF_ANTIGEN_RECEPTOR_MEDIATED_SIGNALING_PATHWAY	15	0.42	1.24	0.174	0.244	1
GO_ALDITOL_PHOSPHATE_METABOLIC_PROCESS	34	0.33	1.24	0.146	0.244	1
GO_EPIDERMAL_GROWTH_FACTOR_RECEPTOR_BINDING	30	0.35	1.23	0.16	0.244	1
GO_REGULATION_OF_CELLULAR_SENESCENCE	26	0.36	1.23	0.167	0.247	1
GO_STEM_CELL_PROLIFERATION	59	0.3	1.23	0.148	0.249	1
GO_PHOSPHATIDYLINOSITOL_ACYL_CHAIN_REMODELING	16	0.41	1.23	0.192	0.249	1
GO_REGULATION_OF_MORPHOGENESIS_OF_A_BRANCHING_STRUCTUR	53	0.3	1.23	0.121	0.249	1
GO_REGULATION_OF_VIRAL_INDUCED_CYTOPLASMIC_PATTERN_RECOGNITION_RECEPTOR_SIGNALING_PATHWAY	15	0.42	1.23	0.193	0.25	1
GO_ORGANELLE_LOCALIZATION	403	0.22	1.23	0.029	0.25	1
GO_4_IRON_4_SULFUR_CLUSTER_BINDING	41	0.32	1.23	0.161	0.25	1
GO_NEGATIVE_REGULATION_OF_DNA_METABOLIC_PROCESS	108	0.26	1.23	0.082	0.251	1
GO_NUCLEOBASE_CONTAINING_COMPOUND_TRANSMEMBRANE_TRANSPORTER_ACTIVITY	31	0.35	1.23	0.182	0.251	1
GO_CYTOPLASMIC_MICROTUBULE	56	0.29	1.23	0.135	0.252	1
GO_BROWN_FAT_CELL_DIFFERENTIATION	30	0.34	1.23	0.172	0.252	1
GO_RESPONSE_TO_ESTROGEN	217	0.24	1.23	0.054	0.252	1
GO_ANTIGEN_PROCESSING_AND_PRESENTATION_OF_PEPTIDE_OR_POLYSACCHARIDE_ANTIGEN_VIA_MHC_CLASS_II	90	0.27	1.23	0.103	0.252	1
GO_INTRACELLULAR_RECEPTOR_SIGNALING_PATHWAY	165	0.24	1.23	0.075	0.253	1
GO_DEFENSE_RESPONSE_TO_GRAM_NEGATIVE_BACTERIUM	41	0.32	1.23	0.147	0.253	1
GO_MYD88_DEPENDENT_TOLL_LIKE_RECEPTOR_SIGNALING_PATHWAY	32	0.34	1.23	0.176	0.254	1
GO_REGULATION_OF_MAST_CELL_ACTIVATION	38	0.32	1.23	0.158	0.254	1
GO_SOMATIC_STEM_CELL_DIVISION	22	0.37	1.23	0.177	0.254	1
GO_APOPTOTIC_MITOCHONDRIAL_CHANGES	57	0.3	1.23	0.129	0.254	1
GO_POSITIVE_REGULATION_OF_CYTOPLASMIC_TRANSPORT	280	0.23	1.23	0.058	0.254	1
GO_PROTEIN_OLIGOMERIZATION	425	0.22	1.23	0.01	0.254	1
GO_RESPONSE_TO_STEROID_HORMONE	492	0.22	1.22	0.023	0.255	1
GO_POSITIVE_REGULATION_OF_ORGANELLE_ASSEMBLY	45	0.31	1.22	0.168	0.255	1
GO_PROTEIN_COMPLEX_INVOLVED_IN_CELL_ADHESION	30	0.35	1.22	0.184	0.255	1
GO_RESPIRATORY_CHAIN_COMPLEX_IV_ASSEMBLY	17	0.4	1.22	0.222	0.255	1
GO_PYRIMIDINE_DEOXYRIBONUCLEOTIDE_METABOLIC_PROCESS	16	0.4	1.22	0.194	0.255	1
GO_NEGATIVE_REGULATION_OF_LIPID_STORAGE	17	0.4	1.22	0.174	0.256	1
GO_REGULATION_OF_ODONTOGENESIS	24	0.36	1.22	0.161	0.256	1
GO_CELLULAR_RESPONSE_TO_VITAMIN	26	0.35	1.22	0.175	0.256	1
GO_NUCLEAR_INNER_MEMBRANE	54	0.29	1.22	0.137	0.256	1
GO_DEAMINASE_ACTIVITY	33	0.33	1.22	0.183	0.256	1
GO_CELLULAR_RESPONSE_TO_DRUG	65	0.29	1.22	0.111	0.256	1
GO_PROTEIN_K11_LINKED_UBIQUITINATION	27	0.35	1.22	0.183	0.257	1
GO_POSITIVE_REGULATION_OF_TELOMERE_CAPPING	16	0.4	1.22	0.189	0.257	1
GO_GLYCOSIDE_METABOLIC_PROCESS	16	0.41	1.22	0.19	0.258	1
GO_BRANCHING_MORPHOGENESIS_OF_AN_EPITHELIAL_TUBE	131	0.25	1.22	0.083	0.258	1
GO_REGULATION_OF_INTERLEUKIN_4_PRODUCTION	29	0.35	1.22	0.176	0.258	1
GO_APOPTOTIC_CELL_CLEARANCE	27	0.35	1.22	0.18	0.259	1
GO_HSP70_PROTEIN_BINDING	28	0.35	1.22	0.195	0.262	1
GO_MICROTUBULE_PLUS_END	17	0.39	1.22	0.216	0.262	1
GO_RESPONSE_TO_RETINOIC_ACID	106	0.26	1.22	0.108	0.263	1
GO_MORPHOGENESIS_OF_EMBRYONIC_EPITHELIUM	133	0.25	1.22	0.106	0.263	1
GO_DNA_DOUBLE_STRAND_BREAK_PROCESSING	17	0.39	1.22	0.21	0.263	1
GO_LAMELLAR_BODY	20	0.39	1.22	0.195	0.263	1
GO_POSITIVE_REGULATION_OF_MITOCHONDRION_ORGANIZATION	163	0.24	1.22	0.099	0.264	1
GO_POSITIVE_REGULATION_OF_REACTIVE_OXYGEN_SPECIES_METABOLIC_PROCESS	84	0.27	1.22	0.109	0.264	1
GO_BONE_RESORPTION	21	0.37	1.22	0.199	0.264	1
GO_GOLGI_CISTERNA_MEMBRANE	71	0.28	1.22	0.122	0.264	1
GO_NEGATIVE_REGULATION_OF_DNA_RECOMBINATION	16	0.4	1.21	0.221	0.264	1
GO_POSITIVE_REGULATION_OF_NATURAL_KILLER_CELL_MEDIATED_IMMUNITY	20	0.38	1.21	0.203	0.264	1
GO_REGULATION_OF_RECEPTOR_BIOSYNTHETIC_PROCESS	21	0.38	1.21	0.23	0.265	1
GO_PROTEIN_PHOSPHATASE_2A_BINDING	28	0.34	1.21	0.186	0.265	1
GO_U2_TYPE_SPLICEOSOMAL_COMPLEX	31	0.34	1.21	0.196	0.265	1
GO_ENZYME_INHIBITOR_ACTIVITY	361	0.22	1.21	0.041	0.266	1
GO_SCAVENGER_RECEPTOR_ACTIVITY	45	0.31	1.21	0.167	0.266	1
GO_TONGUE_DEVELOPMENT	20	0.38	1.21	0.22	0.266	1
GO_PHOSPHATIDYLSERINE_METABOLIC_PROCESS	27	0.35	1.21	0.19	0.268	1
GO_TRANSFERASE_ACTIVITY_TRANSFERRING_ACYL_GROUPS	229	0.23	1.21	0.062	0.268	1
GO_RETROGRADE_PROTEIN_TRANSPORT_ER_TO_CYTOSOL	16	0.41	1.21	0.204	0.269	1
GO_REGULATION_OF_PROTEIN_ACTIVATION_CASCADE	34	0.33	1.21	0.193	0.269	1
GO_REGULATION_OF_PHAGOCYTOSIS	67	0.28	1.21	0.15	0.27	1
GO_REGULATION_OF_PROTEIN_IMPORT	182	0.24	1.21	0.076	0.27	1

GO_TAXIS	462	0.21	1.21	0.035	0.27	1
GO_SEX_DIFFERENTIATION	264	0.23	1.21	0.053	0.272	1
GO_FEMALE_GENITALIA_DEVELOPMENT	16	0.4	1.21	0.232	0.273	1
GO_HYDROLASE_ACTIVITY_HYDROLYZING_N_GLYCOSYL_COMPOUNDS	23	0.37	1.21	0.181	0.273	1
GO_DNA_DIRECTED_RNA_POLYMERASE_II_CORE_COMPLEX	17	0.39	1.21	0.218	0.273	1
GO_INTEGRIN_BINDING	105	0.26	1.21	0.126	0.274	1
GO_ACTIN_FILAMENT_ORGANIZATION	170	0.24	1.2	0.089	0.275	1
GO_CELLULAR_RESPONSE_TO_EPIDERMAL_GROWTH_FACTOR_STIMUL	25	0.36	1.2	0.198	0.276	1
GO_POSITIVE_REGULATION_OF_HOMEOSTATIC_PROCESS	210	0.23	1.2	0.073	0.277	1
GO_PHOSPHATE_TRANSMEMBRANE_TRANSPORTER_ACTIVITY	30	0.33	1.2	0.19	0.277	1
GO_MACROMOLECULE_TRANSMEMBRANE_TRANSPORTER_ACTIVITY	20	0.38	1.2	0.21	0.278	1
GO_CELL_ADHESION_MOLECULE_BINDING	186	0.23	1.2	0.091	0.278	1
GO_POSITIVE_REGULATION_OF_CYTOSKELETON_ORGANIZATION	172	0.24	1.2	0.075	0.279	1
GO_HISTONE_METHYLTRANSFERASE_ACTIVITY	58	0.29	1.2	0.155	0.278	1
GO_REGULATION_OF_PROTEIN_EXPORT_FROM_NUCLEUS	33	0.33	1.2	0.179	0.278	1
GO_GROWTH_FACTOR_RECEPTOR_BINDING	124	0.25	1.2	0.111	0.279	1
GO_METHYLATION	262	0.23	1.2	0.063	0.279	1
GO_ORGANIC_ACID_BIOSYNTHETIC_PROCESS	261	0.23	1.2	0.062	0.279	1
GO_ANGIOGENESIS	292	0.22	1.2	0.077	0.279	1
GO_OSSIFICATION	248	0.23	1.2	0.068	0.28	1
GO_RECEPTOR_CATABOLIC_PROCESS	16	0.4	1.2	0.223	0.281	1
GO_GLUCOSE_METABOLIC_PROCESS	118	0.25	1.2	0.121	0.281	1
GO_RESPONSE_TO_PLATELET_DERIVED_GROWTH_FACTOR	18	0.39	1.2	0.242	0.281	1
GO_PROTEIN_HETERODIMERIZATION_ACTIVITY	456	0.22	1.2	0.039	0.281	1
GO_GTP_DEPENDENT_PROTEIN_BINDING	17	0.39	1.2	0.214	0.283	1
GO_TRANSCRIPTIONAL_REPRESSOR_ACTIVITY_RNA_POLYMERASE_II_CORE_PROMOTER_PROXIMAL_REGION_SEQUENCE_SPECIFIC_BINDING	105	0.26	1.2	0.122	0.284	1
GO_PHOSPHATIDYLGLYCEROL_METABOLIC_PROCESS	29	0.34	1.2	0.212	0.284	1
GO_PROSTATE_GLAND_DEVELOPMENT	41	0.31	1.2	0.152	0.286	1
GO_NEGATIVE_REGULATION_OF_SEQUENCE_SPECIFIC_DNA_BINDING_TRANSCRIPTION_FACTOR_ACTIVITY	133	0.25	1.2	0.127	0.286	1
GO_ESTABLISHMENT_OF_PROTEIN_LOCALIZATION_TO_ORGANELLE	355	0.22	1.19	0.05	0.286	1
GO_PRENYLTRANSFERASE_ACTIVITY	15	0.4	1.19	0.228	0.286	1
GO_LIPOPROTEIN_METABOLIC_PROCESS	124	0.25	1.19	0.103	0.288	1
GO_REGULATION_OF_DNA_METHYLATION	16	0.39	1.19	0.206	0.289	1
GO_MRNA_BINDING	147	0.24	1.19	0.093	0.289	1
GO_RESPONSE_TO_TRANSITION_METAL_NANOPARTICLE	148	0.24	1.19	0.127	0.289	1
GO_HYALURONIC_ACID_BINDING	22	0.35	1.19	0.216	0.289	1
GO_LYSINE_ACETYLATED_HISTONE_BINDING	17	0.39	1.19	0.223	0.289	1
GO_EMBRYONIC_DIGESTIVE_TRACT_MORPHOGENESIS	17	0.39	1.19	0.221	0.29	1
GO_MONOCARBOXYLIC_ACID_METABOLIC_PROCESS	491	0.21	1.19	0.029	0.293	1
GO_PORE_COMPLEX	17	0.39	1.19	0.238	0.293	1
GO_KETONE_BIOSYNTHETIC_PROCESS	24	0.34	1.19	0.208	0.293	1
GO_CHAPERONE_BINDING	81	0.27	1.19	0.152	0.294	1
GO_TRANSCRIPTIONAL_ACTIVATOR_ACTIVITY_RNA_POLYMERASE_II_DISTAL_ENHANCER_SEQUENCE_SPECIFIC_BINDING	26	0.35	1.19	0.196	0.294	1
GO_PROTEIN_SELF_ASSOCIATION	44	0.31	1.19	0.193	0.296	1
GO_CELL_CELL_ADHERENS_JUNCTION	53	0.29	1.19	0.203	0.296	1
GO_REGULATION_OF_MITOPHAGY	42	0.3	1.19	0.176	0.296	1
GO_TISSUE_MIGRATION	82	0.27	1.19	0.156	0.297	1
GO_POSITIVE_REGULATION_OF_ERK1_AND_ERK2_CASCADE	170	0.24	1.19	0.105	0.297	1
GO_REGULATION_OF_MACROPHAGE_CHEMOTAXIS	16	0.39	1.19	0.232	0.297	1
GO_POSITIVE_REGULATION_OF_INTRACELLULAR_TRANSPORT	367	0.22	1.19	0.053	0.298	1
GO_THYMUS_DEVELOPMENT	46	0.29	1.19	0.183	0.298	1
GO_DNA_METHYLATION_OR_DEMETHYLATION	59	0.29	1.19	0.161	0.298	1
GO_INTERFERON_GAMMA_PRODUCTION	15	0.4	1.18	0.242	0.298	1
GO_NUCLEOTIDE_TRANSMEMBRANE_TRANSPORTER_ACTIVITY	19	0.38	1.18	0.217	0.299	1
GO_PROTEIN_K63_LINKED_UBIQUITINATION	36	0.32	1.18	0.199	0.299	1
GO_ANTIGEN_PROCESSING_AND_PRESENTATION_VIA_MHC_CLASS_IB	15	0.4	1.18	0.22	0.3	1
GO_REGULATION_OF_PROTEIN_SERINE_THREONINE_KINASE_ACTIVITY	466	0.21	1.18	0.03	0.301	1
GO_REGULATION_OF_BINDING	283	0.22	1.18	0.08	0.301	1
GO_POSITIVE_REGULATION_OF_MRNA_METABOLIC_PROCESS	45	0.3	1.18	0.201	0.301	1
GO_AROMATIC_AMINO_ACID_FAMILY_METABOLIC_PROCESS	28	0.33	1.18	0.225	0.303	1
GO_ESTROGEN_METABOLIC_PROCESS	23	0.36	1.18	0.219	0.304	1
GO_REGULATION_OF_NITRIC_OXIDE_BIOSYNTHETIC_PROCESS	52	0.29	1.18	0.175	0.305	1
GO_APOPTOTIC_PROCESS_INVOLVED_IN_DEVELOPMENT	21	0.37	1.18	0.23	0.305	1
GO_REGULATION_OF_INTRACELLULAR_ESTROGEN_RECEPTOR_SIGNALING_PATHWAY	27	0.33	1.18	0.19	0.305	1
GO_REGULATION_OF_CELL_SUBSTRATE_ADHESION	171	0.24	1.18	0.114	0.305	1
GO_CCR4_NOT_COMPLEX	15	0.4	1.18	0.253	0.305	1
GO_ACTIN_FILAMENT_BUNDLE_ORGANIZATION	48	0.29	1.18	0.199	0.306	1
GO_THYMIC_T_CELL_SELECTION	19	0.37	1.18	0.229	0.309	1
GO_POSITIVE_REGULATION_OF_TRANSCRIPTION_FACTOR_IMPORT_INTO_NUCLEUS	51	0.3	1.18	0.167	0.309	1
GO_HEME_METABOLIC_PROCESS	28	0.33	1.18	0.237	0.309	1
GO_POSITIVE_REGULATION_OF_SMOOTH_MUSCLE_CELL_PROLIFERATIO	58	0.28	1.18	0.175	0.309	1
GO_CELL_CELL_JUNCTION_ASSEMBLY	73	0.27	1.18	0.168	0.31	1
GO_EMBRYONIC_ORGAN_DEVELOPMENT	404	0.21	1.18	0.065	0.31	1
GO_CELLULAR_AMINO_ACID_BIOSYNTHETIC_PROCESS	91	0.26	1.18	0.151	0.31	1
GO_PROTEIN_SERINE_THREONINE_KINASE_ACTIVITY	441	0.21	1.18	0.053	0.31	1
GO_DIOXYGENASE_ACTIVITY	87	0.26	1.17	0.14	0.31	1
GO_COENZYME_BINDING	175	0.23	1.17	0.119	0.312	1
GO_MODULATION_OF_TRANSCRIPTION_IN_OTHER_ORGANISM_INVOLVED_IN_SYMBIOTIC_INTERACTION	22	0.36	1.17	0.222	0.313	1
GO_REGULATION_OF_ENDOPLASMIC_RETICULUM_UNFOLDED_PROTEIN_RESPONSE	28	0.33	1.17	0.208	0.313	1
GO_PROTEIN_KINASE_C_BINDING	50	0.29	1.17	0.19	0.315	1

GO_POSITIVE_REGULATION_OF_PROTEIN_KINASE_B_SIGNALING	81	0.26	1.17	0.163	0.317	1
GO_TRANSCRIPTIONAL_ACTIVATOR_ACTIVITY_RNA_POLYMERASE_II_CORE_PROMOTER_PROXIMAL_REGION_SEQUENCE_SPECIFIC_BINDING	226	0.22	1.17	0.11	0.318	1
GO_LAMELLIPODIUM_MEMBRANE	18	0.38	1.17	0.246	0.319	1
GO_PURINE_CONTAINING_COMPOUND_BIOSYNTHETIC_PROCESS	137	0.24	1.17	0.15	0.321	1
GO_DNA_DIRECTED_RNA_POLYMERASE_II_HOLOENZYME	95	0.26	1.17	0.17	0.321	1
GO_PROTEIN_ACYLATION	150	0.24	1.17	0.135	0.321	1
GO_GTPASE_ACTIVITY	235	0.22	1.17	0.114	0.322	1
GO_INDOLALKYLAMINE_METABOLIC_PROCESS	17	0.39	1.17	0.261	0.322	1
GO_ORGANOPHOSPHATE_ESTER_TRANSPORT	89	0.26	1.17	0.184	0.323	1
GO_ESTABLISHMENT_OF_TISSUE_POLARITY	17	0.37	1.16	0.268	0.325	1
GO_PROTON_TRANSPORTING_ATP_SYNTHASE_COMPLEX	22	0.35	1.16	0.257	0.326	1
GO_ODONTOGENESIS	105	0.25	1.16	0.143	0.326	1
GO_MANGANESE_ION_BINDING	48	0.29	1.16	0.191	0.326	1
GO_REGULATION_OF_ANION_TRANSMEMBRANE_TRANSPORT	30	0.32	1.16	0.237	0.328	1
GO_MULTICELLULAR_ORGANISMAL_WATER_HOMEOSTASIS	57	0.28	1.16	0.214	0.329	1
GO_COFACTOR_BINDING	258	0.22	1.16	0.118	0.33	1
GO_TRANSCRIPTIONAL_ACTIVATOR_ACTIVITY_RNA_POLYMERASE_II_TRANSCRIPTION_REGULATORY_REGION_SEQUENCE_SPECIFIC_BINDING	315	0.22	1.16	0.072	0.33	1
GO_NEGATIVE_REGULATION_OF_PEPTIDYL_TYROSINE_PHOSPHORYLATI	39	0.31	1.16	0.208	0.33	1
GO_CELLULAR_RESPONSE_TO_FLUID_SHEAR_STRESS	19	0.37	1.16	0.253	0.334	1
GO_REGULATION_OF_SYNCYTIUM_FORMATION_BY_PLASMA_MEMBRANE_FUSION	25	0.33	1.16	0.235	0.335	1
GO_REGULATION_OF_VIRAL_RELEASE_FROM_HOST_CELL	30	0.33	1.16	0.23	0.336	1
GO_REGULATION_OF_GLUCOSE_TRANSPORT	98	0.25	1.16	0.175	0.336	1
GO_RESPONSE_TO_FATTY_ACID	83	0.26	1.16	0.17	0.337	1
GO_ACETYLTRANSFERASE_ACTIVITY	96	0.25	1.16	0.185	0.337	1
GO_NEGATIVE_REGULATION_OF_RESPONSE_TO_EXTERNAL_STIMULUS	269	0.21	1.16	0.101	0.337	1
GO_REGULATION_OF_ERBB_SIGNALING_PATHWAY	83	0.26	1.16	0.192	0.337	1
GO_REGULATION_OF_EPITHELIAL_CELL_PROLIFERATION	284	0.22	1.16	0.098	0.337	1
GO_PROTEIN_REFOLDING	20	0.35	1.15	0.24	0.338	1
GO_CHEMOATTRACTANT_ACTIVITY	27	0.33	1.15	0.249	0.339	1
GO_ERK1_AND_ERK2_CASCADE	22	0.35	1.15	0.242	0.339	1
GO_BONE_REMODELING	35	0.31	1.15	0.258	0.339	1
GO_MORPHOGENESIS_OF_A_POLARIZED_EPITHELIUM	28	0.32	1.15	0.235	0.34	1
GO_MALE_SEX_DIFFERENTIATION	148	0.23	1.15	0.129	0.34	1
GO_ENDOPLASMIC_RETICULUM_GOLGI_INTERMEDIATE_COMPARTMENT	102	0.25	1.15	0.191	0.341	1
GO_EXTRACELLULAR_MATRIX_COMPONENT	123	0.24	1.15	0.15	0.342	1
GO_FRIZZLED_BINDING	36	0.31	1.15	0.239	0.343	1
GO_LIPOPROTEIN_BIOSYNTHETIC_PROCESS	85	0.25	1.15	0.198	0.345	1
GO_POSITIVE_REGULATION_OF_MAST_CELL_ACTIVATION	16	0.37	1.15	0.282	0.345	1
GO_MODIFIED_AMINO_ACID_TRANSPORT	26	0.34	1.15	0.267	0.345	1
GO_NEGATIVE_REGULATION_OF_PRODUCTION_OF_MOLECULAR_MEDIATOR_OF_IMMUNE_RESPONSE	29	0.33	1.15	0.266	0.345	1
GO_POSITIVE_REGULATION_OF_WOUND_HEALING	48	0.29	1.15	0.217	0.347	1
GO_RESPONSE_TO_OXYGEN_LEVELS	310	0.21	1.15	0.089	0.347	1
GO_LONG_CHAIN_FATTY_ACID_METABOLIC_PROCESS	87	0.25	1.15	0.203	0.349	1
GO_DNA_DEMETHYLATION	15	0.38	1.15	0.282	0.351	1
GO_ANTERIOR_POSTERIOR_PATTERN_SPECIFICATION	194	0.22	1.15	0.128	0.351	1
GO_POSITIVE_REGULATION_OF_PROTEIN_TYROSINE_KINASE_ACTIVITY	37	0.3	1.14	0.227	0.352	1
GO_CYTOCHROME_COMPLEX_ASSEMBLY	25	0.33	1.14	0.26	0.352	1
GO_DICARBOXYLIC_ACID_METABOLIC_PROCESS	99	0.25	1.14	0.187	0.352	1
GO_C21_STEROID_HORMONE_METABOLIC_PROCESS	25	0.34	1.14	0.282	0.353	1
GO_PEPTIDE_N_ACETYLTRANSFERASE_ACTIVITY	60	0.27	1.14	0.206	0.353	1
GO_PRESPLICEOSOME	21	0.35	1.14	0.282	0.353	1
GO_N_ACETYLTRANSFERASE_ACTIVITY	80	0.26	1.14	0.195	0.353	1
GO_TRANSFERASE_ACTIVITY_TRANSFERRING_ALKYL_OR_ARYL_OTHER_THAN_METHYL_GROUPS	61	0.27	1.14	0.249	0.353	1
GO_NEGATIVE_REGULATION_OF_VIRAL_RELEASE_FROM_HOST_CELL	15	0.38	1.14	0.276	0.354	1
GO_VITAMIN_BIOSYNTHETIC_PROCESS	15	0.38	1.14	0.298	0.355	1
GO_PROTEIN_ACETYLATION	120	0.24	1.14	0.177	0.354	1
GO_POSITIVE_REGULATION_OF_CELL_ADHESION_MEDIATED_BY_INTEG	16	0.38	1.14	0.278	0.356	1
GO_EMBRYONIC_DIGESTIVE_TRACT_DEVELOPMENT	33	0.31	1.14	0.257	0.356	1
GO_HOMEOSTASIS_OF_NUMBER_OF_CELLS	174	0.23	1.14	0.144	0.356	1
GO_CYTOCHROME_COMPLEX	21	0.35	1.14	0.275	0.356	1
GO_TBP_CLASS_PROTEIN_BINDING	20	0.35	1.14	0.273	0.359	1
GO_REGULATION_OF_MYOTUBE_DIFFERENTIATION	55	0.28	1.14	0.258	0.359	1
GO_CELL_CELL_RECOGNITION	60	0.27	1.14	0.228	0.359	1
GO_RETINOIC_ACID_RECEPTOR_BINDING	40	0.3	1.14	0.244	0.36	1
GO_BRANCH_ELONGATION_OF_AN_EPITHELIUM	17	0.38	1.14	0.268	0.36	1
GO_BINDING_OF_SPERM_TO_ZONA_PELLUCIDA	33	0.31	1.14	0.25	0.362	1
GO_REGULATION_OF_NITRIC_OXIDE_SYNTHASE_BIOSYNTHETIC_PROCE	19	0.36	1.14	0.279	0.363	1
GO_CELLULAR_RESPONSE_TO_GAMMA_RADIATION	19	0.36	1.14	0.29	0.363	1
GO_HEMOSTASIS	308	0.21	1.14	0.118	0.363	1
GO_RECYCLING_ENDOSOME_MEMBRANE	40	0.3	1.14	0.273	0.364	1
GO_WATER_HOMEOSTASIS	69	0.26	1.14	0.232	0.364	1
GO_NEGATIVE_REGULATION_OF_MEIOTIC_CELL_CYCLE	19	0.36	1.14	0.288	0.365	1
GO_LUNG_ALVEOLUS_DEVELOPMENT	41	0.29	1.13	0.235	0.366	1
GO_ECTODERM_DEVELOPMENT	21	0.35	1.13	0.26	0.367	1
GO_CYTOKINE_PRODUCTION_INVOLVED_IN_IMMUNE_RESPONSE	17	0.37	1.13	0.279	0.368	1
GO_REGULATION_OF_EPIDERMAL_GROWTH_FACTOR_ACTIVATED_RECEPTOR_ACTIVITY	23	0.35	1.13	0.269	0.368	1
GO_ENDOCYTIC_VESICLE_LUMEN	17	0.37	1.13	0.266	0.369	1
GO_REGULATION_OF_DEFENSE_RESPONSE_TO_VIRUS_BY_VIRUS	29	0.32	1.13	0.277	0.369	1
GO_SPLICEOSOMAL_COMPLEX_ASSEMBLY	43	0.29	1.13	0.25	0.369	1
GO_EPITHELIAL_CELL_PROLIFERATION	88	0.25	1.13	0.206	0.369	1
GO_NEGATIVE_REGULATION_OF_CELL_SUBSTRATE_ADHESION	53	0.28	1.13	0.235	0.369	1

GO_REGULATION_OF_RNA_SPLICING	83	0.25	1.13	0.193	0.37	1
GO_S_ADENOSYLMETHIONINE_DEPENDENT_METHYLTRANSFERASE_ACTIVITY	128	0.24	1.13	0.186	0.371	1
GO_MEMBRANE_PROTEIN_PROTEOLYSIS	35	0.3	1.13	0.265	0.372	1
GO_REGULATION_OF_PROTEIN_COMPLEX_ASSEMBLY	366	0.21	1.13	0.096	0.372	1
GO_POSITIVE_REGULATION_OF_PROTEASOMAL_PROTEIN_CATABOLIC_PROCESS	98	0.24	1.13	0.225	0.372	1
GO_EPITHELIAL_STRUCTURE_MAINTENANCE	21	0.35	1.13	0.261	0.372	1
GO_DNA_ALKYLATION	45	0.28	1.13	0.242	0.373	1
GO_PROTEIN_ACTIVATION_CASCADE	67	0.26	1.13	0.218	0.375	1
GO_CANONICAL_WNT_SIGNALING_PATHWAY	95	0.25	1.13	0.239	0.375	1
GO_REGULATION_OF_ACTIN_FILAMENT_LENGTH	152	0.23	1.13	0.201	0.375	1
GO_NEGATIVE_REGULATION_OF_ANOIKIS	17	0.37	1.13	0.285	0.375	1
GO_COCHLEA_MORPHOGENESIS	21	0.34	1.13	0.305	0.376	1
GO_NEGATIVE_REGULATION_OF_REACTIVE_OXYGEN_SPECIES_METABOLIC_PROCESS	42	0.29	1.13	0.24	0.377	1
GO_ORGANIC_ACID_BINDING	205	0.22	1.13	0.157	0.378	1
GO_POSITIVE_REGULATION_OF_MAPK_CASCADE	466	0.2	1.13	0.109	0.378	1
GO_PHOSPHOLIPID_TRANSPORT	58	0.27	1.13	0.261	0.378	1
GO_SUPEROXIDE_METABOLIC_PROCESS	33	0.31	1.12	0.277	0.381	1
GO_CLUSTER_OF_ACTIN_BASED_CELL_PROJECTIONS	137	0.23	1.12	0.193	0.381	1
GO_REGULATION_OF_TRANSCRIPTION_FROM_RNA_POLYMERASE_II_PROMOTER_IN_RESPONSE_TO_HYPOXIA	32	0.31	1.12	0.328	0.382	1
GO_NEGATIVE_REGULATION_OF_BINDING	131	0.23	1.12	0.191	0.387	1
GO_ER_ASSOCIATED_UBIQUITIN_DEPENDENT_PROTEIN_CATABOLIC_PROCESS	61	0.27	1.12	0.232	0.389	1
GO_REGULATION_OF_TISSUE_REMODELING	62	0.26	1.12	0.246	0.392	1
GO_POLYOL_TRANSPORT	18	0.36	1.12	0.309	0.391	1
GO_B_CELL_RECEPTOR_SIGNALING_PATHWAY	34	0.3	1.12	0.278	0.393	1
GO_PROTEIN_TRANSPORTER_ACTIVITY	97	0.24	1.12	0.231	0.393	1
GO_P53_BINDING	66	0.26	1.12	0.251	0.393	1
GO_NEGATIVE_REGULATION_OF_LIPID_METABOLIC_PROCESS	77	0.25	1.12	0.249	0.393	1
GO_REGULATION_OF_OXIDATIVE_STRESS_INDUCED_CELL_DEATH	46	0.28	1.12	0.277	0.394	1
GO_FEMALE_GAMETE_GENERATION	96	0.24	1.12	0.249	0.394	1
GO_ENDOMEMBRANE_SYSTEM_ORGANIZATION	458	0.2	1.12	0.101	0.394	1
GO_HISTONE_DEACETYLASE_BINDING	104	0.24	1.12	0.225	0.394	1
GO_NEGATIVE_REGULATION_OF_TELOMERE_MAINTENANCE_VIA_TELOMERE_LENGTHENING	17	0.36	1.11	0.286	0.395	1
GO_REGULATION_OF_OSTEOBLAST_PROLIFERATION	23	0.34	1.11	0.305	0.396	1
GO_INTRACELLULAR_ESTROGEN_RECEPTOR_SIGNALING_PATHWAY	17	0.37	1.11	0.319	0.396	1
GO_ALCOHOL_METABOLIC_PROCESS	345	0.2	1.11	0.148	0.396	1
GO_CELLULAR_SENESCENCE	31	0.31	1.11	0.271	0.397	1
GO_REGULATION_OF_TRANSCRIPTION_INITIATION_FROM_RNA_POLYMERASE_II_PROMOTER	23	0.33	1.11	0.306	0.397	1
GO_NEGATIVE_REGULATION_OF_LIPID_TRANSPORT	26	0.32	1.11	0.279	0.398	1
GO_FLAVIN_ADENINE_DINUCLEOTIDE_BINDING	73	0.25	1.11	0.263	0.399	1
GO_ATP_BIOSYNTHETIC_PROCESS	36	0.3	1.11	0.287	0.4	1
GO_MITOCHONDRIAL_PROTEIN_COMPLEX	131	0.23	1.11	0.222	0.399	1
GO_NEGATIVE_REGULATION_OF_PROTEIN_SERINE_THREONINE_KINASE_ACTIVITY	126	0.23	1.11	0.241	0.399	1
GO_MODULATION_OF_GROWTH_OF_SYMBIONT_INVOLVED_IN_INTERACTION_WITH_HOST	16	0.37	1.11	0.309	0.399	1
GO_GENITALIA_DEVELOPMENT	42	0.29	1.11	0.262	0.399	1
GO_NEGATIVE_REGULATION_OF_FIBROBLAST_PROLIFERATION	27	0.32	1.11	0.303	0.399	1
GO_PYRIMIDINE_CONTAINING_COMPOUND_CATABOLIC_PROCESS	32	0.31	1.11	0.307	0.4	1
GO_OXIDOREDUCTASE_ACTIVITY_ACTING_ON_THE_CH_NH_GROUP_OF_DONORS_NAD_OR_NADP_AS_ACCEPTOR	17	0.36	1.11	0.298	0.401	1
GO_LIPASE_INHIBITOR_ACTIVITY	17	0.37	1.11	0.309	0.401	1
GO_MYELOID_CELL_HOMEOSTASIS	87	0.25	1.11	0.268	0.401	1
GO_REGULATION_OF_PROTEIN_BINDING	168	0.22	1.11	0.197	0.401	1
GO_NEGATIVE_REGULATION_OF_CHEMOKINE_PRODUCTION	16	0.36	1.11	0.291	0.402	1
GO_PROTEIN_PHOSPHATASE_1_BINDING	19	0.35	1.11	0.305	0.402	1
GO_REGULATION_OF_B_CELL_RECEPTOR_SIGNALING_PATHWAY	15	0.37	1.11	0.313	0.402	1
GO_METHYL_CPG_BINDING	18	0.36	1.11	0.28	0.402	1
GO_PEPTIDE_CATABOLIC_PROCESS	24	0.33	1.11	0.314	0.405	1
GO_BLOOD_VESSEL_MORPHOGENESIS	363	0.2	1.11	0.133	0.406	1
GO_REGULATION_OF_BONE_RESORPTION	33	0.3	1.11	0.288	0.408	1
GO_POSITIVE_REGULATION_OF_MRNA_PROCESSING	32	0.3	1.11	0.28	0.409	1
GO_POSITIVE_REGULATION_OF_PROTEIN_LOCALIZATION_TO_CELL_PERIPHERY	37	0.29	1.1	0.293	0.409	1
GO_G_PROTEIN_COUPLED_PURINERGIC_RECEPTOR_SIGNALING_PATH	20	0.34	1.1	0.304	0.409	1
GO_CIS_TRANS_ISOMERASE_ACTIVITY	44	0.28	1.1	0.303	0.413	1
GO_REGULATION_OF_ENDOPLASMIC_RETICULUM_STRESS_INDUCED_INTRINSIC_APOPTOTIC_SIGNALING_PATHWAY	30	0.31	1.1	0.315	0.414	1
GO_MIDBRAIN_DEVELOPMENT	89	0.25	1.1	0.262	0.416	1
GO_GUANYL_NUCLEOTIDE_BINDING	360	0.2	1.1	0.133	0.416	1
GO_PLATELET_ACTIVATION	141	0.23	1.1	0.232	0.416	1
GO_MONOOXYGENASE_ACTIVITY	91	0.24	1.1	0.253	0.417	1
GO_PEPTIDYL_THREONINE_MODIFICATION	46	0.28	1.1	0.293	0.417	1
GO_S_ACYLTRANSFERASE_ACTIVITY	28	0.31	1.1	0.311	0.42	1
GO_PROSTATE_GLAND_MORPHOGENESIS	23	0.33	1.1	0.298	0.421	1
GO_NEURON_APOPTOTIC_PROCESS	34	0.29	1.1	0.316	0.424	1
GO_POLYUBIQUITIN_BINDING	41	0.29	1.1	0.315	0.425	1
GO_PATTERNING_OF_BLOOD_VESSELS	32	0.3	1.1	0.309	0.426	1
GO_CYSTEINE_TYPE_ENDOPEPTIDASE_INHIBITOR_ACTIVITY_INVOLVED_IN_APOPTOTIC_PROCESS	23	0.33	1.09	0.307	0.427	1
GO_PHOSPHOTYROSINE_BINDING	15	0.37	1.09	0.335	0.43	1

GO_VENTRICULAR_CARDIAC_MUSCLE_CELL_DIFFERENTIATION	19	0.35	1.09	0.329	0.43	1
GO_MITOCHONDRIAL_ATP_SYNTHESIS_COUPLED_PROTON_TRANSPORT	17	0.35	1.09	0.343	0.43	1
GO_REGULATION_OF_PLASMA_MEMBRANE_ORGANIZATION	73	0.25	1.09	0.27	0.431	1
GO_RESPONSE_TO_ORGANOPHOSPHORUS	139	0.23	1.09	0.249	0.431	1
GO_POSITIVE_REGULATION_OF_INTRACELLULAR_PROTEIN_TRANSPORT	255	0.2	1.09	0.201	0.431	1
GO_INTERACTION_WITH_SYMBIONT	52	0.27	1.09	0.292	0.432	1
GO_REGULATION_OF_ESTABLISHMENT_OF_PROTEIN_LOCALIZATION_TO_PLASMA_MEMBRANE	48	0.27	1.09	0.295	0.434	1
GO_SYNAPSIS	34	0.3	1.09	0.286	0.434	1
GO_MEDIATOR_COMPLEX	34	0.29	1.09	0.298	0.435	1
GO_REGULATION_OF_OXIDOREDUCTASE_ACTIVITY	88	0.24	1.09	0.305	0.436	1
GO_NEGATIVE_REGULATION_OF_REACTIVE_OXYGEN_SPECIES_BIOSYNTHETIC_PROCESS	16	0.37	1.09	0.344	0.436	1
GO_EMBRYONIC_FORELIMB_MORPHOGENESIS	32	0.3	1.09	0.315	0.437	1
GO_ACTIN_CYTOSKELETON	432	0.19	1.09	0.156	0.437	1
GO_GPI_ANCHOR_METABOLIC_PROCESS	33	0.3	1.09	0.32	0.437	1
GO_ACTIVATION_OF_JUN_KINASE_ACTIVITY	35	0.29	1.09	0.301	0.437	1
GO_PYRIMIDINE_NUCLEOSIDE_CATABOLIC_PROCESS	21	0.34	1.09	0.325	0.438	1
GO_POSITIVE_REGULATION_OF_HISTONE_H3_K4_METHYLATION	16	0.35	1.09	0.335	0.439	1
GO_B_CELL_HOMEOSTASIS	21	0.33	1.09	0.334	0.439	1
GO_ADA2_GCN5_ADA3_TRANSCRIPTION_ACTIVATOR_COMPLEX	15	0.37	1.09	0.326	0.44	1
GO_BETA_CATENIN_DESTRUCTION_COMPLEX_DISASSEMBLY	22	0.33	1.09	0.33	0.441	1
GO_OXIDOREDUCTASE_ACTIVITY_ACTING_ON_NAD_P_H	91	0.24	1.08	0.261	0.443	1
GO_RESPONSE_TO_OXYGEN_RADICAL	18	0.34	1.08	0.347	0.443	1
GO_NEGATIVE_REGULATION_OF_CHROMATIN_MODIFICATION	45	0.27	1.08	0.315	0.445	1
GO_NEGATIVE_REGULATION_OF_LIPID_BIOSYNTHETIC_PROCESS	43	0.28	1.08	0.312	0.445	1
GO_OXIDOREDUCTASE_ACTIVITY_ACTING_ON_THE_CH_NH2_GROUP_OF_DONORS_OXYGEN_AS_ACCEPTOR	15	0.37	1.08	0.343	0.446	1
GO_REGULATION_OF_NATURAL_KILLER_CELL_ACTIVATION	27	0.31	1.08	0.324	0.445	1
GO_RESPONSE_TO_ANGIOTENSIN	17	0.36	1.08	0.333	0.448	1
GO_BENZENE_CONTAINING_COMPOUND_METABOLIC_PROCESS	24	0.32	1.08	0.328	0.448	1
GO_TISSUE_HOMEOSTASIS	164	0.22	1.08	0.261	0.448	1
GO_REGULATION_OF_STRIATED_MUSCLE_CELL_DIFFERENTIATION	84	0.24	1.08	0.288	0.449	1
GO_REGULATION_OF_EMBRYONIC_DEVELOPMENT	112	0.23	1.08	0.255	0.451	1
GO_MISFOLDED_OR_INCOMPLETELY_SYNTHESIZED_PROTEIN_CATABOLIC_PROCESS	16	0.35	1.08	0.333	0.451	1
GO_LYSOPHOSPHOLIPASE_ACTIVITY	19	0.34	1.08	0.325	0.452	1
GO_REGULATION_OF_PROTEIN_STABILITY	220	0.21	1.08	0.239	0.454	1
GO_POSITIVE_REGULATION_OF_EPITHELIAL_CELL_PROLIFERATION	153	0.22	1.08	0.281	0.455	1
GO_NEGATIVE_REGULATION_OF_EPITHELIAL_CELL_DIFFERENTIATION	36	0.29	1.08	0.333	0.455	1
GO_REGULATION_OF_CELLULAR_EXTRAVASATION	22	0.33	1.08	0.317	0.456	1
GO_CORE_PROMOTER_PROXIMAL_REGION_DNA_BINDING	366	0.19	1.08	0.198	0.456	1
GO_NEUTROPHIL_MEDIATED_IMMUNITY	22	0.33	1.07	0.332	0.46	1
GO_CARBOXYLIC_ESTER_HYDROLASE_ACTIVITY	130	0.22	1.07	0.299	0.463	1
GO_REGULATION_OF_MYELOID_LEUKOCYTE_DIFFERENTIATION	107	0.23	1.07	0.261	0.464	1
GO_MEMBRANE_INVAGINATION	28	0.31	1.07	0.33	0.467	1
GO_POSITIVE_REGULATION_OF_BINDING	127	0.22	1.07	0.288	0.467	1
GO_DRUG_BINDING	108	0.23	1.07	0.291	0.468	1
GO_RESPONSE_TO_ACID_CHEMICAL	313	0.2	1.07	0.236	0.471	1
GO_CELLULAR_CARBOHYDRATE_BIOSYNTHETIC_PROCESS	51	0.26	1.07	0.349	0.471	1
GO_RESPONSE_TO_MECHANICAL_STIMULUS	208	0.21	1.07	0.272	0.471	1
GO_POSITIVE_CHEMOTAXIS	36	0.29	1.07	0.347	0.471	1
GO_CYCLIN_DEPENDENT_PROTEIN_KINASE_HOLOENZYME_COMPLEX	30	0.3	1.07	0.316	0.471	1
GO_NEGATIVE_REGULATION_OF_HISTONE_METHYLATION	16	0.35	1.07	0.39	0.471	1
GO_REGULATION_OF_RECEPTOR_BINDING	17	0.35	1.07	0.359	0.472	1
GO_N_ACYLTRANSFERASE_ACTIVITY	91	0.24	1.07	0.292	0.477	1
GO_REGULATION_OF_MRNA_METABOLIC_PROCESS	105	0.23	1.07	0.296	0.477	1
GO_TETRAPYRROLE_BIOSYNTHETIC_PROCESS	26	0.31	1.07	0.37	0.477	1
GO_GOLGI_CISTERNA	90	0.24	1.07	0.321	0.478	1
GO_EXTRINSIC_COMPONENT_OF_PLASMA_MEMBRANE	135	0.22	1.06	0.295	0.479	1
GO_TRANSITION_METAL_ION_TRANSPORT	106	0.22	1.06	0.3	0.48	1
GO_RHO_GTPASE_BINDING	76	0.24	1.06	0.309	0.48	1
GO_PROTEIN_HOMOTETRAMERIZATION	59	0.25	1.06	0.374	0.481	1
GO_REGULATION_OF_CARDIAC_MUSCLE_CELL_ACTION_POTENTIAL	19	0.34	1.06	0.371	0.482	1
GO_RESPONSE_TO_PURINE_CONTAINING_COMPOUND	158	0.21	1.06	0.289	0.482	1
GO_NEGATIVE_REGULATION_OF_HEMOPOIESIS	127	0.22	1.06	0.302	0.486	1
GO_RESPONSE_TO_CADMIUM_ION	40	0.27	1.06	0.326	0.487	1
GO_PROTEIN_N_LINKED_GLYCOSYLATION	75	0.24	1.06	0.334	0.488	1
GO_ORGANELLE_ASSEMBLY	471	0.19	1.06	0.221	0.488	1
GO_RESPONSE_TO_EPIDERMAL_GROWTH_FACTOR	30	0.3	1.06	0.366	0.489	1
GO_ACETYLTRANSFERASE_COMPLEX	95	0.23	1.06	0.289	0.49	1
GO_ONE_CARBON_METABOLIC_PROCESS	36	0.28	1.06	0.357	0.49	1
GO_THYROID_HORMONE_RECEPTOR_BINDING	30	0.3	1.06	0.371	0.491	1
GO_TRANSLATION_PREINITIATION_COMPLEX	16	0.35	1.06	0.357	0.491	1
GO_COFACTOR_CATABOLIC_PROCESS	20	0.33	1.06	0.384	0.492	1
GO_RESPONSE_TO_MANGANESE_ION	17	0.35	1.06	0.362	0.492	1
GO_ENDOPLASMIC_RETICULUM_TO_CYTOSOL_TRANSPORT	23	0.32	1.06	0.38	0.493	1
GO_MACROMITOPHAGY	126	0.22	1.06	0.337	0.492	1
GO_REGULATION_OF_BONE_REMODELING	42	0.27	1.06	0.349	0.493	1
GO_ANCHORED_COMPONENT_OF_MEMBRANE	148	0.21	1.06	0.315	0.494	1
GO_PLATELET_MORPHOGENESIS	19	0.33	1.05	0.379	0.496	1
GO_PORPHYRIN_CONTAINING_COMPOUND_METABOLIC_PROCESS	35	0.29	1.05	0.382	0.497	1
GO_REGULATION_OF_MYELOID_CELL_APOPTOTIC_PROCESS	23	0.32	1.05	0.367	0.497	1
GO_LIGASE_ACTIVITY	390	0.19	1.05	0.268	0.497	1
GO_REGULATION_OF_STEROL_TRANSPORT	37	0.28	1.05	0.367	0.498	1
GO_GAP_JUNCTION_CHANNEL_ACTIVITY	16	0.35	1.05	0.364	0.498	1
GO_OOCYTE_DIFFERENTIATION	38	0.28	1.05	0.375	0.5	1

GO_GLYCOSAMINOGLYCAN_BINDING	202	0.2	1.05	0.292	0.5	1
GO_BIOMINERAL_TISSUE_DEVELOPMENT	75	0.24	1.05	0.354	0.501	1
GO_HYALURONAN_CATABOLIC_PROCESS	15	0.35	1.05	0.395	0.501	1
GO_MULTIVESICULAR_BODY_ORGANIZATION	29	0.3	1.05	0.376	0.501	1
GO_PROTEIN_METHYLTRANSFERASE_ACTIVITY	77	0.24	1.05	0.355	0.502	1
GO_NEGATIVE_REGULATION_OF_REPRODUCTIVE_PROCESS	54	0.26	1.05	0.335	0.502	1
GO_REGULATION_OF_POSTTRANSCRIPTIONAL_GENE_SILENCING	21	0.33	1.05	0.389	0.502	1
GO_PURINERGIC_NUCLEOTIDE_RECEPTOR_SIGNALING_PATHWAY	22	0.32	1.05	0.374	0.504	1
GO_TRANSFERASE_ACTIVITY_TRANSFERRING_ACYL_GROUPS_OTHER_THAN_AMINO_ACYL_GROUPS	194	0.21	1.05	0.309	0.504	1
GO_SKELETAL_SYSTEM_MORPHOGENESIS	200	0.21	1.05	0.332	0.507	1
GO_RIBONUCLEOSIDE_CATABOLIC_PROCESS	25	0.31	1.05	0.392	0.51	1
GO_TUBE_FORMATION	128	0.22	1.05	0.359	0.51	1
GO_THYMOCYTE_AGGREGATION	45	0.27	1.05	0.381	0.511	1
GO_EMBRYONIC_ORGAN_MORPHOGENESIS	277	0.2	1.04	0.309	0.514	1
GO_ERAD_PATHWAY	72	0.24	1.04	0.355	0.515	1
GO_K63_LINKED_POLYUBIQUITIN_BINDING	19	0.33	1.04	0.37	0.515	1
GO_ISOPRENOID_BINDING	39	0.28	1.04	0.382	0.518	1
GO_NEGATIVE_REGULATION_OF_CELL_MATRIX_ADHESION	30	0.29	1.04	0.4	0.518	1
GO_TELOMERE_MAINTENANCE_VIA_TELOMERASE	17	0.35	1.04	0.384	0.518	1
GO_HAIR_CELL_DIFFERENTIATION	34	0.28	1.04	0.387	0.518	1
GO_PURINE_CONTAINING_COMPOUND_METABOLIC_PROCESS	390	0.19	1.04	0.327	0.518	1
GO_FILAMENTOUS_ACTIN	20	0.33	1.04	0.412	0.519	1
GO_ACTIVATION_OF_MAPK_ACTIVITY	137	0.22	1.04	0.35	0.521	1
GO_ORGANIC_HYDROXY_COMPOUND_TRANSMEMBRANE_TRANSPORTER_ACTIVITY	60	0.24	1.04	0.372	0.521	1
GO_SWI_SNF_SUPERFAMILY_TYPE_COMPLEX	71	0.24	1.04	0.358	0.522	1
GO_POSITIVE_REGULATION_OF_PROTEIN_MATURATION	18	0.34	1.04	0.39	0.522	1
GO_SIGNAL_SEQUENCE_BINDING	39	0.27	1.04	0.394	0.522	1
GO_NEGATIVE_REGULATION_OF_STRESS_ACTIVATED_PROTEIN_KINASE_SIGNALING_CASCADE	41	0.27	1.04	0.397	0.522	1
GO_ENERGY_COUPLED_PROTON_TRANSPORT_DOWN_ELECTROCHEMICAL_GRADIENT	24	0.31	1.04	0.401	0.523	1
GO_EPITHELIAL_CELL_DEVELOPMENT	184	0.21	1.04	0.336	0.523	1
GO_SKELETAL_MUSCLE_TISSUE_REGENERATION	26	0.3	1.04	0.42	0.524	1
GO_SALIVARY_GLAND_DEVELOPMENT	32	0.29	1.04	0.388	0.524	1
GO_ODONTOGENESIS_OF_DENTIN_CONTAINING_TOOTH	75	0.24	1.04	0.378	0.524	1
GO_HORMONE_RECEPTOR_BINDING	163	0.21	1.04	0.36	0.525	1
GO_SIGNAL_TRANSDUCTION_BY_PROTEIN_PHOSPHORYLATION	402	0.19	1.04	0.323	0.525	1
GO_POSITIVE_REGULATION_OF_NOTCH_SIGNALING_PATHWAY	33	0.28	1.04	0.401	0.526	1
GO_PROXIMAL_DISTAL_PATTERN_FORMATION	32	0.29	1.04	0.402	0.525	1
GO_MORPHOGENESIS_OF_AN_EPITHELIAL_FOLD	15	0.36	1.04	0.425	0.526	1
GO_MULTICELLULAR_ORGANISMAL_HOMEOSTASIS	264	0.2	1.04	0.324	0.526	1
GO_APICAL_PART_OF_CELL	356	0.19	1.04	0.321	0.527	1
GO_AEROBIC_RESPIRATION	53	0.26	1.04	0.379	0.526	1
GO_EPHRIN_RECEPTOR_BINDING	24	0.31	1.04	0.382	0.527	1
GO_STEROID_HORMONE_RECEPTOR_BINDING	81	0.23	1.03	0.372	0.531	1
GO_VIRAL_GENOME_REPLICATION	21	0.32	1.03	0.377	0.532	1
GO_GDP_BINDING	50	0.25	1.03	0.364	0.535	1
GO_REGULATION_OF_STAT_CASCADE	144	0.21	1.03	0.374	0.538	1
GO_ERBB_SIGNALING_PATHWAY	79	0.23	1.03	0.391	0.538	1
GO_REGULATION_OF_TRANSCRIPTION_FACTOR_IMPORT_INTO_NUCLEU	94	0.23	1.03	0.37	0.538	1
GO_BASEMENT_MEMBRANE	92	0.22	1.03	0.381	0.539	1
GO_CELLULAR_METABOLIC_COMPOUND_SALVAGE	37	0.28	1.03	0.383	0.539	1
GO_U2_TYPE_PRESPLICEOSOME	16	0.34	1.03	0.389	0.539	1
GO_PHOSPHATIDIC_ACID_METABOLIC_PROCESS	32	0.29	1.03	0.394	0.541	1
GO_REGULATION_OF_ORGAN_FORMATION	32	0.29	1.03	0.415	0.542	1
GO_ESC_E_Z_COMPLEX	16	0.34	1.03	0.414	0.542	1
GO_POSITIVE_REGULATION_OF_MAP_KINASE_ACTIVITY	207	0.2	1.03	0.34	0.542	1
GO_ENDOTHELIAL_CELL_MIGRATION	57	0.25	1.03	0.419	0.541	1
GO_CELLULAR_RESPONSE_TO_NITRIC_OXIDE	15	0.34	1.03	0.407	0.541	1
GO_OXIDOREDUCTASE_ACTIVITY_ACTING_ON_A_HEME_GROUP_OF_DO	25	0.29	1.03	0.395	0.541	1
GO_RECEPTOR_ACTIVATOR_ACTIVITY	32	0.28	1.03	0.419	0.543	1
GO_POSITIVE_REGULATION_OF_PROTEIN_SERINE_THREONINE_KINASE_ACTIVITY	288	0.19	1.03	0.346	0.543	1
GO_EUKARYOTIC_TRANSLATION_INITIATION_FACTOR_3_COMPLEX	16	0.34	1.03	0.438	0.544	1
GO_POSITIVE_REGULATION_OF_REPRODUCTIVE_PROCESS	52	0.26	1.03	0.396	0.547	1
GO_CHONDROCYTE_DEVELOPMENT	21	0.31	1.03	0.401	0.547	1
GO_POSITIVE_REGULATION_OF_CELL_SUBSTRATE_ADHESION	98	0.22	1.03	0.403	0.548	1
GO_KERATAN_SULFATE_METABOLIC_PROCESS	33	0.28	1.03	0.387	0.548	1
GO_RESPONSE_TO_METAL_ION	333	0.19	1.03	0.364	0.548	1
GO_REGULATION_OF_REACTIVE_OXYGEN_SPECIES_BIOSYNTHETIC_PROCESS	64	0.24	1.02	0.397	0.548	1
GO_SKELETAL_SYSTEM_DEVELOPMENT	453	0.18	1.02	0.363	0.551	1
GO_FMN_BINDING	15	0.35	1.02	0.43	0.551	1
GO_MITOCHONDRIAL_ELECTRON_TRANSPORT_CYTOCHROME_C_TO_OX	16	0.34	1.02	0.438	0.551	1
GO_INTRINSIC_COMPONENT_OF_MITOCHONDRIAL_MEMBRANE	48	0.26	1.02	0.397	0.551	1
GO_POLYSOME	43	0.27	1.02	0.404	0.551	1
GO_KIDNEY_MESENCHYME_DEVELOPMENT	18	0.32	1.02	0.415	0.551	1
GO_REGULATION_OF_EPITHELIAL_CELL_DIFFERENTIATION	121	0.21	1.02	0.392	0.551	1
GO_INNER_EAR_MORPHOGENESIS	91	0.23	1.02	0.409	0.551	1
GO_H4_HISTONE_ACETYLTRANSFERASE_COMPLEX	18	0.33	1.02	0.402	0.552	1
GO_NEGATIVE_REGULATION_OF_CELLULAR_CATABOLIC_PROCESS	153	0.21	1.02	0.389	0.552	1
GO_RUFFLE	155	0.21	1.02	0.38	0.552	1
GO_NEGATIVE_REGULATION_OF_SIGNAL_TRANSDUCTION_IN_ABSENCE_OF_LIGAND	32	0.28	1.02	0.429	0.553	1
GO_PROTEIN_KINASE_C_SIGNALING	16	0.34	1.02	0.416	0.553	1

GO_SPROUTING_ANGIOGENESIS	45	0.26	1.02	0.43	0.554	1
GO_HYPEROSMOTIC_RESPONSE	20	0.32	1.02	0.423	0.554	1
GO_TETRAPYRROLE_METABOLIC_PROCESS	56	0.24	1.02	0.42	0.556	1
GO_DNA_TEMPLATED_TRANSCRIPTION_INITIATION	200	0.2	1.02	0.389	0.556	1
GO_GLYCOSYL_COMPOUND_CATABOLIC_PROCESS	42	0.26	1.02	0.422	0.558	1
GO_LIPID_DIGESTION	22	0.31	1.02	0.412	0.559	1
GO_LIGAND_DEPENDENT_NUCLEAR_RECEPTOR_TRANSCRIPTION_COACTIVATOR_ACTIVITY	52	0.25	1.02	0.398	0.559	1
GO_REGULATION_OF_EPITHELIAL_CELL_APOPTOTIC_PROCESS	58	0.25	1.02	0.383	0.559	1
GO_GLYCOPROTEIN_BINDING	101	0.22	1.02	0.407	0.559	1
GO_RESPONSE_TO_NITRIC_OXIDE	21	0.31	1.02	0.436	0.56	1
GO_ORGANELLE_DISASSEMBLY	176	0.2	1.02	0.398	0.562	1
GO_DYNEIN_BINDING	24	0.3	1.02	0.407	0.562	1
GO_CELLULAR_PROTEIN_COMPLEX_ASSEMBLY	339	0.19	1.02	0.395	0.565	1
GO_L_ASCORBIC_ACID_BINDING	21	0.31	1.02	0.445	0.565	1
GO_POSITIVE_REGULATION_OF_JUN_KINASE_ACTIVITY	63	0.24	1.01	0.424	0.567	1
GO_NOTOCHORD_DEVELOPMENT	18	0.33	1.01	0.44	0.567	1
GO_PEPTIDYL_ASPARAGINE_MODIFICATION	39	0.26	1.01	0.441	0.568	1
GO_NEGATIVE_REGULATION_OF_HEMATOPOIETIC_PROGENITOR_CELL_DIFFERENTIATION	23	0.3	1.01	0.425	0.568	1
GO_RAS_PROTEIN_SIGNAL_TRANSDUCTION	142	0.21	1.01	0.42	0.569	1
GO_REGULATION_OF_NOTCH_SIGNALING_PATHWAY	66	0.24	1.01	0.442	0.57	1
GO_ATPASE_REGULATOR_ACTIVITY	30	0.29	1.01	0.428	0.57	1
GO_ALCOHOL_CATABOLIC_PROCESS	58	0.24	1.01	0.417	0.573	1
GO_REGULATION_OF_INTRACELLULAR_PROTEIN_TRANSPORT	377	0.18	1.01	0.403	0.577	1
GO_MESODERM_DEVELOPMENT	114	0.21	1.01	0.433	0.577	1
GO_AMINOGLYCAN_BIOSYNTHETIC_PROCESS	107	0.22	1.01	0.442	0.578	1
GO_FATTY_ACID_BINDING	30	0.28	1.01	0.435	0.578	1
GO_SODIUM_INDEPENDENT_ORGANIC_ANION_TRANSMEMBRANE_TRANSPORTER_ACTIVITY	21	0.32	1.01	0.432	0.578	1
GO_NEGATIVE_REGULATION_OF_PROTEIN_LOCALIZATION_TO_CELL_PERIPHERY	20	0.31	1.01	0.44	0.579	1
GO_CELLULAR_RESPONSE_TO_ACID_CHEMICAL	172	0.2	1.01	0.436	0.579	1
GO_LACTATION	41	0.26	1.01	0.437	0.581	1
GO_PURINE_DEOXYRIBONUCLEOTIDE_METABOLIC_PROCESS	15	0.34	1.01	0.445	0.581	1
GO_POSITIVE_REGULATION_OF_MYOTUBE_DIFFERENTIATION	29	0.29	1.01	0.448	0.581	1
GO_REGULATION_OF_CYTOPLASMIC_TRANSPORT	475	0.18	1.01	0.432	0.583	1
GO_KINESIN_BINDING	33	0.27	1.01	0.456	0.585	1
GO_REGULATION_OF_DNA_DAMAGE_RESPONSE_SIGNAL_TRANSDUCTION_BY_P53_CLASS_MEDIATOR	28	0.29	1.01	0.428	0.584	1
GO_NEGATIVE_REGULATION_OF_CATABOLIC_PROCESS	199	0.2	1	0.433	0.585	1
GO_RUFFLE_MEMBRANE	80	0.23	1	0.434	0.585	1
GO_CELLULAR_RESPONSE_TO_OSMOTIC_STRESS	21	0.31	1	0.438	0.587	1
GO_NEGATIVE_REGULATION_OF_MEGAKARYOCYTE_DIFFERENTIATION	17	0.33	1	0.434	0.588	1
GO_REGULATION_OF_MYELOID_CELL_DIFFERENTIATION	178	0.2	1	0.455	0.59	1
GO_PROTEIN_TARGETING	400	0.18	1	0.462	0.591	1
GO_LIPID_TRANSPORTER_ACTIVITY	107	0.21	1	0.446	0.591	1
GO_NEGATIVE_REGULATION_OF_STRIATED_MUSCLE_CELL_DIFFERENTI	26	0.29	1	0.44	0.592	1
GO_CEREBRAL_CORTEX_DEVELOPMENT	104	0.22	1	0.438	0.593	1
GO_BASOLATERAL_PLASMA_MEMBRANE	211	0.19	1	0.442	0.593	1
GO_PROTEIN_TYROSINE_KINASE_ACTIVATOR_ACTIVITY	15	0.33	1	0.45	0.596	1
GO_PROTEIN_COMPLEX_LOCALIZATION	49	0.25	1	0.44	0.596	1
GO_REGULATION_OF_JNK_CASCADE	159	0.2	1	0.427	0.596	1
GO_REGULATION_OF_SMOOTH_MUSCLE_CELL_PROLIFERATION	98	0.22	1	0.441	0.596	1
GO_PROTEIN_ALKYLATION	112	0.21	1	0.42	0.6	1
GO_REGULATION_OF_MITOCHONDRIAL_MEMBRANE_POTENTIAL	54	0.25	1	0.469	0.602	1
GO_REGULATION_OF_STEM_CELL_PROLIFERATION	88	0.22	1	0.45	0.604	1
GO_NEGATIVE_REGULATION_OF_MAP_KINASE_ACTIVITY	73	0.23	1	0.44	0.604	1
GO_MALE_GENITALIA_DEVELOPMENT	21	0.31	1	0.468	0.604	1
GO_SMALL_MOLECULE_CATABOLIC_PROCESS	325	0.18	1	0.481	0.604	1
GO_EMBRYONIC_CRANIAL_SKELETON_MORPHOGENESIS	46	0.25	0.99	0.443	0.605	1
GO_CARBON_OXYGEN_LYASE_ACTIVITY	70	0.23	0.99	0.473	0.608	1
GO_REGULATION_OF_RESPONSE_TO_OXIDATIVE_STRESS	63	0.23	0.99	0.475	0.61	1
GO_GENERATION_OF_PRECURSOR_METABOLITES_AND_ENERGY	290	0.19	0.99	0.503	0.61	1
GO_COLUMNAR_CUBOIDAL_EPITHELIAL_CELL_DEVELOPMENT	47	0.25	0.99	0.464	0.612	1
GO_POSITIVE_REGULATION_OF_TRANSCRIPTION_FROM_RNA_POLYMERASE_II_PROMOTER_IN_RESPONSE_TO_STRESS	23	0.31	0.99	0.499	0.612	1
GO_OXIDATIVE_PHOSPHORYLATION	83	0.22	0.99	0.467	0.614	1
GO_PHOSPHATASE_BINDING	151	0.2	0.99	0.437	0.613	1
GO_OVULATION_CYCLE_PROCESS	88	0.22	0.99	0.493	0.614	1
GO_REGULATION_OF_MULTICELLULAR_ORGANISMAL_METABOLIC_PROC	38	0.26	0.99	0.476	0.613	1
GO_CELLULAR_TRANSITION_METAL_ION_HOMEOSTASIS	75	0.23	0.99	0.483	0.615	1
GO_VASCULATURE_DEVELOPMENT	468	0.18	0.99	0.491	0.619	1
GO_KERATAN_SULFATE_BIOSYNTHETIC_PROCESS	28	0.29	0.99	0.489	0.619	1
GO_REGULATION_OF_JUN_KINASE_ACTIVITY	81	0.22	0.99	0.513	0.622	1
GO_ORGANIC_HYDROXY_COMPOUND_CATABOLIC_PROCESS	72	0.22	0.99	0.487	0.622	1
GO_ANDROGEN_RECEPTOR_BINDING	39	0.26	0.99	0.507	0.623	1
GO_ANDROGEN_RECEPTOR_SIGNALING_PATHWAY	41	0.26	0.99	0.453	0.624	1
GO_SPERMATID_NUCLEUS_DIFFERENTIATION	18	0.32	0.99	0.491	0.624	1
GO_LOW_DENSITY_LIPOPROTEIN_PARTICLE_BINDING	15	0.33	0.99	0.481	0.624	1
GO_RNA_POLYMERASE_II_CORE_PROMOTER_SEQUENCE_SPECIFIC_DNA_BINDING	53	0.24	0.98	0.483	0.625	1
GO_HISTONE_METHYLATION	84	0.22	0.98	0.495	0.626	1
GO_HEMATOPOIETIC_PROGENITOR_CELL_DIFFERENTIATION	97	0.21	0.98	0.505	0.627	1
GO_BONE_MINERALIZATION	38	0.26	0.98	0.493	0.629	1
GO_CELLULAR_RESPONSE_TO_ALKALOID	34	0.27	0.98	0.482	0.628	1
GO_POSITIVE_REGULATION_OF_PROTEIN_COMPLEX_DISASSEMBLY	25	0.29	0.98	0.486	0.629	1

GO_RESPONSE_TO_IRON_ION	35	0.26	0.98	0.484	0.629	1
GO_HISTONE_LYSINE_N_METHYLTRANSFERASE_ACTIVITY	45	0.25	0.98	0.484	0.629	1
GO_REGULATION_OF_INTRINSIC_APOPTOTIC_SIGNALING_PATHWAY_IN_RESPONSE_TO_DNA_DAMAGE	34	0.27	0.98	0.464	0.629	1
GO_MLL1_2_COMPLEX	27	0.28	0.98	0.461	0.632	1
GO_ENDOLYSOSOME	16	0.33	0.98	0.486	0.632	1
GO_MACROAUTOPHAGY	265	0.18	0.98	0.512	0.633	1
GO_LABYRINTHINE_LAYER_BLOOD_VESSEL_DEVELOPMENT	18	0.31	0.98	0.461	0.633	1
GO_POSITIVE_REGULATION_OF_B_CELL_PROLIFERATION	37	0.26	0.98	0.489	0.634	1
GO_MULTI_ORGANISM_MEMBRANE_ORGANIZATION	30	0.28	0.98	0.487	0.634	1
GO_POSITIVE_REGULATION_OF_NEURON_APOPTOTIC_PROCESS	47	0.25	0.98	0.49	0.634	1
GO_CYSTEINE_TYPE_ENDOPEPTIDASE_REGULATOR_ACTIVITY_INVOLVED_IN_APOPTOTIC_PROCESS	42	0.25	0.98	0.463	0.634	1
GO_TUBE_MORPHOGENESIS	321	0.18	0.98	0.53	0.634	1
GO_PARTURITION	19	0.31	0.98	0.489	0.637	1
GO_NEGATIVE_REGULATION_OF_EPITHELIAL_CELL_APOPTOTIC_PROCE	34	0.27	0.98	0.455	0.637	1
GO_POSITIVE_REGULATION_OF_PHAGOCYTOSIS	45	0.25	0.98	0.476	0.637	1
GO_REGULATION_OF_PROTEIN_IMPORT_INTO_NUCLEUS_TRANSLOCATI	21	0.3	0.98	0.449	0.637	1
GO_ROUGH_ENDOPLASMIC_RETICULUM	71	0.23	0.98	0.489	0.638	1
GO_RETINOID_X_RECEPTOR_BINDING	17	0.32	0.98	0.469	0.637	1
GO_POSITIVE_REGULATION_OF_NITRIC_OXIDE_SYNTHASE_ACTIVITY	21	0.3	0.98	0.485	0.638	1
GO_ERBB2_SIGNALING_PATHWAY	39	0.26	0.98	0.494	0.639	1
GO_RESPONSE_TO_ETHANOL	135	0.2	0.98	0.52	0.639	1
GO_COLLAGEN_BINDING	65	0.23	0.98	0.48	0.64	1
GO_VIRION_ASSEMBLY	36	0.26	0.98	0.501	0.641	1
GO_CELLULAR_RESPIRATION	142	0.2	0.98	0.548	0.641	1
GO_STEROL_METABOLIC_PROCESS	121	0.21	0.98	0.522	0.642	1
GO_NEGATIVE_REGULATION_OF_RESPONSE_TO_CYTOKINE_STIMULUS	42	0.25	0.98	0.477	0.641	1
GO_REGULATION_OF_MRNA_SPLICING_VIA_SPLICEOSOME	49	0.25	0.97	0.482	0.645	1
GO_ORGANIC_HYDROXY_COMPOUND_BIOSYNTHETIC_PROCESS	173	0.19	0.97	0.538	0.644	1
GO_APICAL_PLASMA_MEMBRANE	287	0.18	0.97	0.526	0.647	1
GO_VASCULAR_ENDOTHELIAL_GROWTH_FACTOR_RECEPTOR_SIGNALING_PATHWAY	73	0.22	0.97	0.5	0.646	1
GO_MULTI_ORGANISM_ORGANELLE_ORGANIZATION	23	0.29	0.97	0.494	0.648	1
GO_LEUKOCYTE_PROLIFERATION	88	0.21	0.97	0.496	0.649	1
GO_POSITIVE_REGULATION_OF_MESENCHYMAL_CELL_PROLIFERATION	27	0.28	0.97	0.496	0.652	1
GO_PROTEIN_POLYMERIZATION	66	0.22	0.97	0.515	0.654	1
GO_PROTEIN_TYROSINE_PHOSPHATASE_ACTIVITY	103	0.21	0.97	0.519	0.654	1
GO_CELLULAR_RESPONSE_TO_ORGANIC_CYCLIC_COMPOUND	457	0.17	0.97	0.618	0.654	1
GO_NADP_BINDING	43	0.25	0.97	0.525	0.655	1
GO_PEPTIDASE_ACTIVATOR_ACTIVITY_INVOLVED_IN_APOPTOTIC_PROC	21	0.3	0.97	0.477	0.655	1
GO_GLYCOPROTEIN_CATABOLIC_PROCESS	15	0.33	0.97	0.506	0.655	1
GO_NUCLEOTIDE_TRANSPORT	25	0.29	0.97	0.481	0.655	1
GO_MITOCHONDRIAL_TRANSMEMBRANE_TRANSPORT	53	0.24	0.97	0.515	0.655	1
GO_AMINOGLYCAN_METABOLIC_PROCESS	164	0.19	0.97	0.562	0.656	1
GO_NEGATIVE_REGULATION_OF_CARBOHYDRATE_METABOLIC_PROCES	52	0.24	0.97	0.543	0.657	1
GO_MICROFILAMENT_MOTOR_ACTIVITY	21	0.3	0.97	0.513	0.658	1
GO_PURINERGIC_RECEPTOR_SIGNALING_PATHWAY	28	0.27	0.97	0.487	0.659	1
GO_THIOESTER_METABOLIC_PROCESS	83	0.21	0.97	0.542	0.66	1
GO_EPITHELIAL_TO_MESENCHYMAL_TRANSITION	56	0.23	0.97	0.528	0.66	1
GO_GLANDULAR_EPITHELIAL_CELL_DIFFERENTIATION	39	0.25	0.97	0.517	0.66	1
GO_T_CELL_PROLIFERATION	36	0.26	0.96	0.493	0.662	1
GO_GOLGI_ORGANIZATION	83	0.21	0.96	0.51	0.662	1
GO_PITUITARY_GLAND_DEVELOPMENT	42	0.24	0.96	0.521	0.662	1
GO_OUTER_MEMBRANE	188	0.19	0.96	0.57	0.662	1
GO_NEGATIVE_REGULATION_OF_INTERLEUKIN_6_PRODUCTION	34	0.27	0.96	0.52	0.661	1
GO_CELLULAR_RESPONSE_TO_LITHIUM_ION	17	0.32	0.96	0.502	0.661	1
GO_POSITIVE_REGULATION_OF_SECRETION	365	0.18	0.96	0.608	0.661	1
GO_TRANSCRIPTION_INITIATION_FROM_RNA_POLYMERASE_II_PROMOT	152	0.19	0.96	0.549	0.663	1
GO_REGULATION_OF_PROTEIN_TARGETING	303	0.18	0.96	0.586	0.664	1
GO_PROTEOGLYCAN_BINDING	30	0.27	0.96	0.523	0.664	1
GO_G_PROTEIN_COUPLED_RECEPTOR_BINDING	257	0.18	0.96	0.594	0.665	1
GO_REGULATION_OF_NITRIC_OXIDE_SYNTHASE_ACTIVITY	48	0.24	0.96	0.516	0.668	1
GO_POSITIVE_REGULATION_OF_PEPTIDYL_THREONINE_PHOSPHORYLAT	25	0.28	0.96	0.494	0.669	1
GO_MEMBRANE_MICRODOMAIN	285	0.18	0.96	0.586	0.669	1
GO_POSITIVE_REGULATION_OF_MRNA_3_END_PROCESSING	17	0.32	0.96	0.521	0.67	1
GO_OOGENESIS	65	0.23	0.96	0.542	0.67	1
GO_NAD_BINDING	53	0.23	0.96	0.55	0.67	1
GO_REGULATION_OF_PROTEIN_SECRETION	382	0.17	0.96	0.618	0.67	1
GO_NEGATIVE_REGULATION_OF_RESPONSE_TO_ENDOPLASMIC_RETICULUM_STRESS	39	0.25	0.96	0.507	0.67	1
GO_REGULATION_OF_GASTRULATION	35	0.26	0.96	0.529	0.67	1
GO_NEURON_FATE_SPECIFICATION	31	0.26	0.96	0.508	0.673	1
GO_APICOLATERAL_PLASMA_MEMBRANE	15	0.33	0.96	0.486	0.673	1
GO_CRANIAL_NERVE_MORPHOGENESIS	23	0.29	0.96	0.494	0.674	1
GO_INNER_EAR_RECEPTOR_STEREOCILIUM_ORGANIZATION	21	0.29	0.96	0.517	0.675	1
GO_REGULATION_OF_GLIAL_CELL_PROLIFERATION	19	0.3	0.96	0.529	0.676	1
GO_REGULATION_OF_PROTEIN_PHOSPHATASE_TYPE_2A_ACTIVITY	24	0.28	0.96	0.537	0.676	1
GO_EAR_MORPHOGENESIS	111	0.2	0.96	0.563	0.676	1
GO_B_CELL_DIFFERENTIATION	89	0.21	0.96	0.532	0.677	1
GO_MITOCHONDRIAL_MEMBRANE_PART	165	0.19	0.96	0.569	0.677	1
GO_POSITIVE_REGULATION_OF_GLIAL_CELL_DIFFERENTIATION	32	0.26	0.96	0.518	0.678	1
GO_PHOSPHATIDYLCHOLINE_METABOLIC_PROCESS	63	0.23	0.95	0.553	0.679	1
GO_CARBOHYDRATE_BINDING	269	0.18	0.95	0.596	0.679	1
GO_GASTRULATION	153	0.19	0.95	0.593	0.679	1
GO_CELLULAR_RESPONSE_TO_RETINOIC_ACID	65	0.23	0.95	0.546	0.679	1
GO_SODIUM_INDEPENDENT_ORGANIC_ANION_TRANSPORT	23	0.29	0.95	0.538	0.679	1

GO_NEGATIVE_REGULATION_OF_CHEMOTAXIS	50	0.24	0.95	0.531	0.68	1
GO_NEGATIVE_REGULATION_OF_MAPK_CASCADE	145	0.19	0.95	0.572	0.68	1
GO_CELLULAR_CARBOHYDRATE_METABOLIC_PROCESS	143	0.19	0.95	0.594	0.681	1
GO_CELLULAR_RESPONSE_TO_STARVATION	115	0.2	0.95	0.53	0.682	1
GO_RESPONSE_TO_COLD	43	0.24	0.95	0.556	0.682	1
GO_STEM_CELL_DIFFERENTIATION	190	0.19	0.95	0.625	0.684	1
GO_UBIQUITIN_LIKE_PROTEIN_LIGASE_ACTIVITY	194	0.18	0.95	0.582	0.684	1
GO_NEGATIVE_REGULATION_OF_INTRINSIC_APOPTOTIC_SIGNALING_PATHWAY_BY_P53_CLASS_MEDIATOR	18	0.3	0.95	0.522	0.685	1
GO_DIGESTIVE_SYSTEM_DEVELOPMENT	145	0.19	0.95	0.598	0.685	1
GO_POSITIVE_REGULATION_OF_CALCIUM_ION_TRANSPORT_INTO_CYTO	52	0.23	0.95	0.516	0.686	1
GO_POSITIVE_REGULATION_OF_LIPID_STORAGE	20	0.3	0.95	0.512	0.686	1
GO_POSITIVE_REGULATION_OF_EPITHELIAL_CELL_DIFFERENTIATION	57	0.23	0.95	0.548	0.686	1
GO_BASAL_TRANSCRIPTION_MACHINERY_BINDING	27	0.27	0.95	0.538	0.686	1
GO_NITROGEN_CYCLE_METABOLIC_PROCESS	15	0.32	0.95	0.536	0.686	1
GO_SKELETAL_MUSCLE_CONTRACTION	31	0.26	0.95	0.54	0.685	1
GO_MYELIN_SHEATH	163	0.19	0.95	0.576	0.687	1
GO_REGULATION_OF_PROTEIN_SUMOYLATION	20	0.3	0.95	0.514	0.687	1
GO_ACID_AMINO_ACID_LIGASE_ACTIVITY	21	0.29	0.95	0.545	0.687	1
GO_NEGATIVE_REGULATION_OF_CELLULAR_AMIDE_METABOLIC_PROCE	129	0.2	0.95	0.59	0.687	1
GO_APPENDAGE_DEVELOPMENT	168	0.19	0.95	0.593	0.687	1
GO_CELLULAR_RESPONSE_TO_STEROID_HORMONE_STIMULUS	213	0.18	0.95	0.628	0.687	1
GO_GLYCINE_METABOLIC_PROCESS	17	0.32	0.95	0.516	0.687	1
GO_VESICLE_TARGETING	74	0.22	0.95	0.555	0.687	1
GO_HORMONE_METABOLIC_PROCESS	164	0.19	0.95	0.618	0.689	1
GO_NEGATIVE_REGULATION_OF_INTRACELLULAR_SIGNAL_TRANSDUCTI	434	0.17	0.95	0.702	0.691	1
GO_LIPID_LOCALIZATION	262	0.18	0.95	0.635	0.69	1
GO_BASAL_LAMINA	21	0.29	0.95	0.536	0.692	1
GO_REGULATION_OF_PROTEIN_EXIT_FROM_ENDOPLASMIC_RETICULUM	19	0.3	0.95	0.535	0.694	1
GO_NEGATIVE_REGULATION_OF_CHONDROCYTE_DIFFERENTIATION	19	0.29	0.94	0.543	0.694	1
GO_PHOSPHOLIPID_TRANSPORTER_ACTIVITY	48	0.24	0.94	0.571	0.695	1
GO_MESODERM_MORPHOGENESIS	66	0.22	0.94	0.569	0.695	1
GO_AMEBOIDAL_TYPE_CELL_MIGRATION	152	0.19	0.94	0.599	0.695	1
GO_EXOCRINE_SYSTEM_DEVELOPMENT	45	0.24	0.94	0.574	0.696	1
GO_REGULATION_OF_LIPID_STORAGE	41	0.25	0.94	0.563	0.696	1
GO_REGULATION_OF_TELOMERE_CAPPING	22	0.29	0.94	0.524	0.696	1
GO_MITOCHONDRIAL_FUSION	19	0.3	0.94	0.538	0.696	1
GO_EPITHELIAL_CELL_MORPHOGENESIS	41	0.24	0.94	0.529	0.697	1
GO_OMEGA_PEPTIDASE_ACTIVITY	17	0.3	0.94	0.53	0.696	1
GO_SULFUR_COMPOUND_TRANSPORT	32	0.26	0.94	0.56	0.7	1
GO_REGULATION_OF_MAP_KINASE_ACTIVITY	318	0.17	0.94	0.65	0.701	1
GO_AXIS_ELONGATION	27	0.27	0.94	0.527	0.701	1
GO_DISRUPTION_OF_CELLS_OF_OTHER_ORGANISM	24	0.28	0.94	0.547	0.703	1
GO_REGULATION_OF_FIBROBLAST_MIGRATION	27	0.27	0.94	0.563	0.706	1
GO_REGULATION_OF_ACTIN_CYTOSKELETON_REORGANIZATION	32	0.26	0.94	0.562	0.711	1
GO_AMIDE_TRANSPORT	95	0.21	0.94	0.58	0.711	1
GO_SCAFFOLD_PROTEIN_BINDING	45	0.24	0.94	0.566	0.711	1
GO_POSITIVE_REGULATION_OF_CARDIAC_MUSCLE_TISSUE_DEVELOPM	28	0.27	0.94	0.547	0.711	1
GO_MULTICELLULAR_ORGANISM_GROWTH	74	0.21	0.94	0.602	0.712	1
GO_ODORANT_BINDING	82	0.21	0.94	0.571	0.712	1
GO_REGULATION_OF_MRNA_CATABOLIC_PROCESS	26	0.27	0.93	0.561	0.713	1
GO_MACROLIDE_BINDING	18	0.31	0.93	0.556	0.716	1
GO_INTRACELLULAR_STEROID_HORMONE_RECEPTOR_SIGNALING_PAT	68	0.22	0.93	0.575	0.716	1
GO_FATTY_ACID_BIOSYNTHETIC_PROCESS	108	0.2	0.93	0.615	0.719	1
GO_APICAL_JUNCTION_COMPLEX	124	0.2	0.93	0.611	0.719	1
GO_SH3_SH2_ADAPTOR_ACTIVITY	52	0.23	0.93	0.586	0.719	1
GO_TRABECULA_FORMATION	23	0.28	0.93	0.571	0.72	1
GO_NEGATIVE_REGULATION_OF_TOLL_LIKE_RECEPTOR_SIGNALING_PATHWAY	25	0.27	0.93	0.581	0.72	1
GO_GLUTAMATE_METABOLIC_PROCESS	28	0.27	0.93	0.568	0.722	1
GO_POSITIVE_REGULATION_OF_ATPASE_ACTIVITY	40	0.25	0.93	0.559	0.724	1
GO_CARBOHYDRATE_DERIVATIVE_CATABOLIC_PROCESS	174	0.18	0.93	0.668	0.725	1
GO_TRANSCRIPTION_COACTIVATOR_ACTIVITY	293	0.17	0.93	0.747	0.725	1
GO_CHROMATIN_DISASSEMBLY	17	0.31	0.93	0.558	0.726	1
GO_TETRAPYRROLE_BINDING	133	0.19	0.93	0.646	0.728	1
GO_ERYTHROCYTE_HOMEOSTASIS	73	0.21	0.93	0.641	0.728	1
GO_CUL4_RING_E3_UBIQUITIN_LIGASE_COMPLEX	25	0.27	0.93	0.554	0.729	1
GO_PROTEIN_MODIFICATION_BY_SMALL_PROTEIN_REMOVAL	114	0.2	0.93	0.67	0.729	1
GO_JNK_CASCADE	82	0.21	0.92	0.642	0.734	1
GO_POSITIVE_REGULATION_OF_GLIOGENESIS	46	0.23	0.92	0.572	0.736	1
GO_BRANCHING_INVOLVED_IN_SALIVARY_GLAND_MORPHOGENESIS	16	0.31	0.92	0.567	0.736	1
GO_PROTEIN_HOMOOLIGOMERIZATION	246	0.18	0.92	0.731	0.737	1
GO_SPERM_EGG_RECOGNITION	45	0.23	0.92	0.581	0.736	1
GO_FATTY_ACYL_COA_METABOLIC_PROCESS	51	0.23	0.92	0.611	0.738	1
GO_MYELOID_CELL_ACTIVATION_INVOLVED_IN_IMMUNE_RESPONSE	40	0.24	0.92	0.576	0.741	1
GO_REGULATION_OF_MESONEPHROS_DEVELOPMENT	26	0.27	0.92	0.571	0.742	1
GO_UTERUS_DEVELOPMENT	18	0.3	0.92	0.584	0.742	1
GO_CELLULAR_RESPONSE_TO_ANTIBIOTIC	16	0.31	0.92	0.566	0.743	1
GO_ACTIN_FILAMENT_BINDING	121	0.19	0.92	0.644	0.743	1
GO_THIOLESTER_HYDROLASE_ACTIVITY	33	0.25	0.92	0.595	0.744	1
GO_REGULATION_OF_CELL_MATRIX_ADHESION	89	0.2	0.92	0.636	0.745	1
GO_CARBOXY_LYASE_ACTIVITY	34	0.25	0.92	0.58	0.745	1
GO_MICROTUBULE_POLYMERIZATION	27	0.26	0.92	0.573	0.746	1
GO_GOLGI_STACK	120	0.19	0.92	0.658	0.748	1
GO_VESICLE_CYTOSKELETAL_TRAFFICKING	39	0.24	0.92	0.603	0.748	1
GO_LYSOSOMAL_LUMEN	87	0.21	0.92	0.647	0.748	1
GO_N_METHYLTRANSFERASE_ACTIVITY	77	0.21	0.92	0.636	0.748	1

GO_LIVER_REGENERATION	18	0.29	0.92	0.56	0.749	1
GO_CRANIAL_SKELETAL_SYSTEM_DEVELOPMENT	55	0.22	0.92	0.595	0.75	1
GO_PROTEIN_TYROSINE_SERINE_THREONINE_PHOSPHATASE_ACTIVITY	45	0.23	0.92	0.622	0.75	1
GO_GROWTH_FACTOR_BINDING	122	0.19	0.91	0.665	0.753	1
GO_CELLULAR_RESPONSE_TO_AMINO_ACID_STARVATION	25	0.27	0.91	0.597	0.754	1
GO_REGULATION_OF_HYDROGEN_PEROXIDE_INDUCED_CELL_DEATH	19	0.29	0.91	0.585	0.755	1
GO_SYNCYTIUM_FORMATION	25	0.27	0.91	0.578	0.755	1
GO_RESPONSE_TO_CAFFEINE	18	0.29	0.91	0.567	0.755	1
GO_EMBRYONIC_CAMERA_TYPE_EYE_DEVELOPMENT	35	0.24	0.91	0.623	0.755	1
GO_REGULATION_OF_MACROPHAGE_DIFFERENTIATION	20	0.29	0.91	0.57	0.755	1
GO_PROTEIN_K63_LINKED_DEUBIQUITINATION	23	0.27	0.91	0.59	0.755	1
GO_2_IRON_2_SULFUR_CLUSTER_BINDING	21	0.28	0.91	0.591	0.756	1
GO_CARBOHYDRATE_TRANSPORT	94	0.2	0.91	0.638	0.756	1
GO_ESTABLISHMENT_OF_ENDOTHELIAL_BARRIER	30	0.25	0.91	0.592	0.756	1
GO_DEMETHYLATION	54	0.22	0.91	0.626	0.756	1
GO_REGULATION_OF_ENDOTHELIAL_CELL_APOPTOTIC_PROCESS	41	0.24	0.91	0.603	0.756	1
GO_GAMMA_TUBULIN_BINDING	22	0.28	0.91	0.604	0.757	1
GO_STRESS_ACTIVATED_PROTEIN_KINASE_SIGNALING_CASCADE	103	0.19	0.91	0.636	0.757	1
GO_ALDEHYDE_DEHYDROGENASE_NAD_ACTIVITY	19	0.29	0.91	0.593	0.758	1
GO_POSITIVE_REGULATION_OF_SMOOTH_MUSCLE_CELL_MIGRATION	30	0.25	0.91	0.619	0.758	1
GO_REGULATION_OF_SMOOTH_MUSCLE_CELL_MIGRATION	49	0.23	0.91	0.63	0.757	1
GO_RESPONSE_TO_LIGHT_STIMULUS	278	0.17	0.91	0.768	0.758	1
GO_REGULATION_OF_DNA_TEMPLATED_TRANSCRIPTION_INITIATION	31	0.25	0.91	0.637	0.758	1
GO_ACTIN_FILAMENT_BUNDLE	52	0.23	0.91	0.631	0.758	1
GO_POST_TRANSLATIONAL_PROTEIN_MODIFICATION	35	0.24	0.91	0.602	0.76	1
GO_NEGATIVE_REGULATION_OF_EPITHELIAL_CELL_PROLIFERATION	115	0.19	0.91	0.702	0.763	1
GO_IRON_ION_BINDING	158	0.18	0.91	0.749	0.764	1
GO_REGULATION_OF_EXTRINSIC_APOPTOTIC_SIGNALING_PATHWAY_IN _ABSENCE_OF_LIGAND	46	0.23	0.91	0.62	0.764	1
GO_POLYOL_TRANSMEMBRANE_TRANSPORTER_ACTIVITY	15	0.31	0.91	0.581	0.766	1
GO_REGULATION_OF_FATTY_ACID_METABOLIC_PROCESS	85	0.2	0.91	0.662	0.767	1
GO_RESPONSE_TO_ATP	30	0.25	0.9	0.621	0.769	1
GO_CELL_FATE_COMMITMENT	227	0.17	0.9	0.788	0.771	1
GO_NEURAL_TUBE_DEVELOPMENT	148	0.18	0.9	0.714	0.771	1
GO_NEGATIVE_REGULATION_OF_MYELOID_LEUKOCYTE_DIFFERENTIATI	44	0.23	0.9	0.639	0.771	1
GO_MAINTENANCE_OF_LOCATION	135	0.19	0.9	0.715	0.771	1
GO_NEUTRAL_AMINO_ACID_TRANSPORT	34	0.25	0.9	0.615	0.774	1
GO_CRANIAL_NERVE_DEVELOPMENT	43	0.23	0.9	0.623	0.775	1
GO_MEMBRANE_PROTEIN_INTRACELLULAR_DOMAIN_PROTEOLYSIS	17	0.3	0.9	0.576	0.775	1
GO_ELECTRON_TRANSPORT_CHAIN	93	0.2	0.9	0.676	0.776	1
GO_CELLULAR_RESPONSE_TO_REACTIVE_NITROGEN_SPECIES	19	0.29	0.9	0.608	0.776	1
GO_PREASSEMBLY_OF_GPI_ANCHOR_IN_ER_MEMBRANE	15	0.3	0.9	0.57	0.779	1
GO_CELLULAR_RESPONSE_TO_INORGANIC_SUBSTANCE	156	0.18	0.9	0.725	0.779	1
GO_PROTEIN_LOCALIZATION_TO_ENDOPLASMIC_RETICULUM	123	0.18	0.9	0.738	0.781	1
GO_LOW_DENSITY_LIPOPROTEIN_PARTICLE_RECEPTOR_BINDING	17	0.29	0.9	0.616	0.786	1
GO_REGULATION_OF_CHOLESTEROL_EFFLUX	19	0.28	0.9	0.613	0.786	1
GO_PHOSPHOPROTEIN_BINDING	60	0.21	0.9	0.683	0.786	1
GO_ENDOPLASMIC_RETICULUM_GOLGI_INTERMEDIATE_COMPARTMENT _MEMBRANE	61	0.21	0.89	0.655	0.786	1
GO_POSITIVE_REGULATION_OF_ENDOTHELIAL_CELL_APOPTOTIC_PROC	15	0.29	0.89	0.624	0.786	1
GO_ORGANIC_HYDROXY_COMPOUND_METABOLIC_PROCESS	476	0.16	0.89	0.91	0.787	1
GO_NEGATIVE_REGULATION_OF_SIGNAL_TRANSDUCTION_BY_P53_CLAS S_MEDIATOR	27	0.25	0.89	0.609	0.787	1
GO_BONE_MORPHOGENESIS	79	0.2	0.89	0.697	0.789	1
GO_LYSINE_N_METHYLTRANSFERASE_ACTIVITY	52	0.22	0.89	0.654	0.792	1
GO_LIPOPROTEIN_PARTICLE_RECEPTOR_ACTIVITY	15	0.31	0.89	0.603	0.793	1
GO_POSITIVE_REGULATION_OF_TRIGLYCERIDE_METABOLIC_PROCESS	20	0.27	0.89	0.618	0.795	1
GO_TRIGLYCERIDE_RICH_LIPOPROTEIN_PARTICLE	19	0.28	0.89	0.597	0.796	1
GO_EMBRYONIC_HINDLIMB_MORPHOGENESIS	29	0.25	0.89	0.63	0.798	1
GO_AMINOGLYCAN_CATABOLIC_PROCESS	68	0.21	0.89	0.696	0.801	1
GO_NUCLEAR_SPECK	181	0.18	0.89	0.769	0.802	1
GO_POSITIVE_REGULATION_OF_NEURON_DEATH	67	0.21	0.88	0.692	0.806	1
GO_TRANSITION_METAL_ION_HOMEOSTASIS	104	0.19	0.88	0.751	0.807	1
GO_POSITIVE_REGULATION_OF_RELEASE_OF_SEQUESTERED_CALCIUM _ION_INTO_CYTOSOL	39	0.23	0.88	0.68	0.808	1
GO_MONOCARBOXYLIC_ACID_TRANSMEMBRANE_TRANSPORTER_ACTIVI	45	0.22	0.88	0.672	0.808	1
GO_HYDROLASE_ACTIVITY_ACTING_ON_GLYCOSYL_BONDS	117	0.19	0.88	0.755	0.808	1
GO_AUTOPHAGY	377	0.16	0.88	0.897	0.809	1
GO_REGULATION_OF_INTRINSIC_APOPTOTIC_SIGNALING_PATHWAY_BY _P53_CLASS_MEDIATOR	22	0.26	0.88	0.648	0.809	1
GO_RESPONSE_TO_LITHIUM_ION	27	0.25	0.88	0.644	0.81	1
GO_RESPONSE_TO_EXOGENOUS_DSRNA	44	0.23	0.88	0.656	0.809	1
GO_REGULATION_OF_CELL_MATURATION	18	0.28	0.88	0.627	0.809	1
GO_FATTY_ACID_METABOLIC_PROCESS	287	0.16	0.88	0.874	0.809	1
GO_GTP_METABOLIC_PROCESS	21	0.27	0.88	0.665	0.809	1
GO_NEGATIVE_REGULATION_OF_MYOTUBE_DIFFERENTIATION	18	0.29	0.88	0.625	0.809	1
GO_CELLULAR_EXTRAVASATION	25	0.26	0.88	0.646	0.81	1
GO_OVULATION_CYCLE	113	0.19	0.88	0.734	0.811	1
GO_NEURON_DEATH	46	0.22	0.88	0.691	0.812	1
GO_PALMITOYLTRANSFERASE_ACTIVITY	33	0.24	0.88	0.666	0.814	1
GO_TISSUE_REGENERATION	51	0.22	0.88	0.701	0.814	1
GO_NEGATIVE_REGULATION_OF_MITOCHONDRION_ORGANIZATION	39	0.23	0.88	0.697	0.815	1
GO_REGULATION_OF_PRI_MIRNA_TRANSCRIPTION_FROM_RNA_POLYME RASE_II_PROMOTER	17	0.29	0.88	0.627	0.816	1
GO_MAMMARY_GLAND_LOBULE_DEVELOPMENT	17	0.29	0.88	0.611	0.817	1
GO_OSTEOBLAST_DEVELOPMENT	18	0.28	0.88	0.654	0.818	1

GO_NUCLEAR_TRANSCRIBED_MRNA_CATABOLIC_PROCESS_NONSENSE_MEDIATED_DECAY	115	0.19	0.88	0.785	0.819	1
GO_HISTONE_DEMETHYLASE_ACTIVITY	26	0.25	0.87	0.674	0.821	1
GO_REGULATION_OF_TYROSINE_PHOSPHORYLATION_OF_STAT5_PROT	20	0.26	0.87	0.649	0.821	1
GO_CARBOHYDRATE_TRANSMEMBRANE_TRANSPORT	24	0.26	0.87	0.634	0.822	1
GO_FIBROBLAST_GROWTH_FACTOR_BINDING	23	0.26	0.87	0.632	0.822	1
GO_NEGATIVE_REGULATION_OF_ENDOPLASMIC_RETICULUM_STRESS_INDUCED_INTRINSIC_APOPTOTIC_SIGNALING_PATHWAY	19	0.27	0.87	0.663	0.822	1
GO_COPPER_ION_BINDING	52	0.22	0.87	0.702	0.822	1
GO_PEPTIDYL_LYSINE_TRIMETHYLATION	23	0.26	0.87	0.66	0.823	1
GO_PURINE_CONTAINING_COMPOUND_SALVAGE	16	0.28	0.87	0.628	0.823	1
GO_NEUTRAL_LIPID_BIOSYNTHETIC_PROCESS	28	0.25	0.87	0.647	0.823	1
GO_REGULATION_OF_ERAD_PATHWAY	27	0.26	0.87	0.654	0.822	1
GO_POSITIVE_REGULATION_OF_CALCIUM_ION_IMPORT	54	0.21	0.87	0.71	0.822	1
GO_REGULATION_OF_LIPID_BIOSYNTHETIC_PROCESS	126	0.18	0.87	0.796	0.822	1
GO_SOLUTE_PROTON_SYMPORTER_ACTIVITY	26	0.26	0.87	0.662	0.823	1
GO_ENDOSOME_LUMEN	26	0.25	0.87	0.656	0.823	1
GO_REGULATION_OF_HISTONE_METHYLATION	55	0.21	0.87	0.717	0.824	1
GO_POSITIVE_REGULATION_OF_NATURAL_KILLER_CELL_ACTIVATION	18	0.28	0.87	0.646	0.824	1
GO_INNER_MITOCHONDRIAL_MEMBRANE_PROTEIN_COMPLEX	101	0.19	0.87	0.777	0.824	1
GO_ENDOSOME_ORGANIZATION	59	0.21	0.87	0.729	0.826	1
GO_RENAL_SYSTEM_PROCESS_INVOLVED_IN_REGULATION_OF_SYSTEMIC_ARTERIAL_BLOOD_PRESSURE	23	0.26	0.87	0.668	0.826	1
GO_PROTEIN_K48_LINKED_UBIQUITINATION	46	0.22	0.87	0.699	0.829	1
GO_GROWTH	401	0.16	0.87	0.914	0.829	1
GO_NEGATIVE_REGULATION_OF_DNA_BIOSYNTHETIC_PROCESS	29	0.25	0.87	0.677	0.83	1
GO_CELLULAR_RESPONSE_TO_INTERLEUKIN_6	22	0.26	0.87	0.672	0.831	1
GO_MALE_GAMETE_GENERATION	461	0.15	0.87	0.956	0.833	1
GO_PEPTIDYL_LYSINE_METHYLATION	69	0.2	0.87	0.742	0.834	1
GO_SOLUTE_SODIUM_SYMPORTER_ACTIVITY	51	0.21	0.86	0.713	0.834	1
GO_ALPHA_TUBULIN_BINDING	24	0.26	0.86	0.661	0.834	1
GO_POSITIVE_REGULATION_OF_EPITHELIAL_CELL_APOPTOTIC_PROCES	24	0.26	0.86	0.689	0.833	1
GO_REGULATION_OF_MEMBRANE_LIPID_DISTRIBUTION	37	0.23	0.86	0.68	0.835	1
GO_REGULATION_OF_MRNA_3_END_PROCESSING	28	0.24	0.86	0.678	0.835	1
GO_TRIGLYCERIDE_LIPASE_ACTIVITY	20	0.27	0.86	0.67	0.836	1
GO_CORTICAL_ACTIN_CYTOSKELETON	57	0.21	0.86	0.721	0.836	1
GO_MAMMARY_GLAND_EPITHELIAL_CELL_DIFFERENTIATION	16	0.29	0.86	0.647	0.836	1
GO_LIPOSACCHARIDE_METABOLIC_PROCESS	113	0.18	0.86	0.793	0.837	1
GO_BILE_ACID_METABOLIC_PROCESS	35	0.23	0.86	0.688	0.838	1
GO_FAT_SOLUBLE_VITAMIN_METABOLIC_PROCESS	32	0.24	0.86	0.685	0.838	1
GO_PROTEINACEOUS_EXTRACELLULAR_MATRIX	349	0.16	0.86	0.908	0.838	1
GO_REGULATION_OF_CHROMATIN_ORGANIZATION	151	0.17	0.86	0.832	0.837	1
GO_CLATHRIN_COATED_ENDOCYTIC_VESICLE	63	0.21	0.86	0.734	0.837	1
GO_REGULATION_OF_STEROID_METABOLIC_PROCESS	72	0.2	0.86	0.78	0.837	1
GO_THIOESTER_BIOSYNTHETIC_PROCESS	54	0.21	0.86	0.747	0.837	1
GO_AMIDE_BINDING	263	0.16	0.86	0.885	0.838	1
GO_PRIMARY_LYSOSOME	16	0.29	0.86	0.661	0.838	1
GO_CELLULAR_RESPONSE_TO_KETONE	72	0.2	0.86	0.729	0.837	1
GO_OXIDOREDUCTASE_ACTIVITY_ACTING_ON_THE_CH_NH_GROUP_OF_DONORS	26	0.25	0.86	0.665	0.838	1
GO_VACUOLAR_LUMEN	112	0.19	0.86	0.794	0.839	1
GO_NUCLEAR_MEMBRANE_PART	15	0.29	0.86	0.641	0.841	1
GO_RETINA_HOMEOSTASIS	65	0.2	0.86	0.728	0.843	1
GO_ACTIVATION_OF_GTPASE_ACTIVITY	74	0.19	0.86	0.777	0.843	1
GO_EXOPEPTIDASE_ACTIVITY	103	0.18	0.86	0.813	0.843	1
GO_IRON_ION_TRANSPORT	55	0.21	0.86	0.754	0.845	1
GO_HINDLIMB_MORPHOGENESIS	37	0.22	0.86	0.725	0.845	1
GO_REGULATION_OF_B_CELL_DIFFERENTIATION	22	0.26	0.85	0.667	0.845	1
GO_NEGATIVE_REGULATION_OF_HISTONE_MODIFICATION	36	0.23	0.85	0.664	0.845	1
GO_TRANSCRIPTION_FACTOR_ACTIVITY_RNA_POLYMERASE_II_DISTAL_ENHANCER_SEQUENCE_SPECIFIC_BINDING	90	0.19	0.85	0.789	0.848	1
GO_PROTEIN_LOCALIZATION_TO_VACUOLE	44	0.22	0.85	0.75	0.848	1
GO_THIOL_DEPENDENT_UBIQUITIN_SPECIFIC_PROTEASE_ACTIVITY	73	0.19	0.85	0.797	0.849	1
GO_HISTONE_H3_ACETYLATION	43	0.22	0.85	0.745	0.851	1
GO_CORECEPTOR_ACTIVITY	38	0.22	0.85	0.717	0.852	1
GO_PROTEIN_TARGETING_TO_MITOCHONDRION	49	0.21	0.85	0.753	0.855	1
GO_RESPIRATORY_SYSTEM_DEVELOPMENT	194	0.17	0.85	0.884	0.855	1
GO_LIPASE_ACTIVITY	116	0.18	0.85	0.841	0.855	1
GO_ESTROGEN_RECEPTOR_BINDING	40	0.22	0.85	0.72	0.855	1
GO_REGULATION_OF_OLIGODENDROCYTE_DIFFERENTIATION	29	0.24	0.85	0.725	0.855	1
GO_REGULATION_OF_MITOCHONDRIAL_DEPOLARIZATION	18	0.27	0.85	0.682	0.855	1
GO_O_ACYLTRANSFERASE_ACTIVITY	48	0.21	0.85	0.742	0.859	1
GO_POSITIVE_REGULATION_OF_SMOOTH_MUSCLE_CONTRACTION	30	0.24	0.85	0.714	0.86	1
GO_COATED_VESICLE	232	0.16	0.84	0.922	0.86	1
GO_POSITIVE_REGULATION_OF_REACTIVE_OXYGEN_SPECIES_BIOSYNTHETIC_PROCESS	47	0.21	0.84	0.791	0.861	1
GO_NUCLEOPHAGY	17	0.27	0.84	0.669	0.861	1
GO_FLUID_TRANSPORT	27	0.25	0.84	0.701	0.861	1
GO_PHOSPHATIDYLINOSITOL_4_5_BISPHOSPHATE_BINDING	51	0.21	0.84	0.752	0.861	1
GO_REGULATION_OF_DNA_TEMPLATED_TRANSCRIPTION_ELONGATION	43	0.22	0.84	0.74	0.862	1
GO_MAP_KINASE_KINASE_KINASE_ACTIVITY	22	0.25	0.84	0.678	0.862	1
GO_INTRINSIC_COMPONENT_OF_ORGANELLE_MEMBRANE	272	0.16	0.84	0.951	0.862	1
GO_SULFUR_COMPOUND_BIOSYNTHETIC_PROCESS	201	0.16	0.84	0.904	0.862	1
GO_AMINE_CATABOLIC_PROCESS	21	0.26	0.84	0.714	0.863	1
GO_ESTABLISHMENT_OR_MAINTENANCE_OF_BIPOLAR_CELL_POLARITY	36	0.22	0.84	0.754	0.863	1
GO_HEPARIN_BINDING	155	0.17	0.84	0.896	0.863	1
GO_POSITIVE_REGULATION_OF_CHROMATIN_MODIFICATION	84	0.19	0.84	0.812	0.863	1

GO_REGULATION_OF_OSTEOCLAST_DIFFERENTIATION	62	0.2	0.84	0.746	0.864	1
GO_ENDOCRINE_PANCREAS_DEVELOPMENT	40	0.22	0.84	0.748	0.864	1
GO_MITOCHONDRION_MORPHOGENESIS	18	0.27	0.84	0.686	0.864	1
GO_MEMBRANE_BUDDING	111	0.18	0.84	0.857	0.864	1
GO_POLYOL_BIOSYNTHETIC_PROCESS	26	0.24	0.84	0.734	0.864	1
GO_CARBON_CARBON_LYASE_ACTIVITY	48	0.21	0.84	0.786	0.865	1
GO_RESPONSE_TO_FIBROBLAST_GROWTH_FACTOR	115	0.18	0.84	0.858	0.865	1
GO_MYELOID_LEUKOCYTE_MEDIATED_IMMUNITY	42	0.22	0.84	0.782	0.866	1
GO_MIDDLE_EAR_MORPHOGENESIS	20	0.26	0.84	0.722	0.866	1
GO_REGULATION_OF_PROTEIN_KINASE_B_SIGNALING	121	0.18	0.84	0.881	0.866	1
GO_REGULATION_OF_CARDIAC_MUSCLE_CELL_CONTRACTION	27	0.24	0.84	0.74	0.867	1
GO_CYTOSOLIC_LARGE_RIBOSOMAL_SUBUNIT	59	0.2	0.84	0.814	0.869	1
GO_PROTEIN_N_TERMINUS_BINDING	102	0.18	0.84	0.84	0.869	1
GO_POSITIVE_REGULATION_OF_MULTICELLULAR_ORGANISMAL_METABOLIC_PROCESS	23	0.25	0.84	0.733	0.869	1
GO_NEGATIVE_REGULATION_OF_ERBB_SIGNALING_PATHWAY	44	0.21	0.84	0.789	0.869	1
GO_ASTROCYTE_DEVELOPMENT	19	0.27	0.83	0.71	0.872	1
GO_TRANSCRIPTIONALLY_ACTIVE_CHROMATIN	19	0.26	0.83	0.709	0.872	1
GO_RESPONSE_TO_UV_B	16	0.27	0.83	0.7	0.873	1
GO_DETOXIFICATION	76	0.19	0.83	0.808	0.873	1
GO_LYASE_ACTIVITY	175	0.16	0.83	0.92	0.873	1
GO_REGIONALIZATION	310	0.15	0.83	0.969	0.874	1
GO_RESPONSE_TO_INTERLEUKIN_6	26	0.24	0.83	0.745	0.875	1
GO_ENDOCYTIC_VESICLE	251	0.16	0.83	0.967	0.876	1
GO_STEM_CELL_DIVISION	29	0.23	0.83	0.759	0.877	1
GO_OXIDOREDUCTASE_COMPLEX	93	0.18	0.83	0.814	0.877	1
GO_BODY_FLUID_SECRETION	71	0.19	0.83	0.799	0.877	1
GO_MAST_CELL_ACTIVATION	20	0.26	0.83	0.717	0.877	1
GO_GLOBAL_GENOME_NUCLEOTIDE_EXCISION_REPAIR	32	0.23	0.83	0.758	0.878	1
GO_NEGATIVE_REGULATION_OF_MUSCLE_CELL_DIFFERENTIATION	55	0.2	0.83	0.808	0.879	1
GO_POSITIVE_REGULATION_OF_FAT_CELL_DIFFERENTIATION	51	0.21	0.83	0.786	0.878	1
GO_MONOVALENT_INORGANIC_ANION_HOMEOSTASIS	19	0.26	0.83	0.694	0.88	1
GO_PURINE_RIBONUCLEOSIDE_BISPHOSPHATE_METABOLIC_PROCESS	20	0.26	0.83	0.731	0.88	1
GO_STEROID_HYDROXYLASE_ACTIVITY	31	0.23	0.82	0.765	0.882	1
GO_NEGATIVE_REGULATION_OF_INTRINSIC_APOPTOTIC_SIGNALING_PATHWAY_IN_RESPONSE_TO_DNA_DAMAGE	27	0.23	0.82	0.757	0.883	1
GO_NEGATIVE_REGULATION_OF_ENDOTHELIAL_CELL_APOPTOTIC_PRO	26	0.24	0.82	0.726	0.884	1
GO_LRR_DOMAIN_BINDING	17	0.27	0.82	0.751	0.884	1
GO_POSITIVE_REGULATION_OF_HISTONE_METHYLATION	32	0.22	0.82	0.773	0.885	1
GO_REGULATION_OF_GENE_EXPRESSION_BY_GENETIC_IMPRINTING	16	0.27	0.82	0.693	0.884	1
GO_REGULATION_OF_ACROSOME_REACTION	17	0.26	0.82	0.728	0.885	1
GO_NEGATIVE_REGULATION_OF_I_KAPPAB_KINASE_NF_KAPPAB_SIGNA	51	0.2	0.82	0.795	0.885	1
GO_REGULATION_OF_STEM_CELL_POPULATION_MAINTENANCE	17	0.27	0.82	0.723	0.885	1
GO_NEGATIVE_REGULATION_OF_KIDNEY_DEVELOPMENT	17	0.27	0.82	0.727	0.886	1
GO_SULFUR_COMPOUND_METABOLIC_PROCESS	354	0.15	0.82	0.965	0.886	1
GO_NUCLEOTIDE_EXCISION_REPAIR_PREINCISION_COMPLEX_STABILIZA	21	0.25	0.82	0.73	0.886	1
GO_POSITIVE_REGULATION_OF_STRIATED_MUSCLE_CELL_DIFFERENTIA	51	0.2	0.82	0.813	0.887	1
GO_GTP_RHO_BINDING	16	0.27	0.82	0.698	0.887	1
GO_REGULATION_OF_TRANSCRIPTION_ELONGATION_FROM_RNA_POLYMERASE_II_PROMOTER	24	0.24	0.82	0.741	0.89	1
GO_SENSORY_ORGAN_MORPHOGENESIS	238	0.16	0.82	0.946	0.89	1
GO_MANNOSYLTRANSFERASE_ACTIVITY	25	0.24	0.82	0.763	0.892	1
GO_MEMBRANE_LIPID_METABOLIC_PROCESS	174	0.16	0.81	0.938	0.893	1
GO_INACTIVATION_OF_MAPK_ACTIVITY	26	0.24	0.81	0.779	0.894	1
GO_HIPPO_SIGNALING	25	0.24	0.81	0.776	0.894	1
GO_UBIQUITIN_LIKE_PROTEIN_SPECIFIC_PROTEASE_ACTIVITY	99	0.18	0.81	0.87	0.894	1
GO_POSITIVE_REGULATION_OF_VASCULATURE_DEVELOPMENT	133	0.17	0.81	0.919	0.893	1
GO_RESPONSE_TO_PROSTAGLANDIN	34	0.22	0.81	0.795	0.894	1
GO_NEGATIVE_REGULATION_OF_DNA_BINDING	46	0.21	0.81	0.789	0.894	1
GO_INTRACELLULAR_CALCIUM_ACTIVATED_CHLORIDE_CHANNEL_ACTIVI	16	0.27	0.81	0.738	0.898	1
GO_RESPONSE_TO_STARVATION	152	0.17	0.81	0.93	0.898	1
GO_CLATHRIN_COATED_ENDOCYTIC_VESICLE_MEMBRANE	46	0.2	0.81	0.837	0.898	1
GO_CYTOPLASMIC_SEQUESTERING_OF_PROTEIN	39	0.21	0.81	0.821	0.898	1
GO_PROTEIN_DESTABILIZATION	34	0.22	0.81	0.765	0.898	1
GO_SIGNAL_TRANSDUCTION_INVOLVED_IN_REGULATION_OF_GENE_EXPRESSION	18	0.26	0.81	0.746	0.899	1
GO_POSITIVE_REGULATION_OF_EXTRINSIC_APOPTOTIC_SIGNALING_PATHWAY_VIA_DEATH_DOMAIN_RECEPTORS	16	0.26	0.81	0.726	0.901	1
GO_POSITIVE_REGULATION_OF_RNA_SPLICING	25	0.24	0.81	0.797	0.901	1
GO_ACROSOMAL_VESICLE	88	0.18	0.81	0.885	0.902	1
GO_HISTONE_UBIQUITINATION	34	0.22	0.81	0.788	0.901	1
GO_RESPONSE_TO_GROWTH_HORMONE	30	0.22	0.81	0.779	0.901	1
GO_RESPONSE_TO_CARBOHYDRATE	167	0.16	0.81	0.951	0.902	1
GO_NEGATIVE_REGULATION_OF_CELL_CYCLE_ARREST	20	0.25	0.81	0.769	0.902	1
GO_ENDOCYTOSIS	474	0.14	0.8	0.997	0.904	1
GO_NEGATIVE_REGULATION_OF_BIOMINERAL_TISSUE_DEVELOPMENT	19	0.25	0.8	0.734	0.905	1
GO_REGULATION_OF_PROTEIN_HOMODIMERIZATION_ACTIVITY	22	0.25	0.8	0.762	0.906	1
GO_POSITIVE_REGULATION_OF_GLYCOPROTEIN_METABOLIC_PROCESS	20	0.25	0.8	0.748	0.906	1
GO_VITAMIN_BINDING	80	0.18	0.8	0.89	0.909	1
GO_LIPOPROTEIN_PARTICLE_RECEPTOR_BINDING	22	0.24	0.8	0.772	0.91	1
GO_NEGATIVE_REGULATION_OF_MYELOID_CELL_DIFFERENTIATION	83	0.18	0.8	0.886	0.91	1
GO_NEGATIVE_REGULATION_OF_RNA_SPLICING	18	0.26	0.8	0.762	0.911	1
GO_RESPONSE_TO_STEROL	24	0.23	0.8	0.765	0.91	1
GO_TOR_SIGNALING	16	0.26	0.8	0.752	0.91	1
GO_POSITIVE_REGULATION_OF_LIPID_TRANSPORT	50	0.2	0.8	0.861	0.911	1
GO_POSITIVE_REGULATION_OF_DNA_TEMPLATED_TRANSCRIPTION_ELONGATION	22	0.24	0.8	0.745	0.911	1

GO_NEUROPILIN_BINDING	15	0.27	0.8	0.738	0.911	1
GO_AMINE_METABOLIC_PROCESS	130	0.17	0.8	0.919	0.912	1
GO_MUSCLE_ORGAN_MORPHOGENESIS	70	0.18	0.79	0.873	0.913	1
GO_SOMITOGENESIS	62	0.19	0.79	0.851	0.913	1
GO_GOLGI_ASSOCIATED_VESICLE_MEMBRANE	50	0.2	0.79	0.849	0.913	1
GO_BETA_CATENIN_TCF_COMPLEX_ASSEMBLY	42	0.2	0.79	0.842	0.913	1
GO_PROTEIN_LIPID_COMPLEX	39	0.21	0.79	0.838	0.913	1
GO_AUDITORY_RECEPTOR_CELL_DIFFERENTIATION	27	0.23	0.79	0.769	0.913	1
GO_MAGNESIUM_ION_TRANSMEMBRANE_TRANSPORT	16	0.26	0.79	0.758	0.913	1
GO_ESTABLISHMENT_OF_PROTEIN_LOCALIZATION_TO_ENDOPLASMIC_RETICULUM	104	0.17	0.79	0.904	0.913	1
GO_HYPOTHALAMUS_DEVELOPMENT	24	0.23	0.79	0.786	0.913	1
GO_REGULATION_OF_EPITHELIAL_CELL_DIFFERENTIATION_INVOLVED_IN_KIDNEY_DEVELOPMENT	15	0.27	0.79	0.751	0.913	1
GO_MITOGEN_ACTIVATED_PROTEIN_KINASE_KINASE_KINASE_BINDING	18	0.25	0.79	0.769	0.914	1
GO_CARTILAGE_DEVELOPMENT	147	0.16	0.79	0.942	0.915	1
GO_LUNG_EPITHELIUM_DEVELOPMENT	34	0.22	0.79	0.814	0.916	1
GO_POSITIVE_REGULATION_OF_MITOCHONDRIAL_MEMBRANE_PERMEABILITY	18	0.25	0.79	0.78	0.916	1
GO_ZINC_ION_HOMEOSTASIS	21	0.24	0.79	0.8	0.916	1
GO_SIN3_TYPE_COMPLEX	16	0.27	0.79	0.746	0.916	1
GO_NEGATIVE_REGULATION_OF_NEUROLOGICAL_SYSTEM_PROCESS	15	0.27	0.79	0.773	0.918	1
GO_LUNG_CELL_DIFFERENTIATION	25	0.23	0.79	0.809	0.918	1
GO_SOMATIC_STEM_CELL_POPULATION_MAINTENANCE	66	0.18	0.79	0.894	0.919	1
GO_PHOSPHATIDYLINOSITOL_BISPHOSPHATE_BINDING	73	0.18	0.79	0.884	0.919	1
GO_NEGATIVE_REGULATION_OF_CELL_JUNCTION_ASSEMBLY	20	0.24	0.79	0.779	0.919	1
GO_PHAGOCYTOSIS_ENGULFMENT	18	0.25	0.79	0.774	0.919	1
GO_FORELIMB_MORPHOGENESIS	40	0.2	0.78	0.828	0.92	1
GO_RENAL_SYSTEM_PROCESS_INVOLVED_IN_REGULATION_OF_BLOOD_VOLUME	17	0.26	0.78	0.771	0.921	1
GO_REGULATION_OF_CELL_SHAPE	136	0.16	0.78	0.947	0.922	1
GO_STEROL_HOMEOSTASIS	57	0.19	0.78	0.886	0.924	1
GO_NEGATIVE_REGULATION_OF_ADHERENS_JUNCTION_ORGANIZATION	16	0.26	0.78	0.785	0.924	1
GO_COLUMNAR_CUBOIDAL_EPITHELIAL_CELL_DIFFERENTIATION	109	0.17	0.78	0.935	0.924	1
GO_POSITIVE_REGULATION_OF_STEM_CELL_PROLIFERATION	61	0.19	0.78	0.886	0.924	1
GO_ANTERIOR_POSTERIOR_AXIS_SPECIFICATION	48	0.2	0.78	0.862	0.925	1
GO_METALLOEXOPEPTIDASE_ACTIVITY	51	0.19	0.78	0.88	0.928	1
GO_MYELOID_CELL_DEVELOPMENT	44	0.2	0.78	0.869	0.929	1
GO_SUBSTRATE_ADHESION_DEPENDENT_CELL_SPREADING	36	0.21	0.78	0.844	0.928	1
GO_DETECTION_OF_CHEMICAL_STIMULUS_INVOLVED_IN_SENSORY_PERCEPTION_OF_TASTE	44	0.2	0.78	0.874	0.928	1
GO_NEGATIVE_REGULATION_OF_TISSUE_REMODELING	17	0.25	0.77	0.799	0.929	1
GO_TRIGLYCERIDE_CATABOLIC_PROCESS	21	0.24	0.77	0.807	0.932	1
GO_MICROVILLUS	75	0.18	0.77	0.899	0.932	1
GO_TELOMERE_CAPPING	28	0.22	0.77	0.798	0.932	1
GO_LIPID_CATABOLIC_PROCESS	243	0.15	0.77	1	0.934	1
GO_COLLAGEN_TRIMER	86	0.17	0.77	0.926	0.934	1
GO_REGULATION_OF_PEPTIDYL_THREONINE_PHOSPHORYLATION	37	0.21	0.77	0.856	0.936	1
GO_OXIDOREDUCTASE_ACTIVITY_ACTING_ON_NAD_P_H_QUINONE_OR_SIMILAR_COMPOUND_AS_ACCEPTOR	51	0.19	0.77	0.883	0.937	1
GO_RESPONSE_TO_MINERALOCORTICOID	35	0.21	0.77	0.854	0.937	1
GO_POSITIVE_REGULATION_OF_PHOSPHOLIPID_METABOLIC_PROCESS	41	0.2	0.77	0.861	0.936	1
GO_POSITIVE_REGULATION_OF_CELL_CYCLE_G2_M_PHASE_TRANSITIO	17	0.25	0.76	0.795	0.938	1
GO_SOLUTE_CATION_SYMPORTER_ACTIVITY	99	0.16	0.76	0.943	0.938	1
GO_BLOOD_VESSEL_ENDOTHELIAL_CELL_MIGRATION	24	0.23	0.76	0.813	0.939	1
GO_POSITIVE_REGULATION_OF_LIPID_CATABOLIC_PROCESS	25	0.22	0.76	0.861	0.939	1
GO_REGULATION_OF_ACTIN_FILAMENT_BASED_MOVEMENT	32	0.21	0.76	0.834	0.94	1
GO_OXYGEN_BINDING	47	0.19	0.76	0.904	0.94	1
GO_MUCOPOLYSACCHARIDE_METABOLIC_PROCESS	106	0.17	0.76	0.943	0.94	1
GO_ZINC_II_ION_TRANSPORT	26	0.22	0.76	0.819	0.94	1
GO_POSITIVE_REGULATION_OF_FATTY_ACID_BIOSYNTHETIC_PROCESS	17	0.24	0.76	0.791	0.94	1
GO_CYTOSOLIC_RIBOSOME	109	0.16	0.76	0.959	0.941	1
GO_CELLULAR_LIPID_CATABOLIC_PROCESS	148	0.15	0.76	0.988	0.941	1
GO_COBALAMIN_METABOLIC_PROCESS	21	0.23	0.76	0.836	0.941	1
GO_ANATOMICAL_STRUCTURE_ARRANGEMENT	17	0.25	0.76	0.789	0.941	1
GO_RECEPTOR_SERINE_THREONINE_KINASE_BINDING	15	0.26	0.76	0.811	0.941	1
GO_DIGESTIVE_TRACT_MORPHOGENESIS	48	0.19	0.76	0.914	0.941	1
GO_PYRIDOXAL_PHOSPHATE_BINDING	51	0.19	0.76	0.907	0.941	1
GO_ORGANIC_ACID_TRANSMEMBRANE_TRANSPORTER_ACTIVITY	142	0.15	0.76	0.987	0.941	1
GO_RESPONSE_TO_LEAD_ION	20	0.23	0.76	0.803	0.941	1
GO_OXIDOREDUCTASE_ACTIVITY_ACTING_ON_THE_CH_NH2_GROUP_OF_DONORS	19	0.24	0.76	0.813	0.942	1
GO_EXCRETION	44	0.2	0.75	0.874	0.942	1
GO_REGULATION_OF_MITOCHONDRIAL_MEMBRANE_PERMEABILITY_INVOLVED_IN_APOPTOTIC_PROCESS	22	0.23	0.75	0.839	0.943	1
GO_G_PROTEIN_ALPHA_SUBUNIT_BINDING	22	0.23	0.75	0.81	0.943	1
GO_REGULATION_OF_HORMONE_BIOSYNTHETIC_PROCESS	18	0.24	0.75	0.836	0.943	1
GO_ORGANIC_ANION_TRANSMEMBRANE_TRANSPORTER_ACTIVITY	177	0.15	0.75	0.982	0.942	1
GO_NEURONAL_STEM_CELL_POPULATION_MAINTENANCE	19	0.24	0.75	0.845	0.942	1
GO_ESTROUS_CYCLE	19	0.24	0.75	0.822	0.945	1
GO_REGULATION_OF_MESENCHYMAL_CELL_PROLIFERATION	34	0.2	0.75	0.869	0.945	1
GO_NEGATIVE_REGULATION_OF_NOTCH_SIGNALING_PATHWAY	28	0.22	0.75	0.862	0.946	1
GO_POSITIVE_REGULATION_OF_CALCIUM_MEDIATED_SIGNALING	38	0.2	0.75	0.874	0.946	1
GO_POSITIVE_REGULATION_OF_PROTEIN_AUTOPHOSPHORYLATION	22	0.23	0.75	0.845	0.945	1
GO_LAMELLIPODIUM_ASSEMBLY	30	0.21	0.75	0.868	0.945	1
GO_NEGATIVE_REGULATION_OF_MUSCLE_CONTRACTION	22	0.23	0.75	0.846	0.947	1
GO_POSITIVE_REGULATION_OF_STEM_CELL_DIFFERENTIATION	50	0.18	0.75	0.912	0.948	1

GO_RHO_PROTEIN_SIGNAL_TRANSDUCTION	50	0.19	0.75	0.89	0.948	1
GO_RESPIRATORY_CHAIN	78	0.17	0.74	0.961	0.949	1
GO_CARDIAC_CHAMBER_MORPHOGENESIS	104	0.16	0.74	0.977	0.954	1
GO_SINGLE_ORGANISM_MEMBRANE_BUDDING	70	0.17	0.74	0.943	0.955	1
GO_BONE_DEVELOPMENT	155	0.15	0.74	0.997	0.955	1
GO_PROTEIN_MONOUBIQUITINATION	51	0.18	0.74	0.905	0.955	1
GO_SECRETORY_GRANULE_ORGANIZATION	27	0.21	0.73	0.886	0.957	1
GO_STRIATED_MUSCLE_CELL_PROLIFERATION	15	0.24	0.73	0.819	0.957	1
GO_REGULATION_OF_SULFUR_METABOLIC_PROCESS	20	0.23	0.73	0.839	0.957	1
GO_TRANSCRIPTION_FACTOR_TFIID_COMPLEX	23	0.22	0.73	0.857	0.96	1
GO_COATED_VESICLE_MEMBRANE	137	0.15	0.73	0.986	0.961	1
GO_ASPARTIC_TYPE_PEPTIDASE_ACTIVITY	25	0.21	0.73	0.877	0.961	1
GO_BLOOD_COAGULATION_FIBRIN_CLOT_FORMATION	24	0.22	0.73	0.816	0.961	1
GO_RESPONSE_TO_ACTIVITY	69	0.17	0.73	0.978	0.961	1
GO_MEMBRANE_LIPID_CATABOLIC_PROCESS	24	0.22	0.73	0.87	0.962	1
GO_REGULATION_OF_INSULIN_LIKE_GROWTH_FACTOR_RECEPTOR_SIGNALING_PATHWAY	22	0.22	0.73	0.879	0.962	1
GO_DNA_METHYLATION_INVOLVED_IN_GAMETE_GENERATION	18	0.23	0.72	0.845	0.963	1
GO_ACETYL_COA_METABOLIC_PROCESS	26	0.21	0.72	0.864	0.965	1
GO_RESPONSE_TO_FLUID_SHEAR_STRESS	34	0.2	0.72	0.901	0.965	1
GO_ARGININE_METABOLIC_PROCESS	17	0.23	0.72	0.864	0.965	1
GO_REGULATION_OF_KIDNEY_DEVELOPMENT	55	0.17	0.72	0.951	0.966	1
GO_FATTY_ACID_DERIVATIVE_TRANSPORT	20	0.22	0.72	0.884	0.969	1
GO_POSITIVE_REGULATION_OF_EPITHELIAL_TO_MESENCHYMAL_TRANSI	34	0.19	0.71	0.918	0.969	1
GO_NEGATIVE_REGULATION_OF_RESPONSE_TO_REACTIVE_OXYGEN_SPECIES	18	0.23	0.71	0.853	0.969	1
GO_CELLULAR_RESPONSE_TO_PROSTAGLANDIN_STIMULUS	24	0.21	0.71	0.877	0.969	1
GO_CELLULAR_MODIFIED_AMINO_ACID_CATABOLIC_PROCESS	17	0.23	0.71	0.872	0.97	1
GO_BLASTODERM_SEGMENTATION	15	0.24	0.71	0.845	0.97	1
GO_TRANSCRIPTION_COFACTOR_BINDING	24	0.21	0.71	0.88	0.969	1
GO_DIENCEPHALON_DEVELOPMENT	77	0.16	0.71	0.969	0.969	1
GO_TRICARBOXYLIC_ACID_METABOLIC_PROCESS	37	0.19	0.71	0.923	0.969	1
GO_REGULATION_OF_P38MAPK_CASCADE	26	0.2	0.71	0.919	0.971	1
GO_WATER_TRANSPORT	20	0.22	0.71	0.879	0.972	1
GO_SEGMENTATION	89	0.16	0.71	0.972	0.972	1
GO_NUCLEOSIDE_TRANSPORT	16	0.24	0.71	0.881	0.972	1
GO_NEGATIVE_REGULATION_OF_PHOSPHOPROTEIN_PHOSPHATASE_ACTIVITY	15	0.24	0.71	0.878	0.972	1
GO_EXON_EXON_JUNCTION_COMPLEX	22	0.21	0.71	0.899	0.972	1
GO_ENDOCRINE_SYSTEM_DEVELOPMENT	123	0.15	0.7	0.982	0.972	1
GO_MATURE_B_CELL_DIFFERENTIATION	17	0.23	0.7	0.871	0.972	1
GO_PROTEIN_TARGETING_TO_MEMBRANE	157	0.14	0.7	0.989	0.973	1
GO_RESPONSE_TO_HYDROPEROXIDE	15	0.24	0.7	0.885	0.973	1
GO_LENS_MORPHOGENESIS_IN_CAMERA_TYPE_EYE	19	0.22	0.7	0.907	0.973	1
GO_NITRIC_OXIDE_METABOLIC_PROCESS	15	0.24	0.7	0.861	0.972	1
GO_FACE_DEVELOPMENT	50	0.17	0.7	0.954	0.972	1
GO_POSITIVE_REGULATION_OF_ERYTHROCYTE_DIFFERENTIATION	23	0.21	0.7	0.9	0.972	1
GO_REGULATION_OF_SYSTEMIC_ARTERIAL_BLOOD_PRESSURE_BY_RENIN_ANGIOTENSIN	22	0.21	0.7	0.91	0.973	1
GO_POSITIVE_REGULATION_OF_KIDNEY_DEVELOPMENT	41	0.18	0.7	0.95	0.973	1
GO_MAINTENANCE_OF_CELL_NUMBER	132	0.15	0.7	0.992	0.973	1
GO_MUSCLE_CELL_MIGRATION	18	0.22	0.7	0.891	0.974	1
GO_NEURON_FATE_COMMITMENT	67	0.16	0.69	0.974	0.975	1
GO_VASODILATION	26	0.2	0.69	0.921	0.975	1
GO_REGULATION_OF_HEMATOPOIETIC_PROGENITOR_CELL_DIFFERENTIATION	38	0.18	0.69	0.937	0.975	1
GO_HEXOSAMINIDASE_ACTIVITY	15	0.23	0.69	0.88	0.975	1
GO_NEGATIVE_REGULATION_OF_LEUKOCYTE_MIGRATION	31	0.19	0.69	0.934	0.975	1
GO_ANION_HOMEOSTASIS	41	0.18	0.69	0.959	0.974	1
GO_VESICLE_LUMEN	102	0.15	0.69	0.995	0.974	1
GO_BONE_GROWTH	20	0.21	0.69	0.897	0.974	1
GO_COPI_COATED_VESICLE	23	0.2	0.69	0.911	0.975	1
GO_COPPER_ION_HOMEOSTASIS	16	0.23	0.69	0.886	0.975	1
GO_REGULATION_OF_CATENIN_IMPORT_INTO_NUCLEUS	27	0.2	0.69	0.931	0.978	1
GO_REGULATION_OF_LIPOPOLYSACCHARIDE_MEDIATED_SIGNALING_PATHWAY	18	0.21	0.68	0.894	0.978	1
GO_EXTRACELLULAR_MATRIX_STRUCTURAL_CONSTITUENT	74	0.16	0.68	0.988	0.978	1
GO_ENDOCYTIC_RECYCLING	24	0.2	0.68	0.905	0.978	1
GO_ICOSANOID_RECEPTOR_ACTIVITY	15	0.23	0.68	0.894	0.979	1
GO_NEGATIVE_REGULATION_OF_AXON_GUIDANCE	27	0.19	0.68	0.918	0.979	1
GO_BLOOD_VESSEL_REMODELING	32	0.19	0.68	0.937	0.978	1
GO_CARBOXY_TERMINAL_DOMAIN_PROTEIN_KINASE_COMPLEX	22	0.21	0.68	0.921	0.978	1
GO_PLASMA_LIPOPROTEIN_PARTICLE_CLEARANCE	21	0.21	0.68	0.907	0.978	1
GO_LEUKOCYTE_DEGRANULATION	28	0.19	0.68	0.918	0.978	1
GO_REGULATION_OF_TRANSCRIPTION_REGULATORY_REGION_DNA_BIN	36	0.18	0.68	0.945	0.978	1
GO_NUCLEOSIDE_BISPHOSPHATE_METABOLIC_PROCESS	37	0.18	0.67	0.963	0.981	1
GO_ESCRT_COMPLEX	25	0.2	0.67	0.946	0.98	1
GO_SUBSTRATE_DEPENDENT_CELL_MIGRATION	27	0.19	0.67	0.937	0.981	1
GO_REGULATION_OF_ERYTHROCYTE_DIFFERENTIATION	35	0.18	0.67	0.954	0.981	1
GO_LIPID_STORAGE	27	0.19	0.67	0.927	0.982	1
GO_ORGAN_INDUCTION	16	0.22	0.67	0.898	0.982	1
GO_PROTEIN_PHOSPHORYLATED_AMINO_ACID_BINDING	24	0.2	0.66	0.945	0.984	1
GO_PHOSPHATASE_COMPLEX	47	0.17	0.66	0.971	0.983	1
GO_METANEPHROS_MORPHOGENESIS	28	0.19	0.66	0.956	0.983	1
GO_CATION_SUGAR_SYMPORTER_ACTIVITY	15	0.22	0.66	0.923	0.984	1
GO_RNA_POLYMERASE_CORE_ENZYME_BINDING	22	0.2	0.66	0.914	0.984	1
GO_RESPONSE_TO_ZINC_ION	55	0.16	0.66	0.969	0.983	1

GO_HISTONE_H2A_UBIQUITINATION	16	0.22	0.66	0.93	0.984	1
GO_TRANS_GOLGI_NETWORK_TRANSPORT_VESICLE	27	0.19	0.66	0.95	0.985	1
GO_NUCLEAR_CYCLIN_DEPENDENT_PROTEIN_KINASE_HOLOENZYME_COMPLEX	15	0.22	0.65	0.898	0.986	1
GO_HISTONE_MONOUBIQUITINATION	23	0.2	0.65	0.947	0.987	1
GO_EMBRYONIC_EYE_MORPHOGENESIS	33	0.17	0.65	0.957	0.987	1
GO_REGULATION_OF_GLUCONEOGENESIS	37	0.17	0.64	0.969	0.989	1
GO_PHARYNGEAL_SYSTEM_DEVELOPMENT	18	0.2	0.64	0.95	0.99	1
GO_ENDOPLASMIC_RETICULUM_SUBCOMPARTMENT	16	0.21	0.63	0.934	0.992	1
GO_MONOSACCHARIDE_TRANSPORT	54	0.15	0.63	0.98	0.993	1
GO_RNA_POLYMERASE_BINDING	35	0.17	0.63	0.975	0.993	1
GO_REGULATION_OF_TRIGLYCERIDE_METABOLIC_PROCESS	32	0.17	0.63	0.982	0.993	1
GO_MRNA_TRANSCRIPTION	21	0.19	0.63	0.945	0.993	1
GO_REACTIVE_OXYGEN_SPECIES_BIOSYNTHETIC_PROCESS	23	0.19	0.63	0.944	0.993	1
GO_HEPARAN_SULFATE_PROTEOGLYCAN_BINDING	18	0.2	0.63	0.947	0.993	1
GO_NEGATIVE_REGULATION_OF_CALCIUM_ION_IMPORT	24	0.18	0.63	0.959	0.993	1
GO_AZOLE_TRANSPORT	15	0.21	0.63	0.938	0.993	1
GO_VENOUS_BLOOD_VESSEL_DEVELOPMENT	15	0.21	0.62	0.936	0.993	1
GO_LENS_DEVELOPMENT_IN_CAMERA_TYPE_EYE	66	0.15	0.62	0.998	0.993	1
GO_POSITIVE_REGULATION_OF_MESONEPHROS_DEVELOPMENT	22	0.19	0.62	0.939	0.993	1
GO_REGULATION_OF_CHONDROCYTE_DIFFERENTIATION	45	0.16	0.62	0.993	0.994	1
GO_REGULATION_OF_RHODOPSIN_MEDIATED_SIGNALING_PATHWAY	27	0.18	0.62	0.969	0.993	1
GO_ASPARTATE_FAMILY_AMINO_ACID_BIOSYNTHETIC_PROCESS	23	0.18	0.62	0.971	0.993	1
GO_NEGATIVE_REGULATION_OF_REGULATED_SECRETORY_PATHWAY	21	0.19	0.62	0.957	0.994	1
GO_AMINO_ACID_TRANSMEMBRANE_TRANSPORT	67	0.14	0.61	1	0.994	1
GO_VITAMIN_D_RECEPTOR_BINDING	17	0.2	0.61	0.962	0.994	1
GO_ACROSOMAL_MEMBRANE	22	0.19	0.61	0.958	0.994	1
GO_MACROPHAGE_ACTIVATION	31	0.17	0.61	0.965	0.994	1
GO_NOSE_DEVELOPMENT	15	0.21	0.61	0.942	0.994	1
GO_OXIDOREDUCTASE_ACTIVITY_ACTING_ON_PEROXIDE_AS_ACCEPTO	42	0.16	0.61	0.991	0.994	1
GO_POSITIVE_REGULATION_OF_EMBRYONIC_DEVELOPMENT	33	0.17	0.61	0.983	0.994	1
GO_HEAD_MORPHOGENESIS	36	0.16	0.6	0.988	0.995	1
GO_REGULATION_OF_HEART_MORPHOGENESIS	29	0.17	0.6	0.986	0.995	1
GO_SMOOTH_MUSCLE_TISSUE_DEVELOPMENT	18	0.19	0.6	0.966	0.995	1
GO_PEROXISOME_PROLIFERATOR_ACTIVATED_RECEPTOR_BINDING	15	0.2	0.6	0.95	0.995	1
GO_PLATELET_ALPHA_GRANULE_LUMEN	54	0.15	0.59	0.998	0.995	1
GO_GRANULOCYTE_DIFFERENTIATION	15	0.2	0.58	0.948	0.998	1
GO_AORTA_MORPHOGENESIS	22	0.17	0.57	0.983	0.999	1
GO_REGULATION_OF_MEGAKARYOCYTE_DIFFERENTIATION	25	0.17	0.57	0.975	0.999	1
GO_CELL_SURFACE_RECEPTOR_SIGNALING_PATHWAY_INVOLVED_IN_HEART_DEVELOPMENT	16	0.19	0.57	0.974	0.999	1
GO_CORE_PROMOTER_SEQUENCE_SPECIFIC_DNA_BINDING	101	0.12	0.56	1	0.999	1
GO_REGULATION_OF_HORMONE_METABOLIC_PROCESS	26	0.17	0.56	0.987	0.999	1
GO_PHAGOCYTIC_CUP	18	0.18	0.56	0.977	0.999	1
GO_NEUTRAL_LIPID_CATABOLIC_PROCESS	26	0.16	0.56	0.989	0.999	1
GO_MEGAKARYOCYTE_DIFFERENTIATION	20	0.17	0.55	0.982	1	1
GO_REGULATION_OF_PLASMA_LIPOPROTEIN_PARTICLE_LEVELS	45	0.14	0.55	0.995	1	1
GO_CYTOSOLIC_SMALL_RIBOSOMAL_SUBUNIT	42	0.14	0.54	0.993	1	1
GO_REGULATION_OF_PLATELET_ACTIVATION	31	0.15	0.53	0.996	1	1
GO_SUMO_TRANSFERASE_ACTIVITY	15	0.18	0.53	0.981	1	1
GO_EMBRYONIC_CAMERA_TYPE_EYE_MORPHOGENESIS	24	0.16	0.52	0.991	1	1
GO_RNA_DESTABILIZATION	16	0.17	0.51	0.995	1	1
GO_GLUTAMINE_FAMILY_AMINO_ACID_CATABOLIC_PROCESS	24	0.15	0.51	1	1	1
GO_PLATELET_DERIVED_GROWTH_FACTOR_RECEPTOR_BINDING	15	0.17	0.5	0.989	1	1
GO_SENSORY_PERCEPTION_OF_TASTE	65	0.12	0.48	1	1	1
GO_NEGATIVE_REGULATION_OF_LIPASE_ACTIVITY	15	0.16	0.48	0.998	1	1
GO_POSITIVE_REGULATION_OF_STEROL_TRANSPORT	17	0.16	0.48	0.996	1	1
GO_WNT_PROTEIN_BINDING	30	0.14	0.48	0.998	1	1
GO_MAST_CELL_GRANULE	20	0.15	0.48	0.995	1	1
GO_PHOSPHATE_ION_TRANSPORT	18	0.15	0.47	1	1	1
GO_MANNOSE_BINDING	18	0.14	0.42	0.998	1	1
GO_RESPONSE_TO_WATER	18	0.13	0.42	0.998	1	1
GO_PHOSPHATIDYLINOSITOL_3_KINASE_COMPLEX	18	0.13	0.42	1	1	1
GO_SOLUTE_PROTON_ANTIPORTER_ACTIVITY	17	0.13	0.41	1	1	1
GO_MAST_CELL_MEDIATED_IMMUNITY	17	0.13	0.41	1	1	1

Preranked gene set enrichment analysis (GSEA) results using the correlation levels of individual genes with TMB are shown. The number of genes in the Gene Ontology (SIZE) and other results are shown as output of GSEA. Enrichment score (ES) and normalized ES (NES) are shown with significance levels. The significance level of zero indicates < 0.001
