# Supplementary material for: Correlation-based and feature-driven mutation signature analyses to identify genetic features associated with DNA mutagenic processes in cancer genomes
Source: Genomics Inform. 2021 Dec 31;19(4):e40. doi: 10.5808/gi.21047 (PMC8752981; doi:10.5808/gi.21047)
Supplement: Supplemental Table 2. — Molecular functions enriched for low tumor mutation burden (TMB). Preranked gene set enrichment analysis results using the correlation levels of individual genes with TMB. Similarly shown with Supplementary Table 1. [file gi-21047suppl6.pdf]

Supplementary Table. 2. Molecular functions enriched for low tumor mutation burden (TMB)

Gene Ontology terms (MSigDB, c5)	SIZE	ES	NES	NOM p-val	FDR q-val	FWER p-va
GO_CATION_CHANNEL_COMPLEX	165	-0.54	-2.57	0	0	0
GO_REGULATION_OF_POSTSYNAPTIC_MEMBRANE_POTENTIAL	55	-0.64	-2.53	0	0	0
GO_SYNAPTIC_MEMBRANE	260	-0.5	-2.49	0	0	0
GO_VOLTAGE_GATED_CALCIUM_CHANNEL_COMPLEX	40	-0.66	-2.46	0	0	0
GO_MODULATION_OF_SYNAPTIC_TRANSMISSION	298	-0.48	-2.45	0	0	0
GO_POSTSYNAPSE	374	-0.47	-2.45	0	0	0
GO_EXCITATORY_SYNAPSE	196	-0.5	-2.42	0	0	0
GO_SYNAPTIC_SIGNALING	423	-0.46	-2.42	0	0	0
GO_MAIN_AXON	57	-0.61	-2.4	0	0	0
GO_NEURONAL_POSTSYNAPTIC_DENSITY	53	-0.61	-2.4	0	0	0
GO_POSTSYNAPTIC_MEMBRANE	204	-0.49	-2.39	0	0	0
GO_PRESYNAPTIC_ACTIVE_ZONE	29	-0.7	-2.38	0	0	0
GO_REGULATION_OF_SYNAPTIC_TRANSMISSION_GLUTAMATERGIC	50	-0.61	-2.38	0	0	0
GO_VOLTAGE_GATED_CATION_CHANNEL_ACTIVITY	133	-0.52	-2.38	0	0	0
GO_PRESYNAPSE	280	-0.47	-2.38	0	0	0
GO_POTASSIUM_CHANNEL_COMPLEX	90	-0.55	-2.37	0	0	0.001
GO_PRESYNAPTIC_MEMBRANE	55	-0.59	-2.37	0	0	0.001
GO_ACTION_POTENTIAL	94	-0.54	-2.36	0	0	0.001
GO_VOLTAGE_GATED_ION_CHANNEL_ACTIVITY	189	-0.48	-2.34	0	0	0.002
GO_GLUTAMATE_RECEPTOR_SIGNALING_PATHWAY	41	-0.63	-2.34	0	0	0.002
GO_VOLTAGE_GATED_CALCIUM_CHANNEL_ACTIVITY	42	-0.63	-2.34	0	0	0.002
GO_CALCIUM_CHANNEL_COMPLEX	60	-0.58	-2.33	0	0	0.002
GO_MEMBRANE_DEPOLARIZATION_DURING_ACTION_POTENTIAL	39	-0.64	-2.32	0	0	0.002
GO_EXOCYTIC_VESICLE_MEMBRANE	56	-0.58	-2.3	0	0	0.004
GO_GATED_CHANNEL_ACTIVITY	322	-0.45	-2.3	0	0	0.004
GO_TRANSPORTER_COMPLEX	318	-0.45	-2.29	0	0	0.005
GO_REGULATION_OF_NEUROTRANSMITTER_LEVELS	188	-0.47	-2.27	0	0	0.008
GO_CATION_CHANNEL_ACTIVITY	295	-0.44	-2.27	0	0	0.009
GO_REGULATION_OF_SYNAPSE_STRUCTURE_OR_ACTIVITY	230	-0.46	-2.26	0	0	0.009
GO_PRESYNAPTIC_PROCESS_INVOLVED_IN_SYNAPTIC_TRANSMISSION	115	-0.5	-2.26	0	0	0.009
GO_DENDRITE	445	-0.43	-2.25	0	0	0.01
GO_NEURONAL_CELL_BODY_MEMBRANE	20	-0.72	-2.25	0	0	0.01
GO_REGULATION_OF_DOPAMINE_SECRETION	22	-0.7	-2.24	0	0	0.01
GO_AXON_PART	216	-0.46	-2.23	0	0	0.011
GO_AXON	412	-0.43	-2.23	0	0	0.011
GO_REGULATION_OF_NEUROTRANSMITTER_TRANSPORT	62	-0.55	-2.23	0	0	0.011
GO_NEUROTRANSMITTER_TRANSPORT	154	-0.48	-2.22	0	0	0.013
GO_MEMBRANE_DEPOLARIZATION	61	-0.55	-2.22	0	0	0.014
GO_EXCITATORY_POSTSYNAPTIC_POTENTIAL	27	-0.66	-2.21	0	0	0.014
GO_MULTICELLULAR_ORGANISMAL_SIGNALING	123	-0.49	-2.2	0	0	0.014
GO_GLUTAMATE_RECEPTOR_ACTIVITY	27	-0.66	-2.2	0	0	0.014
GO_POTASSIUM_CHANNEL_ACTIVITY	119	-0.49	-2.2	0	0	0.017
GO_PERIKARYON	107	-0.49	-2.19	0	0	0.017
GO_NEURON_MATURATION	30	-0.63	-2.19	0	0	0.017
GO_TRANSMITTER_GATED_CHANNEL_ACTIVITY	26	-0.65	-2.18	0	0	0.018
GO_SYNAPSE_ORGANIZATION	145	-0.47	-2.18	0	0	0.018
GO_POTASSIUM_ION_TRANSPORT	153	-0.46	-2.18	0	0	0.018
GO_IONOTROPIC_GLUTAMATE_RECEPTOR_COMPLEX	46	-0.59	-2.18	0	0	0.018
GO_ADULT_BEHAVIOR	132	-0.48	-2.17	0	0	0.018
GO_REGULATION_OF_SYNAPTIC_PLASTICITY	139	-0.47	-2.17	0	0	0.018
GO_REGULATION_OF_NEUROTRANSMITTER_SECRETION	49	-0.56	-2.17	0	0	0.02
GO_AMPA_GLUTAMATE_RECEPTOR_COMPLEX	26	-0.66	-2.16	0	0	0.023
GO_POSITIVE_REGULATION_OF_SYNAPTIC_TRANSMISSION	110	-0.48	-2.16	0	0	0.023
GO_SITE_OF_POLARIZED_GROWTH	149	-0.46	-2.16	0	0	0.024
GO_LOCOMOTORY_BEHAVIOR	180	-0.45	-2.16	0	0	0.026
GO_VOLTAGE_GATED_SODIUM_CHANNEL_ACTIVITY	20	-0.69	-2.16	0	0	0.026
GO_REGULATION_OF_SYNAPTIC_VESICLE_TRANSPORT	29	-0.64	-2.15	0	0	0.027
GO_CILIARY_TIP	42	-0.58	-2.15	0	0	0.027
GO_NEURON_PROJECTION_TERMINUS	127	-0.47	-2.14	0	0	0.032
GO_IONOTROPIC_GLUTAMATE_RECEPTOR_SIGNALING_PATHWAY	24	-0.65	-2.13	0	0	0.037
GO_POSITIVE_REGULATION_OF_SYNAPSE_ASSEMBLY	60	-0.53	-2.13	0	0	0.037
GO_REGULATION_OF_MEMBRANE_POTENTIAL	343	-0.41	-2.13	0	0	0.037
GO_REGULATION_OF_SYNAPTIC_TRANSMISSION_GABAERGIC	29	-0.62	-2.13	0	0	0.038
GO_TRANSMISSION_OF_NERVE_IMPULSE	54	-0.54	-2.12	0	0.001	0.053
GO_NEURON_PROJECTION_MEMBRANE	36	-0.59	-2.12	0	0.001	0.057
GO_T_TUBULE	45	-0.57	-2.12	0	0.001	0.057
GO_REGULATION_OF_AMINE_TRANSPORT	71	-0.5	-2.1	0	0.001	0.081
GO_REGULATION_OF_POTASSIUM_ION_TRANSPORT	83	-0.49	-2.09	0	0.001	0.094
GO_CYCLIC_NUCLEOTIDE_PHOSPHODIESTERASE_ACTIVITY	25	-0.65	-2.07	0	0.001	0.123
GO_REGULATION_OF_NEUROTRANSMITTER_RECEPTOR_ACTIVITY	30	-0.61	-2.07	0	0.001	0.123
GO_VOLTAGE_GATED_POTASSIUM_CHANNEL_ACTIVITY	88	-0.48	-2.07	0	0.001	0.128
GO_REGULATION_OF_SYNAPSE_ORGANIZATION	112	-0.46	-2.06	0	0.001	0.135
GO_REGULATION_OF_CALCIUM_ION_TRANSMEMBRANE_TRANSPORTER _ACTIVITY	70	-0.5	-2.06	0	0.001	0.136
GO_MODULATION_OF_EXCITATORY_POSTSYNAPTIC_POTENTIAL	30	-0.59	-2.06	0.002	0.001	0.139
GO_PRIMARY_CILIUM	195	-0.42	-2.06	0	0.001	0.14
GO_REGULATION_OF_POTASSIUM_ION_TRANSMEMBRANE_TRANSPORT	61	-0.51	-2.06	0	0.001	0.142
GO_PROTEIN_LOCALIZATION_TO_CILIUM	25	-0.62	-2.06	0	0.001	0.151
GO_POSITIVE_REGULATION_OF_EXCITATORY_POSTSYNAPTIC_POTENTI	21	-0.65	-2.06	0	0.001	0.153
GO_REGULATION_OF_ALPHA_AMINO_3_HYDROXY_5_METHYL_4_ISOXAZ OLE_PROPIONATE_SELECTIVE_GLUTAMATE_RECEPTOR_ACTIVITY	19	-0.67	-2.05	0	0.002	0.156
GO_REGULATION_OF_VOLTAGE_GATED_CALCIUM_CHANNEL_ACTIVITY	25	-0.64	-2.05	0	0.002	0.158
GO_CILIUM_MORPHOGENESIS	190	-0.43	-2.05	0	0.002	0.186

GO_REGULATION_OF_CATECHOLAMINE_SECRETION	43	-0.54	-2.04	0	0.002	0.208
GO_REGULATION_OF_SYNAPSE_ASSEMBLY	78	-0.49	-2.04	0	0.002	0.212
GO_CARDIAC_CONDUCTION	82	-0.48	-2.03	0	0.002	0.218
GO_CARDIAC_MUSCLE_CELL_ACTION_POTENTIAL	37	-0.56	-2.03	0	0.002	0.229
GO_CELLULAR_COMPONENT_ASSEMBLY_INVOLVED_IN_MORPHOGENES	232	-0.41	-2.03	0	0.002	0.233
GO_COGNITION	249	-0.4	-2.03	0	0.002	0.234
GO_POSTSYNAPTIC_MEMBRANE_ORGANIZATION	25	-0.61	-2.03	0	0.002	0.235
GO_REGULATION_OF_DENDRITE_MORPHOGENESIS	74	-0.48	-2.02	0	0.002	0.253
GO_TRANSLATION_REPRESSOR_ACTIVITY	20	-0.66	-2.02	0	0.002	0.258
GO_REGULATION_OF_TRANSPORTER_ACTIVITY	197	-0.42	-2.02	0	0.002	0.259
GO_INTRACILIARY_TRANSPORT_PARTICLE	29	-0.6	-2.02	0	0.002	0.266
GO_HOMOPHILIC_CELL_ADHESION_VIA_PLASMA_MEMBRANE_ADHESIO N_MOLECULES	153	-0.43	-2.02	0	0.002	0.266
GO_REGULATION_OF_HEART_CONTRACTION	221	-0.41	-2.02	0	0.002	0.274
GO_PASSIVE_TRANSMEMBRANE_TRANSPORTER_ACTIVITY	457	-0.38	-2.01	0	0.002	0.294
GO_NEURON_SPINE	117	-0.44	-2.01	0	0.003	0.307
GO_TERMINAL_BOUTON	63	-0.49	-2.01	0	0.003	0.31
GO_PROTEIN_LOCALIZATION_TO_SYNAPSE	15	-0.69	-2	0	0.003	0.317
GO_EXTRACELLULAR_GLUTAMATE_GATED_ION_CHANNEL_ACTIVITY	20	-0.64	-2	0	0.003	0.336
GO_CILIUM_ORGANIZATION	173	-0.42	-1.99	0	0.003	0.355
GO_REGULATION_OF_NEURONAL_SYNAPTIC_PLASTICITY	48	-0.53	-1.99	0	0.003	0.355
GO_LIGAND_GATED_CHANNEL_ACTIVITY	141	-0.44	-1.99	0	0.003	0.361
GO_ADENYLATE_CYCLASE_INHIBITING_G_PROTEIN_COUPLED_RECEPT OR_SIGNALING_PATHWAY	68	-0.49	-1.99	0	0.003	0.362
GO_EXOCYTIC_VESICLE	140	-0.43	-1.99	0	0.003	0.365
GO_CAMP_BINDING	23	-0.6	-1.98	0.002	0.003	0.399
GO_CILIARY_PLASM	75	-0.47	-1.98	0	0.003	0.402
GO_EXTRACELLULAR_LIGAND_GATED_ION_CHANNEL_ACTIVITY	75	-0.48	-1.98	0	0.003	0.402
GO_NONMOTILE_PRIMARY_CILIUM_ASSEMBLY	22	-0.63	-1.98	0	0.003	0.406
GO_CELL_BODY	488	-0.37	-1.98	0	0.003	0.422
GO_LEARNING	131	-0.43	-1.98	0	0.004	0.429
GO_ACTIVATION_OF_PROTEIN_KINASE_A_ACTIVITY	17	-0.67	-1.98	0	0.003	0.429
GO_DELAYED_RECTIFIER_POTASSIUM_CHANNEL_ACTIVITY	36	-0.55	-1.98	0	0.004	0.434
GO_GABA_RECEPTOR_BINDING	15	-0.7	-1.97	0	0.004	0.441
GO_REGULATION_OF_ADENYLATE_CYCLASE_ACTIVITY	70	-0.48	-1.97	0	0.004	0.445
GO_CELLULAR_RESPONSE_TO_GLUCAGON_STIMULUS	38	-0.55	-1.97	0	0.004	0.448
GO_SYNAPTIC_VESICLE_CYCLE	88	-0.45	-1.97	0	0.004	0.482
GO_RECEPTOR_CLUSTERING	41	-0.53	-1.97	0	0.004	0.482
GO_SARCOLEMMA	125	-0.43	-1.96	0	0.004	0.498
GO_NEUROMUSCULAR_PROCESS_CONTROLLING_BALANCE	50	-0.5	-1.96	0	0.004	0.498
GO_NEUROMUSCULAR_PROCESS	96	-0.45	-1.96	0	0.004	0.512
GO_POSITIVE_REGULATION_OF_ADENYLATE_CYCLASE_ACTIVITY	48	-0.52	-1.96	0	0.004	0.513
GO_NEURON_NEURON_SYNAPTIC_TRANSMISSION	56	-0.5	-1.96	0	0.004	0.513
GO_SINGLE_ORGANISM_BEHAVIOR	381	-0.38	-1.96	0	0.004	0.519
GO_SECOND_MESSENGER_MEDIATED_SIGNALING	157	-0.41	-1.96	0	0.004	0.524
GO_AXONEME_ASSEMBLY	40	-0.53	-1.95	0	0.004	0.538
GO_POSITIVE_REGULATION_OF_DENDRITIC_SPINE_DEVELOPMENT	34	-0.54	-1.95	0.002	0.004	0.547
GO_REGULATION_OF_LYASE_ACTIVITY	86	-0.45	-1.95	0	0.005	0.563
GO_STARTLE_RESPONSE	25	-0.59	-1.95	0	0.005	0.573
GO_CALCIUM_ION_REGULATED_EXOCYTOSIS	80	-0.47	-1.94	0	0.005	0.582
GO_CALCIUM_ION_TRANSMEMBRANE_TRANSPORTER_ACTIVITY	127	-0.43	-1.94	0	0.005	0.583
GO_REGULATION_OF_CATION_CHANNEL_ACTIVITY	88	-0.45	-1.94	0	0.005	0.583
GO_CILIARY_PART	286	-0.38	-1.94	0	0.005	0.623
GO_REGULATION_OF_TRANSMEMBRANE_TRANSPORT	422	-0.37	-1.93	0	0.005	0.63
GO_CYCLIC_NUCLEOTIDE_CATABOLIC_PROCESS	17	-0.65	-1.93	0	0.005	0.643
GO_NONMOTILE_PRIMARY_CILIUM	130	-0.42	-1.93	0	0.005	0.646
GO_CELL_CELL_SIGNALING_INVOLVED_IN_CARDIAC_CONDUCTION	22	-0.61	-1.93	0	0.005	0.649
GO_ADENYLATE_CYCLASE_MODULATING_G_PROTEIN_COUPLED_RECE PTOR_SIGNALING_PATHWAY	145	-0.41	-1.93	0	0.005	0.655
GO_POSITIVE_REGULATION_OF_NEURON_PROJECTION_DEVELOPMENT	227	-0.39	-1.93	0	0.006	0.658
GO_REGULATION_OF_NEURON_PROJECTION_DEVELOPMENT	400	-0.37	-1.92	0	0.006	0.686
GO_ADULT_LOCOMOTORY_BEHAVIOR	79	-0.45	-1.92	0	0.006	0.695
GO_NEUROTRANSMITTER_RECEPTOR_ACTIVITY	68	-0.47	-1.92	0	0.006	0.705
GO_CENTRAL_NERVOUS_SYSTEM_NEURON_DEVELOPMENT	69	-0.47	-1.91	0	0.006	0.714
GO_NEGATIVE_REGULATION_OF_SYNAPTIC_TRANSMISSION	61	-0.47	-1.91	0	0.006	0.715
GO_DENDRITE_DEVELOPMENT	78	-0.46	-1.91	0	0.007	0.747
GO_REGULATION_OF_N_METHYL_D_ASPARTATE_SELECTIVE_GLUTAMA TE_RECEPTOR_ACTIVITY	15	-0.66	-1.91	0	0.007	0.747
GO_POSITIVE_REGULATION_OF_AMINE_TRANSPORT	32	-0.55	-1.9	0.002	0.007	0.763
GO_NEGATIVE_REGULATION_OF_ACTIN_FILAMENT_BUNDLE_ASSEMBLY	19	-0.61	-1.9	0.002	0.007	0.771
GO_WALKING_BEHAVIOR	31	-0.55	-1.9	0	0.007	0.773
GO_CILIARY_TRANSITION_ZONE	23	-0.59	-1.9	0	0.007	0.779
GO_STRIATED_MUSCLE_ADAPTATION	23	-0.58	-1.9	0.004	0.007	0.788
GO_POSITIVE_REGULATION_OF_LYASE_ACTIVITY	61	-0.47	-1.89	0	0.008	0.804
GO_CYCLIC_NUCLEOTIDE_MEDIATED_SIGNALING	46	-0.5	-1.89	0	0.008	0.807
GO_LONG_TERM_SYNAPTIC_POTENTIATION	39	-0.53	-1.89	0	0.008	0.811
GO_SIGNAL_RELEASE	170	-0.4	-1.89	0	0.008	0.829
GO_REGULATION_OF_RESPIRATORY_SYSTEM_PROCESS	16	-0.65	-1.89	0	0.008	0.833
GO_SYNAPTIC_VESICLE_LOCALIZATION	104	-0.42	-1.88	0	0.008	0.849
GO_CYCLIC_NUCLEOTIDE_BINDING	36	-0.52	-1.88	0	0.009	0.854
GO_SYNTAXIN_BINDING	91	-0.43	-1.88	0.002	0.009	0.857
GO_DENDRITIC_SHAFT	36	-0.53	-1.88	0	0.009	0.865
GO_CHANNEL_REGULATOR_ACTIVITY	129	-0.41	-1.88	0	0.009	0.871
GO_CELL_MORPHOGENESIS_INVOLVED_IN_NEURON_DIFFERENTIATION	363	-0.36	-1.88	0	0.009	0.874
GO_REGULATION_OF_NEUROTRANSMITTER_UPTAKE	15	-0.65	-1.88	0.002	0.009	0.876
GO_POSITIVE_REGULATION_OF_NEURON_DIFFERENTIATION	301	-0.37	-1.87	0	0.009	0.877
GO_NEURON_RECOGNITION	33	-0.54	-1.87	0	0.009	0.88

GO_SYNAPTIC_TRANSMISSION_GLUTAMATERGIC	22	-0.59	-1.87	0	0.009	0.88
GO_CILIUM	440	-0.35	-1.87	0	0.009	0.888
GO_NEURON_CELL_CELL_ADHESION	16	-0.64	-1.87	0.004	0.009	0.89
GO_REGULATION_OF_BLOOD_CIRCULATION	295	-0.37	-1.87	0	0.009	0.891
GO_NODE_OF_RANVIER	15	-0.65	-1.87	0	0.009	0.891
GO_EXPLORATION_BEHAVIOR	24	-0.57	-1.87	0	0.009	0.895
GO_EXCITATORY_EXTRACELLULAR_LIGAND_GATED_ION_CHANNEL_AC	55	-0.47	-1.87	0	0.009	0.895
GO_PHOSPHOLIPID_DEPHOSPHORYLATION	34	-0.53	-1.87	0.002	0.009	0.903
GO_REGULATION_OF_CAMP_METABOLIC_PROCESS	129	-0.4	-1.87	0	0.009	0.903
GO_PROTEIN_KINASE_A_BINDING	40	-0.51	-1.86	0	0.009	0.907
GO_METAL_ION_TRANSMEMBRANE_TRANSPORTER_ACTIVITY	414	-0.35	-1.86	0	0.01	0.911
GO_DENDRITE_MEMBRANE	20	-0.6	-1.86	0.002	0.01	0.912
GO_3_5_CYCLIC_AMP_PHOSPHODIESTERASE_ACTIVITY	15	-0.65	-1.86	0	0.01	0.913
GO_GLYCOPROTEIN_COMPLEX	21	-0.59	-1.86	0.002	0.01	0.917
GO_PROTEIN_TRANSPORT_ALONG_MICROTUBULE	26	-0.56	-1.86	0	0.01	0.93
GO_1_PHOSPHATIDYLINOSITOL_BINDING	19	-0.6	-1.85	0.006	0.011	0.943
GO_SODIUM_CHANNEL_ACTIVITY	37	-0.52	-1.85	0	0.011	0.944
GO_POSITIVE_REGULATION_OF_AXON_EXTENSION	36	-0.5	-1.85	0.003	0.011	0.946
GO_CELL_DIFFERENTIATION_IN_HINDBRAIN	20	-0.59	-1.85	0	0.011	0.947
GO_DENDRITE_MORPHOGENESIS	41	-0.51	-1.85	0	0.011	0.949
GO_REGULATION_OF_CARDIAC_CONDUCTION	66	-0.45	-1.84	0	0.011	0.951
GO_REGULATION_OF_SYNAPTIC_VESICLE_EXOCYTOSIS	19	-0.6	-1.84	0.004	0.011	0.953
GO_REGULATION_OF_RESPIRATORY_GASEOUS_EXCHANGE	23	-0.58	-1.84	0.004	0.011	0.955
GO_CYCLIC_NUCLEOTIDE_METABOLIC_PROCESS	56	-0.46	-1.84	0	0.012	0.959
GO_REGULATION_OF_CARDIAC_MUSCLE_CELL_MEMBRANE_REPOLARIZATION	21	-0.58	-1.82	0.004	0.013	0.977
GO_REGULATION_OF_HEART_RATE	86	-0.43	-1.82	0	0.014	0.981
GO_REGULATION_OF_MEMBRANE_REPOLARIZATION	30	-0.54	-1.82	0	0.014	0.981
GO_REGULATION_OF_LONG_TERM_SYNAPTIC_POTENTIATION	19	-0.59	-1.82	0.002	0.014	0.981
GO_RESPONSE_TO_GLUCAGON	48	-0.47	-1.82	0.003	0.015	0.985
GO_POSITIVE_REGULATION_OF_TRANSPORTER_ACTIVITY	75	-0.43	-1.82	0	0.015	0.985
GO_NEURON_PROJECTION_MORPHOGENESIS	398	-0.35	-1.81	0	0.015	0.986
GO_CILIUM_MOVEMENT	33	-0.52	-1.81	0	0.015	0.987
GO_ION_CHANNEL_BINDING	111	-0.41	-1.81	0	0.015	0.99
GO_POSITIVE_REGULATION_OF_DENDRITE_DEVELOPMENT	63	-0.44	-1.81	0	0.016	0.991
GO_CAMP_MEDIATED_SIGNALING	37	-0.49	-1.8	0	0.016	0.991
GO_POSITIVE_REGULATION_OF_POTASSIUM_ION_TRANSPORT	38	-0.49	-1.8	0.002	0.016	0.993
GO_NEUROPEPTIDE_RECEPTOR_ACTIVITY	44	-0.48	-1.8	0.002	0.017	0.993
GO_PHOSPHATIDYLINOSITOL_DEPHOSPHORYLATION	23	-0.56	-1.8	0	0.017	0.993
GO_SYNTAXIN_1_BINDING	18	-0.6	-1.8	0.009	0.017	0.993
GO_GLUTAMATE_SECRETION	28	-0.53	-1.8	0.004	0.017	0.993
GO_CENTRAL_NERVOUS_SYSTEM_NEURON_DIFFERENTIATION	164	-0.38	-1.79	0	0.019	0.995
GO_REGULATION_OF_CALCIUM_ION_DEPENDENT_EXOCYTOSIS	84	-0.41	-1.78	0	0.019	0.995
GO_G_PROTEIN_COUPLED_RECEPTOR_SIGNALING_PATHWAY_COUPLED_TO_CYCLIC_NUCLEOTIDE_SECOND_MESSENGER	172	-0.38	-1.78	0.002	0.019	0.995
GO_CELL_CELL_ADHESION_VIA_PLASMA_MEMBRANE_ADHESION_MOLECULES	204	-0.37	-1.78	0	0.019	0.996
GO_ACTIVATION_OF_ADENYLATE_CYCLASE_ACTIVITY	39	-0.48	-1.78	0.004	0.02	0.996
GO_REGULATION_OF_CYCLIC_NUCLEOTIDE_METABOLIC_PROCESS	155	-0.37	-1.77	0.002	0.021	0.998
GO_PHOSPHATIDYLINOSITOL_PHOSPHATE_PHOSPHATASE_ACTIVITY	29	-0.52	-1.77	0.004	0.022	0.998
GO_REGULATION_OF_DENDRITIC_SPINE_MORPHOGENESIS	30	-0.52	-1.77	0.002	0.022	0.998
GO_MUSCLE_SYSTEM_PROCESS	281	-0.35	-1.77	0	0.022	0.998
GO_REGULATION_OF_CARDIAC_MUSCLE_CONTRACTION_BY_REGULATION_OF_THE_RELEASE_OF_SEQUESTERED_CALCIUM_ION	19	-0.57	-1.77	0.005	0.022	0.998
GO_REGULATION_OF_RELEASE_OF_SEQUESTERED_CALCIUM_ION_INTO_CYTOSOL_BY_SARCOPLASMIC_RETICULUM	25	-0.55	-1.77	0.005	0.022	0.998
GO_GAMMA_AMINOBUTYRIC_ACID_SIGNALING_PATHWAY	23	-0.55	-1.76	0.006	0.022	0.998
GO_POSITIVE_REGULATION_OF_NERVOUS_SYSTEM_DEVELOPMENT	429	-0.34	-1.76	0	0.022	0.998
GO_CARDIAC_MUSCLE_CELL_CONTRACTION	29	-0.51	-1.76	0.002	0.022	0.999
GO_AXON_EXTENSION	36	-0.48	-1.76	0.004	0.023	0.999
GO_REGULATION_OF_DENDRITE_DEVELOPMENT	118	-0.39	-1.76	0	0.022	0.999
GO_I_BAND	120	-0.38	-1.76	0	0.023	1
GO_CELL_SURFACE_RECEPTOR_SIGNALING_PATHWAY_INVOLVED_IN_CELL_CELL_SIGNALING	71	-0.43	-1.76	0	0.023	1
GO_POSITIVE_REGULATION_OF_SYNAPTIC_TRANSMISSION_GLUTAMATERGIC	18	-0.59	-1.75	0.007	0.024	1
GO_NEUROPEPTIDE_SIGNALING_PATHWAY	98	-0.4	-1.75	0	0.024	1
GO_CAMP_METABOLIC_PROCESS	34	-0.5	-1.75	0.005	0.024	1
GO_BETA_AMYLOID_BINDING	34	-0.5	-1.75	0.004	0.024	1
GO_REGULATION_OF_CATION_TRANSMEMBRANE_TRANSPORT	207	-0.36	-1.75	0	0.024	1
GO_MUSCLE_CONTRACTION	232	-0.35	-1.75	0	0.024	1
GO_CGMP_METABOLIC_PROCESS	23	-0.54	-1.75	0.01	0.024	1
GO_MELANOCYTE_DIFFERENTIATION	19	-0.57	-1.75	0.005	0.024	1
GO_REGULATION_OF_DENDRITIC_SPINE_DEVELOPMENT	54	-0.45	-1.73	0.002	0.027	1
GO_NEGATIVE_REGULATION_OF_AMINE_TRANSPORT	25	-0.53	-1.73	0.004	0.028	1
GO_REGULATION_OF_RESPONSE_TO_FOOD	19	-0.58	-1.73	0.005	0.027	1
GO_ENSHEATHMENT_OF_NEURONS	91	-0.4	-1.73	0	0.028	1
GO_NEUROTRANSMITTER_UPTAKE	15	-0.6	-1.73	0.013	0.028	1
GO_NEGATIVE_REGULATION_OF_CYCLIC_NUCLEOTIDE_METABOLIC_PROCESS	42	-0.47	-1.73	0.002	0.028	1
GO_REGULATION_OF_ARF_PROTEIN_SIGNAL_TRANSDUCTION	16	-0.6	-1.73	0.002	0.028	1
GO_WW_DOMAIN_BINDING	31	-0.51	-1.73	0.008	0.028	1
GO_GLUTAMATE_RECEPTOR_BINDING	36	-0.48	-1.73	0.007	0.028	1
GO_REGULATION_OF_MICROTUBULE_BASED_MOVEMENT	18	-0.57	-1.72	0.016	0.03	1
GO_REGULATION_OF_AXONOGENESIS	166	-0.36	-1.72	0	0.03	1
GO_CELL_COMMUNICATION_INVOLVED_IN_CARDIAC_CONDUCTION	37	-0.48	-1.72	0.01	0.031	1
GO_CALCIUM_ION_TRANSMEMBRANE_TRANSPORT	157	-0.36	-1.71	0	0.032	1

GO_SODIUM_CHANNEL_COMPLEX	17	-0.57	-1.71	0.015	0.033	1
GO_CELL_COMMUNICATION_BY_ELECTRICAL_COUPLING	15	-0.59	-1.71	0.007	0.033	1
GO_POTASSIUM_CHANNEL_REGULATOR_ACTIVITY	45	-0.45	-1.71	0.007	0.034	1
GO_DIVALENT_INORGANIC_CATION_TRANSMEMBRANE_TRANSPORTER_ACTIVITY	166	-0.36	-1.71	0	0.034	1
GO_FOREBRAIN_CELL_MIGRATION	61	-0.42	-1.7	0.002	0.035	1
GO_REGULATION_OF_AMINO_ACID_TRANSPORT	25	-0.52	-1.7	0.008	0.035	1
GO_ARF_GUANYL_NUCLEOTIDE_EXCHANGE_FACTOR_ACTIVITY	24	-0.52	-1.7	0.011	0.036	1
GO_LONG_TERM_MEMORY	28	-0.5	-1.7	0.007	0.036	1
GO_RIBONUCLEOTIDE_CATABOLIC_PROCESS	28	-0.5	-1.7	0.011	0.036	1
GO_MEMORY	98	-0.39	-1.69	0	0.039	1
GO_AXONAL_FASCICULATION	20	-0.55	-1.69	0.011	0.039	1
GO_FIBROBLAST_GROWTH_FACTOR_RECEPTOR_BINDING	28	-0.5	-1.69	0.021	0.04	1
GO_RENAL_WATER_HOMEOSTASIS	34	-0.48	-1.69	0.004	0.039	1
GO_REGULATION_OF_HEART_RATE_BY_CARDIAC_CONDUCTION	30	-0.48	-1.68	0.005	0.04	1
GO_MOTILE_CILIUM	101	-0.38	-1.68	0	0.04	1
GO_CYTOSOLIC_TRANSPORT	210	-0.34	-1.68	0	0.04	1
GO_GUANYL_NUCLEOTIDE_EXCHANGE_FACTOR_ACTIVITY	297	-0.33	-1.68	0	0.04	1
GO_RETINA_MORPHOGENESIS_IN_CAMERA_TYPE_EYE	45	-0.45	-1.68	0.006	0.041	1
GO_POSITIVE_REGULATION_OF_CALCIUM_ION_DEPENDENT_EXOCYTOS	26	-0.51	-1.68	0.009	0.042	1
GO_SNARE_BINDING	124	-0.37	-1.68	0	0.042	1
GO_REGULATION_OF_SMOOTHENED_SIGNALING_PATHWAY	62	-0.41	-1.68	0	0.042	1
GO_CAMERA_TYPE_EYE_PHOTORECEPTOR_CELL_DIFFERENTIATION	15	-0.57	-1.67	0.01	0.042	1
GO_MUSCLE_HYPERTROPHY	28	-0.49	-1.67	0.014	0.044	1
GO_SARCOPLASM	67	-0.41	-1.67	0.002	0.044	1
GO_SYNAPTIC_TRANSMISSION_DOPAMINERGIC	17	-0.57	-1.67	0.018	0.044	1
GO_REGULATION_OF_LONG_TERM_NEURONAL_SYNAPTIC_PLASTICITY	24	-0.51	-1.67	0.014	0.044	1
GO_POSITIVE_REGULATION_OF_CAMP_METABOLIC_PROCESS	89	-0.39	-1.67	0	0.044	1
GO_PHENOL_CONTAINING_COMPOUND_METABOLIC_PROCESS	78	-0.4	-1.67	0.002	0.044	1
GO_POSITIVE_REGULATION_OF_CALCIUM_ION_TRANSMEMBRANE_TRANSPORTER_ACTIVITY	31	-0.48	-1.67	0.015	0.045	1
GO_CEREBELLAR_CORTEX_FORMATION	21	-0.53	-1.66	0.015	0.045	1
GO_CALCIUM_ION_TRANSPORT	220	-0.34	-1.66	0	0.046	1
GO_REGULATION_OF_BEHAVIOR	65	-0.41	-1.66	0.003	0.046	1
GO_REGULATION_OF_METAL_ION_TRANSPORT	322	-0.32	-1.66	0	0.047	1
GO_RESPONSE_TO_AUDITORY_STIMULUS	23	-0.51	-1.66	0.025	0.048	1
GO_REGULATION_OF_PROTEIN_KINASE_A_SIGNALING	17	-0.56	-1.66	0.023	0.048	1
GO_AXONAL_GROWTH_CONE	20	-0.53	-1.65	0.009	0.049	1
GO_SYNAPSE_ASSEMBLY	69	-0.41	-1.65	0.002	0.049	1
GO_POTASSIUM_ION_IMPORT	28	-0.49	-1.65	0.009	0.051	1
GO_REGULATION_OF_MITOCHONDRIAL_FISSION	17	-0.55	-1.64	0.022	0.053	1
GO_INOSITOL_PHOSPHATE_METABOLIC_PROCESS	56	-0.41	-1.64	0.005	0.054	1
GO_ALPHA_ACTININ_BINDING	21	-0.52	-1.64	0.022	0.055	1
GO_GABA_RECEPTOR_ACTIVITY	22	-0.52	-1.64	0.013	0.055	1
GO_TRANSPORT_VESICLE_MEMBRANE	149	-0.35	-1.64	0	0.056	1
GO_POSITIVE_REGULATION_OF_DENDRITE_MORPHOGENESIS	32	-0.47	-1.63	0.019	0.057	1
GO_POSITIVE_REGULATION_OF_CELL_PROJECTION_ORGANIZATION	293	-0.32	-1.63	0	0.057	1
GO_ALKALI_METAL_ION_BINDING	21	-0.52	-1.63	0.011	0.058	1
GO_VENTRAL_SPINAL_CORD_INTERNEURON_DIFFERENTIATION	17	-0.54	-1.63	0.031	0.059	1
GO_SMOOTHENED_SIGNALING_PATHWAY	70	-0.39	-1.63	0.012	0.059	1
GO_REGULATION_OF_CARDIAC_MUSCLE_CONTRACTION_BY_CALCIUM_ION_SIGNALING	23	-0.51	-1.62	0.016	0.062	1
GO_RETROGRADE_TRANSPORT_VESICLE_RECYCLING_WITHIN_GOLGI	23	-0.5	-1.62	0.009	0.063	1
GO_POSITIVE_REGULATION_OF_NEUROTRANSMITTER_TRANSPORT	16	-0.56	-1.62	0.021	0.063	1
GO_REGULATION_OF_ACTIN_FILAMENT_BUNDLE_ASSEMBLY	77	-0.38	-1.62	0.007	0.064	1
GO_CENTRAL_NERVOUS_SYSTEM_NEURON_AXONOGENESIS	26	-0.48	-1.61	0.014	0.065	1
GO_RETINA_DEVELOPMENT_IN_CAMERA_TYPE_EYE	131	-0.35	-1.61	0.002	0.065	1
GO_PHOTORECEPTOR_CONNECTING_CILIUM	30	-0.47	-1.61	0.018	0.065	1
GO_CALMODULIN_BINDING	177	-0.34	-1.61	0	0.065	1
GO_NEURONAL_ACTION_POTENTIAL	28	-0.48	-1.61	0.012	0.065	1
GO_ADRENERGIC_RECEPTOR_SIGNALING_PATHWAY	23	-0.51	-1.61	0.02	0.066	1
GO_CALCIUM_ION_REGULATED_EXOCYTOSIS_OF_NEUROTRANSMITTER	34	-0.45	-1.61	0.014	0.066	1
GO_AMINO_ACID_BETAINE_METABOLIC_PROCESS	18	-0.53	-1.61	0.024	0.066	1
GO_PHOTORECEPTOR_CELL_DIFFERENTIATION	50	-0.42	-1.61	0.006	0.067	1
GO_RNA_POLYMERASE_II_TRANSCRIPTION_COACTIVATOR_ACTIVITY	36	-0.45	-1.61	0.014	0.068	1
GO_PHOSPHORIC_DIESTER_HYDROLASE_ACTIVITY	89	-0.37	-1.6	0.002	0.068	1
GO_NEUROMUSCULAR_JUNCTION_DEVELOPMENT	36	-0.45	-1.6	0.004	0.068	1
GO_TRANSLATION_REGULATOR_ACTIVITY_NUCLEIC_ACID_BINDING	18	-0.53	-1.6	0.019	0.069	1
GO_TELENCEPHALON_GLIAL_CELL_MIGRATION	19	-0.53	-1.6	0.014	0.069	1
GO_EPITHELIAL_CILIUM_MOVEMENT	17	-0.53	-1.6	0.029	0.07	1
GO_INWARD_RECTIFIER_POTASSIUM_CHANNEL_ACTIVITY	21	-0.51	-1.6	0.021	0.07	1
GO_RAS_GUANYL_NUCLEOTIDE_EXCHANGE_FACTOR_ACTIVITY	225	-0.33	-1.6	0	0.07	1
GO_REGULATION_OF_CILIUM_ASSEMBLY	48	-0.42	-1.6	0.012	0.07	1
GO_POSITIVE_REGULATION_OF_CATION_CHANNEL_ACTIVITY	37	-0.44	-1.6	0.012	0.071	1
GO_SIALYLATION	21	-0.51	-1.6	0.025	0.071	1
GO_SODIUM_ION_TRANSMEMBRANE_TRANSPORT	89	-0.37	-1.59	0.003	0.072	1
GO_NEURON_MIGRATION	109	-0.35	-1.59	0.003	0.073	1
GO_REGULATION_OF_AUTOPHAGOSOME_ASSEMBLY	34	-0.45	-1.59	0.021	0.073	1
GO_CILIARY_MEMBRANE	77	-0.39	-1.59	0.007	0.074	1
GO_ADENYLATE_CYCLASE_ACTIVATING_G_PROTEIN_COUPLED_RECEPTOR_SIGNALING_PATHWAY	73	-0.38	-1.59	0.005	0.075	1
GO_RESPONSE_TO_MONOAMINE	35	-0.45	-1.59	0.021	0.075	1
GO_GABA_RECEPTOR_COMPLEX	18	-0.53	-1.59	0.026	0.076	1
GO_MONOVALENT_INORGANIC_CATION_TRANSPORT	429	-0.3	-1.58	0	0.079	1
GO_POSITIVE_REGULATION_OF_NUCLEOTIDE_METABOLIC_PROCESS	133	-0.34	-1.58	0.003	0.08	1
GO_MONOVALENT_INORGANIC_CATION_TRANSMEMBRANE_TRANSPORTER_ACTIVITY	360	-0.3	-1.58	0	0.08	1

GO_RENAL_SYSTEM_PROCESS	99	-0.36	-1.58	0.002	0.08	1
GO_CATECHOLAMINE_BINDING	16	-0.53	-1.57	0.024	0.081	1
GO_REGULATION_OF_NUCLEOTIDE_METABOLIC_PROCESS	211	-0.32	-1.57	0	0.081	1
GO_POSITIVE_REGULATION_OF_POTASSIUM_ION_TRANSMEMBRANE_TRANSPORT	27	-0.47	-1.57	0.03	0.082	1
GO_NEGATIVE_REGULATION_OF_STRESS_FIBER_ASSEMBLY	16	-0.54	-1.57	0.025	0.082	1
GO_FERROUS_IRON_BINDING	22	-0.49	-1.57	0.042	0.083	1
GO_PHOTORECEPTOR_CELL_MAINTENANCE	35	-0.44	-1.57	0.028	0.083	1
GO_INTRACILIARY_TRANSPORT_PARTICLE_B	19	-0.52	-1.57	0.027	0.084	1
GO_INSULIN_RECEPTOR_BINDING	32	-0.45	-1.57	0.015	0.084	1
GO_SCF_UBIQUITIN_LIGASE_COMPLEX	34	-0.44	-1.57	0.016	0.084	1
GO_NEUROTROPHIN_SIGNALING_PATHWAY	23	-0.49	-1.57	0.028	0.084	1
GO_POSITIVE_REGULATION_OF_CYCLIC_NUCLEOTIDE_METABOLIC_PROCESS	109	-0.35	-1.57	0.003	0.085	1
GO_NEURAL_RETINA_DEVELOPMENT	50	-0.41	-1.57	0.01	0.084	1
GO_REGULATION_OF_POTASSIUM_ION_TRANSMEMBRANE_TRANSPORTER_ACTIVITY	41	-0.43	-1.57	0.012	0.084	1
GO_SENSORY_PERCEPTION_OF_PAIN	75	-0.37	-1.56	0.005	0.084	1
GO_NEUROMUSCULAR_SYNAPTIC_TRANSMISSION	26	-0.48	-1.56	0.018	0.085	1
GO_MEMBRANE_BIOGENESIS	30	-0.45	-1.56	0.028	0.085	1
GO_CALCIUM_MEDIATED_SIGNALING_USING_INTRACELLULAR_CALCIUM_SOURCE	16	-0.54	-1.56	0.041	0.085	1
GO_VENTRICULAR_CARDIAC_MUSCLE_CELL_ACTION_POTENTIAL	15	-0.53	-1.56	0.034	0.085	1
GO_METENCEPHALON_DEVELOPMENT	99	-0.35	-1.56	0.002	0.085	1
GO_NEUROPEPTIDE_HORMONE_ACTIVITY	29	-0.46	-1.56	0.021	0.085	1
GO_CELL_PROJECTION_ASSEMBLY	248	-0.31	-1.56	0.002	0.088	1
GO_MYOFIBRIL_ASSEMBLY	48	-0.41	-1.56	0.014	0.089	1
GO_DENDRITE_CYTOPLASM	16	-0.53	-1.55	0.028	0.089	1
GO_POSITIVE_REGULATION_OF_AXONOGENESIS	68	-0.38	-1.55	0.009	0.089	1
GO_MULTI_ORGANISM_BEHAVIOR	75	-0.37	-1.55	0.003	0.089	1
GO_PDZ_DOMAIN_BINDING	90	-0.36	-1.55	0.008	0.09	1
GO_PEPTIDE_HORMONE_BINDING	36	-0.43	-1.55	0.021	0.09	1
GO_POSITIVE_REGULATION_OF_BEHAVIOR	26	-0.48	-1.55	0.024	0.091	1
GO_FOREBRAIN_NEURON_DEVELOPMENT	34	-0.43	-1.55	0.02	0.091	1
GO_REGULATION_OF_DELAYED_RECTIFIER_POTASSIUM_CHANNEL_ACT	18	-0.51	-1.55	0.052	0.092	1
GO_PHOTORECEPTOR_CELL_DEVELOPMENT	39	-0.42	-1.55	0.022	0.092	1
GO_NUCLEUS_LOCALIZATION	20	-0.5	-1.55	0.026	0.092	1
GO_ACROSOME_REACTION	17	-0.51	-1.55	0.042	0.092	1
GO_DEVELOPMENTAL_CELL_GROWTH	76	-0.36	-1.54	0.011	0.095	1
GO_MUSCLE_MYOSIN_COMPLEX	19	-0.5	-1.54	0.038	0.096	1
GO_ORGANELLE_MEMBRANE_FUSION	95	-0.35	-1.54	0.005	0.096	1
GO_NEUROMUSCULAR_JUNCTION	54	-0.39	-1.54	0.019	0.097	1
GO_SPINAL_CORD_PATTERNING	24	-0.47	-1.54	0.037	0.098	1
GO_REGULATION_OF_CIRCADIAN_SLEEP_WAKE_CYCLE	25	-0.48	-1.54	0.026	0.098	1
GO_REGULATION_OF_GLUTAMATE_SECRETION	15	-0.54	-1.54	0.039	0.097	1
GO_CALCIUM_MEDIATED_SIGNALING	88	-0.36	-1.54	0.008	0.098	1
GO_REGULATION_OF_CALCIUM_ION_TRANSMEMBRANE_TRANSPORT	116	-0.34	-1.53	0.003	0.099	1
GO_CILIARY_BASAL_BODY	76	-0.37	-1.53	0.017	0.1	1
GO_TRANSLATION_REGULATOR_ACTIVITY	35	-0.43	-1.53	0.02	0.102	1
GO_APICAL_DENDRITE	15	-0.54	-1.53	0.036	0.101	1
GO_REGULATION_OF_VESICLE_FUSION	60	-0.38	-1.53	0.018	0.102	1
GO_POTASSIUM_ION_BINDING	15	-0.53	-1.53	0.047	0.103	1
GO_REGULATION_OF_G_PROTEIN_COUPLED_RECEPTOR_PROTEIN_SIGNALING_PATHWAY	125	-0.33	-1.53	0.005	0.103	1
GO_INTRA_GOLGI_VESICLE_MEDIATED_TRANSPORT	46	-0.41	-1.53	0.017	0.103	1
GO_NEGATIVE_REGULATION_OF_ORGANIC_ACID_TRANSPORT	18	-0.5	-1.53	0.018	0.103	1
GO_POSITIVE_REGULATION_OF_BLOOD_PRESSURE	37	-0.42	-1.52	0.028	0.106	1
GO_SNARE_COMPLEX	53	-0.39	-1.52	0.008	0.11	1
GO_NEGATIVE_REGULATION_OF_CATECHOLAMINE_SECRETION	16	-0.53	-1.52	0.045	0.11	1
GO_ANATOMICAL_STRUCTURE_MATURATION	39	-0.41	-1.52	0.02	0.111	1
GO_CONTRACTILE_FIBER	209	-0.31	-1.51	0.002	0.112	1
GO_MYELIN_ASSEMBLY	17	-0.5	-1.51	0.051	0.113	1
GO_NEGATIVE_REGULATION_OF_NUCLEOTIDE_METABOLIC_PROCESS	63	-0.37	-1.51	0.026	0.115	1
GO_CALCIUM_DEPENDENT_CELL_CELL_ADHESION_VIA_PLASMA_MEMBRANE_CELL_ADHESION_MOLECULES	27	-0.45	-1.51	0.029	0.115	1
GO_POSITIVE_REGULATION_OF_ION_TRANSPORT	234	-0.3	-1.51	0	0.114	1
GO_NEGATIVE_REGULATION_OF_NEUROTRANSMITTER_TRANSPORT	15	-0.53	-1.51	0.053	0.115	1
GO_NEGATIVE_REGULATION_OF_SMOOTHENED_SIGNALING_PATHWAY	25	-0.47	-1.51	0.042	0.115	1
GO_PEROXISOMAL_TRANSPORT	18	-0.5	-1.51	0.055	0.115	1
GO_GANGLIOSIDE_BIOSYNTHETIC_PROCESS	18	-0.49	-1.51	0.051	0.116	1
GO_NEGATIVE_REGULATION_OF_PEPTIDE_SECRETION	49	-0.38	-1.5	0.021	0.117	1
GO_CALCIUM_CHANNEL_REGULATOR_ACTIVITY	37	-0.41	-1.5	0.04	0.117	1
GO_PROTEIN_LOCALIZATION_TO_GOLGI_APPARATUS	32	-0.42	-1.5	0.041	0.117	1
GO_GLIAL_CELL_PROJECTION	15	-0.52	-1.5	0.046	0.117	1
GO_NEGATIVE_REGULATION_OF_CALCIUM_ION_TRANSMEMBRANE_TRANSPORT	29	-0.43	-1.5	0.045	0.117	1
GO_MICROTUBULE_BUNDLE_FORMATION	64	-0.37	-1.5	0.009	0.118	1
GO_INTRASPECIES_INTERACTION_BETWEEN_ORGANISMS	46	-0.4	-1.5	0.027	0.118	1
GO_DIVALENT_INORGANIC_CATION_TRANSPORT	265	-0.3	-1.5	0.005	0.118	1
GO_CEREBELLAR_PURKINJE_CELL_LAYER_DEVELOPMENT	24	-0.45	-1.5	0.047	0.119	1
GO_PEROXISOME_ORGANIZATION	32	-0.43	-1.5	0.031	0.122	1
GO_PHOSPHATIDYLINOSITOL_3_PHOSPHATE_BIOSYNTHETIC_PROCESS	49	-0.39	-1.49	0.029	0.123	1
GO_CALCIUM_ACTIVATED_POTASSIUM_CHANNEL_ACTIVITY	17	-0.5	-1.49	0.052	0.124	1
GO_LYSOSOMAL_TRANSPORT	67	-0.37	-1.49	0.017	0.125	1
GO_HEART_PROCESS	84	-0.35	-1.49	0.013	0.125	1
GO_PROTEIN_KINASE_A_REGULATORY_SUBUNIT_BINDING	16	-0.5	-1.49	0.056	0.125	1

GO_POSITIVE_REGULATION_OF_G_PROTEIN_COUPLED_RECEPTOR_PROTEIN_SIGNALING_PATHWAY	25	-0.45	-1.49	0.045	0.125	1
GO_CARDIAC_MYOFIBRIL_ASSEMBLY	16	-0.51	-1.49	0.053	0.125	1
GO_REFLEX	20	-0.49	-1.49	0.044	0.125	1
GO_EXOCYTOSIS	299	-0.29	-1.49	0.001	0.128	1
GO_AXONEMAL_DYNEIN_COMPLEX_ASSEMBLY	19	-0.48	-1.49	0.057	0.128	1
GO_SARCOPLASMIC_RETICULUM_MEMBRANE	37	-0.41	-1.48	0.031	0.129	1
GO_REGULATION_OF_CELL_MORPHOGENESIS_INVOLVED_IN_DIFFERENTIATION	335	-0.29	-1.48	0	0.129	1
GO_MUSCLE_FIBER_DEVELOPMENT	47	-0.39	-1.48	0.026	0.128	1
GO_TRANSMEMBRANE_RECEPTOR_PROTEIN_SERINE_THREONINE_KINASE_ACTIVITY	17	-0.51	-1.48	0.047	0.129	1
GO_REGULATION_OF_EXTENT_OF_CELL_GROWTH	100	-0.34	-1.48	0.012	0.132	1
GO_NEGATIVE_REGULATION_OF_LYASE_ACTIVITY	27	-0.44	-1.48	0.05	0.133	1
GO_FEEDING_BEHAVIOR	91	-0.34	-1.48	0.024	0.133	1
GO_CILIARY_BASE	23	-0.46	-1.48	0.049	0.133	1
GO_ENDOSOME_TO_LYSOSOME_TRANSPORT	39	-0.4	-1.48	0.043	0.132	1
GO_POSITIVE_REGULATION_OF_SODIUM_ION_TRANSPORT	33	-0.42	-1.48	0.036	0.133	1
GO_DETECTION_OF_ABIOTIC_STIMULUS	115	-0.33	-1.48	0.005	0.133	1
GO_MONOAMINE_TRANSPORT	22	-0.47	-1.48	0.049	0.133	1
GO_DYNEIN_COMPLEX	43	-0.39	-1.47	0.03	0.136	1
GO_REGULATION_OF_VACUOLE_ORGANIZATION	41	-0.4	-1.47	0.042	0.136	1
GO_ADRENAL_GLAND_DEVELOPMENT	23	-0.46	-1.47	0.044	0.136	1
GO_PRIMARY_AMINO_COMPOUND_METABOLIC_PROCESS	15	-0.5	-1.47	0.062	0.136	1
GO_ASSOCIATIVE_LEARNING	73	-0.36	-1.47	0.025	0.137	1
GO_EYE_PHOTORECEPTOR_CELL_DIFFERENTIATION	42	-0.4	-1.47	0.031	0.137	1
GO_POSITIVE_REGULATION_OF_DEVELOPMENTAL_GROWTH	154	-0.31	-1.47	0.005	0.139	1
GO_POSITIVE_REGULATION_OF_CATION_TRANSMEMBRANE_TRANSPOR	97	-0.34	-1.47	0.017	0.139	1
GO_REGULATION_OF_CARDIAC_MUSCLE_CONTRACTION	66	-0.35	-1.46	0.032	0.142	1
GO_RAC_PROTEIN_SIGNAL_TRANSDUCTION	18	-0.49	-1.46	0.049	0.143	1
GO_IONOTROPIC_GLUTAMATE_RECEPTOR_BINDING	23	-0.46	-1.46	0.06	0.144	1
GO_THYROID_HORMONE_METABOLIC_PROCESS	16	-0.49	-1.46	0.071	0.145	1
GO_NEGATIVE_REGULATION_OF_STRIATED_MUSCLE_CELL_APOPTOTIC_PROCESS	17	-0.49	-1.46	0.074	0.145	1
GO_MEMBRANE_ASSEMBLY	25	-0.45	-1.46	0.049	0.145	1
GO_REGULATION_OF_FATTY_ACID_OXIDATION	27	-0.44	-1.46	0.032	0.146	1
GO_REGULATION_OF_CORTICOSTEROID_HORMONE_SECRETION	15	-0.51	-1.46	0.064	0.146	1
GO_REGULATION_OF_NEURON_MIGRATION	29	-0.43	-1.46	0.045	0.147	1
GO_NEURON_PROJECTION_GUIDANCE	204	-0.3	-1.46	0.005	0.147	1
GO_NEGATIVE_REGULATION_OF_BLOOD_VESSEL_ENDOTHELIAL_CELL_MIGRATION	24	-0.45	-1.46	0.045	0.147	1
GO_CALMODULIN_DEPENDENT_PROTEIN_KINASE_ACTIVITY	28	-0.43	-1.45	0.065	0.148	1
GO_NEGATIVE_REGULATION_OF_NERVOUS_SYSTEM_DEVELOPMENT	260	-0.29	-1.45	0.005	0.148	1
GO_HEART_GROWTH	26	-0.44	-1.45	0.052	0.15	1
GO_CEREBRAL_CORTEX_CELL_MIGRATION	42	-0.38	-1.45	0.037	0.15	1
GO_REGULATION_OF_CYTOSOLIC_CALCIUM_ION_CONCENTRATION	202	-0.3	-1.45	0	0.15	1
GO_CHONDROITIN_SULFATE_PROTEOGLYCAN_METABOLIC_PROCESS	42	-0.39	-1.45	0.039	0.149	1
GO_NEURON_PROJECTION_EXTENSION	52	-0.37	-1.45	0.035	0.15	1
GO_REGULATED_EXOCYTOSIS	219	-0.3	-1.45	0.005	0.151	1
GO_NEGATIVE_REGULATION_OF_POTASSIUM_ION_TRANSPORT	32	-0.41	-1.45	0.047	0.153	1
GO_REGULATION_OF_MICROTUBULE_POLYMERIZATION	31	-0.42	-1.45	0.054	0.153	1
GO_POSITIVE_REGULATION_OF_CELL_DEVELOPMENT	463	-0.27	-1.45	0.001	0.153	1
GO_REGULATION_OF_APPETITE	24	-0.44	-1.45	0.064	0.153	1
GO_SPERM_FLAGELLUM	53	-0.37	-1.45	0.036	0.153	1
GO_REGULATION_OF_SODIUM_ION_TRANSPORT	75	-0.34	-1.45	0.025	0.154	1
GO_SENSORY_PERCEPTION_OF_LIGHT_STIMULUS	211	-0.3	-1.44	0.002	0.156	1
GO_REGULATION_OF_MUSCLE_SYSTEM_PROCESS	192	-0.3	-1.44	0.002	0.156	1
GO_CUL3_RING_UBIQUITIN_LIGASE_COMPLEX	63	-0.36	-1.44	0.035	0.156	1
GO_REGULATION_OF_MULTICELLULAR_ORGANISM_GROWTH	66	-0.35	-1.44	0.032	0.159	1
GO_ACTIN_MEDIATED_CELL_CONTRACTION	74	-0.35	-1.44	0.026	0.16	1
GO_PHENOL_CONTAINING_COMPOUND_BIOSYNTHETIC_PROCESS	32	-0.41	-1.44	0.071	0.16	1
GO_REGULATION_OF_SENSORY_PERCEPTION	36	-0.4	-1.44	0.059	0.16	1
GO_ORGANELLE_FUSION	128	-0.31	-1.44	0.022	0.16	1
GO_A_BAND	34	-0.41	-1.44	0.048	0.16	1
GO_DIPEPTIDASE_ACTIVITY	15	-0.5	-1.44	0.092	0.161	1
GO_REGULATION_OF_CATECHOLAMINE_METABOLIC_PROCESS	17	-0.48	-1.44	0.07	0.161	1
GO_CENTRAL_NERVOUS_SYSTEM_PROJECTION_NEURON_AXONOGENE	21	-0.46	-1.43	0.084	0.162	1
GO_CALCIUM_ACTIVATED_CATION_CHANNEL_ACTIVITY	28	-0.42	-1.43	0.055	0.164	1
GO_SECONDARY_METABOLITE_BIOSYNTHETIC_PROCESS	19	-0.47	-1.43	0.067	0.164	1
GO_GLIOGENESIS	174	-0.3	-1.43	0.005	0.165	1
GO_PHOSPHOLIPASE_BINDING	18	-0.47	-1.43	0.066	0.165	1
GO_CERAMIDE_BIOSYNTHETIC_PROCESS	37	-0.4	-1.43	0.054	0.165	1
GO_DOPAMINE_RECEPTOR_SIGNALING_PATHWAY	30	-0.41	-1.43	0.042	0.164	1
GO_NEGATIVE_REGULATION_OF_SMALL_GTPASE_MEDIATED_SIGNAL_TRANSDUCTION	38	-0.39	-1.43	0.05	0.167	1
GO_PROTEIN_MANNOSYLATION	22	-0.45	-1.43	0.066	0.17	1
GO_HINDBRAIN_MORPHOGENESIS	39	-0.39	-1.42	0.045	0.17	1
GO_POSITIVE_REGULATION_OF_MULTICELLULAR_ORGANISM_GROWTH	32	-0.41	-1.42	0.068	0.17	1
GO_REGULATION_OF_RYANODINE_SENSITIVE_CALCIUM_RELEASE_CHANNEL_ACTIVITY	27	-0.42	-1.42	0.055	0.172	1
GO_PIGMENTATION	83	-0.33	-1.42	0.028	0.175	1
GO_SINGLE_ORGANISM_MEMBRANE_FUSION	128	-0.31	-1.42	0.018	0.174	1
GO_REGULATION_OF_STRIATED_MUSCLE_CONTRACTION	79	-0.34	-1.42	0.029	0.178	1
GO_VESICLE_DOCKING_INVOLVED_IN_EXOCYTOSIS	35	-0.4	-1.42	0.056	0.179	1
GO_REGULATION_OF_STEROID_HORMONE_SECRETION	20	-0.45	-1.42	0.085	0.178	1
GO_REGULATION_OF_DEVELOPMENTAL_GROWTH	287	-0.28	-1.42	0.003	0.178	1
GO_MYOSIN_FILAMENT	22	-0.44	-1.41	0.055	0.18	1

GO_PROTEIN_KINASE_A_CATALYTIC_SUBUNIT_BINDING	15	-0.5	-1.41	0.083	0.181	1
GO_TRANSMEMBRANE_RECEPTOR_PROTEIN_PHOSPHATASE_ACTIVITY	17	-0.48	-1.41	0.078	0.181	1
GO_TRANSPORT_VESICLE	333	-0.27	-1.41	0.002	0.182	1
GO_RETINA_VASCULATURE_DEVELOPMENT_IN_CAMERA_TYPE_EYE	16	-0.49	-1.41	0.089	0.182	1
GO_CEREBELLAR_CORTEX_MORPHOGENESIS	29	-0.41	-1.41	0.063	0.183	1
GO_ACTIVATING_TRANSCRIPTION_FACTOR_BINDING	57	-0.35	-1.41	0.051	0.183	1
GO_HETEROTRIMERIC_G_PROTEIN_COMPLEX	32	-0.41	-1.41	0.062	0.183	1
GO_LIGAND_GATED_CALCIUM_CHANNEL_ACTIVITY	16	-0.48	-1.41	0.082	0.184	1
GO_DENDRITIC_SPINE_ORGANIZATION	17	-0.47	-1.41	0.076	0.183	1
GO_SODIUM_ION_TRANSPORT	142	-0.3	-1.41	0.017	0.185	1
GO_SECRETION_BY_CELL	474	-0.26	-1.41	0.001	0.185	1
GO_REGULATION_OF_MEMBRANE_DEPOLARIZATION	41	-0.39	-1.41	0.051	0.185	1
GO_HINDBRAIN_DEVELOPMENT	135	-0.3	-1.4	0.012	0.186	1
GO_CLATHRIN_BINDING	65	-0.35	-1.4	0.043	0.187	1
GO_POSITIVE_REGULATION_OF_RESPONSE_TO_EXTRACELLULAR_STIM	48	-0.37	-1.4	0.046	0.189	1
GO_GLIAL_CELL_DIFFERENTIATION	136	-0.3	-1.4	0.03	0.189	1
GO_NITRIC_OXIDE_MEDIATED_SIGNAL_TRANSDUCTION	18	-0.46	-1.4	0.083	0.188	1
GO_NEGATIVE_REGULATION_OF_BEHAVIOR	17	-0.47	-1.4	0.081	0.188	1
GO_REGULATION_OF_CLATHRIN_MEDIATED_ENDOCYTOSIS	16	-0.49	-1.4	0.097	0.189	1
GO_VESICLE_LOCALIZATION	220	-0.29	-1.4	0.005	0.189	1
GO_NEGATIVE_REGULATION_OF_CATION_CHANNEL_ACTIVITY	34	-0.39	-1.4	0.067	0.189	1
GO_AUTOPHAGOSOME	76	-0.34	-1.4	0.038	0.191	1
GO_DEVELOPMENTAL_MATURATION	191	-0.29	-1.4	0.014	0.19	1
GO_DOPAMINE_RECEPTOR_BINDING	17	-0.47	-1.4	0.088	0.191	1
GO_INCLUSION_BODY	68	-0.34	-1.4	0.046	0.191	1
GO_REGULATION_OF_SKELETAL_MUSCLE_CELL_DIFFERENTIATION	17	-0.46	-1.4	0.092	0.191	1
GO_MEMBRANE_FUSION	157	-0.3	-1.39	0.011	0.194	1
GO_N_GLYCAN_PROCESSING	20	-0.45	-1.39	0.103	0.194	1
GO_ESTABLISHMENT_OF_LOCALIZATION_BY_MOVEMENT_ALONG_MICROTUBULE	95	-0.32	-1.39	0.034	0.194	1
GO_CGMP_BINDING	16	-0.48	-1.39	0.088	0.195	1
GO_SPECIFICATION_OF_SYMMETRY	114	-0.31	-1.39	0.033	0.195	1
GO_REGULATION_OF_CALCIUM_ION_TRANSPORT	208	-0.28	-1.39	0.015	0.196	1
GO_REGULATION_OF_DEVELOPMENTAL_PIGMENTATION	16	-0.48	-1.39	0.104	0.196	1
GO_NEGATIVE_REGULATION_OF_NEURON_DIFFERENTIATION	189	-0.29	-1.39	0.009	0.196	1
GO_PHOTORECEPTOR_OUTER_SEGMENT	69	-0.34	-1.39	0.036	0.198	1
GO_REGULATION_OF_PROTEIN_ACETYLATION	63	-0.35	-1.39	0.035	0.198	1
GO_CYTOSKELETAL_ADAPTOR_ACTIVITY	16	-0.48	-1.39	0.08	0.198	1
GO_PERICENTRIOLAR_MATERIAL	17	-0.47	-1.39	0.091	0.198	1
GO_M_BAND	21	-0.44	-1.39	0.093	0.199	1
GO_REGULATION_OF_HORMONE_SECRETION	258	-0.27	-1.39	0.003	0.2	1
GO_REGULATION_OF_SODIUM_ION_TRANSMEMBRANE_TRANSPORT	47	-0.36	-1.38	0.062	0.201	1
GO_RETROGRADE_TRANSPORT_ENDOSOME_TO_GOLGI	71	-0.34	-1.38	0.039	0.203	1
GO_REGULATION_OF_RECEPTOR_ACTIVITY	117	-0.31	-1.38	0.038	0.203	1
GO_POSITIVE_REGULATION_OF_CIRCADIAN_RHYTHM	21	-0.44	-1.38	0.095	0.205	1
GO_POSITIVE_REGULATION_OF_TRANSMEMBRANE_TRANSPORT	131	-0.3	-1.38	0.018	0.205	1
GO_PROTON_TRANSPORTING_V_TYPE_ATPASE_COMPLEX	23	-0.43	-1.38	0.082	0.206	1
GO_POSITIVE_REGULATION_OF_MUSCLE_HYPERTROPHY	20	-0.45	-1.38	0.086	0.208	1
GO_TRANSMEMBRANE_RECEPTOR_PROTEIN_KINASE_ACTIVITY	81	-0.32	-1.38	0.036	0.209	1
GO_REGULATION_OF_PEPTIDE_SECRETION	206	-0.28	-1.38	0.014	0.21	1
GO_RECEPTOR_COMPLEX	326	-0.27	-1.38	0	0.209	1
GO_EYE_PHOTORECEPTOR_CELL_DEVELOPMENT	31	-0.4	-1.38	0.065	0.21	1
GO_HORMONE_BINDING	65	-0.34	-1.37	0.041	0.21	1
GO_NEUROEPITHELIAL_CELL_DIFFERENTIATION	61	-0.34	-1.37	0.038	0.212	1
GO_RETINA_LAYER_FORMATION	22	-0.43	-1.37	0.092	0.216	1
GO_REGULATION_OF_MUSCLE_CONTRACTION	147	-0.29	-1.37	0.016	0.216	1
GO_REGULATION_OF_DENDRITE_EXTENSION	19	-0.45	-1.37	0.091	0.216	1
GO_KIDNEY_EPITHELIUM_DEVELOPMENT	125	-0.3	-1.37	0.016	0.221	1
GO_TRANS_GOLGI_NETWORK	186	-0.28	-1.36	0.02	0.223	1
GO_NEGATIVE_REGULATION_OF_VASCULATURE_DEVELOPMENT	80	-0.33	-1.36	0.045	0.223	1
GO_SYNAPTIC_VESICLE_ENDOCYTOSIS	17	-0.46	-1.36	0.117	0.223	1
GO_PHOSPHATIDYLINOSITOL_BINDING	198	-0.28	-1.36	0.017	0.223	1
GO_RESPONSE_TO_FOLIC_ACID	15	-0.49	-1.36	0.102	0.223	1
GO_OLFACTORY_BULB_INTERNEURON_DIFFERENTIATION	15	-0.47	-1.36	0.121	0.223	1
GO_MEMBRANE_REPOLARIZATION	15	-0.48	-1.36	0.125	0.225	1
GO_BRANCHED_CHAIN_AMINO_ACID_METABOLIC_PROCESS	23	-0.41	-1.36	0.108	0.227	1
GO_FEAR_RESPONSE	29	-0.41	-1.36	0.096	0.231	1
GO_SCF_DEPENDENT_PROTEASOMAL_UBIQUITIN_DEPENDENT_PROTEIN_CATABOLIC_PROCESS	23	-0.42	-1.36	0.094	0.231	1
GO_BETA_CATENIN_BINDING	84	-0.31	-1.35	0.034	0.233	1
GO_INTRACELLULAR_LIGAND_GATED_ION_CHANNEL_ACTIVITY	28	-0.39	-1.35	0.102	0.234	1
GO_CAMP_BIOSYNTHETIC_PROCESS	17	-0.45	-1.35	0.098	0.234	1
GO_VISUAL_BEHAVIOR	50	-0.35	-1.35	0.069	0.234	1
GO_NITRIC_OXIDE_SYNTHASE_BINDING	19	-0.44	-1.35	0.102	0.233	1
GO_DENDRITIC_SPINE_DEVELOPMENT	19	-0.44	-1.35	0.115	0.233	1
GO_STRIATED_MUSCLE_CONTRACTION	98	-0.32	-1.35	0.04	0.234	1
GO_DEVELOPMENTAL_PIGMENTATION	38	-0.37	-1.35	0.082	0.234	1
GO_MUSCLE_ADAPTATION	29	-0.4	-1.35	0.091	0.239	1
GO_ADRENERGIC_RECEPTOR_BINDING	19	-0.44	-1.35	0.118	0.24	1
GO_CATECHOLAMINE_METABOLIC_PROCESS	40	-0.37	-1.35	0.08	0.24	1
GO_RESPONSE_TO_AMPHETAMINE	30	-0.4	-1.35	0.101	0.24	1
GO_DETECTION_OF_MECHANICAL_STIMULUS	41	-0.36	-1.35	0.075	0.24	1
GO_DIVALENT_INORGANIC_CATION_HOMEOSTASIS	340	-0.26	-1.35	0.008	0.24	1
GO_VENTRICULAR_SYSTEM_DEVELOPMENT	25	-0.41	-1.35	0.105	0.241	1
GO_CELL_PROJECTION_CYTOPLASM	52	-0.34	-1.35	0.064	0.241	1
GO_CIRCULATORY_SYSTEM_PROCESS	364	-0.26	-1.34	0.005	0.243	1
GO_INSULIN_SECRETION	38	-0.37	-1.34	0.079	0.244	1

GO_PEPTIDE_SECRETION	57	-0.34	-1.34	0.073	0.244	1
GO_POSITIVE_REGULATION_OF_POTASSIUM_ION_TRANSMEMBRANE_TRANSPORTER_ACTIVITY	16	-0.47	-1.34	0.126	0.245	1
GO_REGULATION_OF_SMOOTH_MUSCLE_CELL_DIFFERENTIATION	20	-0.44	-1.34	0.123	0.246	1
GO_SPECTRIN_BINDING	24	-0.42	-1.34	0.111	0.246	1
GO_CYCLASE_ACTIVITY	22	-0.42	-1.34	0.095	0.248	1
GO_STRIATED_MUSCLE_CELL_DIFFERENTIATION	172	-0.28	-1.34	0.029	0.25	1
GO_CELLULAR_RESPONSE_TO_CAMP	50	-0.35	-1.34	0.069	0.25	1
GO_POSITIVE_REGULATION_OF_DEPHOSPHORYLATION	47	-0.35	-1.34	0.059	0.25	1
GO_INTERCALATED_DISC	51	-0.35	-1.34	0.091	0.251	1
GO_OTIC_VESICLE_DEVELOPMENT	15	-0.47	-1.33	0.112	0.252	1
GO_PROTEOGLYCAN_METABOLIC_PROCESS	82	-0.31	-1.33	0.05	0.252	1
GO_CELL_PROJECTION_MEMBRANE	295	-0.26	-1.33	0.007	0.252	1
GO_HIPPOCAMPUS_DEVELOPMENT	72	-0.32	-1.33	0.07	0.256	1
GO_GLYCOSPHINGOLIPID_BIOSYNTHETIC_PROCESS	25	-0.41	-1.33	0.102	0.256	1
GO_MULTICELLULAR_ORGANISMAL_RESPONSE_TO_STRESS	68	-0.32	-1.33	0.084	0.257	1
GO_DICARBOXYLIC_ACID_TRANSPORT	71	-0.32	-1.33	0.08	0.258	1
GO_PROTEOGLYCAN_BIOSYNTHETIC_PROCESS	58	-0.33	-1.33	0.093	0.259	1
GO_PHOSPHATIDYLINOSITOL_BIOSYNTHETIC_PROCESS	120	-0.29	-1.33	0.042	0.259	1
GO_REGULATION_OF_ANION_TRANSPORT	136	-0.29	-1.33	0.046	0.26	1
GO_SODIUM_ION_TRANSMEMBRANE_TRANSPORTER_ACTIVITY	135	-0.29	-1.33	0.03	0.26	1
GO_PROTEIN_LOCALIZATION_TO_CELL_PERIPHERY	150	-0.28	-1.33	0.026	0.26	1
GO_REGULATION_OF_FILOPODIUM_ASSEMBLY	38	-0.36	-1.33	0.093	0.26	1
GO_TRANSCRIPTIONAL_REPRESSOR_ACTIVITY_RNA_POLYMERASE_II_ACTIVATING_TRANSCRIPTION_FACTOR_BINDING	53	-0.34	-1.33	0.094	0.261	1
GO_SMAD_PROTEIN_SIGNAL_TRANSDUCTION	56	-0.34	-1.33	0.081	0.26	1
GO_CORONARY_VASCULATURE_DEVELOPMENT	37	-0.37	-1.32	0.08	0.26	1
GO_TRANSCRIPTIONAL_REPRESSOR_ACTIVITY_RNA_POLYMERASE_II_TRANSCRIPTION_FACTOR_BINDING	85	-0.31	-1.32	0.057	0.26	1
GO_NEGATIVE_REGULATION_OF_GLIAL_CELL_DIFFERENTIATION	26	-0.39	-1.32	0.112	0.262	1
GO_AUTOPHAGOSOME_MEMBRANE	26	-0.41	-1.32	0.111	0.262	1
GO_REGULATION_OF_SMALL_GTPASE_MEDIATED_SIGNAL_TRANSDUCTI	271	-0.26	-1.32	0.015	0.262	1
GO_REGULATION_OF_PATHWAY_RESTRICTED_SMAD_PROTEIN_PHOSPHORYLATION	60	-0.33	-1.32	0.102	0.266	1
GO_NEGATIVE_REGULATION_OF_CELL_DEVELOPMENT	300	-0.26	-1.32	0.019	0.265	1
GO_FOREBRAIN_DEVELOPMENT	353	-0.25	-1.32	0.015	0.265	1
GO_REGULATION_OF_VASCULAR_PERMEABILITY	29	-0.38	-1.32	0.105	0.267	1
GO_VASCULOGENESIS	59	-0.33	-1.32	0.073	0.267	1
GO_REGULATION_OF_NEUROLOGICAL_SYSTEM_PROCESS	68	-0.32	-1.32	0.062	0.271	1
GO_NEGATIVE_REGULATION_OF_ION_TRANSPORT	126	-0.29	-1.32	0.039	0.271	1
GO_NEGATIVE_REGULATION_OF_ANION_TRANSPORT	33	-0.37	-1.32	0.095	0.27	1
GO_SPINAL_CORD_DEVELOPMENT	106	-0.3	-1.32	0.049	0.271	1
GO_BICARBONATE_TRANSPORT	44	-0.35	-1.32	0.109	0.27	1
GO_REGULATION_OF_ION_HOMEOSTASIS	201	-0.27	-1.31	0.024	0.272	1
GO_MUSCLE_CELL_DIFFERENTIATION	236	-0.26	-1.31	0.022	0.273	1
GO_CEREBRAL_CORTEX_RADIALLY_ORIENTED_CELL_MIGRATION	28	-0.39	-1.31	0.117	0.273	1
GO_RAB_GUANYL_NUCLEOTIDE_EXCHANGE_FACTOR_ACTIVITY	30	-0.38	-1.31	0.101	0.273	1
GO_FATTY_ACID_BETA_OXIDATION_USING_ACYL_COA_DEHYDROGENA	18	-0.43	-1.31	0.134	0.276	1
GO_POSITIVE_REGULATION_OF_GLYCOGEN_METABOLIC_PROCESS	17	-0.45	-1.31	0.137	0.276	1
GO_DETECTION_OF_LIGHT_STIMULUS	58	-0.33	-1.31	0.094	0.276	1
GO_STRIATUM_DEVELOPMENT	16	-0.44	-1.31	0.139	0.277	1
GO_INOSITOL_PHOSPHATE_PHOSPHATASE_ACTIVITY	20	-0.42	-1.31	0.144	0.278	1
GO_NUCLEOSIDE_TRIPHOSPHATASE_REGULATOR_ACTIVITY	320	-0.25	-1.31	0.013	0.281	1
GO_REGULATION_OF_CELL_SIZE	170	-0.27	-1.31	0.045	0.28	1
GO_HEART_DEVELOPMENT	462	-0.24	-1.31	0.01	0.28	1
GO_G_PROTEIN_BETA_GAMMA_SUBUNIT_COMPLEX_BINDING	21	-0.41	-1.31	0.124	0.28	1
GO_REGULATION_OF_SODIUM_ION_TRANSMEMBRANE_TRANSPORTER_ACTIVITY	38	-0.36	-1.31	0.107	0.28	1
GO_RESPONSE_TO_EPINEPHRINE	16	-0.44	-1.3	0.133	0.286	1
GO_CYCLIC_NUCLEOTIDE_BIOSYNTHETIC_PROCESS	33	-0.37	-1.3	0.113	0.288	1
GO_NEGATIVE_REGULATION_OF_NEURON_APOPTOTIC_PROCESS	134	-0.28	-1.3	0.052	0.291	1
GO_REGULATION_OF_HEART_GROWTH	41	-0.35	-1.3	0.108	0.292	1
GO_PROTEIN_ADP_RIBOSYLATION	20	-0.42	-1.3	0.147	0.296	1
GO_PIGMENT_CELL_DIFFERENTIATION	28	-0.38	-1.29	0.143	0.297	1
GO_NEUROTRANSMITTER_METABOLIC_PROCESS	25	-0.4	-1.29	0.134	0.297	1
GO_ORGANIC_CATION_TRANSMEMBRANE_TRANSPORTER_ACTIVITY	15	-0.45	-1.29	0.132	0.298	1
GO_REGULATION_OF_NOREPINEPHRINE_SECRETION	17	-0.44	-1.29	0.145	0.298	1
GO_CATION_CATION_ANTIPORTER_ACTIVITY	23	-0.4	-1.29	0.153	0.3	1
GO_SYNAPTIC_VESICLE_RECYCLING	23	-0.4	-1.29	0.15	0.3	1
GO_PROTEIN_O_LINKED_MANNOSYLATION	16	-0.43	-1.29	0.152	0.303	1
GO_NEGATIVE_REGULATION_OF_AXONOGENESIS	65	-0.32	-1.29	0.089	0.304	1
GO_ACTININ_BINDING	29	-0.38	-1.29	0.118	0.303	1
GO_GMP_METABOLIC_PROCESS	20	-0.42	-1.29	0.179	0.304	1
GO_VASCULAR_PROCESS_IN_CIRCULATORY_SYSTEM	162	-0.27	-1.29	0.07	0.305	1
GO_SMOOTH_MUSCLE_CONTRACTION	45	-0.34	-1.29	0.111	0.305	1
GO_CHLORIDE_TRANSPORT	99	-0.29	-1.29	0.072	0.305	1
GO_ORGAN_GROWTH	68	-0.32	-1.29	0.099	0.306	1
GO_SMOOTH_MUSCLE_CELL_DIFFERENTIATION	30	-0.38	-1.29	0.132	0.307	1
GO_DETECTION_OF_MECHANICAL_STIMULUS_INVOLVED_IN_SENSORY_PERCEPTION	24	-0.39	-1.29	0.15	0.307	1
GO_NEPHRON_DEVELOPMENT	115	-0.29	-1.29	0.072	0.306	1
GO_SULFOTRANSFERASE_ACTIVITY	51	-0.34	-1.29	0.106	0.306	1
GO_RNA_POLYMERASE_II_ACTIVATING_TRANSCRIPTION_FACTOR_BINDI	36	-0.36	-1.28	0.113	0.307	1
GO_ACIDIC_AMINO_ACID_TRANSPORT	21	-0.41	-1.28	0.139	0.307	1
GO_CEREBELLAR_CORTEX_DEVELOPMENT	45	-0.34	-1.28	0.12	0.307	1
GO_CHLORIDE_CHANNEL_COMPLEX	50	-0.33	-1.28	0.115	0.309	1
GO_CYTOSKELETON_DEPENDENT_INTRACELLULAR_TRANSPORT	113	-0.29	-1.28	0.061	0.309	1

GO_POSITIVE_REGULATION_OF_TRANSCRIPTION_FROM_RNA_POLYMERASE_II_PROMOTER_INVOLVED_IN_CELLULAR_RESPONSE_TO_CHEMICAL_STIMULUS	27	-0.38	-1.28	0.157	0.31	1
GO_LIPID_MODIFICATION	208	-0.26	-1.28	0.039	0.312	1
GO_VASOCONSTRICTION	28	-0.38	-1.28	0.13	0.314	1
GO_NEGATIVE_REGULATION_OF_DENDRITE_MORPHOGENESIS	15	-0.46	-1.28	0.161	0.315	1
GO_REGULATION_OF_RENAL_SODIUM_EXCRETION	24	-0.39	-1.28	0.14	0.315	1
GO_REGULATION_OF_CIRCADIAN_RHYTHM	103	-0.29	-1.28	0.076	0.316	1
GO_COCHLEA_DEVELOPMENT	39	-0.35	-1.28	0.118	0.319	1
GO_ENDOCARDIAL_CUSHION_FORMATION	15	-0.45	-1.27	0.151	0.321	1
GO_REGULATION_OF_CELLULAR_RESPONSE_TO_GROWTH_FACTOR_STIMULUS	227	-0.26	-1.27	0.042	0.322	1
GO_STRUCTURAL_CONSTITUENT_OF_MUSCLE	41	-0.35	-1.27	0.131	0.326	1
GO_TRANSFERASE_ACTIVITY_TRANSFERRING_SULFUR_CONTAINING_GROUPS	66	-0.31	-1.27	0.117	0.329	1
GO_CELL_GROWTH	129	-0.28	-1.27	0.074	0.329	1
GO_ASPARTATE_FAMILY_AMINO_ACID_CATABOLIC_PROCESS	19	-0.42	-1.27	0.152	0.329	1
GO_RNA_POLYMERASE_II_REPRESSING_TRANSCRIPTION_FACTOR_BIN	27	-0.38	-1.27	0.148	0.334	1
GO_CLATHRIN_MEDIATED_ENDOCYTOSIS	35	-0.36	-1.26	0.136	0.335	1
GO_RESPONSE_TO_STIMULUS_INVOLVED_IN_REGULATION_OF_MUSCLE_ADAPTATION	15	-0.44	-1.26	0.168	0.335	1
GO_PROTEIN_KINASE_C_ACTIVATING_G_PROTEIN_COUPLED_RECEPTOR_SIGNALING_PATHWAY	32	-0.36	-1.26	0.138	0.336	1
GO_REGULATION_OF_STRIATED_MUSCLE_CELL_APOPTOTIC_PROCESS	22	-0.4	-1.26	0.15	0.336	1
GO_PIGMENT_GRANULE_LOCALIZATION	25	-0.39	-1.26	0.157	0.336	1
GO_NEGATIVE_REGULATION_OF_CELL_PROJECTION_ORGANIZATION	141	-0.27	-1.26	0.058	0.335	1
GO_POSITIVE_REGULATION_OF_SMOOTHENED_SIGNALING_PATHWAY	24	-0.38	-1.26	0.158	0.335	1
GO_BRAIN_MORPHOGENESIS	34	-0.36	-1.26	0.155	0.335	1
GO_REGULATION_OF_EXCRETION	29	-0.36	-1.26	0.132	0.335	1
GO_CELL_CELL_CONTACT_ZONE	64	-0.31	-1.26	0.118	0.334	1
GO_RETROMER_COMPLEX	21	-0.4	-1.26	0.158	0.334	1
GO_REGULATION_OF_BLOOD_PRESSURE	169	-0.27	-1.26	0.057	0.335	1
GO_VACUOLE_FUSION	21	-0.4	-1.26	0.182	0.337	1
GO_INSULIN_RECEPTOR_SIGNALING_PATHWAY	79	-0.3	-1.26	0.098	0.337	1
GO_NEGATIVE_REGULATION_OF_PROTEIN_POLYMERIZATION	55	-0.32	-1.26	0.124	0.338	1
GO_INTRINSIC_COMPONENT_OF_GOLGI_MEMBRANE	56	-0.32	-1.26	0.13	0.338	1
GO_AUTONOMIC_NERVOUS_SYSTEM_DEVELOPMENT	42	-0.33	-1.26	0.14	0.338	1
GO_RECEPTOR_SIGNALING_PROTEIN_ACTIVITY	172	-0.26	-1.26	0.061	0.338	1
GO_ACYL_COA_DEHYDROGENASE_ACTIVITY	17	-0.43	-1.26	0.178	0.341	1
GO_PHOSPHATIDYLINOSITOL_3_KINASE_BINDING	30	-0.37	-1.26	0.147	0.34	1
GO_NEGATIVE_REGULATION_OF_RESPONSE_TO_EXTRACELLULAR_STIMULUS	36	-0.34	-1.26	0.15	0.34	1
GO_LOCALIZATION_WITHIN_MEMBRANE	120	-0.28	-1.26	0.085	0.342	1
GO_MUSCLE_CELL_DEVELOPMENT	128	-0.27	-1.26	0.075	0.342	1
GO_PROTEIN_TARGETING_TO_PLASMA_MEMBRANE	23	-0.39	-1.26	0.17	0.342	1
GO_PROTEIN_LOCALIZATION_TO_MEMBRANE	374	-0.24	-1.26	0.038	0.342	1
GO_ANTIPORTER_ACTIVITY	71	-0.3	-1.25	0.112	0.342	1
GO_CELLULAR_RESPONSE_TO_PEPTIDE	271	-0.25	-1.25	0.044	0.342	1
GO_STEROID_HORMONE_RECEPTOR_ACTIVITY	59	-0.31	-1.25	0.14	0.346	1
GO_ORGAN_MATURATION	18	-0.42	-1.25	0.169	0.348	1
GO_CELLULAR_RESPONSE_TO_NITROGEN_COMPOUND	497	-0.23	-1.25	0.017	0.348	1
GO_CULLIN_RING_UBIQUITIN_LIGASE_COMPLEX	149	-0.27	-1.25	0.078	0.348	1
GO_PHOSPHORIC_ESTER_HYDROLASE_ACTIVITY	361	-0.24	-1.25	0.036	0.35	1
GO_POSITIVE_REGULATION_OF_NEUROLOGICAL_SYSTEM_PROCESS	23	-0.38	-1.25	0.19	0.35	1
GO_LYTIC_VACUOLE_ORGANIZATION	50	-0.32	-1.25	0.127	0.35	1
GO_VESICLE_DOCKING	56	-0.31	-1.25	0.132	0.352	1
GO_CATION_AMINO_ACID_SYMPORTER_ACTIVITY	16	-0.43	-1.25	0.167	0.353	1
GO_TRANSMEMBRANE_RECEPTOR_PROTEIN_TYROSINE_KINASE_ACTIV	64	-0.31	-1.25	0.135	0.353	1
GO_NEGATIVE_REGULATION_OF_HORMONE_SECRETION	74	-0.3	-1.25	0.114	0.353	1
GO_MUSCLE_STRUCTURE_DEVELOPMENT	431	-0.24	-1.25	0.029	0.354	1
GO_PHOSPHOLIPID_BINDING	353	-0.24	-1.24	0.032	0.357	1
GO_AXO_DENDRITIC_TRANSPORT	37	-0.34	-1.24	0.159	0.357	1
GO_REGULATION_OF_URINE_VOLUME	20	-0.41	-1.24	0.182	0.357	1
GO_PROTEIN_LOCALIZATION_TO_LYSOSOME	20	-0.41	-1.24	0.183	0.357	1
GO_MYOSIN_II_COMPLEX	25	-0.38	-1.24	0.162	0.362	1
GO_SPERM_PRINCIPAL_PIECE	17	-0.41	-1.24	0.189	0.362	1
GO_HISTONE_H4_K16_ACETYLATION	17	-0.41	-1.24	0.185	0.361	1
GO_ACTIN_FILAMENT_BASED_MOVEMENT	93	-0.28	-1.24	0.109	0.362	1
GO_SODIUM_CHANNEL_REGULATOR_ACTIVITY	31	-0.36	-1.24	0.148	0.364	1
GO_REGULATION_OF_PH	89	-0.28	-1.24	0.126	0.367	1
GO_REGULATION_OF_PROTEIN_TARGETING_TO_MEMBRANE	30	-0.36	-1.24	0.185	0.367	1
GO_INORGANIC_ANION_TRANSMEMBRANE_TRANSPORTER_ACTIVITY	130	-0.27	-1.24	0.093	0.367	1
GO_REGULATION_OF_PHOSPHOLIPASE_C_ACTIVITY	39	-0.34	-1.24	0.161	0.366	1
GO_RESPONSE_TO_PAIN	29	-0.36	-1.23	0.168	0.373	1
GO_COMPACT_MYELIN	15	-0.44	-1.23	0.193	0.372	1
GO_OLIGODENDROCYTE_DIFFERENTIATION	60	-0.31	-1.23	0.139	0.372	1
GO_SYNAPTIC_TRANSMISSION_CHOLINERGIC	36	-0.34	-1.23	0.159	0.372	1
GO_DEACETYLASE_ACTIVITY	55	-0.31	-1.23	0.149	0.374	1
GO_POSITIVE_REGULATION_OF_GLUCOSE_TRANSPORT	42	-0.33	-1.23	0.154	0.374	1
GO_NEGATIVE_REGULATION_OF_GLIOGENESIS	36	-0.34	-1.23	0.172	0.373	1
GO_LIMBIC_SYSTEM_DEVELOPMENT	99	-0.28	-1.23	0.114	0.375	1
GO_RECEPTOR_INTERNALIZATION	50	-0.32	-1.23	0.155	0.375	1
GO_PHOSPHOLIPASE_C_ACTIVATING_G_PROTEIN_COUPLED_RECEPTOR_SIGNALING_PATHWAY	83	-0.29	-1.23	0.121	0.375	1
GO_REGULATION_OF_PEPTIDYL_SERINE_PHOSPHORYLATION	117	-0.27	-1.23	0.119	0.377	1
GO_DETECTION_OF_VISIBLE_LIGHT	43	-0.33	-1.23	0.166	0.377	1
GO_POSITIVE_REGULATION_OF_PHOSPHOLIPASE_ACTIVITY	53	-0.31	-1.23	0.147	0.378	1

GO_SH3_DOMAIN_BINDING	116	-0.27	-1.23	0.105	0.379	1
GO_LEADING_EDGE_MEMBRANE	132	-0.27	-1.23	0.092	0.379	1
GO_ACID_SECRETION	66	-0.3	-1.23	0.149	0.379	1
GO_POSITIVE_REGULATION_OF_CALCIUM_ION_TRANSMEMBRANE_TRANSPORT	59	-0.3	-1.23	0.151	0.38	1
GO_PHASIC_SMOOTH_MUSCLE_CONTRACTION	16	-0.42	-1.23	0.211	0.38	1
GO_ENDOTHELIUM_DEVELOPMENT	88	-0.29	-1.23	0.128	0.379	1
GO_ATRIOVENTRICULAR_VALVE_MORPHOGENESIS	16	-0.42	-1.23	0.207	0.379	1
GO_RESPONSE_TO_BMP	94	-0.28	-1.23	0.118	0.378	1
GO_INORGANIC_ANION_TRANSPORT	128	-0.27	-1.23	0.102	0.378	1
GO_EATING_BEHAVIOR	30	-0.36	-1.22	0.177	0.382	1
GO_ENERGY_RESERVE_METABOLIC_PROCESS	72	-0.3	-1.22	0.141	0.382	1
GO_INTRACELLULAR_LIPID_TRANSPORT	20	-0.4	-1.22	0.194	0.383	1
GO_SOLUTE_CATION_ANTIPORTER_ACTIVITY	29	-0.36	-1.22	0.18	0.384	1
GO_MICROTUBULE_BASED_MOVEMENT	199	-0.25	-1.22	0.084	0.386	1
GO_CLATHRIN_COAT_OF_COATED_PIT	16	-0.42	-1.22	0.214	0.386	1
GO_TELENCEPHALON_DEVELOPMENT	225	-0.25	-1.22	0.075	0.39	1
GO_REGULATION_OF_FEEDING_BEHAVIOR	22	-0.39	-1.22	0.201	0.395	1
GO_RAB_GTPASE_BINDING	119	-0.27	-1.22	0.124	0.397	1
GO_DERMATAN_SULFATE_PROTEOGLYCAN_METABOLIC_PROCESS	16	-0.42	-1.21	0.223	0.398	1
GO_RESPONSE_TO_AXON_INJURY	47	-0.32	-1.21	0.166	0.398	1
GO_HETEROPHILIC_CELL_CELL_ADHESION_VIA_PLASMA_MEMBRANE_CELL_ADHESION_MOLECULES	45	-0.32	-1.21	0.17	0.398	1
GO_POSITIVE_REGULATION_OF_BMP_SIGNALING_PATHWAY	32	-0.35	-1.21	0.172	0.398	1
GO_CELLULAR_RESPONSE_TO_ZINC_ION	16	-0.41	-1.21	0.195	0.398	1
GO_PHOSPHATIDYLSERINE_BINDING	33	-0.35	-1.21	0.18	0.399	1
GO_ION_GATED_CHANNEL_ACTIVITY	43	-0.33	-1.21	0.174	0.399	1
GO_FOREBRAIN_GENERATION_OF_NEURONS	66	-0.3	-1.21	0.168	0.399	1
GO_REGULATION_OF_ASTROCYTE_DIFFERENTIATION	27	-0.37	-1.21	0.181	0.4	1
GO_POTASSIUM_ION_HOMEOSTASIS	18	-0.4	-1.21	0.214	0.4	1
GO_AP_TYPE_MEMBRANE_COAT_ADAPTOR_COMPLEX	40	-0.33	-1.21	0.183	0.401	1
GO_MITOCHONDRION_LOCALIZATION	35	-0.33	-1.21	0.178	0.401	1
GO_NEGATIVE_REGULATION_OF_MUSCLE_TISSUE_DEVELOPMENT	36	-0.34	-1.21	0.188	0.403	1
GO_POSITIVE_REGULATION_OF_TRANSMEMBRANE_RECEPTOR_PROTEIN_SERINE_THREONINE_KINASE_SIGNALING_PATHWAY	100	-0.27	-1.21	0.121	0.404	1
GO_DETECTION_OF_LIGHT_STIMULUS_INVOLVED_IN_SENSORY_PERCEPTION	18	-0.4	-1.21	0.217	0.403	1
GO_SENSORY_PERCEPTION_OF_MECHANICAL_STIMULUS	150	-0.26	-1.21	0.115	0.407	1
GO_SNAP_RECEPTOR_ACTIVITY	38	-0.33	-1.21	0.196	0.407	1
GO_GLUCAN_METABOLIC_PROCESS	58	-0.3	-1.21	0.154	0.407	1
GO_SARCOMERE_ORGANIZATION	27	-0.36	-1.21	0.2	0.407	1
GO_NEGATIVE_REGULATION_OF_PEPTIDYL_THREONINE_PHOSPHORYLATION	15	-0.41	-1.21	0.22	0.407	1
GO_REGULATION_OF_RESPONSE_TO_EXTRACELLULAR_STIMULUS	171	-0.25	-1.2	0.126	0.409	1
GO_CELLULAR_MONOVALENT_INORGANIC_CATION_HOMEOSTASIS	95	-0.28	-1.2	0.134	0.409	1
GO_POSITIVE_REGULATION_OF_FILOPODIUM_ASSEMBLY	26	-0.36	-1.2	0.222	0.409	1
GO_AXONEME_PART	22	-0.38	-1.2	0.208	0.409	1
GO_METHYLATED_HISTONE_BINDING	49	-0.31	-1.2	0.175	0.409	1
GO_BETA_TUBULIN_BINDING	36	-0.34	-1.2	0.187	0.408	1
GO_CEREBRAL_CORTEX_NEURON_DIFFERENTIATION	22	-0.37	-1.2	0.207	0.412	1
GO_MESONEPHROS_DEVELOPMENT	90	-0.28	-1.2	0.164	0.417	1
GO_CYTOPLASMIC_MICROTUBULE_ORGANIZATION	42	-0.32	-1.2	0.176	0.417	1
GO_SPHINGOLIPID_BIOSYNTHETIC_PROCESS	69	-0.29	-1.2	0.16	0.417	1
GO_PHOSPHATIDYLINOSITOL_3_PHOSPHATE_BINDING	31	-0.35	-1.2	0.221	0.417	1
GO_AXON_REGENERATION	23	-0.37	-1.2	0.201	0.417	1
GO_POSITIVE_REGULATION_OF_BLOOD_CIRCULATION	93	-0.28	-1.2	0.157	0.417	1
GO_POSITIVE_REGULATION_OF_NUCLEOTIDE_CATABOLIC_PROCESS	17	-0.41	-1.2	0.239	0.419	1
GO_REGULATION_OF_REGULATED_SECRETORY_PATHWAY	128	-0.26	-1.2	0.138	0.422	1
GO_BHLH_TRANSCRIPTION_FACTOR_BINDING	28	-0.35	-1.2	0.209	0.422	1
GO_ANKYRIN_BINDING	20	-0.39	-1.2	0.237	0.422	1
GO_ENDOTHELIAL_CELL_DIFFERENTIATION	70	-0.29	-1.2	0.155	0.422	1
GO_PEPTIDE_RECEPTOR_ACTIVITY	133	-0.26	-1.19	0.126	0.424	1
GO_SUBPALLIUM_DEVELOPMENT	22	-0.38	-1.19	0.242	0.425	1
GO_SECRETORY_GRANULE_MEMBRANE	78	-0.28	-1.19	0.146	0.424	1
GO_BICARBONATE_TRANSMEMBRANE_TRANSPORTER_ACTIVITY	19	-0.38	-1.19	0.227	0.424	1
GO_CERAMIDE_METABOLIC_PROCESS	67	-0.29	-1.19	0.173	0.424	1
GO_MONOVALENT_INORGANIC_CATION_HOMEOSTASIS	122	-0.26	-1.19	0.147	0.423	1
GO_VESICLE_ORGANIZATION	276	-0.24	-1.19	0.089	0.423	1
GO_AMINO_ACID_TRANSPORT	124	-0.26	-1.19	0.125	0.425	1
GO_GOLGI_TO_PLASMA_MEMBRANE_PROTEIN_TRANSPORT	26	-0.36	-1.19	0.215	0.425	1
GO_COATED_PIT	66	-0.29	-1.19	0.179	0.425	1
GO_DEVELOPMENTAL_GROWTH_INVOLVED_IN_MORPHOGENESIS	103	-0.27	-1.19	0.132	0.426	1
GO_PHOSPHOLIPASE_C_ACTIVITY	31	-0.35	-1.19	0.199	0.426	1
GO_LYMPH_VESSEL_DEVELOPMENT	20	-0.39	-1.19	0.236	0.427	1
GO_G_PROTEIN_COUPLED_AMINE_RECEPTOR_ACTIVITY	50	-0.3	-1.19	0.187	0.428	1
GO_EPITHELIAL_CELL_DIFFERENTIATION_INVOLVED_IN_KIDNEY_DEVELOPMENT	24	-0.36	-1.19	0.22	0.429	1
GO_ESTABLISHMENT_OF_PROTEIN_LOCALIZATION_TO_GOLGI	19	-0.39	-1.19	0.226	0.43	1
GO_NEPHRON_EPITHELIUM_DEVELOPMENT	93	-0.28	-1.19	0.173	0.432	1
GO_RNA_POLYMERASE_II_TRANSCRIPTION_COREPRESSOR_ACTIVITY	26	-0.36	-1.19	0.226	0.432	1
GO_PROTEIN_DEACETYLASE_ACTIVITY	43	-0.31	-1.19	0.191	0.432	1
GO_INTRINSIC_COMPONENT_OF_MITOCHONDRIAL_OUTER_MEMBRANE	22	-0.38	-1.19	0.241	0.432	1
GO_CHONDROITIN_SULFATE_PROTEOGLYCAN_BIOSYNTHETIC_PROCES	30	-0.34	-1.18	0.223	0.435	1
GO_BLOC_1_COMPLEX	15	-0.42	-1.18	0.255	0.435	1
GO_POSITIVE_REGULATION_OF_PROTEIN_ACETYLATION	36	-0.33	-1.18	0.208	0.435	1
GO_HEART_TRABECULA_MORPHOGENESIS	26	-0.35	-1.18	0.207	0.437	1
GO_NEUROPEPTIDE_BINDING	22	-0.38	-1.18	0.235	0.437	1

GO_AGGRESOME	32	-0.34	-1.18	0.221	0.438	1
GO_POSITIVE_REGULATION_OF_PROTEIN_EXPORT_FROM_NUCLEUS	19	-0.38	-1.18	0.263	0.438	1
GO_GLUCOCORTICOID_METABOLIC_PROCESS	16	-0.41	-1.18	0.222	0.442	1
GO_MITOGEN_ACTIVATED_PROTEIN_KINASE_KINASE_BINDING	16	-0.4	-1.18	0.258	0.443	1
GO_POSITIVE_REGULATION_OF_LIPASE_ACTIVITY	66	-0.29	-1.18	0.194	0.444	1
GO_REGULATION_OF_CELLULAR_PH	75	-0.28	-1.18	0.176	0.444	1
GO_REGULATION_OF_RAS_PROTEIN_SIGNAL_TRANSDUCTION	179	-0.25	-1.18	0.149	0.446	1
GO_SENSORY_PERCEPTION_OF_TEMPERATURE_STIMULUS	21	-0.38	-1.18	0.242	0.445	1
GO_NEUROMUSCULAR_PROCESS_CONTROLLING_POSTURE	15	-0.42	-1.18	0.264	0.445	1
GO_PROTEIN_TARGETING_TO_LYSOSOME	15	-0.41	-1.18	0.246	0.446	1
GO_ION_ANTIPORTER_ACTIVITY	45	-0.31	-1.18	0.216	0.446	1
GO_NEGATIVE_REGULATION_OF_TRANSPORT	451	-0.22	-1.18	0.084	0.445	1
GO_REGULATION_OF_PHOSPHOLIPASE_ACTIVITY	64	-0.29	-1.18	0.201	0.446	1
GO_PERIPHERAL_NERVOUS_SYSTEM_DEVELOPMENT	69	-0.28	-1.17	0.182	0.449	1
GO_NEGATIVE_REGULATION_OF_ENDOTHELIAL_CELL_MIGRATION	39	-0.32	-1.17	0.21	0.449	1
GO_REGULATION_OF_MYOBLAST_DIFFERENTIATION	48	-0.31	-1.17	0.202	0.448	1
GO_CELLULAR_RESPONSE_TO_AMINO_ACID_STIMULUS	50	-0.3	-1.17	0.192	0.45	1
GO_CYTOPLASMIC_REGION	276	-0.23	-1.17	0.118	0.45	1
GO_SMOOTH_ENDOPLASMIC_RETICULUM	33	-0.33	-1.17	0.214	0.45	1
GO_POSITIVE_REGULATION_OF_ACTIN_CYTOSKELETON_REORGANIZAT	16	-0.41	-1.17	0.281	0.45	1
GO_GLOMERULUS_DEVELOPMENT	49	-0.3	-1.17	0.213	0.451	1
GO_REGULATION_OF_AUTOPHAGY	244	-0.24	-1.17	0.111	0.451	1
GO_AMINO_ACID_IMPORT	15	-0.4	-1.17	0.248	0.452	1
GO_GLIAL_CELL_MIGRATION	36	-0.33	-1.17	0.233	0.451	1
GO_MICROBODY_MEMBRANE	58	-0.29	-1.17	0.199	0.451	1
GO_REGULATION_OF_TRANSMEMBRANE_RECEPTOR_PROTEIN_SERINE_THREONINE_KINASE_SIGNALING_PATHWAY	205	-0.24	-1.17	0.134	0.451	1
GO_SECRETORY_VESICLE	452	-0.22	-1.17	0.071	0.452	1
GO_PLASMA_MEMBRANE_ORGANIZATION	202	-0.24	-1.17	0.133	0.452	1
GO_MYOSIN_V_BINDING	17	-0.39	-1.17	0.245	0.452	1
GO_REGULATION_OF_GLUCOSE_IMPORT_IN_RESPONSE_TO_INSULIN_STIMULUS	16	-0.41	-1.17	0.246	0.452	1
GO_NEGATIVE_REGULATION_OF_OSTEOCLAST_DIFFERENTIATION	25	-0.36	-1.17	0.228	0.453	1
GO_PHOSPHATIDYLINOSITOL_PHOSPHATE_BINDING	116	-0.25	-1.17	0.17	0.455	1
GO_BASAL_PLASMA_MEMBRANE	33	-0.33	-1.17	0.22	0.454	1
GO_ANION_CHANNEL_ACTIVITY	91	-0.27	-1.17	0.191	0.454	1
GO_ESTABLISHMENT_OF_MITOCHONDRION_LOCALIZATION	17	-0.39	-1.17	0.264	0.455	1
GO_HEART_VALVE_DEVELOPMENT	34	-0.33	-1.17	0.224	0.457	1
GO_GANGLIOSIDE_METABOLIC_PROCESS	26	-0.35	-1.17	0.244	0.457	1
GO_NEGATIVE_REGULATION_OF_HEART_CONTRACTION	22	-0.36	-1.17	0.262	0.456	1
GO_REGULATION_OF_HORMONE_LEVELS	472	-0.22	-1.17	0.089	0.456	1
GO_PHOSPHOTRANSFERASE_ACTIVITY_PHOSPHATE_GROUP_AS_ACCEPTOR	37	-0.33	-1.17	0.229	0.458	1
GO_CARDIAC_SEPTUM_DEVELOPMENT	85	-0.27	-1.16	0.177	0.458	1
GO_PURINE_CONTAINING_COMPOUND_CATABOLIC_PROCESS	50	-0.3	-1.16	0.216	0.459	1
GO_SENSORY_ORGAN_DEVELOPMENT	490	-0.22	-1.16	0.071	0.46	1
GO_PEPTIDE_HORMONE_RECEPTOR_BINDING	17	-0.4	-1.16	0.287	0.463	1
GO_REGULATION_OF_CHROMATIN_BINDING	17	-0.39	-1.16	0.262	0.463	1
GO_RESPONSE_TO_MUSCLE_ACTIVITY	20	-0.37	-1.16	0.247	0.463	1
GO_REGULATION_OF_RECEPTOR_INTERNALIZATION	37	-0.32	-1.16	0.234	0.464	1
GO_HEART_MORPHOGENESIS	211	-0.23	-1.16	0.14	0.465	1
GO_GAS_TRANSPORT	19	-0.38	-1.16	0.267	0.465	1
GO_ENZYME_ACTIVATOR_ACTIVITY	459	-0.22	-1.16	0.099	0.467	1
GO_RESPONSE_TO_NERVE_GROWTH_FACTOR	37	-0.32	-1.16	0.212	0.467	1
GO_REGULATION_OF_BMP_SIGNALING_PATHWAY	76	-0.28	-1.16	0.183	0.468	1
GO_SPERM_MOTILITY	48	-0.3	-1.16	0.241	0.47	1
GO_REGULATION_OF_VASCULAR_ENDOTHELIAL_GROWTH_FACTOR_RECEPTOR_SIGNALING_PATHWAY	27	-0.34	-1.16	0.257	0.473	1
GO_CELL_LEADING_EDGE	346	-0.22	-1.15	0.107	0.476	1
GO_PHOSPHATIDYLINOSITOL_KINASE_ACTIVITY	51	-0.3	-1.15	0.224	0.477	1
GO_NEGATIVE_REGULATION_OF_ACTIN_FILAMENT_DEPOLYMERIZATION	35	-0.32	-1.15	0.244	0.477	1
GO_PHOSPHATIDYLINOSITOL_METABOLIC_PROCESS	193	-0.24	-1.15	0.161	0.477	1
GO_REGULATION_OF_OSSIFICATION	177	-0.24	-1.15	0.148	0.477	1
GO_NEGATIVE_CHEMOTAXIS	39	-0.31	-1.15	0.241	0.477	1
GO_REGULATION_OF_EXOCYTOSIS	182	-0.24	-1.15	0.159	0.477	1
GO_RESPONSE_TO_PH	42	-0.31	-1.15	0.221	0.48	1
GO_REGULATION_OF_SKELETAL_MUSCLE_TISSUE_DEVELOPMENT	50	-0.3	-1.15	0.228	0.482	1
GO_ACTOMYOSIN_STRUCTURE_ORGANIZATION	77	-0.27	-1.15	0.203	0.481	1
GO_POSITIVE_REGULATION_OF_HEART_GROWTH	26	-0.35	-1.15	0.246	0.482	1
GO_NEGATIVE_REGULATION_OF_CATION_TRANSMEMBRANE_TRANSPO	61	-0.29	-1.15	0.242	0.482	1
GO_REGULATION_OF_ENDOCRINE_PROCESS	47	-0.3	-1.15	0.243	0.483	1
GO_EYE_DEVELOPMENT	323	-0.22	-1.15	0.125	0.484	1
GO_MUSCLE_ORGAN_DEVELOPMENT	277	-0.23	-1.15	0.153	0.484	1
GO_POSITIVE_REGULATION_OF_PHOSPHOPROTEIN_PHOSPHATASE_ACTIVITY	16	-0.39	-1.15	0.274	0.486	1
GO_POSITIVE_REGULATION_OF_ORGAN_GROWTH	37	-0.32	-1.15	0.251	0.488	1
GO_REGULATION_OF_RENAL_SYSTEM_PROCESS	38	-0.32	-1.15	0.262	0.489	1
GO_POSITIVE_REGULATION_OF_PATHWAY_RESTRICTED_SMAD_PROTEIN_PHOSPHORYLATION	48	-0.3	-1.14	0.232	0.491	1
GO_VENTRAL_SPINAL_CORD_DEVELOPMENT	46	-0.3	-1.14	0.239	0.494	1
GO_GLANDULAR_EPITHELIAL_CELL_DEVELOPMENT	18	-0.38	-1.14	0.282	0.497	1
GO_REGULATION_OF_ORGANIC_ACID_TRANSPORT	48	-0.3	-1.14	0.258	0.498	1
GO_OLIGOSACCHARIDE_METABOLIC_PROCESS	65	-0.28	-1.14	0.274	0.498	1
GO_NEURAL_NUCLEUS_DEVELOPMENT	65	-0.28	-1.14	0.217	0.498	1
GO_DRUG_TRANSPORTER_ACTIVITY	21	-0.35	-1.14	0.267	0.497	1
GO_VACUOLAR_ACIDIFICATION	15	-0.39	-1.14	0.286	0.497	1
GO_REGULATION_OF_MUSCLE_TISSUE_DEVELOPMENT	101	-0.26	-1.14	0.206	0.497	1

GO_NEGATIVE_REGULATION_OF_CALCIUM_ION_TRANSPORT	50	-0.3	-1.14	0.223	0.497	1
GO_PRE_AUTOPHAGOSOMAL_STRUCTURE	26	-0.35	-1.14	0.274	0.497	1
GO_RESPONSE_TO_PEPTIDE	400	-0.22	-1.14	0.135	0.497	1
GO_REGULATION_OF_LIPASE_ACTIVITY	83	-0.27	-1.14	0.219	0.498	1
GO_NEUROPEPTIDE_RECEPTOR_BINDING	28	-0.34	-1.14	0.28	0.501	1
GO_CARBOHYDRATE_HOMEOSTASIS	168	-0.24	-1.14	0.198	0.504	1
GO_NEURON_PROJECTION_REGENERATION	32	-0.33	-1.14	0.268	0.504	1
GO_OUTFLOW_TRACT_MORPHOGENESIS	56	-0.29	-1.14	0.254	0.505	1
GO_ENTRAINMENT_OF_CIRCADIAN_CLOCK_BY_PHOTOPERIOD	19	-0.37	-1.14	0.282	0.505	1
GO_ADRENERGIC_RECEPTOR_ACTIVITY	16	-0.39	-1.14	0.282	0.505	1
GO_EXTRACELLULAR_MATRIX_ASSEMBLY	16	-0.4	-1.14	0.272	0.505	1
GO_REGULATION_OF_ALTERNATIVE_MRNA_SPLICING_VIA_SPLICEOSOM	28	-0.33	-1.13	0.24	0.506	1
GO_REGULATION_OF_VASOCONSTRICTION	66	-0.28	-1.13	0.235	0.506	1
GO_CELL_CORTEX_REGION	16	-0.4	-1.13	0.297	0.505	1
GO_REGULATION_OF_GLUCOSE_IMPORT	59	-0.28	-1.13	0.252	0.506	1
GO_GOLGI_TO_VACUOLE_TRANSPORT	27	-0.34	-1.13	0.303	0.508	1
GO_PHOSPHATIDYLINOSITOL_4_PHOSPHATE_BINDING	20	-0.37	-1.13	0.274	0.514	1
GO_PHOSPHATIDYLINOSITOL_3_5_BISPHOSPHATE_BINDING	21	-0.36	-1.13	0.291	0.515	1
GO_CYTOSOLIC_CALCIUM_ION_TRANSPORT	53	-0.29	-1.13	0.276	0.515	1
GO_NAD_DEPENDENT_PROTEIN_DEACETYLASE_ACTIVITY	17	-0.37	-1.13	0.291	0.515	1
GO_REGULATION_OF_PEPTIDE_TRANSPORT	253	-0.23	-1.13	0.165	0.516	1
GO_POSITIVE_REGULATION_OF_ENDOTHELIAL_CELL_MIGRATION	67	-0.27	-1.13	0.237	0.517	1
GO_FILOPODIUM	94	-0.26	-1.13	0.241	0.52	1
GO_SPINAL_CORD_MOTOR_NEURON_DIFFERENTIATION	34	-0.32	-1.13	0.275	0.523	1
GO_MICROBODY_PART	92	-0.26	-1.13	0.25	0.523	1
GO_REPLACEMENT_OSSIFICATION	26	-0.34	-1.12	0.291	0.524	1
GO_ACTIVATION_OF_PROTEIN_KINASE_B_ACTIVITY	22	-0.36	-1.12	0.315	0.525	1
GO_OLIGODENDROCYTE_DEVELOPMENT	34	-0.32	-1.12	0.258	0.524	1
GO_MRNA_SPLICE_SITE_SELECTION	19	-0.36	-1.12	0.297	0.525	1
GO_PROTEIN_COMPLEX_SCAFFOLD	64	-0.28	-1.12	0.237	0.526	1
GO_TRANSCRIPTION_FACTOR_ACTIVITY_DIRECT_LIGAND_REGULATED_SEQUENCE_SPECIFIC_DNA_BINDING	48	-0.29	-1.12	0.281	0.527	1
GO_PHOSPHATIDYLINOSITOL_3_4_BISPHOSPHATE_BINDING	20	-0.36	-1.12	0.302	0.531	1
GO_WNT_SIGNALING_PATHWAY_CALCIUM_MODULATING_PATHWAY	39	-0.31	-1.12	0.283	0.531	1
GO_NEGATIVE_REGULATION_OF_NEURON_DEATH	170	-0.23	-1.12	0.208	0.533	1
GO_POLYSACCHARIDE_METABOLIC_PROCESS	80	-0.27	-1.12	0.248	0.533	1
GO_MYOTUBE_CELL_DEVELOPMENT	30	-0.32	-1.12	0.312	0.534	1
GO_POSITIVE_REGULATION_OF_HEART_RATE	22	-0.36	-1.12	0.319	0.534	1
GO_POSITIVE_REGULATION_OF_ENDOTHELIAL_CELL_DIFFERENTIATION	15	-0.39	-1.12	0.325	0.533	1
GO_RNA_POLYMERASE_II_TRANSCRIPTION_COFACTOR_ACTIVITY	91	-0.26	-1.12	0.234	0.533	1
GO_POSITIVE_REGULATION_OF_INSULIN_SECRETION	63	-0.28	-1.12	0.271	0.533	1
GO_R_SMAD_BINDING	23	-0.34	-1.12	0.32	0.533	1
GO_POSITIVE_REGULATION_OF_PEPTIDYL_SERINE_PHOSPHORYLATION	88	-0.26	-1.12	0.264	0.533	1
GO_SMAD_BINDING	70	-0.27	-1.12	0.273	0.534	1
GO_KINASE_INHIBITOR_ACTIVITY	89	-0.26	-1.12	0.26	0.536	1
GO_HORMONE_TRANSPORT	76	-0.27	-1.12	0.26	0.536	1
GO_MITOGEN_ACTIVATED_PROTEIN_KINASE_BINDING	24	-0.34	-1.12	0.304	0.538	1
GO_MAINTENANCE_OF_PROTEIN_LOCALIZATION_IN_ORGANELLE	29	-0.33	-1.11	0.312	0.539	1
GO_REGULATION_OF_ENDOTHELIAL_CELL_MIGRATION	114	-0.25	-1.11	0.246	0.541	1
GO_REGULATION_OF_SEQUESTERING_OF_CALCIUM_ION	107	-0.25	-1.11	0.235	0.541	1
GO_REGULATION_OF_SMOOTH_MUSCLE_CONTRACTION	60	-0.28	-1.11	0.281	0.542	1
GO_NEGATIVE_REGULATION_OF_CELL_MORPHOGENESIS_INVOLVED_IN_DIFFERENTIATION	117	-0.25	-1.11	0.222	0.542	1
GO_POSITIVE_REGULATION_OF_SMALL_GTPASE_MEDIATED_SIGNAL_TRANSDUCTION	39	-0.3	-1.11	0.293	0.545	1
GO_NEGATIVE_REGULATION_OF_OSTEOBLAST_DIFFERENTIATION	40	-0.3	-1.11	0.289	0.545	1
GO_ACTIVATION_OF_MAPKK_ACTIVITY	51	-0.29	-1.11	0.293	0.545	1
GO_REGULATION_OF_GLYCOGEN_METABOLIC_PROCESS	35	-0.32	-1.11	0.289	0.545	1
GO_HORMONE_MEDIATED_SIGNALING_PATHWAY	155	-0.23	-1.11	0.237	0.547	1
GO_REGULATION_OF_MUSCLE_HYPERTROPHY	34	-0.32	-1.11	0.323	0.547	1
GO_SYNAPTIC_VESICLE_CYTOSKELETAL_TRANSPORT	15	-0.38	-1.11	0.335	0.549	1
GO_REGULATION_OF_RELEASE_OF_SEQUESTERED_CALCIUM_ION_INTO_CYTOSOL	75	-0.27	-1.11	0.264	0.549	1
GO_POSITIVE_REGULATION_OF_REGULATED_SECRETORY_PATHWAY	49	-0.29	-1.11	0.276	0.549	1
GO_NEGATIVE_REGULATION_OF_PROTEIN_KINASE_B_SIGNALING	36	-0.3	-1.11	0.31	0.549	1
GO_ATPASE_ACTIVITY_COUPLED_TO_MOVEMENT_OF_SUBSTANCES	121	-0.25	-1.11	0.267	0.551	1
GO_LONG_TERM_SYNAPTIC_DEPRESSION	15	-0.38	-1.11	0.331	0.552	1
GO_NEGATIVE_REGULATION_OF_POTASSIUM_ION_TRANSMEMBRANE_TRANSPORT	21	-0.35	-1.11	0.329	0.552	1
GO_GOLGI_TO_ENDOSOME_TRANSPORT	19	-0.37	-1.11	0.322	0.552	1
GO_EMBRYONIC_HEART_TUBE_DEVELOPMENT	72	-0.27	-1.11	0.276	0.552	1
GO_GOLGI_TO_PLASMA_MEMBRANE_TRANSPORT	41	-0.3	-1.11	0.31	0.552	1
GO_FATTY_ACYL_COA_BINDING	30	-0.32	-1.11	0.332	0.552	1
GO_NARROW_PORE_CHANNEL_ACTIVITY	17	-0.38	-1.1	0.32	0.552	1
GO_REGULATION_OF_ENERGY_HOMEOSTASIS	17	-0.37	-1.1	0.321	0.552	1
GO_S_ADENOSYLMETHIONINE_METABOLIC_PROCESS	18	-0.37	-1.1	0.325	0.558	1
GO_GENETIC_IMPRINTING	20	-0.36	-1.1	0.308	0.56	1
GO_REGULATION_OF_VESICLE_MEDIATED_TRANSPORT	456	-0.21	-1.1	0.192	0.562	1
GO_SYMPATHETIC_NERVOUS_SYSTEM_DEVELOPMENT	21	-0.35	-1.1	0.324	0.565	1
GO_REGULATION_OF_NEURON_APOPTOTIC_PROCESS	191	-0.23	-1.1	0.241	0.564	1
GO_POST_GOLGI_VESICLE_MEDIATED_TRANSPORT	83	-0.26	-1.1	0.262	0.566	1
GO_REGULATION_OF_ANDROGEN_RECEPTOR_SIGNALING_PATHWAY	22	-0.35	-1.1	0.333	0.571	1
GO_MYOBLAST_FUSION	20	-0.35	-1.1	0.327	0.571	1
GO_OXYGEN_TRANSPORT	15	-0.39	-1.1	0.339	0.571	1
GO_ENDOCARDIAL_CUSHION_MORPHOGENESIS	22	-0.35	-1.09	0.318	0.573	1
GO_REGULATION_OF_VASODILATION	48	-0.29	-1.09	0.295	0.574	1
GO_NEGATIVE_REGULATION_OF_PROTEIN_COMPLEX_ASSEMBLY	106	-0.24	-1.09	0.285	0.575	1

GO_MEMBRANE_DOCKING	68	-0.26	-1.09	0.293	0.578	1
GO_NEGATIVE_REGULATION_OF_INTRACELLULAR_PROTEIN_TRANSPO	94	-0.25	-1.09	0.287	0.578	1
GO_NEGATIVE_REGULATION_OF_LIPID_CATABOLIC_PROCESS	19	-0.35	-1.09	0.346	0.577	1
GO_POLYSACCHARIDE_CATABOLIC_PROCESS	24	-0.33	-1.09	0.335	0.578	1
GO_DORSAL_VENTRAL_NEURAL_TUBE_PATTERNING	17	-0.36	-1.09	0.344	0.582	1
GO_REGULATION_OF_ANATOMICAL_STRUCTURE_SIZE	466	-0.21	-1.09	0.208	0.584	1
GO_ENDOCARDIAL_CUSHION_DEVELOPMENT	32	-0.31	-1.09	0.313	0.584	1
GO_DEPHOSPHORYLATION	280	-0.22	-1.09	0.227	0.586	1
GO_POSITIVE_REGULATION_OF_ACTIN_NUCLEATION	15	-0.37	-1.09	0.357	0.589	1
GO_DOPAMINE_METABOLIC_PROCESS	25	-0.33	-1.09	0.323	0.589	1
GO_CELL_CORTEX	227	-0.22	-1.08	0.246	0.593	1
GO_SPERMATID_DIFFERENTIATION	124	-0.24	-1.08	0.286	0.593	1
GO_NEGATIVE_REGULATION_OF_TOR_SIGNALING	29	-0.33	-1.08	0.347	0.593	1
GO_MICROBODY	132	-0.24	-1.08	0.301	0.6	1
GO_REGULATION_OF_CALCIUM_ION_TRANSPORT_INTO_CYTOSOL	92	-0.25	-1.08	0.295	0.6	1
GO_REGULATION_OF_NON_CANONICAL_WNT_SIGNALING_PATHWAY	19	-0.36	-1.08	0.352	0.6	1
GO_SPERM_PART	134	-0.23	-1.08	0.283	0.601	1
GO_REGULATION_OF_MUSCLE_CELL_DIFFERENTIATION	151	-0.23	-1.08	0.276	0.6	1
GO_DETECTION_OF_TEMPERATURE_STIMULUS	17	-0.36	-1.08	0.372	0.6	1
GO_POSITIVE_REGULATION_OF_MICROTUBULE_POLYMERIZATION	18	-0.36	-1.08	0.331	0.601	1
GO_EXTRINSIC_COMPONENT_OF_CYTOPLASMIC_SIDE_OF_PLASMA_MEMBRANE	97	-0.25	-1.08	0.313	0.606	1
GO_GLIAL_CELL_DEVELOPMENT	76	-0.26	-1.08	0.312	0.605	1
GO_CELL_DIFFERENTIATION_IN_SPINAL_CORD	54	-0.27	-1.08	0.313	0.606	1
GO_EMBRYONIC_HEMOPOIESIS	20	-0.34	-1.08	0.36	0.606	1
GO_REGULATION_OF_CAMP_MEDIATED_SIGNALING	23	-0.34	-1.08	0.366	0.607	1
GO_CELL_RECOGNITION	128	-0.23	-1.08	0.311	0.607	1
GO_BASAL_PART_OF_CELL	51	-0.28	-1.08	0.346	0.607	1
GO_METANEPHRIC_EPITHELIUM_DEVELOPMENT	20	-0.35	-1.08	0.331	0.607	1
GO_CORTICAL_CYTOSKELETON	80	-0.25	-1.08	0.316	0.608	1
GO_NUCLEOTIDE_KINASE_ACTIVITY	22	-0.34	-1.08	0.341	0.608	1
GO_NEGATIVE_REGULATION_OF_CALCIUM_MEDIATED_SIGNALING	19	-0.35	-1.08	0.352	0.607	1
GO_REGULATION_OF_ORGANELLE_ASSEMBLY	143	-0.23	-1.08	0.289	0.608	1
GO_REGULATION_OF_FATTY_ACID_BETA_OXIDATION	15	-0.37	-1.07	0.375	0.608	1
GO_POLYOL_METABOLIC_PROCESS	95	-0.25	-1.07	0.329	0.608	1
GO_REGULATION_OF_COLLATERAL_SPROUTING	17	-0.37	-1.07	0.383	0.608	1
GO_ESTABLISHMENT_OF_PROTEIN_LOCALIZATION_TO_PLASMA_MEMBR	90	-0.25	-1.07	0.317	0.608	1
GO_GLOMERULAR_EPITHELIUM_DEVELOPMENT	19	-0.35	-1.07	0.355	0.608	1
GO_RENAL_TUBULE_DEVELOPMENT	78	-0.26	-1.07	0.326	0.608	1
GO_NEGATIVE_REGULATION_OF_OXIDATIVE_STRESS_INDUCED_INTRINSIC_APOPTOTIC_SIGNALING_PATHWAY	21	-0.34	-1.07	0.367	0.611	1
GO_REGULATION_OF_CARDIAC_MUSCLE_CELL_PROLIFERATION	29	-0.31	-1.07	0.343	0.615	1
GO_RELAXATION_OF_MUSCLE	20	-0.35	-1.07	0.379	0.614	1
GO_NEGATIVE_REGULATION_OF_MYOBLAST_DIFFERENTIATION	24	-0.33	-1.07	0.364	0.614	1
GO_INOSITOL_PHOSPHATE_MEDIATED_SIGNALING	18	-0.37	-1.07	0.36	0.614	1
GO_MODIFIED_AMINO_ACID_TRANSMEMBRANE_TRANSPORTER_ACTIVI	16	-0.37	-1.07	0.375	0.613	1
GO_POSITIVE_REGULATION_OF_PROTEIN_TARGETING_TO_MEMBRANE	23	-0.33	-1.07	0.356	0.613	1
GO_FILOPODIUM_MEMBRANE	18	-0.36	-1.07	0.346	0.613	1
GO_MYOTUBE_DIFFERENTIATION	57	-0.27	-1.07	0.333	0.613	1
GO_PHAGOSOME_ACIDIFICATION	27	-0.32	-1.07	0.33	0.615	1
GO_CELL_SUBSTRATE_ADHESION	161	-0.22	-1.07	0.306	0.616	1
GO_PALLIUM_DEVELOPMENT	151	-0.23	-1.07	0.311	0.616	1
GO_REGULATION_OF_ACTIN_FILAMENT_DEPOLYMERIZATION	47	-0.28	-1.07	0.327	0.616	1
GO_POSITIVE_REGULATION_OF_AUTOPHAGY	74	-0.25	-1.07	0.347	0.615	1
GO_L_AMINO_ACID_TRANSPORT	58	-0.27	-1.07	0.356	0.618	1
GO_E_BOX_BINDING	34	-0.3	-1.07	0.375	0.617	1
GO_ACETYLCHOLINE_RECEPTOR_ACTIVITY	30	-0.31	-1.07	0.348	0.619	1
GO_PHOSPHOLIPID_TRANSLOCATING_ATPASE_ACTIVITY	16	-0.37	-1.07	0.366	0.621	1
GO_REGULATION_OF_CELL_GROWTH	386	-0.2	-1.07	0.27	0.62	1
GO_RNA_POLYMERASE_II_TRANSCRIPTION_FACTOR_BINDING	104	-0.24	-1.07	0.321	0.621	1
GO_PHOTOTRANSDUCTION_VISIBLE_LIGHT	20	-0.34	-1.06	0.386	0.622	1
GO_CELL_MATRIX_ADHESION	118	-0.24	-1.06	0.311	0.622	1
GO_ACETYLGALACTOSAMINYLTRANSFERASE_ACTIVITY	34	-0.3	-1.06	0.353	0.622	1
GO_HISTONE_DEACETYLASE_COMPLEX	61	-0.27	-1.06	0.346	0.622	1
GO_COSTAMERE	19	-0.35	-1.06	0.365	0.621	1
GO_SCHWANN_CELL_DEVELOPMENT	26	-0.32	-1.06	0.377	0.621	1
GO_CLATHRIN_VESICLE_COAT	23	-0.33	-1.06	0.352	0.621	1
GO_REPRESSING_TRANSCRIPTION_FACTOR_BINDING	56	-0.27	-1.06	0.351	0.62	1
GO_PERIPHERAL_NERVOUS_SYSTEM_AXON_ENSHEATHMENT	22	-0.34	-1.06	0.362	0.62	1
GO_ACTIVE_ION_TRANSMEMBRANE_TRANSPORTER_ACTIVITY	169	-0.22	-1.06	0.296	0.619	1
GO_PROTEIN_LOCALIZATION_TO_CELL_SURFACE	22	-0.34	-1.06	0.352	0.619	1
GO_TRANSMEMBRANE_RECEPTOR_PROTEIN_SERINE_THREONINE_KINASE_SIGNALING_PATHWAY	189	-0.22	-1.06	0.314	0.62	1
GO_SERTOLI_CELL_DEVELOPMENT	15	-0.37	-1.06	0.364	0.623	1
GO_NEGATIVE_REGULATION_OF_INTRACELLULAR_TRANSPORT	140	-0.23	-1.06	0.32	0.624	1
GO_POSITIVE_REGULATION_OF_ADHERENS_JUNCTION_ORGANIZATION	21	-0.34	-1.06	0.374	0.624	1
GO_REGULATION_OF_PHOSPHATIDYLINOSITOL_3_KINASE_SIGNALING	138	-0.23	-1.06	0.325	0.624	1
GO_POSITIVE_REGULATION_OF_CELL_MORPHOGENESIS_INVOLVED_IN_DIFFERENTIATION	161	-0.23	-1.06	0.328	0.624	1
GO_ASPARTATE_FAMILY_AMINO_ACID_METABOLIC_PROCESS	55	-0.27	-1.06	0.348	0.625	1
GO_RESPONSE_TO_AMINE	48	-0.28	-1.06	0.34	0.626	1
GO_REGULATION_OF_NEURON_DEATH	251	-0.21	-1.06	0.317	0.628	1
GO_CYTOPLASMIC_DYNEIN_COMPLEX	15	-0.37	-1.06	0.388	0.63	1
GO_14_3_3_PROTEIN_BINDING	19	-0.35	-1.06	0.409	0.63	1
GO_ORGANOPHOSPHATE_CATABOLIC_PROCESS	112	-0.24	-1.06	0.349	0.631	1
GO_RESPONSE_TO_COCAINE	46	-0.28	-1.06	0.349	0.63	1
GO_REGULATION_OF_DEPHOSPHORYLATION	147	-0.23	-1.06	0.322	0.63	1

GO_NUCLEOSIDE_PHOSPHATE_CATABOLIC_PROCESS	69	-0.26	-1.06	0.366	0.63	1
GO_ATPASE_BINDING	75	-0.25	-1.06	0.344	0.63	1
GO_NEGATIVE_REGULATION_OF_NUCLEOCYTOPLASMIC_TRANSPORT	70	-0.25	-1.06	0.348	0.63	1
GO_REGULATION_OF_SYNAPTIC_TRANSMISSION_DOPAMINERGIC	17	-0.36	-1.06	0.39	0.63	1
GO_ACYLGLYCEROL_HOMEOSTASIS	29	-0.31	-1.06	0.371	0.631	1
GO_EMBRYONIC_HEART_TUBE_MORPHOGENESIS	61	-0.26	-1.05	0.361	0.632	1
GO_INNERVATION	23	-0.34	-1.05	0.367	0.632	1
GO_BRANCHING_INVOLVED_IN_URETERIC_BUD_MORPHOGENESIS	44	-0.28	-1.05	0.379	0.632	1
GO_NEURAL_TUBE_PATTERNING	34	-0.3	-1.05	0.373	0.633	1
GO_METALLOCARBOXYPEPTIDASE_ACTIVITY	27	-0.31	-1.05	0.383	0.635	1
GO_RESPONSE_TO_NICOTINE	51	-0.27	-1.05	0.371	0.638	1
GO_CELLULAR_RESPONSE_TO_CADMIUM_ION	15	-0.36	-1.05	0.384	0.64	1
GO_CELLULAR_RESPONSE_TO_CALCIUM_ION	49	-0.27	-1.05	0.362	0.641	1
GO_CLATHRIN_COATED_VESICLE_MEMBRANE	79	-0.25	-1.05	0.355	0.646	1
GO_ACTIN_FILAMENT_BASED_PROCESS	445	-0.2	-1.05	0.297	0.645	1
GO_POSITIVE_REGULATION_OF_ANION_TRANSPORT	57	-0.26	-1.05	0.395	0.644	1
GO_NEGATIVE_REGULATION_OF_ACTIN_FILAMENT_POLYMERIZATION	44	-0.28	-1.05	0.353	0.646	1
GO_NEGATIVE_REGULATION_OF_STAT_CASCADE	44	-0.28	-1.05	0.369	0.646	1
GO_CHANNEL_INHIBITOR_ACTIVITY	37	-0.29	-1.05	0.386	0.647	1
GO_POSITIVE_REGULATION_OF_GROWTH	234	-0.21	-1.05	0.346	0.646	1
GO_REGULATION_OF_CALCIUM_MEDIATED_SIGNALING	76	-0.25	-1.05	0.356	0.646	1
GO_POSITIVE_REGULATION_OF_MUSCLE_CELL_DIFFERENTIATION	83	-0.25	-1.05	0.368	0.65	1
GO_PHAGOSOME_MATURATION	38	-0.29	-1.04	0.383	0.653	1
GO_REGULATION_OF_ENDOTHELIAL_CELL_CHEMOTAXIS	17	-0.34	-1.04	0.391	0.655	1
GO_NEGATIVE_REGULATION_OF_AUTOPHAGY	53	-0.27	-1.04	0.39	0.657	1
GO_CELLULAR_AMINO_ACID_CATABOLIC_PROCESS	111	-0.23	-1.04	0.376	0.657	1
GO_CELL_MATURATION	129	-0.23	-1.04	0.367	0.662	1
GO_BAF_TYPE_COMPLEX	23	-0.33	-1.04	0.418	0.663	1
GO_PHOTOTRANSDUCTION	42	-0.28	-1.04	0.414	0.662	1
GO_REGULATION_OF_OSTEOBLAST_DIFFERENTIATION	112	-0.23	-1.04	0.364	0.663	1
GO_LONG_CHAIN_FATTY_ACID_TRANSPORT	42	-0.28	-1.04	0.383	0.663	1
GO_REGULATION_OF_POSITIVE_CHEMOTAXIS	24	-0.32	-1.04	0.373	0.664	1
GO_TRANSFORMING_GROWTH_FACTOR_BETA_RECEPTOR_BINDING	50	-0.27	-1.04	0.396	0.664	1
GO_CATECHOLAMINE_BIOSYNTHETIC_PROCESS	18	-0.34	-1.04	0.397	0.665	1
GO_TRANSFORMING_GROWTH_FACTOR_BETA_BINDING	16	-0.36	-1.04	0.409	0.664	1
GO_SMALL_GTPASE_MEDIATED_SIGNAL_TRANSDUCTION	345	-0.2	-1.04	0.343	0.666	1
GO_POSITIVE_REGULATION_OF_NUCLEOSIDE_METABOLIC_PROCESS	24	-0.32	-1.04	0.404	0.667	1
GO_CELL_CYCLE_ARREST	150	-0.22	-1.04	0.361	0.669	1
GO_NEURAL_CREST_CELL_DIFFERENTIATION	75	-0.25	-1.03	0.377	0.672	1
GO_POSITIVE_REGULATION_OF_PHOSPHATIDYLINOSITOL_3_KINASE_SIGNALING	62	-0.26	-1.03	0.389	0.673	1
GO_FATTY_ACID_BETA_OXIDATION	49	-0.27	-1.03	0.405	0.679	1
GO_CELL_CORTEX_PART	110	-0.23	-1.03	0.388	0.679	1
GO_QUATERNARY_AMMONIUM_GROUP_TRANSPORT	18	-0.34	-1.03	0.409	0.679	1
GO_RESPONSE_TO_ELECTRICAL_STIMULUS	43	-0.28	-1.03	0.407	0.679	1
GO_POSITIVE_REGULATION_OF_SODIUM_ION_TRANSMEMBRANE_TRANSPORT	17	-0.34	-1.03	0.409	0.679	1
GO_MODIFIED_AMINO_ACID_BINDING	64	-0.25	-1.03	0.408	0.679	1
GO_CELLULAR_RESPONSE_TO_PH	19	-0.34	-1.03	0.407	0.679	1
GO_CHOLESTEROL_EFFLUX	26	-0.31	-1.03	0.409	0.678	1
GO_PROTEIN_TYROSINE_KINASE_BINDING	54	-0.26	-1.03	0.434	0.681	1
GO_NEGATIVE_REGULATION_OF_LOCOMOTION	260	-0.2	-1.03	0.376	0.68	1
GO_ATRIAL_SEPTUM_DEVELOPMENT	18	-0.34	-1.03	0.41	0.684	1
GO_PROTEIN_STABILIZATION	130	-0.23	-1.03	0.398	0.684	1
GO_POSITIVE_REGULATION_OF_HEART_CONTRACTION	35	-0.29	-1.03	0.423	0.684	1
GO_CARDIAC_CHAMBER_DEVELOPMENT	144	-0.22	-1.03	0.388	0.684	1
GO_GOLGI_VESICLE_TRANSPORT	313	-0.2	-1.03	0.381	0.684	1
GO_REGULATION_OF_MUSCLE_ADAPTATION	60	-0.26	-1.03	0.402	0.684	1
GO_GASTRULATION_WITH_MOUTH_FORMING_SECOND	28	-0.3	-1.03	0.428	0.684	1
GO_REGULATION_OF_BIOMINERAL_TISSUE_DEVELOPMENT	74	-0.24	-1.03	0.403	0.684	1
GO_ADHERENS_JUNCTION_ASSEMBLY	34	-0.3	-1.02	0.421	0.688	1
GO_IMPORT_INTO_CELL	36	-0.28	-1.02	0.392	0.687	1
GO_PHOTORECEPTOR_OUTER_SEGMENT_MEMBRANE	15	-0.36	-1.02	0.42	0.687	1
GO_RESPONSE_TO_INSULIN	203	-0.21	-1.02	0.394	0.696	1
GO_PROLINE_RICH_REGION_BINDING	19	-0.34	-1.02	0.418	0.698	1
GO_LYSOSOME_LOCALIZATION	21	-0.33	-1.02	0.437	0.699	1
GO_HYDROGEN_EXPORTING_ATPASE_ACTIVITY	27	-0.3	-1.02	0.442	0.7	1
GO_REGULATION_OF_ACTIVIN_RECEPTOR_SIGNALING_PATHWAY	25	-0.31	-1.02	0.431	0.7	1
GO_INOSITOL_LIPID_MEDIATED_SIGNALING	123	-0.22	-1.02	0.414	0.699	1
GO_NEGATIVE_REGULATION_OF_TRANSPORTER_ACTIVITY	64	-0.25	-1.02	0.402	0.699	1
GO_VACUOLAR_TRANSPORT	243	-0.2	-1.02	0.412	0.7	1
GO_POSITIVE_REGULATION_OF_CALCIUM_ION_TRANSPORT	106	-0.23	-1.02	0.424	0.699	1
GO_NEGATIVE_REGULATION_OF_EPITHELIAL_CELL_MIGRATION	53	-0.26	-1.02	0.435	0.703	1
GO_EXTRINSIC_COMPONENT_OF_MEMBRANE	246	-0.2	-1.02	0.411	0.705	1
GO_ATP_HYDROLYSIS_COUPLED_TRANSMEMBRANE_TRANSPORT	36	-0.28	-1.02	0.433	0.705	1
GO_EARLY_ENDOSOME_MEMBRANE	106	-0.23	-1.02	0.422	0.704	1
GO_GABAERGIC_NEURON_DIFFERENTIATION	15	-0.35	-1.02	0.417	0.705	1
GO_REGULATION_OF_LIPOPROTEIN_LIPASE_ACTIVITY	15	-0.36	-1.01	0.459	0.71	1
GO_NEGATIVE_REGULATION_OF_DEPHOSPHORYLATION	70	-0.25	-1.01	0.433	0.71	1
GO_RESPONSE_TO_ACETYLCHOLINE	18	-0.35	-1.01	0.436	0.71	1
GO_RETINAL_GANGLION_CELL_AXON_GUIDANCE	18	-0.33	-1.01	0.428	0.71	1
GO_REGULATION_OF_PROTEIN_HOMOOLIGOMERIZATION	16	-0.35	-1.01	0.444	0.71	1
GO_CARDIAC_MUSCLE_CELL_DIFFERENTIATION	74	-0.24	-1.01	0.433	0.709	1
GO_REGULATION_OF_TRANSCRIPTION_INVOLVED_IN_CELL_FATE_COMMITMENT	20	-0.32	-1.01	0.448	0.709	1
GO_CIRCADIAN_REGULATION_OF_GENE_EXPRESSION	57	-0.26	-1.01	0.43	0.713	1
GO_ENTRAINMENT_OF_CIRCADIAN_CLOCK	26	-0.31	-1.01	0.45	0.713	1

GO_REGULATION_OF_RECEPTOR_MEDIATED_ENDOCYTOSIS	78	-0.24	-1.01	0.43	0.715	1
GO_ER_TO_GOLGI_VESICLE_MEDIATED_TRANSPORT	163	-0.21	-1.01	0.427	0.715	1
GO_RESPONSE_TO_ISOQUINOLINE_ALKALOID	30	-0.29	-1.01	0.441	0.715	1
GO_ORGANELLE_SUBCOMPARTMENT	301	-0.2	-1.01	0.41	0.715	1
GO_REGULATION_OF_INTRACELLULAR_STEROID_HORMONE_RECEPTOR_SIGNALING_PATHWAY	58	-0.25	-1.01	0.423	0.717	1
GO_SULFUR_COMPOUND_BINDING	231	-0.2	-1.01	0.429	0.716	1
GO_NEGATIVE_REGULATION_OF_SECRETION	196	-0.21	-1.01	0.433	0.72	1
GO_CELLULAR_SODIUM_ION_HOMEOSTASIS	18	-0.33	-1.01	0.444	0.722	1
GO_NEGATIVE_REGULATION_OF_MUSCLE_CELL_APOPTOTIC_PROCESS	30	-0.29	-1.01	0.455	0.722	1
GO_MYOSIN_COMPLEX	67	-0.24	-1.01	0.446	0.723	1
GO_UBIQUITIN_LIGASE_COMPLEX	260	-0.2	-1.01	0.442	0.723	1
GO_POSITIVE_REGULATION_OF_HORMONE_SECRETION	115	-0.22	-1.01	0.434	0.723	1
GO_HISTONE_H4_ACETYLATION	46	-0.27	-1	0.445	0.725	1
GO_POLYPEPTIDE_N_ACETYLGALACTOSAMINYLTRANSFERASE_ACTIVIT	19	-0.33	-1	0.441	0.725	1
GO_ANTEROGRADE_AXONAL_TRANSPORT	26	-0.3	-1	0.477	0.726	1
GO_NEGATIVE_REGULATION_OF_CELL_GROWTH	170	-0.21	-1	0.449	0.725	1
GO_NEGATIVE_REGULATION_OF_CYTOPLASMIC_TRANSPORT	114	-0.22	-1	0.452	0.726	1
GO_LIGAND_DEPENDENT_NUCLEAR_RECEPTOR_BINDING	23	-0.31	-1	0.437	0.728	1
GO_RESPONSE_TO_AMINO_ACID	108	-0.22	-1	0.454	0.728	1
GO_CGMP_BIOSYNTHETIC_PROCESS	15	-0.35	-1	0.454	0.728	1
GO_REGULATION_OF_CELL_PROJECTION_ASSEMBLY	147	-0.21	-1	0.451	0.728	1
GO_ANION_TRANSMEMBRANE_TRANSPORT	251	-0.2	-1	0.457	0.728	1
GO_HMG_BOX_DOMAIN_BINDING	18	-0.34	-1	0.438	0.731	1
GO_ANCHORED_COMPONENT_OF_EXTERNAL_SIDE_OF_PLASMA_MEMBRANE	19	-0.32	-1	0.457	0.733	1
GO_NEGATIVE_REGULATION_OF_CALCIUM_ION_TRANSPORT_INTO_CYT	19	-0.33	-1	0.451	0.733	1
GO_ORGANIC_ACID_TRANSPORT	260	-0.2	-1	0.448	0.734	1
GO_NEGATIVE_REGULATION_OF_KINASE_ACTIVITY	248	-0.2	-1	0.48	0.734	1
GO_NEGATIVE_REGULATION_OF_TRANSMEMBRANE_RECEPTOR_PROTEIN_SERINE_THREONINE_KINASE_SIGNALING_PATHWAY	100	-0.23	-1	0.446	0.734	1
GO_REGULATION_OF_AXON_GUIDANCE	39	-0.27	-1	0.457	0.734	1
GO_LIPID_PHOSPHORYLATION	99	-0.23	-1	0.463	0.734	1
GO_POST_EMBRYONIC_DEVELOPMENT	88	-0.23	-1	0.45	0.736	1
GO_REGULATION_OF_SYSTEMIC_ARTERIAL_BLOOD_PRESSURE	84	-0.23	-1	0.461	0.735	1
GO_POSITIVE_REGULATION_OF_CELLULAR_COMPONENT_BIOGENESIS	396	-0.19	-1	0.464	0.735	1
GO_CARBOXYPEPTIDASE_ACTIVITY	41	-0.27	-0.99	0.47	0.74	1
GO_ATPASE_COUPLED_ION_TRANSMEMBRANE_TRANSPORTER_ACTIVI	71	-0.24	-0.99	0.464	0.74	1
GO_PROTEIN_SERINE_THREONINE_TYROSINE_KINASE_ACTIVITY	39	-0.27	-0.99	0.47	0.743	1
GO_NEGATIVE_REGULATION_OF_G_PROTEIN_COUPLED_RECEPTOR_PROTEIN_SIGNALING_PATHWAY	40	-0.27	-0.99	0.465	0.743	1
GO_STABILIZATION_OF_MEMBRANE_POTENTIAL	16	-0.34	-0.99	0.464	0.744	1
GO_CELLULAR_RESPONSE_TO_ESTROGEN_STIMULUS	40	-0.27	-0.99	0.466	0.744	1
GO_RECEPTOR_METABOLIC_PROCESS	81	-0.23	-0.99	0.493	0.743	1
GO_HEPARAN_SULFATE_PROTEOGLYCAN_BIOSYNTHETIC_PROCESS	23	-0.31	-0.99	0.479	0.744	1
GO_CELLULAR_RESPONSE_TO_TOXIC_SUBSTANCE	24	-0.31	-0.99	0.471	0.745	1
GO_POSITIVE_REGULATION_OF_CREB_TRANSCRIPTION_FACTOR_ACTI	15	-0.34	-0.99	0.472	0.744	1
GO_COPULATION	17	-0.33	-0.99	0.479	0.745	1
GO_CELLULAR_RESPONSE_TO_INSULIN_STIMULUS	144	-0.21	-0.99	0.482	0.745	1
GO_CENTRIOLAR_SATELLITE	24	-0.31	-0.99	0.474	0.746	1
GO_REGULATION_OF_INSULIN_SECRETION_INVOLVED_IN_CELLULAR_RESPONSE_TO_GLUCOSE_STIMULUS	51	-0.25	-0.99	0.459	0.747	1
GO_EAR_DEVELOPMENT	194	-0.21	-0.99	0.49	0.746	1
GO_PLATELET_DERIVED_GROWTH_FACTOR_RECEPTOR_SIGNALING_PATHWAY	34	-0.28	-0.99	0.47	0.75	1
GO_NEGATIVE_REGULATION_OF_OSSIFICATION	68	-0.24	-0.99	0.497	0.752	1
GO_REGULATION_OF_EPITHELIAL_CELL_MIGRATION	166	-0.21	-0.99	0.481	0.753	1
GO_DORSAL_VENTRAL_PATTERN_FORMATION	91	-0.23	-0.99	0.496	0.753	1
GO_NEGATIVE_REGULATION_OF_JNK_CASCADE	33	-0.28	-0.99	0.505	0.753	1
GO_CARDIAC_VENTRICLE_DEVELOPMENT	106	-0.22	-0.99	0.498	0.752	1
GO_UBIQUITIN_LIKE_PROTEIN_TRANSFERASE_ACTIVITY	409	-0.19	-0.99	0.507	0.753	1
GO_NEGATIVE_REGULATION_OF_BLOOD_CIRCULATION	36	-0.28	-0.99	0.464	0.752	1
GO_GUANOSINE_CONTAINING_COMPOUND_METABOLIC_PROCESS	44	-0.26	-0.99	0.481	0.752	1
GO_REGULATION_OF_DIGESTIVE_SYSTEM_PROCESS	35	-0.27	-0.99	0.471	0.752	1
GO_CLATHRIN_COAT	46	-0.26	-0.99	0.478	0.753	1
GO_ACTIN_BASED_CELL_PROJECTION	179	-0.21	-0.99	0.497	0.753	1
GO_REPRODUCTIVE_BEHAVIOR	30	-0.29	-0.98	0.467	0.753	1
GO_MOTOR_NEURON_AXON_GUIDANCE	27	-0.3	-0.98	0.487	0.753	1
GO_NEGATIVE_REGULATION_OF_PHOSPHORYLATION	418	-0.19	-0.98	0.51	0.753	1
GO_RESPONSE_TO_TRANSFORMING_GROWTH_FACTOR_BETA	144	-0.21	-0.98	0.507	0.756	1
GO_RESPONSE_TO_AMMONIUM_ION	51	-0.25	-0.98	0.476	0.756	1
GO_FIBRIL_ORGANIZATION	18	-0.33	-0.98	0.468	0.758	1
GO_LEFT_RIGHT_PATTERN_FORMATION	20	-0.32	-0.98	0.469	0.758	1
GO_COFACTOR_TRANSPORTER_ACTIVITY	21	-0.31	-0.98	0.468	0.759	1
GO_REGULATION_OF_CELLULAR_RESPONSE_TO_TRANSFORMING_GROWTH_FACTOR_BETA_STIMULUS	97	-0.22	-0.98	0.496	0.76	1
GO_POLYSACCHARIDE_BINDING	22	-0.31	-0.98	0.502	0.761	1
GO_REVERSE_CHOLESTEROL_TRANSPORT	17	-0.33	-0.98	0.499	0.762	1
GO_REGULATION_OF_CARBOHYDRATE_BIOSYNTHETIC_PROCESS	86	-0.23	-0.98	0.488	0.761	1
GO_PLASMA_MEMBRANE_RECEPTOR_COMPLEX	174	-0.21	-0.98	0.516	0.763	1
GO_REGULATION_OF_CELLULAR_COMPONENT_SIZE	331	-0.19	-0.98	0.545	0.764	1
GO_PLASMA_MEMBRANE_RAFT	86	-0.23	-0.98	0.513	0.764	1
GO_REGULATION_OF_CELL_MIGRATION_INVOLVED_IN_SPROUTING_ANGIOGENESIS	19	-0.31	-0.98	0.472	0.767	1
GO_METALLO_SULFUR_CLUSTER_ASSEMBLY	17	-0.33	-0.98	0.489	0.768	1
GO_REGULATION_OF_ACTIN_FILAMENT_BASED_PROCESS	308	-0.19	-0.98	0.52	0.77	1
GO_POSITIVE_REGULATION_OF_EPITHELIAL_CELL_MIGRATION	103	-0.22	-0.97	0.514	0.772	1

GO_PHOSPHATIDYLINOSITOL_3_KINASE_ACTIVITY	70	-0.24	-0.97	0.504	0.771	1
GO_CELLULAR_CARBOHYDRATE_CATABOLIC_PROCESS	33	-0.28	-0.97	0.496	0.771	1
GO_LYTIC_VACUOLE_MEMBRANE	265	-0.19	-0.97	0.532	0.772	1
GO_TRANS_GOLGI_NETWORK_MEMBRANE	78	-0.23	-0.97	0.488	0.775	1
GO_NAD_ADP_RIBOSYLTRANSFERASE_ACTIVITY	26	-0.29	-0.97	0.466	0.775	1
GO_ADIPOSE_TISSUE_DEVELOPMENT	32	-0.28	-0.97	0.5	0.776	1
GO_SERTOLI_CELL_DIFFERENTIATION	20	-0.31	-0.97	0.493	0.779	1
GO_CELLULAR_RESPONSE_TO_ESTRADIOL_STIMULUS	30	-0.28	-0.97	0.488	0.78	1
GO_KIDNEY_MORPHOGENESIS	82	-0.23	-0.97	0.539	0.781	1
GO_AMMONIUM_ION_METABOLIC_PROCESS	168	-0.2	-0.97	0.557	0.78	1
GO_CELL_SUBSTRATE_ADHERENS_JUNCTION_ASSEMBLY	24	-0.3	-0.97	0.51	0.781	1
GO_VACUOLE_ORGANIZATION	159	-0.2	-0.97	0.536	0.782	1
GO_REGULATION_OF_CARDIAC_MUSCLE_TISSUE_DEVELOPMENT	47	-0.26	-0.97	0.494	0.783	1
GO_ANION_TRANSMEMBRANE_TRANSPORTER_ACTIVITY	292	-0.19	-0.97	0.546	0.783	1
GO_REGULATION_OF_NUCLEAR_TRANSCRIBED_MRNA_CATABOLIC_PROCESS_DEADENYLATION_DEPENDENT_DECAY	15	-0.34	-0.97	0.477	0.782	1
GO_CALCIUM_DEPENDENT_PROTEIN_BINDING	61	-0.24	-0.97	0.528	0.783	1
GO_AMMONIUM_TRANSPORT	57	-0.24	-0.97	0.5	0.783	1
GO_UBIQUITIN_LIKE_PROTEIN_CONJUGATING_ENZYME_BINDING	33	-0.28	-0.97	0.51	0.783	1
GO_UROGENITAL_SYSTEM_DEVELOPMENT	297	-0.19	-0.97	0.559	0.783	1
GO_NEGATIVE_REGULATION_OF_GTPASE_ACTIVITY	41	-0.26	-0.97	0.495	0.784	1
GO_EYE_MORPHOGENESIS	136	-0.21	-0.97	0.547	0.786	1
GO_METHIONINE_METABOLIC_PROCESS	18	-0.32	-0.97	0.495	0.786	1
GO_POSITIVE_REGULATION_OF_SEQUESTERING_OF_CALCIUM_ION	16	-0.33	-0.96	0.516	0.786	1
GO_ALCOHOL_BINDING	99	-0.22	-0.96	0.537	0.788	1
GO_ATRIOVENTRICULAR_VALVE_DEVELOPMENT	19	-0.31	-0.96	0.491	0.791	1
GO_REGULATION_OF_NUCLEOTIDE_CATABOLIC_PROCESS	36	-0.26	-0.96	0.504	0.791	1
GO_RESPONSE_TO_CALCIUM_ION	115	-0.21	-0.96	0.548	0.791	1
GO_POSITIVE_REGULATION_OF_CELL_JUNCTION_ASSEMBLY	24	-0.3	-0.96	0.511	0.794	1
GO_BITTER_TASTE_RECEPTOR_ACTIVITY	24	-0.29	-0.96	0.516	0.794	1
GO_CYCLIN_BINDING	19	-0.32	-0.96	0.508	0.795	1
GO_REGULATION_OF_LIPID_KINASE_ACTIVITY	47	-0.26	-0.96	0.52	0.795	1
GO_CIS_GOLGI_NETWORK	40	-0.26	-0.96	0.518	0.796	1
GO_HISTONE_H3_DEACETYLATION	22	-0.29	-0.96	0.491	0.796	1
GO_POSITIVE_REGULATION_OF_VASODILATION	32	-0.27	-0.96	0.499	0.797	1
GO_REGULATION_OF_CALCIUM_ION_IMPORT	102	-0.22	-0.96	0.535	0.797	1
GO_REGULATION_OF_ADHERENS_JUNCTION_ORGANIZATION	49	-0.25	-0.96	0.549	0.8	1
GO_CARDIAC_CELL_DEVELOPMENT	49	-0.25	-0.96	0.534	0.8	1
GO_ADHERENS_JUNCTION_ORGANIZATION	71	-0.23	-0.96	0.531	0.802	1
GO_NEGATIVE_REGULATION_OF_ESTABLISHMENT_OF_PROTEIN_LOCALIZATION	205	-0.19	-0.96	0.557	0.803	1
GO_TRANSCRIPTION_FACTOR_ACTIVITY_RNA_POLYMERASE_II_TRANSCRIPTION_FACTOR_BINDING	132	-0.21	-0.96	0.567	0.804	1
GO_O_METHYLTRANSFERASE_ACTIVITY	18	-0.31	-0.96	0.53	0.804	1
GO_NUCLEOBASE_CONTAINING_COMPOUND_KINASE_ACTIVITY	45	-0.25	-0.95	0.531	0.807	1
GO_SWI_SNF_COMPLEX	15	-0.34	-0.95	0.503	0.807	1
GO_ACTOMYOSIN	58	-0.24	-0.95	0.551	0.809	1
GO_CATION_TRANSPORTING_ATPASE_ACTIVITY	59	-0.24	-0.95	0.548	0.809	1
GO_PROTEIN_K48_LINKED_DEUBIQUITINATION	20	-0.3	-0.95	0.53	0.811	1
GO_PEPTIDE_TRANSPORT	70	-0.23	-0.95	0.557	0.81	1
GO_PHOSPHATIDYLINOSITOL_3_4_5_TRISPHOSPHATE_BINDING	34	-0.27	-0.95	0.512	0.812	1
GO_REGULATION_OF_TOR_SIGNALING	63	-0.24	-0.95	0.538	0.815	1
GO_POSITIVE_REGULATION_OF_PEPTIDE_SECRETION	90	-0.22	-0.95	0.562	0.817	1
GO_CELLULAR_PIGMENTATION	46	-0.25	-0.95	0.545	0.818	1
GO_CARDIAC_VENTRICLE_MORPHOGENESIS	62	-0.24	-0.95	0.569	0.819	1
GO_ACTIVATION_OF_PROTEIN_KINASE_ACTIVITY	278	-0.19	-0.95	0.626	0.819	1
GO_PHOTOPERIODISM	24	-0.29	-0.95	0.526	0.818	1
GO_REGULATION_OF_EXTRACELLULAR_MATRIX_ORGANIZATION	29	-0.27	-0.95	0.545	0.818	1
GO_PHOSPHATIDYLETHANOLAMINE_METABOLIC_PROCESS	18	-0.31	-0.95	0.535	0.818	1
GO_REGULATION_OF_CHOLESTEROL_METABOLIC_PROCESS	22	-0.29	-0.95	0.516	0.818	1
GO_PROTEIN_C_TERMINUS_BINDING	184	-0.19	-0.95	0.594	0.819	1
GO_POSITIVE_REGULATION_OF_TOR_SIGNALING	26	-0.29	-0.95	0.511	0.82	1
GO_MATING	37	-0.26	-0.95	0.565	0.82	1
GO_NEGATIVE_REGULATION_OF_CELLULAR_PROTEIN_LOCALIZATION	134	-0.21	-0.95	0.572	0.82	1
GO_INTRINSIC_APOPTOTIC_SIGNALING_PATHWAY_BY_P53_CLASS_MEDIATOR	51	-0.24	-0.95	0.569	0.82	1
GO_PROTEIN_TYROSINE_KINASE_ACTIVITY	174	-0.2	-0.94	0.585	0.82	1
GO_NEGATIVE_REGULATION_OF_CELLULAR_RESPONSE_TO_INSULIN_STIMULUS	30	-0.27	-0.94	0.552	0.822	1
GO_ENDOTHELIAL_CELL_PROLIFERATION	24	-0.29	-0.94	0.557	0.823	1
GO_GERM_CELL_DEVELOPMENT	206	-0.19	-0.94	0.614	0.822	1
GO_BLOC_COMPLEX	20	-0.3	-0.94	0.541	0.823	1
GO_BODY_MORPHOGENESIS	44	-0.25	-0.94	0.54	0.823	1
GO_CALCIUM_ION_IMPORT	63	-0.23	-0.94	0.574	0.824	1
GO_NEGATIVE_REGULATION_OF_RECEPTOR_ACTIVITY	29	-0.27	-0.94	0.55	0.829	1
GO_SULFUR_AMINO_ACID_METABOLIC_PROCESS	40	-0.25	-0.94	0.545	0.828	1
GO_REGULATION_OF_POLYSACCHARIDE_METABOLIC_PROCESS	43	-0.25	-0.94	0.55	0.829	1
GO_GLYCEROPHOSPHOLIPID_METABOLIC_PROCESS	295	-0.18	-0.94	0.648	0.831	1
GO_PCG_PROTEIN_COMPLEX	43	-0.25	-0.94	0.563	0.831	1
GO_ACTIVIN_RECEPTOR_SIGNALING_PATHWAY	21	-0.3	-0.94	0.545	0.831	1
GO_NEGATIVE_REGULATION_OF_BLOOD_PRESSURE	43	-0.25	-0.94	0.543	0.834	1
GO_CYTOPLASMIC_SIDE_OF_MEMBRANE	167	-0.2	-0.94	0.619	0.834	1
GO_DEVELOPMENTAL_INDUCTION	27	-0.28	-0.94	0.553	0.835	1
GO_GTPASE_BINDING	291	-0.18	-0.94	0.645	0.837	1
GO_POSITIVE_REGULATION_OF_CARDIAC_MUSCLE_CELL_PROLIFERATI	19	-0.31	-0.94	0.557	0.836	1
GO_MONOCYTE_DIFFERENTIATION	16	-0.32	-0.94	0.575	0.836	1
GO_NEGATIVE_REGULATION_OF_GROWTH	236	-0.19	-0.93	0.642	0.838	1

GO_CAMERA_TYPE_EYE_MORPHOGENESIS	101	-0.21	-0.93	0.588	0.843	1
GO_REGULATION_OF_PHOSPHATIDYLINOSITOL_3_KINASE_ACTIVITY	39	-0.26	-0.93	0.59	0.846	1
GO_PHOSPHATASE_REGULATOR_ACTIVITY	80	-0.22	-0.93	0.599	0.847	1
GO_PROTEIN_LOCALIZATION_TO_MITOCHONDRION	66	-0.23	-0.93	0.578	0.848	1
GO_PROTEIN_AUTOUBIQUITINATION	47	-0.24	-0.93	0.592	0.848	1
GO_CALCIUM_DEPENDENT_PHOSPHOLIPID_BINDING	55	-0.24	-0.93	0.58	0.849	1
GO_NEGATIVE_REGULATION_OF_ORGANELLE_ASSEMBLY	21	-0.29	-0.93	0.563	0.854	1
GO_ATPASE_ACTIVATOR_ACTIVITY	17	-0.31	-0.93	0.556	0.855	1
GO_ACTIN_POLYMERIZATION_OR_DEPOLYMERIZATION	37	-0.26	-0.93	0.585	0.855	1
GO_TEMPERATURE_HOMEOSTASIS	26	-0.28	-0.93	0.572	0.855	1
GO_SAGA_TYPE_COMPLEX	34	-0.26	-0.93	0.586	0.857	1
GO_HYDRO_LYASE_ACTIVITY	49	-0.24	-0.93	0.59	0.857	1
GO_ECTODERMAL_PLACODE_DEVELOPMENT	15	-0.33	-0.92	0.535	0.857	1
GO_POSITIVE_REGULATION_OF_BIOMINERAL_TISSUE_DEVELOPMENT	38	-0.25	-0.92	0.571	0.859	1
GO_PHOSPHATIDYLINOSITOL_PHOSPHATE_KINASE_ACTIVITY	16	-0.31	-0.92	0.568	0.858	1
GO_REGULATION_OF_SPROUTING_ANGIOGENESIS	28	-0.28	-0.92	0.592	0.858	1
GO_SEX_DETERMINATION	22	-0.29	-0.92	0.572	0.858	1
GO_DORSAL_SPINAL_CORD_DEVELOPMENT	21	-0.3	-0.92	0.568	0.858	1
GO_REGULATION_OF_PHOSPHATASE_ACTIVITY	117	-0.21	-0.92	0.65	0.858	1
GO_NEGATIVE_REGULATION_OF_ENDOTHELIAL_CELL_PROLIFERATION	32	-0.26	-0.92	0.606	0.858	1
GO_REGULATION_OF_HOMEOSTATIC_PROCESS	438	-0.17	-0.92	0.724	0.859	1
GO_SCHWANN_CELL_DIFFERENTIATION	31	-0.27	-0.92	0.585	0.866	1
GO_POSITIVE_REGULATION_OF_CELLULAR_RESPONSE_TO_TRANSFORMING_GROWTH_FACTOR_BETA_STIMULUS	24	-0.28	-0.92	0.585	0.867	1
GO_FERTILIZATION	144	-0.2	-0.92	0.629	0.867	1
GO_POSITIVE_REGULATION_OF_CELL_MATRIX_ADHESION	40	-0.25	-0.92	0.583	0.868	1
GO_NUCLEAR_OUTER_MEMBRANE	23	-0.29	-0.92	0.581	0.868	1
GO_HYDROLASE_ACTIVITY_ACTING_ON_CARBON_NITROGEN_BUT_NOT_PEPTIDE_BONDS_IN_LINEAR_AMIDES	84	-0.21	-0.92	0.639	0.867	1
GO_GLUTATHIONE_TRANSFERASE_ACTIVITY	33	-0.26	-0.92	0.603	0.868	1
GO_MYOSIN_BINDING	59	-0.23	-0.92	0.608	0.869	1
GO_REGULATION_OF_CELL_JUNCTION_ASSEMBLY	67	-0.22	-0.92	0.625	0.868	1
GO_ACTIVATION_OF_PHOSPHOLIPASE_C_ACTIVITY	27	-0.28	-0.92	0.581	0.87	1
GO_RESPONSE_TO_SALT	16	-0.31	-0.92	0.602	0.87	1
GO_ADAPTATION_OF_SIGNALING_PATHWAY	21	-0.3	-0.92	0.566	0.87	1
GO_POSITIVE_REGULATION_OF_RECEPTOR_ACTIVITY	45	-0.24	-0.91	0.601	0.873	1
GO_ATPASE_COMPLEX	24	-0.28	-0.91	0.594	0.874	1
GO_AUTOPHAGOSOME_ORGANIZATION	40	-0.25	-0.91	0.615	0.875	1
GO_HYDROGEN_PEROXIDE_CATABOLIC_PROCESS	20	-0.29	-0.91	0.565	0.875	1
GO_CARDIOCYTE_DIFFERENTIATION	96	-0.21	-0.91	0.644	0.875	1
GO_HEPARAN_SULFATE_PROTEOGLYCAN_METABOLIC_PROCESS	28	-0.27	-0.91	0.582	0.875	1
GO_ACTIN_MYOSIN_FILAMENT_SLIDING	38	-0.25	-0.91	0.617	0.875	1
GO_NEGATIVE_REGULATION_OF_FAT_CELL_DIFFERENTIATION	41	-0.24	-0.91	0.612	0.875	1
GO_GLYCOSPHINGOLIPID_METABOLIC_PROCESS	69	-0.22	-0.91	0.637	0.875	1
GO_RHYTHMIC_BEHAVIOR	19	-0.3	-0.91	0.589	0.876	1
GO_PEPTIDYL_TYROSINE_MODIFICATION	184	-0.19	-0.91	0.701	0.877	1
GO_PEPTIDE_HORMONE_PROCESSING	32	-0.26	-0.91	0.631	0.879	1
GO_POSITIVE_REGULATION_OF_ENDOTHELIAL_CELL_PROLIFERATION	68	-0.22	-0.91	0.632	0.88	1
GO_MESONEPHRIC_TUBULE_MORPHOGENESIS	53	-0.23	-0.91	0.614	0.88	1
GO_FATTY_ACID_TRANSPORT	56	-0.23	-0.91	0.632	0.88	1
GO_CELLULAR_RESPONSE_TO_GROWTH_HORMONE_STIMULUS	20	-0.29	-0.91	0.587	0.883	1
GO_REGULATION_OF_ENDOTHELIAL_CELL_DIFFERENTIATION	27	-0.27	-0.91	0.623	0.883	1
GO_CELLULAR_IRON_ION_HOMEOSTASIS	44	-0.24	-0.91	0.627	0.882	1
GO_FATTY_ACID_LIGASE_ACTIVITY	16	-0.3	-0.91	0.599	0.883	1
GO_SKELETAL_MUSCLE_ORGAN_DEVELOPMENT	137	-0.2	-0.91	0.701	0.883	1
GO_POSITIVE_REGULATION_OF_MUSCLE_TISSUE_DEVELOPMENT	55	-0.23	-0.91	0.639	0.883	1
GO_POSITIVE_REGULATION_OF_MYOBLAST_DIFFERENTIATION	22	-0.29	-0.91	0.582	0.883	1
GO_REACTIVE_NITROGEN_SPECIES_METABOLIC_PROCESS	19	-0.3	-0.91	0.602	0.883	1
GO_PHOSPHOLIPID_METABOLIC_PROCESS	362	-0.17	-0.91	0.759	0.883	1
GO_CLATHRIN_ADAPTOR_COMPLEX	27	-0.27	-0.91	0.609	0.883	1
GO_POSITIVE_REGULATION_OF_KINASE_ACTIVITY	479	-0.17	-0.9	0.814	0.884	1
GO_PROTEIN_DEPHOSPHORYLATION	188	-0.19	-0.9	0.706	0.885	1
GO_REGULATION_OF_VASCULATURE_DEVELOPMENT	233	-0.18	-0.9	0.718	0.884	1
GO_MACROMOLECULE_DEACYLATION	66	-0.22	-0.9	0.629	0.884	1
GO_TRANSCRIPTIONAL_REPRESSOR_COMPLEX	74	-0.22	-0.9	0.654	0.884	1
GO_REGULATION_OF_BLOOD_VESSEL_ENDOTHELIAL_CELL_MIGRATION	51	-0.23	-0.9	0.608	0.884	1
GO_NEGATIVE_REGULATION_OF_BMP_SIGNALING_PATHWAY	41	-0.24	-0.9	0.635	0.883	1
GO_POSITIVE_REGULATION_OF_EXTRACELLULAR_MATRIX_ORGANIZATI	17	-0.3	-0.9	0.595	0.883	1
GO_POSITIVE_REGULATION_OF_PHOSPHATASE_ACTIVITY	28	-0.27	-0.9	0.595	0.883	1
GO_NUCLEOSIDE_DIPHOSPHATE_KINASE_ACTIVITY	15	-0.32	-0.9	0.581	0.883	1
GO_REGULATION_OF_MUSCLE_CELL_APOPTOTIC_PROCESS	42	-0.24	-0.9	0.633	0.882	1
GO_POSITIVE_REGULATION_OF_CELL_GROWTH	144	-0.19	-0.9	0.703	0.883	1
GO_NEGATIVE_REGULATION_OF_POTASSIUM_ION_TRANSMEMBRANE_TRANSPORTER_ACTIVITY	16	-0.31	-0.9	0.587	0.883	1
GO_MEMBRANE_LIPID_BIOSYNTHETIC_PROCESS	106	-0.2	-0.9	0.691	0.882	1
GO_EPHRIN_RECEPTOR_ACTIVITY	19	-0.3	-0.9	0.618	0.885	1
GO_TRANSCRIPTION_COREPRESSOR_ACTIVITY	212	-0.19	-0.9	0.749	0.884	1
GO_DEVELOPMENTAL_GROWTH	328	-0.17	-0.9	0.77	0.884	1
GO_ACTIVE_TRANSMEMBRANE_TRANSPORTER_ACTIVITY	349	-0.17	-0.9	0.791	0.885	1
GO_CELLULAR_PROCESS_INVOLVED_IN_REPRODUCTION_IN_MULTICELLULAR_ORGANISM	249	-0.18	-0.9	0.729	0.885	1
GO_PROTEIN_SERINE_THREONINE_KINASE_INHIBITOR_ACTIVITY	30	-0.26	-0.9	0.634	0.885	1
GO_ASTROCYTE_DIFFERENTIATION	39	-0.25	-0.9	0.634	0.885	1
GO_REGULATION_OF_PHOSPHOPROTEIN_PHOSPHATASE_ACTIVITY	58	-0.22	-0.9	0.662	0.889	1
GO_PROTEIN_KINASE_COMPLEX	88	-0.2	-0.9	0.673	0.888	1
GO_POSITIVE_REGULATION_OF_OSSIFICATION	84	-0.21	-0.9	0.661	0.889	1
GO_ORGANIC_ANION_TRANSPORT	385	-0.17	-0.9	0.793	0.89	1

GO_COFACTOR_TRANSPORT	26	-0.27	-0.9	0.591	0.891	1
GO_SERINE_TYPE_EXOPEPTIDASE_ACTIVITY	18	-0.3	-0.9	0.592	0.891	1
GO_LAMELLIPODIUM	170	-0.19	-0.9	0.704	0.891	1
GO_ASYMMETRIC_PROTEIN_LOCALIZATION	19	-0.29	-0.89	0.592	0.893	1
GO_REGULATION_OF_CELLULAR_RESPIRATION	21	-0.28	-0.89	0.611	0.897	1
GO_ACETYLCHOLINE_BINDING	24	-0.27	-0.89	0.617	0.897	1
GO_NUCLEOTIDE_EXCISION_REPAIR_DNA_DAMAGE_RECOGNITION	23	-0.28	-0.89	0.613	0.896	1
GO_RECYCLING_ENDOSOME	126	-0.2	-0.89	0.736	0.896	1
GO_NEGATIVE_REGULATION_OF_CIRCADIAN_RHYTHM	17	-0.29	-0.89	0.61	0.898	1
GO_REGULATION_OF_FAT_CELL_DIFFERENTIATION	105	-0.2	-0.89	0.678	0.901	1
GO_VENTRICULAR_SEPTUM_DEVELOPMENT	54	-0.23	-0.89	0.668	0.901	1
GO_POSITIVE_REGULATION_OF_PROTEIN_BINDING	73	-0.22	-0.89	0.686	0.901	1
GO_AMMONIUM_ION_BINDING	70	-0.22	-0.89	0.668	0.903	1
GO_REGULATION_OF_HEAT_GENERATION	15	-0.31	-0.89	0.629	0.903	1
GO_MANNOSIDASE_ACTIVITY	15	-0.3	-0.89	0.61	0.906	1
GO_REGULATION_OF_PROTEIN_POLYMERIZATION	169	-0.19	-0.89	0.713	0.906	1
GO_NEGATIVE_REGULATION_OF_CYCLIN_DEPENDENT_PROTEIN_KINASE_ACTIVITY	32	-0.26	-0.89	0.64	0.908	1
GO_CELLULAR_GLUCOSE_HOMEOSTASIS	75	-0.21	-0.89	0.682	0.908	1
GO_AXON_CYTOPLASM	33	-0.25	-0.89	0.643	0.908	1
GO_NEPHRIC_DUCT_DEVELOPMENT	15	-0.31	-0.89	0.611	0.909	1
GO_TRANSMEMBRANE_RECEPTOR_PROTEIN_TYROSINE_KINASE_SIGNALING_PATHWAY	495	-0.17	-0.88	0.855	0.909	1
GO_PROTEIN_PALMITOYLATION	23	-0.27	-0.88	0.633	0.909	1
GO_PHOTORECEPTOR_DISC_MEMBRANE	18	-0.29	-0.88	0.643	0.91	1
GO_CLATHRIN_COATED_VESICLE	155	-0.19	-0.88	0.753	0.912	1
GO_GLYCEROLIPID_METABOLIC_PROCESS	352	-0.17	-0.88	0.837	0.911	1
GO_REGULATION_OF_INCLUSION_BODY_ASSEMBLY	16	-0.3	-0.88	0.612	0.911	1
GO_MRNA_3_UTR_BINDING	48	-0.23	-0.88	0.645	0.912	1
GO_NEGATIVE_REGULATION_OF_TRANSCRIPTION_FACTOR_IMPORT_INTO_NUCLEUS	38	-0.25	-0.88	0.662	0.912	1
GO_RHO_GUANYL_NUCLEOTIDE_EXCHANGE_FACTOR_ACTIVITY	74	-0.21	-0.88	0.685	0.912	1
GO_RESPONSE_TO_CAMP	104	-0.2	-0.88	0.712	0.912	1
GO_MECHANORECEPTOR_DIFFERENTIATION	50	-0.23	-0.88	0.676	0.912	1
GO_LATE_ENDOSOME	202	-0.18	-0.88	0.772	0.913	1
GO_GLYCOPROTEIN_METABOLIC_PROCESS	344	-0.17	-0.88	0.837	0.913	1
GO_REGULATION_OF_VACUOLAR_TRANSPORT	29	-0.26	-0.88	0.637	0.913	1
GO_APOLIPOPROTEIN_BINDING	15	-0.31	-0.88	0.642	0.913	1
GO_ENTEROENDOCRINE_CELL_DIFFERENTIATION	19	-0.29	-0.88	0.647	0.913	1
GO_PROTON_TRANSPORTING_TWO_SECTOR_ATPASE_COMPLEX	47	-0.23	-0.88	0.669	0.915	1
GO_NEGATIVE_REGULATION_OF_OXIDOREDUCTASE_ACTIVITY	26	-0.26	-0.88	0.644	0.916	1
GO_POSITIVE_REGULATION_OF_MICROTUBULE_POLYMERIZATION_OR_DEPOLYMERIZATION	22	-0.28	-0.88	0.636	0.918	1
GO_CYTOPLASMIC_SEQUESTERING_OF_TRANSCRIPTION_FACTOR	18	-0.29	-0.88	0.631	0.919	1
GO_POSITIVE_REGULATION_OF_RECEPTOR_INTERNALIZATION	24	-0.27	-0.88	0.642	0.92	1
GO_REGULATION_OF_NEURON_PROJECTION_REGENERATION	19	-0.29	-0.87	0.683	0.92	1
GO_NEGATIVE_REGULATION_OF_DEVELOPMENTAL_GROWTH	84	-0.21	-0.87	0.729	0.92	1
GO_MUSCLE_CELL_FATE_COMMITMENT	15	-0.31	-0.87	0.635	0.92	1
GO_HISTONE_METHYLTRANSFERASE_ACTIVITY_H3_K4_SPECIFIC_	18	-0.28	-0.87	0.661	0.92	1
GO_MUSCLE_TISSUE_DEVELOPMENT	275	-0.17	-0.87	0.846	0.92	1
GO_POSITIVE_REGULATION_OF_ACTIN_FILAMENT_BUNDLE_ASSEMBLY	48	-0.23	-0.87	0.646	0.92	1
GO_RECEPTOR_SIGNALING_PROTEIN_SERINE_THREONINE_KINASE_ACTIVITY	92	-0.2	-0.87	0.723	0.92	1
GO_NEGATIVE_REGULATION_OF_TRANSMEMBRANE_TRANSPORT	86	-0.2	-0.87	0.741	0.922	1
GO_REGULATION_OF_CELLULAR_RESPONSE_TO_INSULIN_STIMULUS	58	-0.22	-0.87	0.693	0.921	1
GO_POLYOL_CATABOLIC_PROCESS	18	-0.3	-0.87	0.645	0.921	1
GO_REGULATION_OF_NFAT_PROTEIN_IMPORT_INTO_NUCLEUS	17	-0.3	-0.87	0.659	0.92	1
GO_RHYTHMIC_PROCESS	298	-0.17	-0.87	0.838	0.921	1
GO_REGULATION_OF_GLUCOSE_METABOLIC_PROCESS	104	-0.2	-0.87	0.733	0.924	1
GO_GLYCEROLIPID_BIOSYNTHETIC_PROCESS	208	-0.18	-0.87	0.784	0.925	1
GO_ACTIN_BINDING	388	-0.17	-0.87	0.875	0.925	1
GO_SUGAR_TRANSMEMBRANE_TRANSPORTER_ACTIVITY	31	-0.25	-0.87	0.655	0.924	1
GO_POSITIVE_REGULATION_OF_LIPID_KINASE_ACTIVITY	31	-0.25	-0.87	0.658	0.924	1
GO_VOLTAGE_GATED_ANION_CHANNEL_ACTIVITY	17	-0.3	-0.87	0.668	0.923	1
GO_MICROTUBULE_NUCLEATION	18	-0.28	-0.87	0.667	0.923	1
GO_REGULATION_OF_ENDOTHELIAL_CELL_PROLIFERATION	98	-0.2	-0.87	0.73	0.923	1
GO_NADH_DEHYDROGENASE_COMPLEX	42	-0.23	-0.87	0.671	0.923	1
GO_L_ALPHA_AMINO_ACID_TRANSMEMBRANE_TRANSPORT	31	-0.25	-0.87	0.67	0.922	1
GO_REGULATION_OF_GLIOGENESIS	88	-0.2	-0.87	0.724	0.922	1
GO_REGULATION_OF_MYELINATION	33	-0.25	-0.87	0.658	0.922	1
GO_CARDIAC_MUSCLE_TISSUE_DEVELOPMENT	140	-0.19	-0.87	0.774	0.921	1
GO_STEROL_TRANSPORTER_ACTIVITY	18	-0.28	-0.87	0.652	0.922	1
GO_REGULATION_OF_CARDIAC_MUSCLE_CELL_DIFFERENTIATION	18	-0.28	-0.87	0.66	0.922	1
GO_NEGATIVE_REGULATION_OF_ENDOCYTOSIS	39	-0.24	-0.87	0.718	0.923	1
GO_PLATELET_DEGRANULATION	105	-0.2	-0.87	0.749	0.922	1
GO_PH_REDUCTION	39	-0.24	-0.87	0.696	0.923	1
GO_ORGANIC_ACID_CATABOLIC_PROCESS	202	-0.18	-0.87	0.816	0.925	1
GO_PATTERN_SPECIFICATION_PROCESS	415	-0.17	-0.87	0.858	0.925	1
GO_RESPONSE_TO_GROWTH_FACTOR	473	-0.16	-0.86	0.907	0.925	1
GO_REGULATION_OF_ATPASE_ACTIVITY	58	-0.22	-0.86	0.73	0.926	1
GO_CIRCADIAN_RHYTHM	137	-0.19	-0.86	0.791	0.925	1
GO_PHOSPHOLIPID_CATABOLIC_PROCESS	29	-0.25	-0.86	0.678	0.927	1
GO_DICARBOXYLIC_ACID_CATABOLIC_PROCESS	16	-0.3	-0.86	0.659	0.928	1
GO_CONNECTIVE_TISSUE_DEVELOPMENT	194	-0.18	-0.86	0.805	0.927	1
GO_NEUROTRANSMITTER_TRANSPORTER_ACTIVITY	25	-0.26	-0.86	0.673	0.926	1
GO_BILE_ACID_BIOSYNTHETIC_PROCESS	20	-0.28	-0.86	0.696	0.929	1
GO_MEGAKARYOCYTE_DEVELOPMENT	16	-0.3	-0.86	0.664	0.929	1

GO_EMBRYONIC_PATTERN_SPECIFICATION	58	-0.21	-0.86	0.735	0.929	1
GO_RESPONSE_TO_ALKALOID	137	-0.18	-0.86	0.795	0.93	1
GO_MACROMOLECULAR_COMPLEX_REMODELING	24	-0.27	-0.86	0.664	0.93	1
GO_ENDOPLASMIC_RETICULUM_CALCIUM_ION_HOMEOSTASIS	20	-0.27	-0.86	0.673	0.931	1
GO_MESENCHYME_MORPHOGENESIS	38	-0.24	-0.86	0.68	0.93	1
GO_REGULATION_OF_PROTEIN_LOCALIZATION_TO_CELL_SURFACE	27	-0.26	-0.86	0.668	0.931	1
GO_NOTCH_BINDING	18	-0.28	-0.86	0.677	0.931	1
GO_POSITIVE_REGULATION_OF_VASOCONSTRICTION	35	-0.24	-0.86	0.711	0.933	1
GO_MULTIVESICULAR_BODY	37	-0.23	-0.86	0.697	0.932	1
GO_CHEMOREPELLENT_ACTIVITY	27	-0.25	-0.86	0.685	0.932	1
GO_CHLORIDE_CHANNEL_REGULATOR_ACTIVITY	15	-0.3	-0.86	0.677	0.932	1
GO_STEROL_BINDING	43	-0.23	-0.86	0.721	0.932	1
GO_MODULATION_BY_HOST_OF_VIRAL_PROCESS	18	-0.28	-0.86	0.656	0.932	1
GO_REGULATION_OF_ORGAN_GROWTH	72	-0.21	-0.86	0.719	0.932	1
GO_MUSCLE_CELL_PROLIFERATION	19	-0.28	-0.86	0.681	0.932	1
GO_CELLULAR_RESPONSE_TO_PROSTAGLANDIN_E_STIMULUS	18	-0.28	-0.86	0.676	0.931	1
GO_METANEPHROS_DEVELOPMENT	81	-0.2	-0.86	0.768	0.932	1
GO_NEGATIVE_REGULATION_OF_TELOMERE_MAINTENANCE	26	-0.26	-0.86	0.685	0.931	1
GO_NEGATIVE_REGULATION_OF_INTRACELLULAR_STEROID_HORMONE_RECEPTOR_SIGNALING_PATHWAY	30	-0.24	-0.86	0.695	0.931	1
GO_NEGATIVE_REGULATION_OF_HOMEOSTATIC_PROCESS	124	-0.19	-0.85	0.803	0.932	1
GO_RESPONSE_TO_MERCURY_ION	15	-0.3	-0.85	0.673	0.932	1
GO_POSITIVE_REGULATION_OF_GLUCOSE_METABOLIC_PROCESS	36	-0.24	-0.85	0.729	0.932	1
GO_REGULATION_OF_OXIDATIVE_STRESS_INDUCED_INTRINSIC_APOPTOTIC_SIGNALING_PATHWAY	29	-0.25	-0.85	0.695	0.934	1
GO_TELOMERIC_DNA_BINDING	28	-0.25	-0.85	0.704	0.934	1
GO_STRUCTURAL_CONSTITUENT_OF_EYE_LENS	20	-0.28	-0.85	0.682	0.935	1
GO_CELLULAR_RESPONSE_TO_VASCULAR_ENDOTHELIAL_GROWTH_FACTOR_STIMULUS	30	-0.25	-0.85	0.685	0.936	1
GO_ANCHORED_COMPONENT_OF_PLASMA_MEMBRANE	40	-0.23	-0.85	0.722	0.937	1
GO_POSITIVE_REGULATION_OF_CARBOHYDRATE_METABOLIC_PROCES	75	-0.2	-0.85	0.761	0.937	1
GO_PHOSPHOLIPASE_ACTIVITY	93	-0.2	-0.85	0.782	0.938	1
GO_HEPARAN_SULFATE_SULFOTRANSFERASE_ACTIVITY	15	-0.29	-0.85	0.652	0.937	1
GO_REGULATION_OF_EPITHELIAL_TO_MESENCHYMAL_TRANSITION	67	-0.21	-0.85	0.735	0.938	1
GO_EARLY_ENDOSOME	292	-0.17	-0.85	0.911	0.939	1
GO_EMBRYONIC_AXIS_SPECIFICATION	33	-0.24	-0.85	0.734	0.938	1
GO_POSITIVE_REGULATION_OF_RECEPTOR_MEDIATED_ENDOCYTOSIS	47	-0.22	-0.85	0.748	0.94	1
GO_MANNOSYLATION	34	-0.23	-0.85	0.714	0.94	1
GO_CARDIAC_EPITHELIAL_TO_MESENCHYMAL_TRANSITION	24	-0.26	-0.85	0.694	0.941	1
GO_POSITIVE_REGULATION_OF_PROTEIN_DEPOLYMERIZATION	19	-0.28	-0.85	0.653	0.943	1
GO_COATED_MEMBRANE	87	-0.2	-0.84	0.778	0.942	1
GO_ORGAN_FORMATION	34	-0.24	-0.84	0.709	0.944	1
GO_POLYAMINE_METABOLIC_PROCESS	15	-0.3	-0.84	0.678	0.943	1
GO_AU_RICH_ELEMENT_BINDING	23	-0.26	-0.84	0.7	0.943	1
GO_PROTEIN_INSERTION_INTO_MEMBRANE	22	-0.26	-0.84	0.682	0.943	1
GO_REGULATION_OF_PLATELET_AGGREGATION	17	-0.28	-0.84	0.671	0.942	1
GO_SKELETAL_MUSCLE_CELL_DIFFERENTIATION	53	-0.22	-0.84	0.755	0.942	1
GO_STEREOCILIUM_BUNDLE	41	-0.23	-0.84	0.745	0.941	1
GO_ENDOSOMAL_PART	415	-0.16	-0.84	0.931	0.942	1
GO_PHOTORECEPTOR_INNER_SEGMENT	36	-0.24	-0.84	0.715	0.942	1
GO_SPHINGOLIPID_METABOLIC_PROCESS	130	-0.18	-0.84	0.81	0.942	1
GO_MUSCLE_CELL_CELLULAR_HOMEOSTASIS	19	-0.27	-0.84	0.662	0.943	1
GO_ALPHA_AMINO_ACID_CATABOLIC_PROCESS	94	-0.19	-0.84	0.806	0.943	1
GO_PROTEIN_KINASE_B_SIGNALING	34	-0.23	-0.84	0.72	0.943	1
GO_SIGNALING_ADAPTOR_ACTIVITY	74	-0.2	-0.84	0.761	0.942	1
GO_PROTEIN_DEGLYCOSYLATION	21	-0.26	-0.84	0.694	0.942	1
GO_SECRETORY_GRANULE_LUMEN	81	-0.2	-0.84	0.775	0.941	1
GO_NEGATIVE_REGULATION_OF_CARDIAC_MUSCLE_TISSUE_DEVELOP	17	-0.28	-0.84	0.681	0.942	1
GO_NEUROTROPHIN_TRK_RECEPTOR_SIGNALING_PATHWAY	15	-0.29	-0.84	0.689	0.942	1
GO_MONOCARBOXYLIC_ACID_CATABOLIC_PROCESS	93	-0.19	-0.84	0.819	0.944	1
GO_MESENCHYME_DEVELOPMENT	188	-0.17	-0.84	0.876	0.944	1
GO_NEGATIVE_REGULATION_OF_EPITHELIAL_TO_MESENCHYMAL_TRANSITION	23	-0.26	-0.84	0.715	0.948	1
GO_NEGATIVE_REGULATION_OF_CELLULAR_RESPONSE_TO_GROWTH_FACTOR_STIMULUS	119	-0.18	-0.84	0.837	0.948	1
GO_NEGATIVE_REGULATION_OF_EMBRYONIC_DEVELOPMENT	26	-0.25	-0.84	0.737	0.948	1
GO_VESICLE_COATING	73	-0.2	-0.84	0.795	0.948	1
GO_IRON_ION_HOMEOSTASIS	67	-0.2	-0.84	0.787	0.948	1
GO_HORMONE_ACTIVITY	115	-0.19	-0.83	0.839	0.949	1
GO_FAT_CELL_DIFFERENTIATION	104	-0.19	-0.83	0.807	0.949	1
GO_GLYCEROLIPID_CATABOLIC_PROCESS	36	-0.23	-0.83	0.759	0.948	1
GO_NEGATIVE_REGULATION_OF_SMOOTH_MUSCLE_CELL_PROLIFERATI	36	-0.23	-0.83	0.729	0.948	1
GO_KINASE_ACTIVATOR_ACTIVITY	61	-0.21	-0.83	0.771	0.949	1
GO_ANION_ANION_ANTIPORTER_ACTIVITY	22	-0.26	-0.83	0.726	0.95	1
GO_TYPE_I_INTERFERON_RECEPTOR_BINDING	17	-0.28	-0.83	0.699	0.951	1
GO_NERVE_DEVELOPMENT	68	-0.2	-0.83	0.781	0.951	1
GO_POSITIVE_REGULATION_OF_MUSCLE_CONTRACTION	44	-0.22	-0.83	0.758	0.951	1
GO_REGULATION_OF_ACTION_POTENTIAL	38	-0.23	-0.83	0.774	0.953	1
GO_REGULATION_OF_GRANULOCYTE_DIFFERENTIATION	16	-0.29	-0.83	0.718	0.953	1
GO_PLATELET_DENSE_GRANULE	20	-0.27	-0.83	0.694	0.952	1
GO_REGULATION_OF_NUCLEOSIDE_METABOLIC_PROCESS	49	-0.22	-0.83	0.764	0.954	1
GO_DRUG_TRANSMEMBRANE_TRANSPORT	19	-0.27	-0.83	0.7	0.954	1
GO_REGULATION_OF_ENDOCYTOSIS	198	-0.17	-0.83	0.898	0.954	1
GO_REGULATION_OF_HYDROGEN_PEROXIDE_METABOLIC_PROCESS	15	-0.29	-0.83	0.692	0.954	1
GO_GLYCOLIPID_BIOSYNTHETIC_PROCESS	62	-0.2	-0.83	0.776	0.955	1
GO_EPITHELIAL_CELL_FATE_COMMITMENT	15	-0.29	-0.83	0.711	0.955	1
GO_STEROID_HORMONE_MEDIATED_SIGNALING_PATHWAY	122	-0.18	-0.83	0.838	0.955	1

GO_ARTERY_DEVELOPMENT	75	-0.2	-0.83	0.799	0.956	1
GO_LIPID_TRANSLOCATION	21	-0.26	-0.82	0.713	0.959	1
GO_GLUCAN_BIOSYNTHETIC_PROCESS	25	-0.25	-0.82	0.724	0.961	1
GO_SECRETORY_GRANULE	345	-0.16	-0.82	0.96	0.961	1
GO_N_ACETYLGLUCOSAMINE_METABOLIC_PROCESS	16	-0.28	-0.82	0.726	0.964	1
GO_REGULATION_OF_COFACTOR_METABOLIC_PROCESS	50	-0.21	-0.82	0.779	0.964	1
GO_RETINOIC_ACID_RECEPTOR_SIGNALING_PATHWAY	18	-0.27	-0.82	0.716	0.963	1
GO_POSITIVE_REGULATION_OF_CYCLIN_DEPENDENT_PROTEIN_KINASE_ACTIVITY	35	-0.23	-0.82	0.755	0.969	1
GO_RESPONSE_TO_CORTICOSTERONE	26	-0.24	-0.82	0.767	0.97	1
GO_RENAL_SYSTEM_VASCULATURE_DEVELOPMENT	19	-0.27	-0.82	0.707	0.969	1
GO_NEGATIVE_REGULATION_OF_MACROAUTOPHAGY	22	-0.25	-0.81	0.727	0.97	1
GO_DEFINITIVE_HEMOPOIESIS	17	-0.27	-0.81	0.721	0.969	1
GO_PROTEIN_LIPID_COMPLEX_BINDING	24	-0.25	-0.81	0.722	0.97	1
GO_PROTEIN_LIPID_COMPLEX_SUBUNIT_ORGANIZATION	36	-0.22	-0.81	0.777	0.973	1
GO_REGULATION_OF_RESPONSE_TO_REACTIVE_OXYGEN_SPECIES	33	-0.23	-0.81	0.755	0.973	1
GO_CALCIUM_ION_IMPORT_INTO_CYTOSOL	42	-0.22	-0.81	0.79	0.974	1
GO_POSITIVE_REGULATION_OF_VASCULAR_ENDOTHELIAL_GROWTH_FACTOR_RECEPTOR_SIGNALING_PATHWAY	16	-0.28	-0.81	0.703	0.975	1
GO_POSITIVE_REGULATION_OF_SKELETAL_MUSCLE_TISSUE_DEVELOPMENT	26	-0.24	-0.81	0.759	0.975	1
GO_CELL_VOLUME_HOMEOSTASIS	28	-0.24	-0.81	0.749	0.976	1
GO_NEGATIVE_REGULATION_OF_RECEPTOR_MEDIATED_ENDOCYTOSIS	17	-0.27	-0.81	0.739	0.976	1
GO_MONOCARBOXYLIC_ACID_TRANSPORT	123	-0.18	-0.81	0.887	0.976	1
GO_HOMEOSTASIS_OF_NUMBER_OF_CELLS_WITHIN_A_TISSUE	31	-0.23	-0.81	0.769	0.976	1
GO_ACETYLGLUCOSAMINYLTRANSFERASE_ACTIVITY	48	-0.21	-0.81	0.811	0.976	1
GO_AMINO_ACID_BINDING	106	-0.18	-0.81	0.875	0.977	1
GO_POSITIVE_REGULATION_OF_STRIATED_MUSCLE_CONTRACTION	15	-0.28	-0.81	0.718	0.977	1
GO_REGULATION_OF_PEPTIDYL_SERINE_PHOSPHORYLATION_OF_STAT_PROTEIN	21	-0.26	-0.81	0.732	0.976	1
GO_NOTCH_SIGNALING_PATHWAY	114	-0.18	-0.81	0.868	0.976	1
GO_NATURAL_KILLER_CELL_ACTIVATION_INVOLVED_IN_IMMUNE_RESP	24	-0.25	-0.81	0.78	0.976	1
GO_REGULATION_OF_GENERATION_OF_PRECURSOR_METABOLITES_AND_ENERGY	87	-0.19	-0.8	0.847	0.978	1
GO_MULTICELLULAR_ORGANISMAL_MOVEMENT	41	-0.22	-0.8	0.826	0.978	1
GO_KINASE_REGULATOR_ACTIVITY	184	-0.17	-0.8	0.934	0.978	1
GO_TRANSFORMING_GROWTH_FACTOR_BETA_RECEPTOR_SIGNALING_PATHWAY	95	-0.19	-0.8	0.875	0.977	1
GO_ACTIN_FILAMENT_POLYMERIZATION	23	-0.25	-0.8	0.745	0.977	1
GO_REGULATION_OF_RHO_PROTEIN_SIGNAL_TRANSDUCTION	105	-0.18	-0.8	0.867	0.977	1
GO_REGULATION_OF_NEURAL_PRECURSOR_CELL_PROLIFERATION	73	-0.19	-0.8	0.849	0.977	1
GO_NEGATIVE_REGULATION_OF_GLUCOSE_TRANSPORT	16	-0.28	-0.8	0.76	0.976	1
GO_REGULATION_OF_PHOSPHOLIPID_METABOLIC_PROCESS	60	-0.2	-0.8	0.829	0.978	1
GO_REACTIVE_OXYGEN_SPECIES_METABOLIC_PROCESS	96	-0.19	-0.8	0.849	0.978	1
GO_SECONDARY_METABOLIC_PROCESS	47	-0.21	-0.8	0.842	0.979	1
GO_CELLULAR_RESPONSE_TO_LEPTIN_STIMULUS	16	-0.28	-0.8	0.748	0.982	1
GO_PANCREAS_DEVELOPMENT	72	-0.19	-0.8	0.85	0.982	1
GO_NUCLEOSIDE_BISPHOSPHATE_BIOSYNTHETIC_PROCESS	17	-0.27	-0.8	0.773	0.983	1
GO_LIPID_HOMEOSTASIS	107	-0.18	-0.8	0.911	0.983	1
GO_CARBOHYDRATE_TRANSPORTER_ACTIVITY	43	-0.21	-0.8	0.823	0.983	1
GO_DRUG_TRANSPORT	25	-0.24	-0.8	0.785	0.983	1
GO_CATION_TRANSPORTING_ATPASE_COMPLEX	16	-0.27	-0.8	0.74	0.983	1
GO_INDOLE_CONTAINING_COMPOUND_METABOLIC_PROCESS	26	-0.24	-0.79	0.77	0.983	1
GO_ESTABLISHMENT_OF_PROTEIN_LOCALIZATION_TO_MEMBRANE	263	-0.16	-0.79	0.962	0.983	1
GO_INNER_EAR_RECEPTOR_CELL_DEVELOPMENT	34	-0.22	-0.79	0.803	0.983	1
GO_REGULATION_OF_LIPID_METABOLIC_PROCESS	274	-0.16	-0.79	0.971	0.984	1
GO_ENDOCHONDRAL_BONE_MORPHOGENESIS	45	-0.21	-0.79	0.842	0.984	1
GO_UBIQUITIN_DEPENDENT_PROTEIN_CATABOLIC_PROCESS_VIA_THE_MULTIVESICULAR_BODY_SORTING_PATHWAY	18	-0.27	-0.79	0.75	0.984	1
GO_STEROID_BINDING	91	-0.18	-0.79	0.866	0.984	1
GO_SIGNAL_PEPTIDE_PROCESSING	24	-0.25	-0.79	0.773	0.984	1
GO_GLUCOSAMINE_CONTAINING_COMPOUND_METABOLIC_PROCESS	26	-0.24	-0.79	0.799	0.984	1
GO_NEGATIVE_REGULATION_OF_PEPTIDYL_SERINE_PHOSPHORYLATIO	23	-0.24	-0.79	0.796	0.984	1
GO_POSITIVE_REGULATION_OF_BLOOD_VESSEL_ENDOTHELIAL_CELL_MIGRATION	25	-0.24	-0.79	0.774	0.984	1
GO_ENDOPLASMIC_RETICULUM_ORGANIZATION	36	-0.22	-0.79	0.809	0.984	1
GO_SULFATION	16	-0.27	-0.79	0.768	0.984	1
GO_MESENCHYMAL_CELL_DIFFERENTIATION	134	-0.17	-0.79	0.918	0.984	1
GO_SECONDARY_ACTIVE_TRANSMEMBRANE_TRANSPORTER_ACTIVITY	228	-0.16	-0.79	0.976	0.984	1
GO_PHOSPHOLIPID_BIOSYNTHETIC_PROCESS	233	-0.16	-0.79	0.964	0.983	1
GO_NEGATIVE_REGULATION_OF_PROTEIN_ACETYLATION	20	-0.25	-0.79	0.778	0.984	1
GO_POLYSACCHARIDE_BIOSYNTHETIC_PROCESS	42	-0.21	-0.79	0.834	0.984	1
GO_REGULATION_OF_ESTABLISHMENT_OR_MAINTENANCE_OF_CELL_POLARITY	21	-0.25	-0.79	0.786	0.984	1
GO_NEGATIVE_REGULATION_OF_RESPONSE_TO_OXIDATIVE_STRESS	35	-0.22	-0.79	0.833	0.984	1
GO_RESPONSE_TO_MAGNESIUM_ION	23	-0.25	-0.79	0.819	0.984	1
GO_OLFACTORY_LOBE_DEVELOPMENT	36	-0.21	-0.79	0.804	0.984	1
GO_DEMETHYLASE_ACTIVITY	34	-0.22	-0.79	0.815	0.984	1
GO_SINGLE_FERTILIZATION	109	-0.18	-0.79	0.891	0.984	1
GO_ORGANIC_HYDROXY_COMPOUND_TRANSPORT	155	-0.17	-0.78	0.938	0.985	1
GO_PROTEIN_SERINE_THREONINE_PHOSPHATASE_ACTIVITY	62	-0.2	-0.78	0.866	0.986	1
GO_INSULIN_LIKE_GROWTH_FACTOR_RECEPTOR_BINDING	15	-0.27	-0.78	0.752	0.986	1
GO_ENDOTHELIAL_CELL_DEVELOPMENT	44	-0.21	-0.78	0.843	0.987	1
GO_CHONDROITIN_SULFATE_BIOSYNTHETIC_PROCESS	25	-0.24	-0.78	0.805	0.986	1
GO_REGULATION_OF_PROTEIN_AUTOPHOSPHORYLATION	36	-0.22	-0.78	0.809	0.987	1
GO_BINDING_BRIDGING	173	-0.16	-0.78	0.949	0.987	1
GO_NEURAL_CREST_CELL_MIGRATION	51	-0.2	-0.78	0.853	0.986	1

GO_REGULATION_OF_TRANSCRIPTION_FROM_RNA_POLYMERASE_III_PROMOTER	23	-0.24	-0.78	0.793	0.987	1
GO_EXTRACELLULAR_MATRIX	417	-0.15	-0.78	0.994	0.987	1
GO_NEGATIVE_REGULATION_OF_ERK1_AND_ERK2_CASCADE	52	-0.2	-0.78	0.86	0.99	1
GO_COENZYME_A_METABOLIC_PROCESS	17	-0.26	-0.78	0.793	0.99	1
GO_ORGANELLE_TRANSPORT_ALONG_MICROTUBULE	59	-0.19	-0.77	0.876	0.992	1
GO_REGULATION_OF_FIBROBLAST_GROWTH_FACTOR_RECEPTOR_SIGNALING_PATHWAY	25	-0.23	-0.77	0.828	0.993	1
GO_POSITIVE_REGULATION_OF_LIPID_METABOLIC_PROCESS	126	-0.17	-0.77	0.941	0.998	1
GO_TRABECULA_MORPHOGENESIS	39	-0.21	-0.77	0.861	0.997	1
GO_PLATELET_ALPHA_GRANULE	74	-0.18	-0.77	0.894	0.997	1
GO_AMMONIUM_TRANSMEMBRANE_TRANSPORT	24	-0.24	-0.77	0.801	0.998	1
GO_REGULATION_OF_PROTEIN_DEACETYLATION	34	-0.22	-0.77	0.831	0.998	1
GO_MORPHOGENESIS_OF_AN_ENDOTHELIUM	16	-0.27	-0.77	0.806	0.998	1
GO_MAINTENANCE_OF_LOCATION_IN_CELL	93	-0.18	-0.77	0.897	1	1
GO_POSITIVE_REGULATION_OF_EXOCYTOSIS	82	-0.18	-0.76	0.915	1	1
GO_CELL_PROLIFERATION_IN_FOREBRAIN	27	-0.23	-0.76	0.812	1	1
GO_ENDOCRINE_PROCESS	44	-0.21	-0.76	0.833	1	1
GO_REGULATION_OF_GLYCOPROTEIN_METABOLIC_PROCESS	43	-0.2	-0.76	0.837	1	1
GO_GAMMA_TUBULIN_COMPLEX	16	-0.26	-0.76	0.79	1	1
GO_PROTON_TRANSPORTING_TWO_SECTOR_ATPASE_COMPLEX_CATALYTIC_DOMAIN	17	-0.26	-0.76	0.81	1	1
GO_CELLULAR_RESPONSE_TO_CARBOHYDRATE_STIMULUS	74	-0.18	-0.76	0.901	1	1
GO_RESPONSE_TO_ISCHEMIA	28	-0.22	-0.76	0.842	1	1
GO_NEGATIVE_REGULATION_OF_SMOOTH_MUSCLE_CELL_MIGRATION	16	-0.26	-0.76	0.826	1	1
GO_ETHANOLAMINE_CONTAINING_COMPOUND_METABOLIC_PROCESS	84	-0.18	-0.76	0.94	1	1
GO_CADHERIN_BINDING	28	-0.22	-0.75	0.845	1	1
GO_NEGATIVE_REGULATION_OF_NEURAL_PRECURSOR_CELL_PROLIFERATION	21	-0.24	-0.75	0.836	1	1
GO_TRIVALENT_INORGANIC_CATION_TRANSPORT	38	-0.21	-0.75	0.887	1	1
GO_NEGATIVE_REGULATION_OF_MRNA_METABOLIC_PROCESS	28	-0.22	-0.75	0.859	1	1
GO_HYDROGEN_TRANSPORT	134	-0.16	-0.75	0.973	1	1
GO_PROTEIN_SECRETION	115	-0.17	-0.75	0.952	1	1
GO_ESTABLISHMENT_OR_MAINTENANCE_OF_EPITHELIAL_CELL_APICAL_BASAL_POLARITY	29	-0.22	-0.75	0.82	1	1
GO_CELL_DIFFERENTIATION_INVOLVED_IN_KIDNEY_DEVELOPMENT	36	-0.21	-0.75	0.88	1	1
GO_RESPONSE_TO_DIETARY_EXCESS	21	-0.24	-0.75	0.841	1	1
GO_CELL_FATE_DETERMINATION	43	-0.2	-0.75	0.871	1	1
GO_SYMPORTER_ACTIVITY	141	-0.16	-0.75	0.961	1	1
GO_STEROID_CATABOLIC_PROCESS	24	-0.23	-0.75	0.834	1	1
GO_NUCLEOTIDE_EXCISION_REPAIR_PREINCISION_COMPLEX_ASSEMBL	29	-0.22	-0.75	0.845	1	1
GO_POSITIVE_REGULATION_OF_OSTEOBLAST_DIFFERENTIATION	60	-0.19	-0.75	0.894	1	1
GO_NEGATIVE_REGULATION_OF_HISTONE_ACETYLATION	15	-0.26	-0.75	0.815	1	1
GO_REGULATION_OF_CGMP_BIOSYNTHETIC_PROCESS	22	-0.24	-0.75	0.845	1	1
GO_CELLULAR_COMPONENT_MAINTENANCE	17	-0.25	-0.75	0.836	1	1
GO_VERY_LONG_CHAIN_FATTY_ACID_METABOLIC_PROCESS	29	-0.22	-0.75	0.838	1	1
GO_ANION_CATION_SYMPORTER_ACTIVITY	53	-0.19	-0.75	0.896	1	1
GO_NEGATIVE_REGULATION_OF_DENDRITE_DEVELOPMENT	27	-0.23	-0.75	0.853	1	1
GO_REGULATION_OF_CARBOHYDRATE_CATABOLIC_PROCESS	42	-0.2	-0.74	0.87	1	1
GO_MICROBODY_LUMEN	44	-0.2	-0.74	0.886	1	1
GO_CHONDROCYTE_DIFFERENTIATION	60	-0.19	-0.74	0.909	1	1
GO_GROWTH_FACTOR_ACTIVITY	159	-0.16	-0.74	0.972	1	1
GO_REGULATION_OF_CELL_FATE_COMMITMENT	26	-0.22	-0.74	0.869	1	1
GO_REGULATION_OF_PROTEIN_TARGETING_TO_MITOCHONDRION	96	-0.17	-0.74	0.956	1	1
GO_POSITIVE_REGULATION_OF_TYROSINE_PHOSPHORYLATION_OF_STAT5_PROTEIN	16	-0.25	-0.74	0.835	1	1
GO_RESPONSE_TO_PROSTAGLANDIN_E	25	-0.23	-0.74	0.853	1	1
GO_ACTIN_MONOMER_BINDING	24	-0.23	-0.74	0.858	1	1
GO_REGULATION_OF_THE_FORCE_OF_HEART_CONTRACTION	29	-0.22	-0.74	0.863	1	1
GO_WATER_TRANSMEMBRANE_TRANSPORTER_ACTIVITY	15	-0.26	-0.74	0.834	1	1
GO_NEGATIVE_REGULATION_OF_GLYCOPROTEIN_METABOLIC_PROCES	15	-0.26	-0.74	0.835	1	1
GO_PALATE_DEVELOPMENT	84	-0.17	-0.74	0.925	1	1
GO_QUATERNARY_AMMONIUM_GROUP_BINDING	49	-0.19	-0.74	0.909	1	1
GO_ORGANIC_CATION_TRANSPORT	20	-0.24	-0.74	0.839	1	1
GO_LATE_ENDOSOME_MEMBRANE	97	-0.17	-0.74	0.945	1	1
GO_REGULATION_OF_STEM_CELL_DIFFERENTIATION	113	-0.16	-0.74	0.963	1	1
GO_RESPONSE_TO_ACIDIC_PH	21	-0.23	-0.74	0.872	1	1
GO_REGULATION_OF_LIPID_CATABOLIC_PROCESS	50	-0.19	-0.73	0.898	1	1
GO_NEGATIVE_REGULATION_OF_TRANSFORMING_GROWTH_FACTOR_BETA_RECEPTOR_SIGNALING_PATHWAY	64	-0.18	-0.73	0.913	1	1
GO_HISTONE_ACETYLTRANSFERASE_BINDING	28	-0.22	-0.73	0.893	1	1
GO_CARDIAC_RIGHT_VENTRICLE_MORPHOGENESIS	16	-0.25	-0.73	0.818	1	1
GO_MONOCARBOXYLIC_ACID_BIOSYNTHETIC_PROCESS	165	-0.16	-0.73	0.987	1	1
GO_COP9_SIGNALOSOME	34	-0.2	-0.73	0.892	1	1
GO_AUDITORY_RECEPTOR_CELL_DEVELOPMENT	17	-0.25	-0.73	0.834	1	1
GO_STEROL_TRANSPORT	50	-0.19	-0.73	0.918	1	1
GO_HIGH_DENSITY_LIPOPROTEIN_PARTICLE_REMODELING	15	-0.26	-0.73	0.841	1	1
GO_SULFUR_COMPOUND_CATABOLIC_PROCESS	39	-0.2	-0.73	0.885	1	1
GO_CARDIAC_MUSCLE_TISSUE_MORPHOGENESIS	54	-0.19	-0.73	0.908	1	1
GO_REGULATION_OF_HISTONE_DEACETYLATION	24	-0.23	-0.73	0.861	1	1
GO_REGULATION_OF_GLIAL_CELL_DIFFERENTIATION	59	-0.18	-0.73	0.933	1	1
GO_ACTIN_CYTOSKELETON_REORGANIZATION	55	-0.18	-0.73	0.909	1	1
GO_POSITIVE_REGULATION_OF_RESPONSE_TO_OXIDATIVE_STRESS	15	-0.26	-0.73	0.86	1	1
GO_REGULATION_OF_INSULIN_RECEPTOR_SIGNALING_PATHWAY	43	-0.19	-0.73	0.919	1	1
GO_ERYTHROCYTE_DEVELOPMENT	24	-0.22	-0.72	0.876	1	1
GO_2_OXOGLUTARATE_METABOLIC_PROCESS	20	-0.23	-0.72	0.834	1	1
GO_REGULATION_OF_ACTIN_NUCLEATION	26	-0.21	-0.72	0.875	1	1

GO_POSITIVE_REGULATION_OF_CHONDROCYTE_DIFFERENTIATION	19	-0.24	-0.72	0.848	1	1
GO_LENS_FIBER_CELL_DIFFERENTIATION	25	-0.22	-0.72	0.881	1	1
GO_ORGANONITROGEN_COMPOUND_CATABOLIC_PROCESS	341	-0.14	-0.72	0.999	1	1
GO_PLASMA_MEMBRANE_FUSION	26	-0.22	-0.72	0.891	1	1
GO_MATING_BEHAVIOR	23	-0.23	-0.72	0.883	1	1
GO_REGULATION_OF_CARBOHYDRATE_METABOLIC_PROCESS	170	-0.15	-0.72	0.99	1	1
GO_RESPIRATORY_GASEOUS_EXCHANGE	48	-0.19	-0.72	0.915	1	1
GO_NEGATIVE_REGULATION_OF_SMOOTH_MUSCLE_CONTRACTION	15	-0.25	-0.72	0.839	1	1
GO_REGULATION_OF_FATTY_ACID_BIOSYNTHETIC_PROCESS	35	-0.2	-0.72	0.914	1	1
GO_ENERGY_DERIVATION_BY_OXIDATION_OF_ORGANIC_COMPOUNDS	216	-0.15	-0.72	0.992	1	1
GO_AMINE_BIOSYNTHETIC_PROCESS	21	-0.23	-0.72	0.848	1	1
GO_MESENCHYMAL_TO_EPITHELIAL_TRANSITION	15	-0.25	-0.72	0.865	1	1
GO_ACTIN_FILAMENT	70	-0.17	-0.72	0.951	1	1
GO_CHD_TYPE_COMPLEX	17	-0.24	-0.72	0.854	1	1
GO_GLYOXYLATE_METABOLIC_PROCESS	27	-0.21	-0.72	0.882	1	1
GO_REGULATION_OF_OXIDATIVE_PHOSPHORYLATION	16	-0.25	-0.72	0.856	1	1
GO_NEGATIVE_REGULATION_OF_STEM_CELL_DIFFERENTIATION	43	-0.19	-0.71	0.894	1	1
GO_NEGATIVE_REGULATION_OF_PROTEIN_SECRETION	105	-0.16	-0.71	0.968	1	1
GO_ENDOCYTIC_VESICLE_MEMBRANE	149	-0.15	-0.71	0.984	1	1
GO_L_AMINO_ACID_TRANSMEMBRANE_TRANSPORTER_ACTIVITY	54	-0.18	-0.71	0.941	1	1
GO_HIGH_DENSITY_LIPOPROTEIN_PARTICLE	26	-0.22	-0.71	0.884	1	1
GO_SODIUM_ION_HOMEOSTASIS	29	-0.21	-0.71	0.885	1	1
GO_NEGATIVE_REGULATION_OF_PLATELET_ACTIVATION	17	-0.24	-0.71	0.844	1	1
GO_LIPID_OXIDATION	68	-0.17	-0.71	0.941	1	1
GO_CELL_AGGREGATION	21	-0.23	-0.71	0.889	1	1
GO_RESPONSE_TO_LEPTIN	20	-0.23	-0.71	0.899	1	1
GO_TRANSFERASE_ACTIVITY_TRANSFERRING_NITROGENOUS_GROUPS	23	-0.22	-0.71	0.878	1	1
GO_POSITIVE_REGULATION_OF_NEUROBLAST_PROLIFERATION	21	-0.22	-0.71	0.87	1	1
GO_METANEPHRIC_NEPHRON_DEVELOPMENT	32	-0.2	-0.71	0.918	1	1
GO_TASTE_RECEPTOR_ACTIVITY	29	-0.21	-0.71	0.909	1	1
GO_DICARBOXYLIC_ACID_TRANSMEMBRANE_TRANSPORTER_ACTIVITY	33	-0.2	-0.71	0.908	1	1
GO_EMBRYONIC_DIGIT_MORPHOGENESIS	60	-0.18	-0.71	0.95	1	1
GO_REGULATION_OF_SYSTEMIC_ARTERIAL_BLOOD_PRESSURE_MEDIATED_BY_A_CHEMICAL_SIGNAL	45	-0.19	-0.71	0.952	1	1
GO_POSITIVE_REGULATION_OF_ALCOHOL_BIOSYNTHETIC_PROCESS	23	-0.22	-0.7	0.888	1	1
GO_FATTY_ACID_CATABOLIC_PROCESS	71	-0.17	-0.7	0.966	1	1
GO_CELL_FATE_SPECIFICATION	71	-0.17	-0.7	0.973	1	1
GO_SEMAPHORIN_PLEXIN_SIGNALING_PATHWAY	36	-0.19	-0.7	0.92	1	1
GO_ATPASE_ACTIVITY_COUPLED_TO_TRANSMEMBRANE_MOVEMENT_OF_IONS_PHOSPHORYLATIVE_MECHANISM	33	-0.2	-0.7	0.915	1	1
GO_POSITIVE_REGULATION_OF_ORGANIC_ACID_TRANSPORT	29	-0.21	-0.7	0.89	1	1
GO_PHOSPHATIDYLINOSITOL_3_KINASE_SIGNALING	25	-0.21	-0.7	0.885	1	1
GO_PARAXIAL_MESODERM_DEVELOPMENT	16	-0.24	-0.7	0.895	1	1
GO_POSITIVE_REGULATION_OF_PROTEIN_DEACETYLATION	18	-0.23	-0.7	0.899	1	1
GO_SULFUR_COMPOUND_TRANSMEMBRANE_TRANSPORTER_ACTIVITY	27	-0.21	-0.69	0.901	1	1
GO_FIBROBLAST_GROWTH_FACTOR_RECEPTOR_SIGNALING_PATHWAY	83	-0.16	-0.69	0.969	1	1
GO_OLFACTORY_RECEPTOR_ACTIVITY	369	-0.13	-0.69	0.999	1	1
GO_LAMELLIPODIUM_ORGANIZATION	36	-0.19	-0.69	0.913	1	1
GO_SUBSTANTIA_NIGRA_DEVELOPMENT	44	-0.18	-0.69	0.945	1	1
GO_POSITIVE_REGULATION_OF_CARTILAGE_DEVELOPMENT	29	-0.21	-0.69	0.905	1	1
GO_CELL_MIGRATION_INVOLVED_IN_SPROUTING_ANGIOGENESIS	15	-0.24	-0.69	0.892	1	1
GO_ACID_THIOL_LIGASE_ACTIVITY	20	-0.22	-0.69	0.894	1	1
GO_REGULATION_OF_SMAD_PROTEIN_IMPORT_INTO_NUCLEUS	16	-0.24	-0.69	0.884	1	1
GO_CHEMOSENSORY_BEHAVIOR	15	-0.24	-0.69	0.901	1	1
GO_BRUSH_BORDER_MEMBRANE	55	-0.17	-0.69	0.964	1	1
GO_SEROTONIN_RECEPTOR_ACTIVITY	18	-0.23	-0.69	0.881	1	1
GO_REGULATION_OF_TRIGLYCERIDE_BIOSYNTHETIC_PROCESS	17	-0.23	-0.69	0.887	1	1
GO_INORGANIC_ANION_EXCHANGER_ACTIVITY	21	-0.22	-0.68	0.919	1	1
GO_VESICLE_COAT	45	-0.18	-0.68	0.956	1	1
GO_VENTRICULAR_CARDIAC_MUSCLE_TISSUE_DEVELOPMENT	45	-0.18	-0.68	0.947	1	1
GO_AORTA_DEVELOPMENT	41	-0.19	-0.68	0.93	1	1
GO_REGULATION_OF_LIPID_TRANSPORT	93	-0.16	-0.68	0.981	1	1
GO_HYDROGEN_PEROXIDE_METABOLIC_PROCESS	30	-0.2	-0.68	0.921	1	1
GO_HISTONE_DEUBIQUITINATION	21	-0.21	-0.68	0.895	1	1
GO_DORSAL_VENTRAL_AXIS_SPECIFICATION	20	-0.22	-0.68	0.903	1	1
GO_PHOSPHATIDYLCHOLINE_BIOSYNTHETIC_PROCESS	27	-0.2	-0.68	0.935	1	1
GO_REGULATION_OF_MESODERM_DEVELOPMENT	15	-0.23	-0.68	0.872	1	1
GO_REGULATION_OF_NEUROBLAST_PROLIFERATION	28	-0.2	-0.68	0.91	1	1
GO_HYDROLASE_ACTIVITY_HYDROLYZING_O_GLYCOSYL_COMPOUNDS	91	-0.16	-0.67	0.99	1	1
GO_BLOOD_COAGULATION_INTRINSIC_PATHWAY	17	-0.23	-0.67	0.916	1	1
GO_INSULIN_LIKE_GROWTH_FACTOR_BINDING	25	-0.21	-0.67	0.934	1	1
GO_PRE_AUTOPHAGOSOMAL_STRUCTURE_MEMBRANE	15	-0.23	-0.67	0.916	1	1
GO_BONE_CELL_DEVELOPMENT	22	-0.21	-0.67	0.905	1	1
GO_HORMONE_BIOSYNTHETIC_PROCESS	49	-0.17	-0.67	0.963	1	1
GO_ESTABLISHMENT_OF_PROTEIN_LOCALIZATION_TO_VACUOLE	30	-0.2	-0.67	0.932	1	1
GO_WNT_ACTIVATED_RECEPTOR_ACTIVITY	22	-0.21	-0.67	0.922	1	1
GO_PHOSPHATASE_INHIBITOR_ACTIVITY	31	-0.19	-0.67	0.928	1	1
GO_LEUKOTRIENE_BIOSYNTHETIC_PROCESS	20	-0.22	-0.67	0.913	1	1
GO_JAK_STAT_CASCADE_INVOLVED_IN_GROWTH_HORMONE_SIGNALING_PATHWAY	15	-0.23	-0.67	0.906	1	1
GO_VENTRICULAR_SEPTUM_MORPHOGENESIS	28	-0.2	-0.67	0.916	1	1
GO_POSITIVE_REGULATION_OF_SIGNAL_TRANSDUCTION_BY_P53_CLASS_MEDIATOR	17	-0.22	-0.67	0.914	1	1
GO_NUCLEAR_TRANSCRIPTIONAL_REPRESSOR_COMPLEX	22	-0.21	-0.67	0.914	1	1
GO_ORGANIC_ACID_TRANSMEMBRANE_TRANSPORT	103	-0.15	-0.67	0.994	1	1
GO_NOTCH_RECEPTOR_PROCESSING	16	-0.23	-0.67	0.903	1	1
GO_REGULATION_OF_METANEPHROS_DEVELOPMENT	23	-0.21	-0.66	0.941	1	1

GO_GLUTATHIONE_DERIVATIVE_METABOLIC_PROCESS	21	-0.21	-0.66	0.915	1	1
GO_AXIS_SPECIFICATION	90	-0.15	-0.66	0.995	1	1
GO_NEPHRON_TUBULE_FORMATION	18	-0.22	-0.66	0.93	1	1
GO_PSEUDOPODIUM	17	-0.22	-0.66	0.906	1	1
GO_PROTEIN_DEALKYLATION	29	-0.19	-0.66	0.952	1	1
GO_TRACHEA_DEVELOPMENT	20	-0.21	-0.66	0.929	1	1
GO_SPERM_CAPACITATION	17	-0.22	-0.66	0.906	1	1
GO_POSITIVE_REGULATION_OF_MACROPHAGE_DERIVED_FOAM_CELL_DIFFERENTIATION	15	-0.23	-0.66	0.909	1	1
GO_CARDIAC_ATRIUM_DEVELOPMENT	31	-0.19	-0.66	0.95	1	1
GO_POSITIVE_REGULATION_OF_NEURAL_PRECURSOR_CELL_PROLIFERATION	42	-0.18	-0.66	0.961	1	1
GO_B_CELL_PROLIFERATION	38	-0.18	-0.66	0.95	1	1
GO_LIGASE_ACTIVITY_FORMING_CARBON_SULFUR_BONDS	40	-0.18	-0.66	0.963	1	1
GO_REGULATION_OF_EARLY_ENDOSOME_TO_LATE_ENDOSOME_TRANSPORT	16	-0.23	-0.65	0.912	1	1
GO_RECEPTOR_MEDIATED_ENDOCYTOSIS	211	-0.13	-0.65	1	1	1
GO_PROTEIN_TRANSMEMBRANE_TRANSPORT	50	-0.17	-0.65	0.969	1	1
GO_POSITIVE_REGULATION_OF_ENDOCYTOSIS	113	-0.14	-0.65	0.995	1	1
GO_REGULATION_OF_CALCINEURIN_NFAT_SIGNALING_CASCADE	17	-0.22	-0.65	0.926	1	1
GO_GOLGI_ASSOCIATED_VESICLE	82	-0.15	-0.64	0.989	1	1
GO_POSITIVE_REGULATION_OF_INSULIN_SECRETION_INVOLVED_IN_CELLULAR_RESPONSE_TO_GLUCOSE_STIMULUS	27	-0.19	-0.64	0.946	1	1
GO_THYROID_GLAND_DEVELOPMENT	25	-0.19	-0.64	0.956	1	1
GO_REGULATION_OF_SYSTEMIC_ARTERIAL_BLOOD_PRESSURE_BY_HORMONE	35	-0.18	-0.64	0.972	1	1
GO_CELLULAR_ANION_HOMEOSTASIS	15	-0.22	-0.64	0.917	1	1
GO_POSITIVE_REGULATION_OF_LIPID_BIOSYNTHETIC_PROCESS	65	-0.15	-0.63	0.985	1	1
GO_SEMAPHORIN_RECEPTOR_BINDING	22	-0.2	-0.63	0.944	1	1
GO_INTRINSIC_COMPONENT_OF_EXTERNAL_SIDE_OF_PLASMA_MEMBR	24	-0.2	-0.63	0.954	1	1
GO_PEPTIDYL_GLUTAMIC_ACID_MODIFICATION	29	-0.19	-0.63	0.961	1	1
GO_AMINO_ACID_TRANSMEMBRANE_TRANSPORTER_ACTIVITY	79	-0.15	-0.63	0.997	1	1
GO_HISTONE_H3_K4_METHYLATION	32	-0.18	-0.63	0.955	1	1
GO_NEGATIVE_REGULATION_OF_TRANSCRIPTION_REGULATORY_REGION_DNA_BINDING	17	-0.21	-0.63	0.959	1	1
GO_REGULATION_OF_RECEPTOR_RECYCLING	19	-0.21	-0.63	0.952	1	1
GO_RECEPTOR_SIGNALING_COMPLEX_SCAFFOLD_ACTIVITY	23	-0.19	-0.63	0.957	1	1
GO_REGULATION_OF_CARTILAGE_DEVELOPMENT	62	-0.15	-0.62	0.986	1	1
GO_CELLULAR_RESPONSE_TO_STEROL	15	-0.22	-0.62	0.944	1	1
GO_SOMITE_DEVELOPMENT	77	-0.15	-0.62	1	1	1
GO_PROTEIN_PHOSPHATASE_TYPE_2A_COMPLEX	20	-0.2	-0.62	0.965	1	1
GO_NUCLEOTIDE_EXCISION_REPAIR_DNA_DUPLEX_UNWINDING	22	-0.19	-0.62	0.955	1	1
GO_RESPONSE_TO_THYROID_HORMONE	22	-0.19	-0.61	0.951	1	1
GO_GLUTATHIONE_PEROXIDASE_ACTIVITY	18	-0.2	-0.61	0.952	1	1
GO_TRANSCRIPTION_ELONGATION_FACTOR_COMPLEX	45	-0.16	-0.61	0.979	1	1
GO_PHOSPHOTRANSFERASE_ACTIVITY_FOR_OTHER_SUBSTITUTED_PHOSPHATE_GROUPS	19	-0.2	-0.61	0.959	1	1
GO_AMMONIUM_TRANSMEMBRANE_TRANSPORTER_ACTIVITY	27	-0.18	-0.61	0.969	1	1
GO_PROTON_TRANSPORTING_TWO_SECTOR_ATPASE_COMPLEX_PROTON_TRANSPORTING_DOMAIN	21	-0.19	-0.6	0.964	1	1
GO_REGULATION_OF_CGMP_METABOLIC_PROCESS	29	-0.17	-0.6	0.972	1	1
GO_NEUTRAL_LIPID_METABOLIC_PROCESS	83	-0.14	-0.6	0.995	1	1
GO_SEGMENT_SPECIFICATION	17	-0.2	-0.6	0.957	1	1
GO_PARASYMPATHETIC_NERVOUS_SYSTEM_DEVELOPMENT	17	-0.2	-0.59	0.972	1	1
GO_NEGATIVE_REGULATION_OF_NF_KAPPAB_IMPORT_INTO_NUCLEUS	18	-0.2	-0.59	0.97	1	1
GO_NEUROTRANSMITTER_BINDING	30	-0.17	-0.59	0.977	1	1
GO_METANEPHRIC_NEPHRON_MORPHOGENESIS	21	-0.19	-0.59	0.962	1	1
GO_RNA_POLYMERASE_II_CORE_BINDING	19	-0.19	-0.59	0.961	1	1
GO_MONOSACCHARIDE_TRANSMEMBRANE_TRANSPORTER_ACTIVITY	20	-0.19	-0.58	0.972	1	1
GO_RESPONSE_TO_MUSCLE_STRETCH	19	-0.19	-0.58	0.976	1	1
GO_SULFUR_AMINO_ACID_BIOSYNTHETIC_PROCESS	19	-0.19	-0.58	0.971	1	1
GO_NEGATIVE_REGULATION_OF_ORGAN_GROWTH	21	-0.18	-0.58	0.977	1	1
GO_ALCOHOL_TRANSMEMBRANE_TRANSPORTER_ACTIVITY	24	-0.18	-0.57	0.977	1	1
GO_PROTEIN_SERINE_THREONINE_KINASE_ACTIVATOR_ACTIVITY	19	-0.18	-0.56	0.983	1	1
GO_C21_STEROID_HORMONE_BIOSYNTHETIC_PROCESS	15	-0.19	-0.56	0.967	1	1
GO_PIGMENT_GRANULE_ORGANIZATION	23	-0.17	-0.56	0.991	1	1
GO_AMINO_SUGAR_METABOLIC_PROCESS	40	-0.15	-0.55	0.998	1	1
GO_POSITIVE_REGULATION_OF_FATTY_ACID_METABOLIC_PROCESS	33	-0.16	-0.55	0.995	1	1
GO_CARDIAC_SEPTUM_MORPHOGENESIS	49	-0.14	-0.55	0.993	1	1
GO_PERICARDIUM_DEVELOPMENT	18	-0.18	-0.55	0.973	1	1
GO_SULFURIC_ESTER_HYDROLASE_ACTIVITY	17	-0.19	-0.55	0.98	1	1
GO_NEGATIVE_REGULATION_OF_CARTILAGE_DEVELOPMENT	25	-0.17	-0.54	0.989	1	1
GO_NEGATIVE_REGULATION_OF_AXON_EXTENSION	38	-0.15	-0.54	1	1	1
GO_POSITIVE_REGULATION_OF_CELLULAR_RESPONSE_TO_INSULIN_STIMULUS	23	-0.17	-0.54	0.987	1	1
GO_RAC_GTPASE_BINDING	37	-0.15	-0.54	0.996	1	1
GO_NEUROTRANSMITTER_SODIUM_SYMPORTER_ACTIVITY	19	-0.17	-0.53	0.989	1	1
GO_PROTEIN_LIPID_COMPLEX_ASSEMBLY	21	-0.17	-0.53	0.993	1	1
GO_REGULATION_OF_CELL_PROLIFERATION_INVOLVED_IN_HEART_MORPHOGENESIS	15	-0.18	-0.53	0.993	1	1
GO_HYDROGEN_ION_TRANSMEMBRANE_TRANSPORT	102	-0.12	-0.53	1	1	1
GO_ORGANIC_ACID_SODIUM_SYMPORTER_ACTIVITY	30	-0.15	-0.52	0.996	1	1
GO_EXTRINSIC_COMPONENT_OF_ORGANELLE_MEMBRANE	23	-0.16	-0.52	0.995	1	1
GO_RESPONSE_TO_IMMOBILIZATION_STRESS	22	-0.16	-0.52	0.987	1	1
GO_MITOCHONDRIAL_RESPIRATORY_CHAIN_COMPLEX_ASSEMBLY	74	-0.13	-0.52	1	1	1
GO_MRNA_TRANSCRIPTION_FROM_RNA_POLYMERASE_II_PROMOTER	15	-0.18	-0.51	0.991	1	1

GO_VESICLE_MEDIATED_TRANSPORT_BETWEEN_ENDOSOMAL_COMPARTMENTS	20	-0.16	-0.49	0.989	1	1
GO_NEGATIVE_REGULATION_OF_NUCLEOSIDE_METABOLIC_PROCESS	19	-0.16	-0.49	0.991	1	1
GO_PHOSPHATIDYLCHOLINE_BINDING	19	-0.16	-0.49	0.998	1	1
GO_REGULATION_OF_MACROPHAGE_DERIVED_FOAM_CELL_DIFFERENTIATION	28	-0.15	-0.49	0.997	1	1
GO_POSITIVE_REGULATION_OF_STEROID_METABOLIC_PROCESS	23	-0.15	-0.48	0.998	1	1
GO_NEGATIVE_REGULATION_OF_EXOCYTOSIS	27	-0.14	-0.48	0.993	1	1
GO_FILOPODIUM_ASSEMBLY	17	-0.16	-0.48	0.995	1	1
GO_ARTERY_MORPHOGENESIS	51	-0.12	-0.48	1	1	1
GO_SENSORY_PERCEPTION_OF_CHEMICAL_STIMULUS	463	-0.09	-0.46	1	1	1
GO_PROTEIN_KINASE_C_ACTIVITY	16	-0.15	-0.45	1	1	1
GO_MITOCHONDRIAL_RESPIRATORY_CHAIN_COMPLEX_I_BIOGENESIS	55	-0.11	-0.44	1	0.999	1

Preranked gene set enrichment analysis results using the correlation levels of individual genes with TMB. Similarly shown with Supplementary Table 1.
