# Supplementary material for: Correlation-based and feature-driven mutation signature analyses to identify genetic features associated with DNA mutagenic processes in cancer genomes
Source: Genomics Inform. 2021 Dec 31;19(4):e40. doi: 10.5808/gi.21047 (PMC8752981; doi:10.5808/gi.21047)
Supplement: Supplemental Table 3. — Correlation table of DNA damage and repair gene expression and mutation signature levels [file gi-21047suppl7.pdf]

Supplementary Table 3. Correlation table of DNA damage and repair gene expression and mutation signature levels

DDR gene	Sig1	Sig2	Sig3	Sig4	Sig5	Sig6	Sig7	Sig8	Sig9	Sig10	Sig11	Sig12	Sig13	Sig14	Sig15	Sig16	Sig17	Sig18	Sig19	Sig20	Sig21	Sig22	Sig23	Sig24	Sig25	Sig26	Sig27	Sig28	Sig29	Sig30
MLH1	0.016	0.133	0.074	0.053	0.025	-0.552	0.117	0.028	0.033	0.037	0.055	0.008	0.113	-0.059	-0.062	0.024	0.026	0.037	0.043	-0.057	-0.029	0.028	0.049	0.074	0.018	-0.116	0.016	0.02	0.045	0.043
ALKBH3	-0.023	0.051	0.044	0.034	0.018	-0.215	0.093	0.036	-0.013	-0.061	0.025	-0.003	0.068	-0.045	-0.025	0.03	-0.037	0.027	0.02	-0.023	-0.005	-2E-04	0.015	0.043	0.012	-0.019	0.001	-0.017	0.036	0.007
HLTF	-0.17	0.13	0.13	0.099	-0.002	-0.135	-0.005	0.002	-0.009	0.001	0.005	-0.028	0.136	0.015	-0.065	0.01	-0.008	-0.021	-0.025	0.005	-0.026	-0.019	0.009	0.055	-0.01	-6E-04	0.029	-0.044	0.012	#####
RECQL	-0.151	0.105	0.096	0.104	5E-04	-0.133	0.044	0.03	0.003	-0.043	0.02	-0.043	0.13	-0.021	-0.061	-0.037	0.003	0.031	0.004	0.029	-0.018	-0.084	0.008	0.033	-0.006	-0.008	0.012	-0.018	0.025	0.013
PER1	-0.193	-0.037	-0.006	0.168	0.125	-0.105	0.012	0.081	0.017	-0.066	0.042	0.095	-0.047	0.017	-0.021	0.093	-0.05	0.051	0.038	0.101	0.043	0.077	0.031	0.08	0.062	0.005	0.001	0.058	0.042	0.048
PPP4R4	-0.142	-0.022	-0.029	0.153	0.078	-0.102	-0.036	-0.028	-0.026	-0.083	0.015	0.034	0.031	-0.037	-0.003	0.185	-0.072	0.004	0.036	3E-04	-0.014	0.087	8E-04	0.137	0.016	0.007	-0.011	-0.049	0.012	0.024
MSH6	-0.156	0.163	0.103	0.126	-0.046	-0.101	0.008	-0.043	-0.034	-0.01	0.004	-0.092	0.196	-0.061	-0.076	-0.008	-0.054	-0.023	-0.036	-0.088	-0.055	-0.066	-0.027	0.086	-0.031	-0.032	0.018	-0.083	0.026	-0.009
MBD4	-0.167	0.163	0.123	0.092	0.007	-0.098	-0.086	-0.003	0.017	5E-04	-0.009	-0.014	0.154	-0.014	-0.072	-0.018	-0.001	-0.048	0.013	-0.026	-0.028	-0.023	-0.032	0.061	-0.004	1E-04	0.031	-0.032	0.009	0.007
NSMCE2	-0.039	0.021	0.1	-0.022	0.01	-0.094	-0.011	-0.01	0.014	-0.021	0.013	-0.01	0.061	-0.023	-0.044	0.094	-0.008	-0.009	0.038	-0.058	-0.012	0.037	-0.015	0.047	-0.008	-0.007	0.005	-0.051	0.013	0.011
EID3	-0.131	0.026	0.145	0.119	0.021	-0.094	-0.03	0.056	0.005	-0.03	-0.008	-0.014	0.025	0.017	-0.05	-0.058	-0.009	0.014	-1E-04	0.002	-0.003	-0.03	0.013	0.066	0.019	-0.016	0.034	-0.002	0.045	0.028
TREX2	-0.108	0.111	0.084	0.01	0.022	-0.09	0.083	0.063	-0.004	-0.041	0.033	0.007	0.108	-0.021	-0.05	-0.014	-0.077	0.03	0.022	0.012	-0.032	-0.03	0.018	-0.005	0.035	-0.029	0.004	0.004	-0.005	0.014
PPP4R1	-0.184	0.276	-0.087	0.192	-0.069	-0.086	0.026	-0.044	-0.063	-0.006	-0.044	-0.089	0.317	-0.031	-0.089	0.005	-0.005	0.008	-0.056	-0.028	-0.06	-0.106	-0.059	0.008	-0.032	-0.028	-0.02	-0.073	-0.007	-0.029
APLF	-0.029	0.044	0.094	-0.031	0.025	-0.081	0.012	0.004	0.011	-0.029	0.021	-0.005	0.084	-0.004	-0.042	0.021	-0.045	-0.004	0.002	-0.001	-0.028	-0.031	-0.006	0.017	-0.01	-0.006	0.003	-0.036	0.015	0.007
ERCC4	-0.036	-0.002	0.081	0.092	-0.043	-0.08	-0.004	0.039	0.002	-0.033	0.022	-0.048	-0.005	0.029	-0.02	-0.052	-0.013	0.052	-0.026	0.027	-0.007	-0.044	0.035	0.025	-0.017	-0.021	0.028	-0.015	0.02	0.006
RECQL5	-0.133	0.09	0.038	0.021	0.051	-0.075	0.063	-0.007	0.043	-0.006	0.008	0.067	0.079	0.008	-0.026	0.056	-0.094	-0.02	0.049	0.048	-0.012	0.046	0.025	0.036	0.023	-0.012	0.007	-3E-04	-0.015	-0.001
ERCC3	-0.076	0.036	0.052	0.152	-0.065	-0.073	0.114	0.002	-0.014	-0.015	0.004	-0.072	0.075	-0.009	-0.047	-0.078	-0.104	-0.006	-0.025	-0.013	-0.041	-0.089	0.006	0.035	-0.029	-0.002	0.032	-0.045	0.021	-0.04
MRE11A	0.068	0.077	0.051	0.052	-0.116	-0.065	-0.069	-0.03	-0.029	0.009	-0.049	-0.132	0.102	-0.022	-0.034	-0.125	0.123	-0.018	-0.084	-0.088	-0.079	-0.134	-0.042	-0.009	-0.043	-0.047	0.065	-0.051	0.024	-0.058
ATR	0.015	0.113	0.045	0.019	-0.068	-0.062	-0.056	-0.036	-0.005	-0.027	-0.039	-0.094	0.127	-0.042	-0.048	-0.034	0.109	0.007	-0.057	-0.017	-0.07	-0.101	-0.044	-0.018	-0.033	-0.014	0.023	-0.037	-0.017	-0.06
RAD9B	0.084	0.135	0.021	-0.048	-0.073	-0.06	-0.051	-0.026	0.005	-0.013	-0.032	-0.042	0.118	-0.03	-0.016	-0.055	0.011	-0.032	-0.011	-0.089	-0.02	-0.065	-0.025	-0.019	-0.009	-0.039	0.04	-0.047	0.002	-0.035
GADD45A	-0.109	0.024	0.009	0.019	0.106	-0.06	-0.052	0.014	-0.007	-0.014	0.016	0.092	0.036	-7E-04	-0.02	0.092	-0.048	-0.018	0.042	0.04	0.029	0.086	-0.01	0.05	0.043	0.02	-0.002	0.041	0.035	0.042
POLE4	-0.037	0.122	-0.032	0.029	-0.036	-0.057	0.109	-0.039	-0.008	0.021	-0.02	-0.047	0.142	-0.033	-0.048	-0.012	-0.068	-0.013	-0.002	-0.068	-0.03	-0.03	-0.007	-0.006	-0.04	-0.029	-0.008	-0.033	-0.009	-0.02
DDB1	-0.1	-0.017	0.105	0.039	-0.009	-0.055	0.104	0.008	-0.006	-0.011	0.029	-0.049	0.033	-0.011	-0.026	0.005	-0.053	0.055	-0.006	0.022	-0.021	-0.039	0.039	0.046	-0.02	-0.008	0.034	-0.025	0.03	0.01
SMC6	-0.055	0.139	-0.007	0.06	-0.046	-0.055	-0.005	-0.046	-0.029	-0.017	-0.028	-0.064	0.164	-0.031	-0.038	4E-04	0.03	-0.031	-0.026	-0.082	-0.077	-0.064	-0.049	0.031	-0.03	-0.024	1E-03	-0.063	0.009	-0.044
HFM1	0.024	-0.142	0.096	0.03	-0.027	-0.053	0.064	0.065	-0.003	-0.042	0.047	4E-04	-0.116	0.013	0.043	-0.063	0.011	0.015	0.01	0.032	0.003	-0.021	0.062	0.03	0.021	-0.006	0.022	0.015	0.033	0.011
RAD1	-0.121	0.132	0.039	0.229	-0.097	-0.053	-0.036	-0.032	-0.046	0.002	-0.06	-0.116	0.148	-0.011	-0.076	-0.087	-0.029	-0.015	-0.06	-0.063	-0.048	-0.105	-0.057	0.059	-0.037	-0.024	0.005	-0.066	0.02	-0.044
YWHAG	-0.046	0.009	-0.019	0.132	-0.013	-0.052	0.037	-0.025	-0.014	-0.04	-2E-04	-0.064	0.069	-0.027	-0.043	0.016	-0.045	0.019	-0.03	-0.016	-0.037	-0.059	0.005	-0.009	-0.009	0.009	-0.035	-0.046	-0.015	-0.023
RNMT	-0.08	-0.013	0.111	0.101	-0.059	-0.05	0.082	0.002	-0.016	-0.028	0.024	-0.081	0.024	-8E-04	-0.035	-0.054	0.002	0.038	-0.016	-0.007	-0.03	-0.088	0.019	0.035	-0.015	-0.016	0.009	-0.045	0.019	-0.008
BRE	-0.073	-0.074	0.137	-0.046	0.093	-0.049	0.019	0.08	0.073	-0.023	0.033	0.105	-0.089	0.028	7E-04	0.002	-0.036	0.01	0.07	0.08	0.038	0.064	0.049	-0.001	0.033	0.015	0.016	0.083	0.038	0.048
ATM	-0.074	-0.114	0.088	0.07	0.036	-0.049	0.062	0.061	0.038	-0.052	0.043	0.031	-0.112	0.023	0.009	-0.063	0.097	0.029	0.023	0.079	0.031	-0.029	0.076	0.024	-4E-04	0.013	0.049	0.038	0.022	0.027
TCEB1	-0.071	0.031	0.02	0.069	0.009	-0.048	0.015	-0.048	9E-04	-0.036	-0.002	-0.008	0.085	-0.028	-0.066	0.067	-0.026	-0.019	0.021	-0.072	-0.016	0.018	-0.021	0.044	-0.025	0.012	-0.002	-0.049	-0.02	-0.02
RPA2	0.008	0.183	0.033	-0.024	-0.086	-0.047	0.043	-0.005	0.008	0.05	0.005	-0.05	0.129	0.003	-0.02	-0.11	-0.089	-0.025	-0.014	-0.028	0.008	-0.074	0.005	-0.027	-0.017	-0.017	0.032	-0.028	-0.015	-0.005
SMUG1	-0.1	0.111	-0.013	0.042	0.009	-0.045	0.064	-0.049	0.002	-0.012	-0.005	0.015	0.113	-0.015	-0.03	0.035	-0.049	-0.014	6E-04	-0.025	-0.016	0.029	-0.012	0.02	0.015	-0.009	-0.01	-0.034	-0.027	-0.005
RNF168	-0.043	0.119	0.018	0.087	-0.071	-0.044	-0.035	-0.043	-0.049	-0.022	-0.038	-0.119	0.139	-0.035	-0.053	-0.034	0.084	0.017	-0.071	-0.047	-0.055	-0.119	-0.047	0.016	-0.04	-0.018	0.031	-0.064	0.022	-0.054
PMS2	-0.063	-0.041	0.095	-0.011	0.036	-0.042	0.103	0.051	0.069	0.006	0.054	0.041	-0.03	0.038	-0.011	-0.024	-0.073	0.019	0.051	0.098	0.012	-0.027	0.073	-0.036	0.05	0.007	-6E-04	0.044	-0.021	0.021
TOPBP1	0.028	0.155	0.02	0.022	-0.114	-0.041	-0.017	-0.055	-0.04	-0.019	-0.034	-0.138	0.178	-0.026	-0.033	-0.072	0.059	-0.021	-0.071	-0.105	-0.066	-0.13	-0.046	-0.001	-0.046	-0.021	0.029	-0.094	0.003	-0.069
PRKDC	0.088	0.06	-0.032	0.048	-0.109	-0.041	0.019	-0.045	-0.037	-0.032	-0.011	-0.156	0.107	-0.043	-0.03	-0.031	0.05	-5E-04	-0.072	-0.105	-0.075	-0.14	-0.041	0.004	-0.055	-0.029	0.01	-0.084	-0.011	-0.073
REV1	-0.007	0.139	0.004	0.054	-0.071	-0.041	-0.094	-0.014	-0.035	0.003	-0.045	-0.063	0.149	0.002	-0.022	-0.057	0.006	-0.003	-0.052	-0.022	-0.046	-0.094	-0.044	0.015	-0.025	-0.037	0.026	-0.047	0.017	-0.043
PALB2	0.076	0.204	-0.059	-0.016	-0.127	-0.039	0.049	-0.06	-0.015	0.009	-0.055	-0.129	0.186	-0.03	-0.031	-0.061	0.022	-8E-04	-0.093	-0.082	-0.082	-0.126	-0.043	-0.051	-0.048	-0.04	0.012	-0.067	-0.032	-0.067
POLB	-0.094	0.147	-0.017	0.112	-0.083	-0.035	0.059	-0.058	-0.035	0.001	-0.028	-0.075	0.163	-0.035	-0.07	0.012	-0.082	-0.019	-0.052	-0.043	-0.027	-0.028	-0.018	0.036	-0.026	1E-03	-0.007	-0.065	-0.034	-0.016
ALKBH1	-0.003	0.031	0.006	0.035	-0.04	-0.034	0.034	-0.006	-0.007	0.02	0.004	-0.064	0.038	0.025	1E-03	-0.04	-0.018	0.03	-0.034	-0.025	-0.028	-0.041	-0.01	-0.01	-0.011	-0.018	-0.004	-0.032	0.016	-0.02
ERCC1	-0.04	0.038	0.002	0.006	-0.006	-0.034	0.137	0.02	-0.008	-0.018	0.031	0.002	0.042	0.007	-0.03	-0.003	-0.158	0.009	0.033	0.016	0.008	-0.005	0.029	0.007	0.01	-0.014	-0.01	-0.006	0.02	0.011
CETN2	-0.015	-5E-04	0.148	0.003	-0.003	-0.033	-0.075	0.022	0.032	0.033	-0.014	0.021	-0.017	0.044	-0.054	-0.059	-0.065	0.016	-0.01	0.069	0.005	-0.021	0.004	-0.018	0.01	-0.007	-0.009	0.037	0.003	0.027
GTF2H4	-0.169	0.066	0.078	0.033	0.022	-0.032	0.107	-0.024	0.014	0.024	0.026	0.05	0.093	0.003	-0.071	0.046	-0.128	-0.032	0.034	0.016	-0.032	0.044	-0.01	0.062	0.015	0.024	0.021	-0.03	-0.009	0.001
UBE2A	0.115	0.114	-0.081	0.003	-0.12	-0.03	-0.016	-0.053	-0.026	-0.008	-0.027	-0.102	0.13	-0.026	-0.019	-0.068	0.006	0.02	-0.049	-0.082	-0.06	-0.087	-0.033	-0.032	-0.048	-0.044	-0.02	-0.082	0.003	-0.04
MSH3	0.083	0.047	-0.057	-0.107	0.026	-0.029	-0.029	-0.009	0.003	0.043	-0.013	3E-04	-0.005	-0.013	0.037	0.042	0.012	-0.006	-0.01	0.026	0.01	0.027	0.018	-0.049	0.002	0.023	-0.006	0.031	0.01	0.032
DDB2	-0.19	0.152	1E-03	-0.038	0.119	-0.028	0.112	0.022	0.043	0.025	0.013	0.085	0.113	0.013	-0.061	0.054	-0.085	-0.01	0.067	0.095	0.005	0.033	0.035	-0.011	0.043	0.033	0.009	0.06	-0.033	0.043
MDC1	-0.023	-0.041	0.053	0.056	-0.083	-0.028	0.085	-0.016	-0.009	0.013	0.026	-0.036	-0.028	0.016	-0.003	-0.044	0.053	0.005	-0.028	-0.038	-0.032	-0.044	0.008	0.047	-0.014	-0.028	0.041	-0.04	0.035	-0.053
FANCL	0.066	0.159	0.024	-0.056	-0.101	-0.028	0.049	-0.06	-0.031	0.021	-0.026	-0.09	0.142	-0.024	-0.026	-0.091	-0.008	-0.048	-0.044	-0.099	-0.032	-0.079	-0.011	-0.018	-0.046	-0.017	0.028	-0.068	-0.039	-0.039
NSMCE1	0.035	-0.01	-0.004	-0.121	0.077	-0.027	0.039	0.044	0.071	-0.004	0.021	0.095	-0.076	0.007	0.015	0.063	-0.089	-0.008	0.085	0.045	0.047	0.076	0.048	-0.023	0.049	0.011	-0.017	0.054	-0.015	0.033
YWHAB	0.253	0.098	-0.032	-0.161	-0.14	-0.027	0.03	-0.064	-0.014	-0.011	-0.045	-0.148	0.118	-0.029	0.003	-0.116	0.166	0.018	-0.092	-0.099	-0.069	-0.159	-0.047	-0.134	-0.055	-0.071	-0.011	-0.049	-0.032	-0.082
ERCC6	-0.041	0.175	0.026	0.069	-0.088	-0.026	-0.094	-0.025	-0.034	0.003	-0.043	-0.099	0.186	-0.011	-0.04	-0.057	0.012	0.017	-0.079	-0.023	-0.066	-0.125	-0.062	0.005	-0.042	-0.019	0.011	-0.059	-0.006	-0.025
SHPRH	0.058	-0.03	0.063	0.042	-0.051	-0.025	-0.082	0.01	-0.016	-0.028	-0.015	-0.078	-0.021	-0.008	0.008	-0.083	0.09	0.033	-0.051	0.01	-0.023	-0.105	-0.008	-0.003	-0.029	-0.005	0.026	-0.01	0.02	-0.026
PLK3	-0.007	0.156	-0.028	-0.017	-0.098	-0.025	0.139	-0.016	0.008	-0.003	0.014	-0.059	0.161	-0.007	-0.042	-0.071	0.028	0.008	-0.021	-0.034	-0.042	-0.116	-0.018	-0.075	-0.035	-0.02	-0.005	-0.029	0.01	-0.046
NUDT1	0.068	0.146	-0.083	-0.05	-0.098	-0.025	0.119	-0.046	-0.011	0.019	-0.006	-0.09	0.172	-0.023	-0.021	-0.023	-0.042	-0.017	-0.012	-0.107	-0.067	-0.088	-0.027	-0.05	-0.024	-0.043	-0.012	-0.07	-0.022	-0.078
GTF2H1	0.015	-0.051	-0.028	0.018	-0.032	-0.025	0.078	0.014	-0.028	-0.025	-0.006	-0.072	-0.023	-0.003	0.013	-0.013	0.079	0.04	-0.005	-0.03	-0.002	-0.04	0.002	0.032	-0.026	-0.002	-0.003	-0.033	0.002	0.005
PARP3	-0.027	0.009	-0.025	0.013	0.018	-0.024	0.083	0.018	0.038	-0.035	0.04	0.045	-0.036	0.019	0.011	-0.007	-0.052	0.013	0.043	0.071	0.045	0.039	0.039	-0.019	0.041	0.008	0.001	0.05	0.005	0.033
DMC1	-0.046	0.117	0.046	0.03	-0.06	-0.024	-0.003	-0.012	0.008	-0.004	-0.033	-0.048	0.12	-0.016	-0.06	-0.021	0.01	-0.04	-0.005	-0.039	-0.041	-0.057	-0.028	0.009	-0.019	-0.036	0.04	-0.031	-0.005	-0.013
POLG	-0.08	0.024	0.01	0.002	0.018	-0.023	0.127	0.009	-0.024	-0.012	0.036	-0.007	0.038	0.005	4E-04	-0.014	0.031	0.014	0.009	-0.024	-0.007	-0.034	0.02	0.005	0.008	0.003	-0.009	0.003	0.023	-0.003
RFC1	-0.085	-0.013	0.038	0.029	0.052	-0.023	-0.009	0.025	0.005	-0.023	0.039	0.019	-0.03	0.015	0.019	0.057	-0.022	0.011	0.024	-0.012	0.04	0.046	0.037	0.064	5E-04	3E-04	0.011	-0.007	0.024	0.015
FANCE	0.025	0.189	-0.081	0.074	-0.14	-0.021	0.033	-0.077	-0.065	5E-04	-0.05	-0.161	0.238	-0.035	-0.038	-0.043	-0.051	-0.019	-0.078	-0.129	-0.101	-0.138	-0.071	0.012	-0.06	-0.04	-4E-04	-0.127	-0.002	-0.082
RAD51C	-0.093	0.119	0.018	2E-04	-0.022	-0.02	0.086	-0.005	-0.009	0.004	0.019	-0.033	0.106	0.014	-0.018	-0.007	-0.054	-0.026	0.037	-0.029	0.005	-0.03	0.029	0.021	0.002	0.003	0.009	-0.033	0.006	-0.025
RPA1	-0.064	0.091	0.075	0.018	-0.042	-0.019	0.093	0.009	-0.004	0.023	0.028	-0.082	0.091	0.01	-0.047	-0.063	-0.045	-0.002	-0.015	-0.003	-0.035	-0.112	0.006	-0.01	-0.007	-0.019	-0.005	-0.031	0.005	-0.035
RNF169	-0.066	0.025	0.062	0.12	-0.065	-0.016	-0.046	0.016	-0.035	-0.031	-0.005	-0.094	0.051	0.005	-0.029	-0.084	0.064	0.042	-0.065	0.012	-0.029	-0.108	-0.008	0.036	-0.029	-0.012	0.036	-0.028	0.051	-0.024
RPA4	0.066	-0.08	0.007	-0.017	-0.009	-0.016	-0.039	0.007	0.003	-0.028	-0.005	-0.001	-0.07	-0.025	0.033	-0.035	0.152	-0.006	0.002	-0.061	-0.013	-0.008	-0.007	0.029	-0.007	0.01	0.026	-0.01	0.028	-0.018
RPA3	-0.018	0.062	-0.019	-0.022	-0.004	-0.015	-0.013	-0.038	0.047	-0.002	-0.034	0.02	0.055	-0.02	-0.037	0.047	0.094	-0.039	0.03	-0.05	-0.006	0.032	-0.024	-0.023	-0.002	-0.014	-0.012	0.003	-0.026	-0.007
RFC2	-0.042	0.14	-0.006	-0.007	-0.032	-0.014	0.097	-0.026	-0.006	0.032	0.006	-0.035	0.14	-0.018	-0.076	-0.002	-0.1	-0.043	0.008	-0.063	-0.042	-0.045	-0.005	-0.012	-0.009	-0.006	-0.004	-0.046	-0.024	-0.024
PPP4C	-0.05	0.193	-0.05	0.004	-0.052	-0.013	0.033	-0.06	0.005	0.062	-0.044	-0.018	0.165	-0.011	-0.059	0.015	-0.094	-0.024	0.005	-0.044	-0.037	-0.009	-0.041	-0.034	-0.009	-0.019	-0.013	-0.048	-0.028	-0.022
HELQ	-0.11	-0.025	0.099	0.018	0.049	-0.012	-0.007	0.079	0.008	-0.019	0.048	0.024	-0.032	0.035	0.002	-0.008	-0.029	0.038	0.029	0.045	0.041	-0.005	0.029	0.039	3E-04	0.008	0.047	0.016	0.045	0.051
MAD2L2	-0.069	0.059	-0.038	0.026	-0.038	-0.012	0.119	-0.028	-0.015	0.031	0.017	-0.012	0.095	-0.007	-0.026	0.045	-0.098	-0.024	0.018	-0.054	-0.016	0.016	0.015	0.024	-0.02	0.006	-0.003	-0.052	-0.004	-0.002
HES1	0.093	0.224	-0.118	-0.087	-0.07	-0.011	-0.005	-0.042	-0.045	0.061	-0.069	-0.041	0.206	-0.031	-0.043	-0.023	0.012	-0.017	-0.102	-0.013	-0.044	-0.06	-0.066	-0.083	-0.023	-0.018	-0.1	-0.035	-0.035	-0.08
RFC4	-0.023	0.15	0.05	-0.005	-0.07	-0.009	-0.014	-0.07	-0.021	0.022	-0.041	-0.098	0.171	-0.023	-0.063	-0.022	-0.011	-0.062	-0.02	-0.114	-0.064	-0.077	-0.054	0.019	-0.021	-0.01	0.022	-0.082	-0.005	-0.049
POLI	-0.07	0.012	0.094	0.039	-0.002	-0.009	-0.02	0.054	0.032	0.019	0.026	0.035	-0.02	0.051	-0.006	-0.096	-0.064	0.01	-6E-04	0.097	0.033	-0.042	0.033	0.025	0.024	0.027	0.025	0.024	0.006	0.033
RRM2B	-0.112	-0.066	0.094	0.079	0.082	-0.009	-0.063	0.04	0.038	-0.055	0.032	0.048	-0.062	0.022	-0.003	0.014	-0.006	0.025	0.016	0.11	0.053	0.01	0.044	0.02	0.003	0.047	-4E-04	0.066	-0.007	0.049
POLM	0.029	-0.02	-0.017	-0.029	0.004	-0.007	0.083	0.006	0.037	-0.02	0.008	0.038	-0.058	0.001	0.03	-0.052	0.025	-0.021	0.038	0.022	0.006	0.027	0.025	-0.012	0.029	-0.002	0.004	-0.002	#####	-0.014
CLK2	-0.112	0.137	0.07	0.039	-0.035	-0.007	0.038	-0.035	-0.016	0.005	-0.007	-0.021	0.128	8E-04	-0.027	-0.068	-0.035	-0.064	-0.003	-0.05	-0.036	-0.018	-0.027	0.036	-0.004	-0.005	0.027	-0.046	0.019	-0.016
PLRG1	-0.033	0.004	0.001	0.027	-8E-04	-0.007	0.027	-0.012	-0.015	-0.004	0.012	-0.042	0.028	0.004	-0.02	0.066	-0.042	0.005	0.006	-0.002	-0.004	-0.02	0.004	0.022	-0.019	-0.006	-0.008	-0.043	0.009	0.005
RIF1	0.021	0.021	0.045	0.071	-0.079	-0.007	-0.073	-0.006	-0.031	-0.024	-0.024	-0.111	0.054	-0.02	-0.016	-0.09	0.107	0.016	-0.085	-0.015	-0.038	-0.131	-0.033	-0.009	-0.044	-0.015	0.027	-0.03	0.02	-0.035
KAT5	0.014	-0.015	0.021	-0.018	-0.002	-0.007	0.082	0.003	0.014	-0.012	0.005	-0.022	-0.004	-0.01	0.004	-0.033	-0.038	0.049	0.004	0.032	0.015	-0.035	0.024	-0.028	0.001	-0.003	0.028	0.011	-0.01	-0.014
RBX1	-0.087	0.129	-0.011	0.001	-0.009	-0.006	0.076	-0.03	-0.007	0.025	-0.015	-0.009	0.156	-0.011	-0.052	0.013	-0.07	-0.025	0.024	-0.028	-0.015	-0.035	-0.014	-0.005	-0.02	0.003	-0.007	-0.02	-0.027	-0.011
RAD52	0.083	-0.073	0.093	-0.038	-0.013	-0.005	-0.051	0.016	0.024	-0.023	-0.006	0.01	-0.081	0.03	0.037	-0.067	-0.011	-0.014	0.011	0.006	-0.013	-0.034	0.006	-0.002	0.012	0.018	0.057	-0.013	0.01	-0.001
MORF4L1	-0.083	-0.006	0.126	0.012	-0.021	-0.005	5E-04	0.006	-0.017	-0.002	-0.002	-0.016	0.009	0.014	-0.013	-0.059	0.105	0.026	-0.014	0.012	0.018	-0.051	0.001	0.002	0.001	7E-04	0.034	-0.001	0.019	0.025
OGG1	0.081	-0.131	0.087	-0.14	0.079	-0.003	-0.035	0.047	0.094	-0.008	0.039	0.129	-0.176	0.021	0.052	0.019	-0.062	-0.023	0.091	0.073	0.042	0.12	0.067	-0.002	0.055	0.058	0.01	0.049	0.002	0.052
RAD23A	-0.027	0.031	0.016	-0.073	0.048	-0.002	0.076	0.01	-0.003	0.027	0.024	0.03	0.043	0.011	-0.02	0.039	-0.166	-0.006	0.039	-0.009	0.001	0.019	-6E-04	0.013	0.02	0.004	-0.01	-0.005	0.016	0.01
CDC5L	0.006	-0.005	0.034	0.008	-0.054	-0.001	-0.002	-0.034	-0.044	-0.02	0.003	-0.081	0.035	-0.013	0.008	0.005	0.084	-0.013	-0.027	-0.08	-0.025	-0.025	-0.023	0.032	-0.028	-0.01	0.005	-0.056	0.028	-0.021
PARP2	-0.036	0.124	0.024	-0.001	-0.051	-6E-04	-0.008	-0.036	-0.018	0.028	-0.005	-0.059	0.131	0.022	-0.015	-0.003	-0.047	-0.033	-0.008	-0.092	-0.036	-0.041	-0.022	0.027	-0.036	-0.008	0.027	-0.066	0.012	-0.026
CDC25B	0.128	0.01	-0.104	0.022	-0.128	8E-04	0.055	-0.059	-0.029	-0.003	-0.031	-0.117	0.061	-0.021	0.009	-0.03	0.085	-0.024	-0.059	-0.123	-0.069	-0.081	-0.05	-0.026	-0.047	-0.034	-7E-04	-0.055	-0.026	-0.062
POLD4	-0.069	-0.016	-0.109	0.026	0.096	0.001	-0.038	-0.008	0.047	-0.041	0.006	0.084	-0.039	-0.026	0.028	0.114	0.027	0.013	0.076	0.004	0.037	0.132	-0.013	0.039	0.028	0.008	-0.006	0.007	4E-04	0.03
LIG4	0.04	-0.015	-0.048	0.019	-0.022	0.001	-0.046	0.006	0.003	-0.035	-0.016	-0.011	-0.004	0.01	0.02	-9E-04	0.139	-0.012	-0.016	0.008	0.015	-0.045	-0.004	-0.026	-0.023	0.006	-0.001	-0.008	-0.019	-0.029
XPC	-0.042	-0.016	0.033	-0.123	0.074	0.002	0.078	0.073	0.065	-0.004	0.062	0.114	-0.084	0.03	0.043	0.01	-0.015	0.014	0.047	0.103	0.063	0.097	0.071	-0.003	0.044	0.048	-0.008	0.064	0.026	0.058
POLA1	0.222	0.125	-0.042	-0.134	-0.152	0.002	0.012	-0.021	-0.029	0.013	-0.029	-0.152	0.093	-0.026	0.013	-0.132	0.077	-0.009	-0.077	-0.131	-0.053	-0.133	-0.012	-0.053	-0.05	-0.035	0.006	-0.074	0.012	-0.066
LIG3	0.012	0.004	-0.022	0.104	-0.084	0.002	0.017	-0.044	-0.031	0.02	-0.017	-0.108	0.018	-8E-04	-0.008	-0.045	-0.002	-0.014	-0.057	-0.044	-0.052	-0.067	-0.034	0.018	-0.048	-0.014	0.006	-0.068	0.005	-0.05
HUS1	0.058	-0.082	-0.047	0.047	-0.037	0.004	0.04	-0.048	-0.025	-0.013	-0.021	-0.054	-0.027	-0.041	-0.002	-0.001	0.06	0.02	-0.018	-0.036	-0.039	-0.024	-0.027	0.007	-0.003	-0.022	-0.005	-0.049	-0.019	-0.034
TP53BP1	0.087	0.073	0.014	-0.002	-0.135	0.006	0.027	0.014	0.01	0.055	-4E-04	-0.12	0.052	0.036	-0.005	-0.156	-3E-04	0.03	-0.088	0.009	-0.031	-0.188	-0.011	-0.062	-0.038	-0.024	0.008	-0.021	0.016	-0.044
SHFM1	-0.064	0.052	-0.038	-0.033	0.013	0.008	0.034	-0.042	-0.003	0.007	-0.01	0.02	0.09	-0.032	-0.044	0.113	0.058	-0.033	0.028	-0.07	-0.028	0.051	-0.035	0.01	0.007	0.003	-0.023	-0.044	-0.018	-0.026
ASCC3	0.013	0.067	-0.068	0.041	-0.039	0.009	-0.088	-0.029	-0.053	-0.009	-0.025	-0.058	0.106	-0.03	-0.021	-0.003	0.121	-0.004	-0.043	-0.034	-0.019	-0.08	-0.037	-0.021	-0.038	0.003	-0.005	-0.016	-0.017	-0.028
RAD23B	-0.061	0.103	-0.056	0.04	-0.015	0.009	-0.071	-0.033	-0.055	-0.01	-0.04	-0.067	0.102	-0.031	-1E-03	0.067	0.027	0.003	-0.02	-0.081	-0.015	0.014	-0.024	0.052	-0.039	-0.013	-0.006	-0.062	0.034	-0.011
MRPL40	-0.196	-0.024	0.027	0.03	0.142	0.009	0.048	0.02	0.032	3E-04	0.047	0.146	-0.031	0.011	-0.026	0.137	-0.152	-0.015	0.084	0.095	0.057	0.129	0.036	0.058	0.048	0.046	-0.027	0.05	-0.025	0.046
RAD50	0.124	-0.018	0.002	-0.078	-0.016	0.009	-0.129	0.005	0.012	-0.008	-0.015	-0.04	-0.054	0.006	0.032	-0.005	0.093	0.015	-0.025	-0.011	-0.005	-0.03	-0.007	-0.029	-0.009	-0.021	0.005	0.02	0.026	0.018
MSH2	0.017	0.105	0.082	0.046	-0.137	0.01	-0.031	-0.066	-0.028	0.034	-0.041	-0.141	0.127	9E-04	-0.046	-0.115	0.019	-0.039	-0.076	-0.087	-0.082	-0.134	-0.04	-7E-04	-0.045	-0.018	0.037	-0.082	-0.014	-0.046
HERC2	0.127	-0.012	-0.071	-0.088	-0.044	0.01	0.093	0.008	-0.01	-0.003	0.006	-0.057	-0.014	-0.005	0.045	-0.024	0.057	0.027	-0.056	-0.016	-0.026	-0.085	0.024	-0.042	-0.011	-0.025	6E-04	-0.023	0.01	-0.042
EME2	0.1	0.016	-0.046	-0.056	-0.045	0.011	-0.005	0.005	0.019	0.005	-0.005	-0.026	-0.005	0.012	0.034	-0.043	0.038	-0.011	-0.008	-0.034	-0.021	-0.039	-0.015	-0.034	-0.013	-0.009	0.009	0.003	0.012	-0.036
MLH3	0.074	-0.097	0.005	-0.057	0.03	0.011	-0.021	0.036	0.037	-0.028	0.005	0.057	-0.125	0.023	0.029	-0.005	0.08	0.019	0.013	0.041	-0.01	0.03	0.023	-0.02	0.007	0.008	0.021	0.021	0.018	0.028
GADD45G	0.02	-0.104	-0.065	0.063	0.046	0.013	-0.154	0.023	0.007	-1E-03	0.006	0.069	-0.131	0.013	0.077	0.089	-0.053	-0.024	0.041	-0.013	0.049	0.146	0.008	0.069	0.034	0.021	-0.039	0.006	0.023	0.02
DCLRE1B	0.113	0.124	0.009	-0.047	-0.176	0.013	0.039	-0.018	-0.029	0.048	-0.021	-0.153	0.145	0.016	-0.007	-0.136	-1E-03	-0.031	-0.069	-0.124	-0.059	-0.15	-0.029	-0.047	-0.066	-0.026	0.026	-0.067	0.007	-0.046
XRCC1	0.049	0.177	-0.018	-0.128	-0.059	0.013	0.083	-0.007	-0.007	0.088	-0.006	-0.031	0.145	0.026	-0.034	-0.069	-0.099	-0.036	-0.023	-0.026	-0.028	-0.058	-0.042	-0.038	0.014	-0.012	-0.011	-0.017	-0.01	-0.031
GTF2H3	0.08	0.009	-0.036	0.083	-0.09	0.015	-0.063	-0.057	-0.033	-0.01	-0.033	-0.12	0.053	-0.018	-0.003	-0.053	0.027	0.001	-0.076	-0.059	-0.048	-0.133	-0.04	-0.009	-0.055	-0.011	0.007	-0.052	-0.012	-0.052
RAD9A	-0.034	0.136	-0.012	-0.049	-0.034	0.015	8E-04	-0.022	-0.002	0.041	-0.024	0.008	0.142	0.006	-0.015	-0.024	-0.043	-0.039	0.015	-0.037	-0.042	-0.013	-0.027	-0.007	7E-04	-0.002	0.032	-0.048	-0.014	-0.028
TOP3B	-0.059	0.076	-0.017	-1E-03	-0.024	0.015	0.067	-0.022	0.005	0.043	0.003	-0.002	0.097	0.016	-0.013	-0.001	-0.042	-0.034	0.019	-0.059	-0.033	-0.019	-0.029	0.013	-0.003	-0.014	0.021	-0.041	0.007	-0.06
APEX2	0.008	0.185	-0.05	-0.058	-0.085	0.016	0.06	-0.064	-0.009	0.06	-0.04	-0.064	0.195	-0.015	-0.055	-0.043	-0.061	-0.012	-0.029	-0.079	-0.064	-0.062	-0.058	-0.03	-0.018	-0.014	-0.019	-0.062	-0.004	-0.044
XAB2	0.005	0.035	-0.006	-0.114	0.023	0.016	0.061	0.031	0.001	0.047	0.029	0.035	0.016	0.026	0.025	0.048	-0.11	-0.01	0.044	-0.004	0.006	0.017	0.002	0.006	0.012	-9E-04	-0.003	-0.004	0.014	-0.012
POLH	-0.105	0.034	0.163	-0.113	0.024	0.016	0.125	0.046	0.05	0.025	0.046	0.051	-0.008	0.049	-0.001	-0.071	-0.003	-0.011	0.041	0.061	0.033	-0.017	0.055	-0.003	0.016	0.029	0.015	0.072	0.03	0.034
DUT	0.041	0.145	-0.04	-0.119	-0.048	0.016	0.015	-0.034	-0.006	0.044	-0.006	-0.037	0.124	0.007	0.018	0.025	-0.089	-0.025	0.005	-0.071	-0.019	-0.034	-0.011	-0.017	-0.011	-0.001	0.016	-0.043	0.011	-0.012
FANCC	-0.002	-0.036	0.074	-0.122	0.079	0.017	-0.04	0.057	0.031	0.04	-0.002	0.077	-0.063	0.023	0.019	0.001	0.021	-0.013	0.037	0.051	0.018	0.034	0.016	-0.008	0.017	0.017	-0.01	0.06	0.017	0.027
POLD2	0.045	-0.056	-0.003	-0.061	0.009	0.019	0.06	-0.01	0.009	0.027	0.027	0.02	-0.024	-3E-04	0.007	0.046	-0.118	-0.019	0.036	-0.023	-0.009	0.03	0.002	-0.015	0.025	7E-04	-0.034	-0.007	-0.015	-0.006
XPA	0.03	0.013	-0.069	-0.084	0.063	0.019	-0.079	0.023	-0.006	-0.022	-0.017	0.021	-0.042	-0.011	0.048	0.069	0.065	0.001	0.066	-0.031	0.046	0.044	0.007	0.04	0.007	0.003	0.006	0.013	0.012	0.017
UBE2B	0.033	0.006	0.004	-0.053	0.034	0.02	-0.103	0.009	-0.004	-0.022	-0.007	0.009	-0.01	0.006	0.026	0.027	-0.023	0.001	0.006	-0.034	0.037	0.055	-0.003	0.016	0.005	-0.01	-0.015	0.011	0.028	0.034
XRCC4	0.071	0.066	-0.045	-0.027	-0.033	0.02	-0.079	-0.015	0.022	-0.013	-0.042	-0.035	0.039	-2E-04	0.014	-0.033	0.112	-0.029	-0.014	-0.021	-0.016	-0.045	-0.029	-0.069	-0.012	-0.015	-0.012	0.01	-0.023	-0.026
MMS19	-0.061	0.028	-6E-04	0.058	-0.054	0.02	0.127	-0.031	0.009	0.038	0.004	-0.051	0.045	0.008	-0.01	-0.031	-0.086	0.019	-0.012	0.014	-0.049	-0.081	-8E-04	-0.009	-0.012	0.01	0.014	-0.016	-0.01	-0.012
WDR48	0.157	-0.016	0.082	-0.165	-0.064	0.021	-0.024	0.047	0.01	0.021	0.012	-0.014	-0.053	0.035	0.04	-0.086	-0.031	-1E-04	-0.016	0.053	0.011	-0.024	0.032	-0.044	0.007	0.015	0.03	0.008	-0.006	0.021
XRCC6	-0.095	0.082	-0.059	0.096	-0.031	0.021	0.06	-0.05	-0.026	0.046	-0.015	-0.055	0.131	-0.018	-0.037	0.009	-0.065	-0.03	-0.019	-0.038	-0.046	-0.074	-0.048	0.036	-0.031	-0.007	-0.005	-0.038	-0.038	-0.043
SETMAR	0.063	0.073	0.047	-0.078	-0.103	0.021	0.027	0.011	-0.017	0.058	6E-04	-0.045	0.049	0.02	-0.012	-0.108	-0.08	-0.024	-0.026	-0.026	-0.019	-0.012	-0.005	0.011	-0.009	0.004	0.02	-0.039	0.012	-0.003
ERCC8	0.148	-0.099	-0.047	0.03	-0.046	0.022	-0.064	-0.006	-0.003	0.012	-0.031	-0.052	-0.096	0.018	0.032	-0.048	1E-04	0.011	-0.028	0.014	-0.006	-0.075	0.015	-0.032	-0.023	0.007	0.004	2E-04	-0.014	-0.004
PTEN	-0.033	0.048	-0.006	-0.056	0.029	0.024	-0.074	0.009	-0.003	0.031	0.005	0.009	0.035	0.012	0.021	0.029	0.051	0.016	#####	0.008	0.026	-0.006	0.004	0.014	-0.01	-0.003	0.015	0.016	0.016	0.038
PARG	0.033	0.015	0.061	-0.022	-0.055	0.024	-0.106	-0.006	0.012	0.029	-0.039	-0.058	0.02	0.027	0.013	-0.06	0.084	0.009	-0.041	-0.015	-0.053	-0.073	-0.034	-0.008	-0.012	-0.004	-0.005	-0.008	0.009	-0.017
MNAT1	-0.067	0.128	-0.023	0.06	-0.05	0.024	-0.061	-0.048	-0.028	0.046	-0.041	-0.067	0.157	0.022	-0.032	0.035	-0.091	-0.014	-0.034	-0.04	-0.033	-0.047	-0.047	0.015	-0.027	-0.003	-0.008	-0.069	-0.009	-0.03
INO80	0.071	0.054	-0.114	-0.043	-0.042	0.025	0.03	9E-04	-0.014	-0.007	-0.002	-0.063	0.036	0.013	0.043	-0.03	0.118	0.031	-0.056	-0.032	-0.019	-0.092	-0.017	-0.031	-0.025	-0.011	-3E-04	-0.025	0.023	-0.041
NFATC2IP	0.083	0.171	-0.063	-0.033	-0.125	0.025	0.011	-0.045	0.009	0.001	-0.043	-0.102	0.126	-0.013	-0.008	-0.101	0.066	-0.045	-0.044	-0.084	-0.043	-0.089	-0.03	-0.057	-0.033	-0.032	0.031	-0.05	-0.023	-0.07
BCAS2	-0.025	0.118	0.051	-0.025	-0.048	0.026	-0.08	-0.037	-0.033	0.03	-0.038	-0.056	0.133	0.003	-0.035	-0.004	5E-04	-0.052	-0.015	-0.067	-0.007	-0.028	-0.042	0.004	-0.015	-0.003	0.018	-0.045	-0.02	-0.012
FANCM	0.004	0.162	-0.027	-0.007	-0.113	0.026	-0.034	-0.039	-0.031	0.03	-0.033	-0.122	0.179	9E-04	-0.014	-0.033	0.079	0.003	-0.071	-0.079	-0.065	-0.12	-0.05	-0.034	-0.058	-0.019	0.023	-0.065	-0.003	-0.062
UBE2V2	0.025	0.068	-0.048	0.026	-0.037	0.027	-0.08	-0.054	-0.039	0.001	-0.02	-0.077	0.13	-0.012	-0.041	0.049	-0.045	-0.024	-0.023	-0.11	-0.037	-0.041	-0.051	0.018	-0.036	0.007	-0.014	-0.079	-0.02	-0.037
BARD1	0.143	0.057	-0.01	0.038	-0.17	0.027	-0.044	-0.045	-0.059	6E-04	-0.04	-0.167	0.064	-0.005	0.008	-0.151	0.07	-0.008	-0.101	-0.085	-0.052	-0.168	-0.044	-0.03	-0.074	-0.036	0.024	-0.077	0.014	-0.066
GTF2H5	-0.054	-0.035	0.053	-0.062	0.058	0.027	-0.015	0.002	0.019	0.004	0.012	0.059	-0.034	0.019	-0.007	0.078	-0.009	-0.004	0.074	0.029	0.036	0.054	0.031	0.012	-5E-04	0.026	-0.014	0.012	0.004	0.025
RFC5	0.006	0.133	0.014	-0.006	-0.061	0.028	-0.03	-0.04	-0.025	0.034	-0.046	-0.077	0.122	-0.008	-0.03	-0.048	-0.041	-0.037	-0.025	-0.06	-0.043	-0.083	-0.047	-0.011	-0.017	-5E-04	0.018	-0.054	-0.019	-0.042

RBBP8	-0.089	0.033	0.09	0.128	-0.054	0.028	-0.086	-0.014	-0.009	0.067	-0.055	-0.065	0.031	0.035	-0.059	-0.086	-9E-04	0.001	-0.031	0.028	-0.045	-0.125	-0.041	0.025	-0.011	0.004	-0.003	-0.022	0.001	-0.005
RAD17	-0.038	-0.008	0.026	0.004	0.029	0.029	-0.007	0.033	0.035	0.019	0.008	0.013	-0.053	0.04	0.028	-0.017	-0.033	-0.009	0.01	0.04	0.032	0.004	0.021	0.002	0.008	0.022	0.015	0.038	0.018	0.042
UNG	0.041	0.079	0.071	-0.062	-0.014	0.03	-0.048	-0.064	0.012	0.036	-0.028	-0.048	0.107	-0.028	-0.031	-0.016	-0.113	-0.047	-0.029	-0.05	-0.048	-0.042	-0.044	-0.022	-0.01	0.02	0.025	-0.044	-0.038	-0.024
TDP2	0.058	-0.09	0.009	0.017	0.042	0.032	-0.155	-0.008	-0.005	-0.022	-0.016	0.019	-0.088	-0.024	0.008	0.042	0.003	-0.024	0.002	-0.036	0.026	0.043	-0.034	0.04	0.011	0.009	-0.01	8E-04	-0.018	0.012
RRM1	0.062	0.183	-0.101	-0.038	-0.11	0.033	-0.016	-0.054	-0.051	0.044	-0.041	-0.134	0.177	-0.015	-0.012	-0.044	0.024	-0.006	-0.061	-0.115	-0.053	-0.11	-0.051	-0.015	-0.048	-0.021	-0.013	-0.074	-8E-04	-0.051
TCEB2	0.104	-0.014	-0.046	-0.124	-0.008	0.033	0.033	0.001	0.022	0.028	-0.009	0.034	-0.036	-0.003	0.021	0.033	-0.031	-0.016	0.046	-0.023	0.003	0.044	-0.003	-0.043	0.01	-0.009	-0.028	0.007	-0.022	-0.01
PARP1	-0.014	0.081	-6E-04	0.029	-0.09	0.035	0.058	-0.078	-0.026	0.011	0.014	-0.125	0.096	-0.013	-0.008	-0.038	0.002	-0.05	-0.028	-0.107	-0.033	-0.067	-0.005	0.027	-0.044	-0.005	0.011	-0.08	-0.02	-0.028
FAM175A	0.036	-0.147	0.123	0.057	-0.029	0.035	-0.108	0.05	0.032	-0.039	0.017	-0.042	-0.164	0.013	0.009	-0.064	0.021	0.035	-0.011	0.036	0.022	-0.056	-0.003	0.051	-0.018	0.017	0.056	0.017	0.037	0.038
ERCC5	0.068	0.038	-0.024	-0.188	0.042	0.035	-0.036	0.037	0.022	-0.004	1E-04	0.046	0.007	0.011	0.052	0.003	0.117	-0.035	0.024	0.002	0.057	0.009	-0.005	-0.041	0.017	0.015	0.018	0.044	0.001	0.011
CUL4A	0.155	0.025	-0.048	-0.091	-0.071	0.035	-0.027	-0.044	-0.02	0.009	-0.041	-0.089	0.029	-0.012	0.035	-0.042	0.089	-0.041	-0.045	-0.079	-0.018	-0.067	-0.045	-0.036	-0.032	-0.021	0.011	-0.028	-8E-04	-0.04
PPP4R2	0.044	-0.028	0.005	0.037	-0.078	0.035	-0.032	-0.051	-0.033	0.003	-0.013	-0.079	-0.015	-0.014	-0.001	-0.052	0.097	0.01	-0.052	-0.028	-0.026	-0.037	-0.009	0.003	-0.036	-0.005	0.025	-0.043	0.014	-0.009
APITD1	-0.087	0.049	0.077	-0.003	0.029	0.036	-0.063	0.011	0.049	0.056	-0.022	0.034	0.002	0.031	-0.025	-0.05	0.009	-0.026	#####	0.053	0.022	-0.013	-0.005	-0.001	0.022	0.027	0.026	0.03	-0.017	0.016
ERCC2	0.012	-0.088	-0.033	-0.001	0.004	0.036	0.077	0.01	0.019	0.011	0.031	0.036	-0.08	0.024	0.015	0.021	-0.056	-0.016	0.035	0.011	-0.006	0.049	0.029	0.001	0.008	0.021	-0.016	0.01	-0.001	-0.011
APTX	-0.009	0.114	-0.058	-0.009	-0.044	0.038	-0.018	-0.037	-0.011	0.038	-0.029	-0.042	0.098	-0.006	-0.032	0.011	-0.032	-0.02	-3E-04	-0.04	-0.027	-0.03	-0.042	-0.01	-0.024	0.009	-0.019	-0.048	-0.021	-0.041
CUL3	0.101	-0.034	0.034	0.013	-0.071	0.038	-0.119	-0.035	-0.012	-0.005	-0.029	-0.081	-0.044	0.01	0.033	-0.068	0.084	0.017	-0.049	-0.026	-0.014	-0.062	-0.026	-0.013	-0.029	-0.008	0.002	-0.038	-0.028	-0.028
POLK	-0.027	-0.072	0.009	0.054	0.028	0.039	-0.076	0.042	0.008	0.004	0.008	0.004	-0.112	0.05	0.028	-0.039	0.031	0.023	-0.011	0.096	0.041	-0.009	0.037	0.015	-0.01	0.039	0.022	0.033	0.032	0.042
TCEA1	-0.019	-0.02	-0.01	0.124	-0.026	0.039	-0.119	-0.045	-0.02	1E-03	-0.013	-0.043	0.008	-0.005	-0.025	-0.004	0.01	-0.02	-0.013	-0.053	-0.01	-0.013	-0.046	0.044	-0.021	0.016	0.011	-0.045	-0.034	-0.016
USP1	0.104	0.101	0.072	-0.052	-0.157	0.039	-0.082	-0.027	-0.049	0.029	-0.04	-0.146	0.123	-0.005	0.002	-0.119	0.053	-0.034	-0.078	-0.13	-0.052	-0.136	-0.056	-0.031	-0.047	-0.023	0.038	-0.075	0.02	-0.046
SMARCAD1	0.105	-0.019	0.018	0.023	-0.104	0.039	-0.033	-0.017	-0.013	0.01	-0.021	-0.101	-0.01	0.019	0.006	-0.118	0.054	0.014	-0.073	0.005	-0.023	-0.154	0.002	-0.056	-0.055	0.006	0.023	-0.018	-0.009	-0.021
DCLRE1C	0.046	0.153	0.014	-0.04	-0.165	0.039	-0.014	-0.044	-0.022	0.053	-0.057	-0.122	0.156	0.002	-0.024	-0.129	0.159	-0.021	-0.067	-0.071	-0.066	-0.153	-0.061	-0.052	-0.061	-0.014	0.044	-0.056	-0.015	-0.065
POLN	0.045	-0.077	0.056	-0.095	0.015	0.04	-0.041	0.053	0.01	0.038	0.03	0.037	-0.107	0.047	0.066	-0.007	-0.008	0.005	0.032	-0.012	0.059	0.04	0.032	0.013	-0.004	-0.009	0.01	0.018	0.014	0.021
POLD1	0.034	0.145	-0.087	-0.022	-0.104	0.04	0.064	-0.055	-0.016	0.065	-0.023	-0.059	0.154	0.002	-0.034	-0.025	-0.065	-0.05	-0.024	-0.086	-0.069	-0.062	-0.065	-0.025	-0.025	-0.016	0.007	-0.064	-0.024	-0.067
BRIP1	0.054	0.133	-0.043	0.007	-0.138	0.04	0.026	-0.068	-0.054	0.021	-0.034	-0.148	0.162	-0.026	-0.041	-0.079	0.03	-0.043	-0.075	-0.123	-0.074	-0.136	-0.041	-0.003	-0.065	-0.036	0.003	-0.09	0.017	-0.057
NBN	0.021	0.013	0.11	-0.062	-0.014	0.042	-0.113	0.005	0.004	-0.011	-0.005	-0.042	0.046	0.008	-0.001	-0.046	0.068	3E-04	-0.032	-0.012	0.008	-0.074	-0.019	-0.034	-0.03	0.038	0.023	0.001	-0.031	-0.004
MGMT	-0.104	0.025	0.037	-0.066	0.099	0.043	-0.069	0.012	0.023	0.047	-0.017	0.116	-0.016	0.025	-8E-04	0.117	-0.078	-0.047	0.043	0.042	0.047	0.148	-0.02	0.039	0.037	0.039	-0.004	0.037	0.005	0.044
POLE3	0.082	0.057	0.006	-0.015	-0.105	0.043	-0.051	-0.026	-0.025	0.043	-0.05	-0.124	0.075	0.006	-0.013	-0.057	-0.016	-0.011	-0.043	-0.077	-0.048	-0.102	-0.03	-0.035	-0.049	-0.024	0.033	-0.064	-0.006	-0.046
ATRX	0.118	-0.022	-0.006	-0.017	-0.084	0.045	-0.104	0.006	-0.016	-7E-04	-0.024	-0.086	-0.028	0.004	0.013	-0.083	0.172	0.018	-0.066	0.004	-0.019	-0.1	0.003	-0.054	-0.037	-0.017	0.016	0.005	-0.012	-0.012
RNF4	0.003	0.009	0.062	0.004	-0.093	0.046	0.035	0.005	0.017	0.054	0.016	-0.065	0.016	0.037	0.022	-0.139	-0.022	0.019	-0.034	-0.021	-0.002	-0.089	0.005	-0.042	-0.034	-0.008	0.025	-0.002	0.023	-0.004
SMC5	0.137	0.048	-0.028	-0.003	-0.104	0.047	-0.173	-0.028	-0.038	0.005	-0.074	-0.095	0.034	-0.005	0.033	-0.096	0.103	-0.012	-0.068	-0.073	-0.036	-0.095	-0.055	-0.005	-0.045	-0.024	0.03	-0.064	0.013	-0.066
MUTYH	0.143	0.023	-0.051	-0.092	-0.084	0.047	0.038	-0.054	0.015	0.035	-0.017	-0.033	0.034	-0.002	0.022	-0.02	-0.023	-0.079	-0.029	-0.058	-0.036	-0.03	-0.04	-0.043	-0.016	0.003	0.021	-0.055	-0.037	-0.067
UIMC1	0.114	0.016	-0.009	-0.116	-0.041	0.048	-0.056	-0.002	0.017	0.045	-0.018	-0.073	0.016	0.035	0.036	-0.037	0.032	0.005	-0.005	-0.055	-0.014	-0.064	-0.001	-0.031	-0.019	-0.007	-0.004	-0.009	0.038	-0.017
FANCB	0.113	0.151	-0.054	-0.042	-0.189	0.05	0.036	-0.082	-0.052	0.051	-0.06	-0.188	0.189	-0.02	-0.031	-0.1	0.076	-0.032	-0.092	-0.146	-0.092	-0.164	-0.066	-0.052	-0.085	-0.042	0.018	-0.114	-0.012	-0.085
ALKBH2	0.032	-0.018	0.008	-0.101	0.027	0.05	0.02	-0.027	0.008	0.014	0.034	0.022	-0.009	-0.002	0.023	0.076	-0.131	-0.047	0.038	-0.058	0.011	0.075	0.005	0.037	0.004	0.026	-0.001	-0.03	-0.004	0.003
MUS81	0.081	-0.022	-0.015	-0.043	-0.044	0.051	0.007	-0.009	0.019	0.039	-0.013	-0.019	-0.017	0.009	0.029	-0.055	-0.056	-0.012	0.019	-0.046	-0.023	-0.023	0.01	-0.015	-0.026	0.02	0.053	-0.037	0.008	-0.022
NEIL3	0.085	0.078	-0.048	0.003	-0.139	0.051	0.008	-0.069	-0.03	0.026	-0.03	-0.145	0.114	0.003	-0.025	-0.043	0.019	-0.053	-0.05	-0.15	-0.069	-0.12	-0.058	-0.002	-0.064	-0.024	0.026	-0.109	0.009	-0.058
RDM1	0.091	0.109	-0.02	-0.078	-0.091	0.051	-0.05	-0.063	0.01	0.04	-0.05	-0.065	0.099	-0.003	-0.02	-0.05	0.054	-0.081	-0.018	-0.1	-0.027	-0.046	-0.046	-0.037	-0.024	-0.006	0.013	-0.041	-0.043	-0.037
PNKP	-0.013	0.08	-0.078	-0.023	-0.017	0.051	0.078	-0.027	0.008	0.059	-0.012	0.029	0.067	-0.004	-0.032	-0.01	-0.073	-0.032	-0.002	0.018	-0.032	-0.002	-0.034	-0.038	0.006	0.011	-0.011	-0.008	-0.029	-0.022
WRN	0.097	0.004	0.001	0.013	-0.129	0.051	-0.044	-7E-04	-0.014	-0.005	-0.026	-0.113	0.01	-0.009	-0.014	-0.098	0.118	0.017	-0.071	-0.031	-0.031	-0.137	-0.027	-0.02	-0.046	0.003	0.024	-0.05	0.005	-0.053
ATRIP	0.075	0.175	-0.087	-0.172	-0.102	0.052	0.121	-0.028	-0.015	0.08	0.008	-0.061	0.137	0.021	0.004	-0.002	-0.084	-0.023	-0.014	-0.07	-0.023	-0.019	#####	-0.041	-0.025	-0.011	-0.005	-0.065	-0.004	-0.024
XRCC5	0.042	0.021	0.03	-0.01	-0.075	0.052	0.014	-0.041	-0.017	0.048	-0.034	-0.085	0.017	0.002	-0.009	-0.072	-0.015	-0.002	-0.046	-0.009	-0.025	-0.072	-0.026	-0.006	-0.036	0.003	0.017	-0.03	-0.031	-0.031
RAD54B	0.123	0.079	0.03	-0.059	-0.142	0.053	-0.022	-0.062	-0.039	0.04	-0.041	-0.136	0.118	-0.002	-0.044	-0.068	0.039	-0.053	-0.075	-0.129	-0.071	-0.121	-0.07	-0.045	-0.054	-0.011	0.019	-0.088	-0.04	-0.085
BRCA2	0.146	0.122	-0.096	-0.026	-0.158	0.054	-0.025	-0.052	-0.046	0.024	-0.061	-0.163	0.128	-0.004	-0.009	-0.113	0.14	-0.024	-0.1	-0.129	-0.068	-0.16	-0.062	-0.059	-0.071	-0.035	0.03	-0.073	-0.004	-0.084
SOX4	0.1	0.032	0.028	0.062	-0.164	0.055	-0.076	-0.007	-0.033	0.082	-0.073	-0.098	0.04	0.023	-0.012	-0.177	-0.019	-0.008	-0.101	0.012	-0.065	-0.154	-0.041	-0.065	-0.03	-0.013	0.008	-0.026	-0.022	-0.076
PAXIP1	0.083	0.035	-0.006	0.002	-0.095	0.055	0.011	-0.038	-0.022	0.028	-0.028	-0.071	0.013	0.021	-0.001	-0.101	0.01	-0.019	-0.063	-0.072	-0.046	-0.083	-0.026	-0.033	-0.028	0.005	0.015	-0.043	4E-04	-0.053
LIG1	0.092	0.215	-0.076	-0.157	-0.101	0.056	0.109	-0.058	-0.017	0.069	-0.016	-0.05	0.181	-0.004	-0.031	-0.054	-0.033	-0.062	-0.033	-0.098	-0.053	-0.068	-0.037	-0.075	-0.039	-0.031	0.01	-0.052	-0.03	-0.074
PCNA	0.131	0.179	-0.056	-0.12	-0.14	0.057	0.027	-0.09	-0.022	0.047	-0.063	-0.126	0.179	-0.031	-0.026	-0.062	0.065	-0.065	-0.061	-0.144	-0.073	-0.094	-0.049	-0.069	-0.053	-0.023	0.013	-0.073	-0.046	-0.076
GEN1	0.059	0.152	-0.054	-0.039	-0.111	0.057	-0.02	-0.086	-0.039	0.028	-0.055	-0.098	0.171	-0.027	-0.034	-0.045	0.085	-0.053	-0.043	-0.124	-0.074	-0.11	-0.068	-0.04	-0.067	-0.023	0.022	-0.093	-0.024	-0.069
FANCG	0.052	0.175	-0.034	-0.048	-0.131	0.06	0.03	-0.052	-0.023	0.085	-0.035	-0.11	0.164	0.002	-0.027	-0.109	-0.054	-0.042	-0.051	-0.09	-0.063	-0.113	-0.032	-0.044	-0.046	-0.015	0.018	-0.063	-0.009	-0.055
PMS1	0.126	0.007	0.065	-0.057	-0.118	0.06	-0.105	-0.014	-0.009	0.038	-0.037	-0.086	4E-04	0.02	0.036	-0.109	0.059	-0.049	-0.03	-0.076	-0.029	-0.103	-0.03	-0.015	-0.048	-0.001	0.044	-0.043	0.004	-0.034
POLD3	0.118	0.096	0.008	-0.07	-0.16	0.061	-0.056	-0.014	-0.033	0.046	-0.045	-0.142	0.093	0.015	-0.009	-0.157	0.185	-0.007	-0.067	-0.107	-0.084	-0.157	-0.054	-0.047	-0.06	-0.025	0.042	-0.052	0.016	-0.069
RAD18	0.203	0.09	-0.04	-0.087	-0.187	0.062	-0.007	-0.067	-0.047	0.051	-0.044	-0.168	0.08	-0.015	0.012	-0.1	0.045	-0.053	-0.093	-0.131	-0.064	-0.107	-0.058	-0.057	-0.061	-0.027	0.026	-0.094	-0.014	-0.057
XRCC3	0.121	0.153	-0.127	-0.034	-0.143	0.062	0.031	-0.091	-0.038	0.068	-0.054	-0.129	0.17	-0.004	-0.003	-0.057	-0.032	-0.055	-0.065	-0.13	-0.09	-0.112	-0.063	-0.059	-0.055	-0.032	0.013	-0.111	-0.029	-0.094
TREX1	0.029	0.162	-0.012	-0.173	-0.035	0.062	0.089	0.022	0.009	0.064	0.012	-0.006	0.12	0.025	7E-04	-0.05	-0.094	-0.022	-0.002	-0.012	0.014	-0.045	0.023	-0.09	0.019	-0.005	0.002	-1E-04	-0.005	-0.026
STRA13	0.004	0.004	-0.067	-0.006	-0.035	0.063	-0.023	-0.055	-0.009	0.04	-0.02	-0.002	0.019	-0.019	-0.001	0.07	-0.011	-0.046	0.012	-0.073	0.004	0.046	-0.011	0.023	-0.01	0.017	-0.024	-0.03	-0.014	-0.016
FANCI	0.108	0.18	-0.064	-0.072	-0.189	0.063	0.064	-0.082	-0.051	0.055	-0.049	-0.176	0.208	-0.015	-0.037	-0.084	0.06	-0.042	-0.081	-0.156	-0.078	-0.174	-0.063	-0.065	-0.066	-0.043	0.012	-0.11	-0.014	-0.091
TELO2	0.146	0.07	-0.13	-0.152	-0.064	0.064	0.113	-0.021	0.024	0.064	#####	-0.011	0.05	0.011	0.043	0.008	-0.116	-0.035	0.01	-0.051	-0.026	-0.013	-0.003	-0.073	-0.027	-0.012	-0.022	-0.026	-0.037	-0.064
PRPF19	0.119	0.077	-0.067	-0.107	-0.089	0.065	0.02	-0.062	-0.03	0.055	-0.045	-0.09	0.111	-0.04	0.005	0.003	-0.049	-0.025	-0.045	-0.103	-0.056	-0.055	-0.039	-0.021	-0.046	-0.016	0.006	-0.069	-0.028	-0.065
NEIL1	0.046	0.007	-0.049	-0.054	0.011	0.065	-0.082	-0.005	0.023	0.038	-0.033	0.069	-0.043	0.033	0.028	0.004	-0.004	-0.048	0.022	0.02	0.033	0.05	0.008	-0.002	0.017	0.012	-0.022	0.004	-0.018	-0.003
GTF2H2	0.081	-0.072	8E-04	-0.05	0.007	0.066	-0.035	-0.017	0.023	0.025	-0.02	-0.032	-0.08	0.028	0.031	-0.019	-0.024	-0.014	0.012	0.012	0.014	-0.022	0.017	-0.009	-0.004	0.025	0.008	0.014	-0.009	0.015
POLQ	0.109	0.183	-0.074	-0.059	-0.171	0.066	-0.005	-0.099	-0.048	0.05	-0.065	-0.166	0.221	-0.021	-0.033	-0.073	0.083	-0.057	-0.082	-0.179	-0.092	-0.162	-0.097	-0.046	-0.077	-0.04	0.023	-0.114	-0.017	-0.104
APEX1	0.036	-0.042	0.034	-0.002	-0.037	0.066	-0.004	-0.031	0.017	0.07	0.007	-0.053	-0.021	0.026	0.002	-0.032	-0.115	-0.011	-0.011	-0.036	-0.02	-0.035	-0.013	6E-04	-0.024	0.005	0.045	-0.036	-0.013	0.002
BLM	0.211	0.102	-0.11	-0.041	-0.218	0.069	0.034	-0.11	-0.059	0.058	-0.051	-0.19	0.149	-0.008	-0.002	-0.113	0.079	-0.05	-0.092	-0.176	-0.089	-0.184	-0.063	-0.084	-0.08	-0.056	0.019	-0.133	-0.044	-0.114
CCNH	0.17	-0.04	-0.118	-0.143	0.007	0.069	-0.006	0.007	0.04	0.034	-0.012	7E-04	-0.108	0.002	0.073	0.012	0.023	-0.022	0.02	0.02	0.054	0.014	0.033	-0.075	-0.011	0.019	0.015	0.047	-0.008	0.008
WEE1	0.12	0.15	-0.101	-0.043	-0.125	0.07	-0.07	-0.062	-0.05	0.045	-0.057	-0.103	0.127	-0.007	-0.018	-0.04	0.015	-0.033	-0.067	-0.119	-0.059	-0.076	-0.073	-0.009	-0.055	-0.052	0.003	-0.107	0.008	-0.045
CUL5	-0.025	-0.11	0.052	0.091	0.006	0.07	-0.157	0.019	-0.012	0.011	-0.025	-0.02	-0.076	0.021	0.016	-0.051	0.056	0.02	-0.024	0.025	0.007	-0.033	-0.003	0.031	-0.022	0.016	0.046	0.001	0.005	0.006
EME1	0.061	0.087	-0.049	-0.027	-0.14	0.07	0.065	-0.072	-0.029	0.04	-0.036	-0.122	0.126	0.002	-0.017	-0.038	0.023	-0.059	-0.039	-0.147	-0.062	-0.112	-0.039	-0.028	-0.064	-0.027	0.03	-0.1	-0.007	-0.07
SMARCA4	0.116	0.087	-0.034	-0.106	-0.122	0.07	0.077	-0.032	-0.033	0.095	-0.016	-0.098	0.11	0.009	0.014	-0.097	-0.063	-0.003	-0.065	-0.041	-0.057	-0.13	-0.034	-0.08	-0.033	-0.027	-0.006	-0.037	-0.011	-0.059
BRCA1	0.17	0.148	-0.224	-0.047	-0.139	0.072	0.029	-0.075	-0.028	0.048	-0.053	-0.134	0.139	-0.018	0.012	-0.06	0.086	-0.049	-0.082	-0.121	-0.072	-0.144	-0.048	-0.076	-0.085	-0.033	0.002	-0.079	-0.031	-0.083
ZSWIM7	-0.063	-0.089	0.02	-0.051	0.063	0.074	0.065	0.082	0.05	0.02	0.053	0.081	-0.125	0.047	0.022	0.038	-0.05	0.002	0.063	0.092	0.068	0.06	0.063	-0.006	0.027	0.037	0.004	0.046	0.02	0.048
XRCC2	0.121	0.071	-0.042	0.022	-0.177	0.076	0.002	-0.092	-0.048	0.052	-0.057	-0.177	0.105	0.005	-0.024	-0.111	0.047	-0.051	-0.105	-0.142	-0.088	-0.171	-0.069	-0.033	-0.079	-0.026	0.031	-0.101	-0.012	-0.093
POLE2	0.092	0.167	-0.127	-0.023	-0.14	0.079	-0.011	-0.105	-0.048	0.048	-0.07	-0.128	0.178	-0.023	-0.038	-0.03	0.04	-0.06	-0.072	-0.15	-0.087	-0.063	-0.076	-0.027	-0.063	-0.028	0.013	-0.104	-0.028	-0.075
TYMS	-0.008	0.061	0.037	-0.085	-0.053	0.079	0.083	-0.03	0.005	0.043	-1E-04	-0.036	0.083	0.01	-0.033	-0.05	0.001	-0.044	0.007	-0.068	-0.014	-0.072	0.002	-0.031	-0.019	0.002	0.027	-0.02	-0.007	-0.012
TTK	0.152	0.13	-0.045	-0.06	-0.192	0.08	-0.023	-0.097	-0.068	0.056	-0.07	-0.193	0.187	-0.023	-0.026	-0.087	0.076	-0.055	-0.102	-0.196	-0.092	-0.166	-0.101	-0.05	-0.088	-0.04	0.019	-0.122	-0.009	-0.087
CHAF1A	0.095	0.194	-0.129	-0.129	-0.119	0.081	0.088	-0.072	-0.048	0.082	-0.024	-0.116	0.195	-0.01	4E-04	-0.03	-0.015	-0.042	-0.045	-0.147	-0.062	-0.104	-0.054	-0.053	-0.05	-0.03	0.004	-0.08	-0.011	-0.089
POLL	-0.023	-0.066	0.031	-0.032	0.056	0.081	-0.04	0.031	0.045	0.069	0.016	0.092	-0.113	0.051	0.03	-0.003	-0.141	-0.004	0.04	0.068	0.033	0.052	0.008	0.018	0.026	0.046	0.009	0.057	-0.004	0.043
FANCF	0.163	-0.133	0.021	-0.047	-0.062	0.083	-0.061	-0.013	0.017	0.027	-0.029	-0.029	-0.14	0.009	0.035	-0.09	0.079	-0.017	-0.042	-0.001	-0.021	-0.029	-0.017	-0.026	-0.002	-0.002	0.02	0.007	-0.023	0.005
PARP4	0.161	-0.068	-0.056	-0.061	-0.032	0.085	-0.056	0.006	0.002	-0.002	-0.044	-0.013	-0.11	0.011	0.023	-0.094	0.137	-0.003	-0.033	0.031	9E-04	-0.031	-0.014	-0.067	0.006	0.005	0.014	0.036	-0.035	-0.026
RNF8	0.061	0.012	0.023	-0.057	-0.108	0.085	0.015	-0.057	-0.041	0.055	-0.029	-0.082	0.083	0.007	-0.016	-0.033	-0.037	-0.022	-0.029	-0.083	-0.057	-0.076	-0.029	-0.011	-0.025	-0.007	0.004	-0.086	-0.001	-0.042
RAD51	0.138	0.165	-0.099	-0.081	-0.188	0.086	0.046	-0.102	-0.051	0.074	-0.057	-0.182	0.201	-0.011	-0.019	-0.083	0.054	-0.045	-0.081	-0.183	-0.076	-0.168	-0.085	-0.065	-0.079	-0.047	0.018	-0.115	-0.015	-0.095
FEN1	0.056	0.138	-0.081	-0.089	-0.125	0.087	0.078	-0.09	-0.033	0.077	-0.04	-0.121	0.171	-0.015	-0.013	-0.034	0.024	-0.045	-0.034	-0.161	-0.06	-0.086	-0.041	-0.026	-0.058	-0.028	0.012	-0.082	-0.003	-0.065
CHEK2	-0.009	0.092	0.016	0.029	-0.102	0.087	-0.066	-0.069	-0.021	0.05	-0.06	-0.068	0.13	0.001	-0.044	-0.044	-0.01	-0.049	-0.026	-0.092	-0.077	-0.1	-0.095	-0.005	-0.032	4E-04	0.024	-0.072	-0.022	-0.06
RECQL4	0.11	0.112	-0.092	-0.061	-0.148	0.089	0.034	-0.094	-0.032	0.057	-0.049	-0.124	0.147	-0.01	0.002	-0.03	0.02	-0.061	-0.051	-0.157	-0.073	-0.097	-0.082	-0.033	-0.057	-0.027	0.013	-0.115	-0.035	-0.096
TOP3A	-0.025	0.013	-0.044	-0.022	-0.041	0.089	0.11	0.025	-0.011	0.04	0.031	-0.025	0.003	0.032	-0.004	-0.021	-0.025	-0.026	-0.015	-0.01	-0.006	-0.024	0.019	-0.006	-0.004	5E-04	-1E-04	-0.023	0.027	-0.031
RAD54L	0.127	0.144	-0.044	-0.071	-0.201	0.089	0.036	-0.085	-0.042	0.075	-0.057	-0.162	0.192	-0.01	-0.022	-0.101	0.018	-0.063	-0.082	-0.176	-0.092	-0.159	-0.086	-0.061	-0.08	-0.032	0.028	-0.112	-0.015	-0.092
EXO1	0.092	0.137	-0.071	-0.03	-0.179	0.089	0.058	-0.108	-0.06	0.04	-0.045	-0.175	0.187	-0.019	-0.036	-0.072	0.044	-0.056	-0.082	-0.192	-0.084	-0.14	-0.077	-0.025	-0.087	-0.036	0.007	-0.119	-0.012	-0.078
YWHAE	0.091	-0.013	0.036	-0.07	-0.039	0.09	-0.016	-0.015	-0.005	0.08	0.003	-0.03	-0.012	0.02	-0.006	-0.051	-0.117	-0.021	-0.035	0.058	0.001	-0.09	0.006	-0.087	-0.009	0.028	-0.027	-0.007	-0.047	-0.022
POLE	0.07	0.141	-0.097	-0.096	-0.116	0.092	0.047	-0.063	-0.036	0.073	-0.032	-0.083	0.141	-0.008	-0.006	-0.016	0.041	-0.068	-0.034	-0.137	-0.063	-0.077	-0.059	-0.027	-0.041	-0.017	0.024	-0.07	-0.014	-0.082
RTEL1	0.212	-0.032	-0.121	-0.136	-0.069	0.093	0.025	-0.019	0.021	0.05	-0.03	-0.009	-0.051	7E-04	0.074	-0.044	0.1	-0.051	-0.019	-0.084	-0.032	-0.031	-0.024	-0.086	-0.024	-0.014	-0.005	-0.016	-0.035	-0.055
BRCC3	0.216	0.069	-0.052	-0.055	-0.165	0.093	-0.127	-0.07	-0.033	0.046	-0.081	-0.143	0.045	-0.01	0.015	-0.132	0.093	-0.036	-0.084	-0.094	-0.051	-0.108	-0.076	-0.069	-0.04	-0.03	-0.004	-0.066	-0.026	-0.075
IDH1	0.009	-0.006	-0.127	0.063	-0.012	0.093	-0.111	-0.094	-0.051	0.016	-0.051	-0.001	-0.007	-0.012	0.013	0.069	0.046	-0.04	-0.026	-0.059	0.008	0.08	-0.061	0.037	-0.022	0.022	-0.012	-0.042	-0.03	-0.034
AEN	0.087	-0.13	-0.068	-0.077	-0.018	0.093	0.134	-0.033	0.009	0.058	0.03	0.019	-0.128	0.013	0.028	8E-04	0.048	-0.01	0.014	0.038	-0.003	-0.012	0.03	-0.076	-0.009	0.039	-0.045	0.046	-0.038	6E-04
TDG	-0.02	0.114	-0.024	0.084	-0.098	0.094	-0.121	-0.068	-0.041	0.062	-0.069	-0.111	0.169	-0.005	-0.052	-0.072	0.027	-0.033	-0.075	-0.054	-0.078	-0.139	-0.083	-0.025	-0.048	-0.006	0.005	-0.079	-0.04	-0.059
MPG	0.032	-0.035	-0.097	-0.036	-0.024	0.094	0.105	0.009	0.031	0.06	0.008	0.016	-0.063	0.019	0.032	-0.005	-0.098	0.001	0.044	0.032	0.014	0.022	0.017	-0.034	0.004	5E-04	-0.006	0.007	-0.013	-0.014
FANCD2	0.144	0.171	-0.056	-0.088	-0.206	0.094	0.03	-0.089	-0.033	0.072	-0.061	-0.171	0.18	-0.015	-0.01	-0.12	0.058	-0.061	-0.084	-0.152	-0.083	-0.171	-0.072	-0.07	-0.08	-0.027	0.038	-0.111	-0.031	-0.099
UBE2T	0.103	0.124	-0.051	-0.007	-0.189	0.095	0.017	-0.104	-0.048	0.049	-0.061	-0.168	0.148	-0.008	-0.034	-0.096	0.021	-0.062	-0.076	-0.17	-0.086	-0.123	-0.085	-0.03	-0.059	-0.024	-1E-04	-0.104	-0.013	-0.088
DNA2	0.086	0.1	0.011	-0.015	-0.143	0.095	-0.104	-0.081	-0.039	0.057	-0.084	-0.116	0.11	-0.004	-0.013	-0.092	0.083	-0.07	-0.065	-0.132	-0.074	-0.136	-0.085	-0.032	-0.058	-0.005	0.023	-0.073	-0.023	-0.08
CHEK1	0.146	0.04	-0.01	-0.063	-0.149	0.096	-0.046	-0.058	-0.02	0.063	-0.05	-0.13	0.084	-0.008	-0.008	-0.079	0.04	-0.047	-0.078	-0.12	-0.072	-0.142	-0.059	-0.051	-0.063	-0.018	0.037	-0.065	-0.023	-0.072
TDP1	0.117	0.147	3E-04	-0.15	-0.114	0.096	-0.018	-0.038	-0.015	0.102	-0.036	-0.106	0.164	0.023	-0.008	-0.086	-0.038	-0.054	-0.053	-0.088	-0.063	-0.124	-0.05	-0.108	-0.048	-0.003	0.016	-0.065	-0.011	-0.053
SMARCC1	0.177	0.059	-0.035	-0.069	-0.179	0.097	0.012	-0.059	-0.04	0.066	-0.048	-0.149	0.057	-0.002	0.011	-0.123	0.032	-0.029	-0.101	-0.069	-0.067	-0.125	-0.052	-0.07	-0.047	-0.032	0.02	-0.067	-0.019	-0.072
DCLRE1A	0.047	-0.058	0.047	0.026	-0.052	0.098	-0.153	-0.041	-0.025	0.025	-0.046	-0.045	-0.069	0.011	0.022	-0.045	0.062	-0.014	-0.036	-0.018	-0.009	-0.039	-0.026	0.008	-0.011	0.04	0.023	-0.023	-0.025	-7E-04
RMI1	0.107	0.076	-0.028	-0.01	-0.124	0.103	-0.162	-0.041	-0.054	0.043	-0.074	-0.125	0.067	0.01	0.016	-0.074	0.033	-0.022	-0.078	-0.085	-0.043	-0.092	-0.045	-0.018	-0.05	-0.013	0.022	-0.084	0.004	-0.05
NHEJ1	0.111	-0.221	0.006	-0.106	0.048	0.103	0.02	0.009	0.042	-0.009	0.021	0.042	-0.219	-0.008	0.042	0.016	0.073	0.014	0.014	0.027	0.039	0.061	0.025	-0.006	0.026	0.022	0.007	0.07	-0.007	0.014
REV3L	0.086	-0.128	0.009	-0.042	-0.063	0.105	0.019	0.04	-0.023	0.044	0.003	-0.034	-0.103	0.046	0.024	-0.072	0.079	0.015	-0.041	0.043	0.001	-0.094	0.023	-0.028	-0.028	0.007	0.016	0.02	0.003	-0.012
UBE2N	0.171	0.021	-0.035	-0.058	-0.126	0.105	-0.045	-0.069	-0.026	0.057	-0.04	-0.114	0.056	-0.011	0.01	-0.115	-0.019	-0.025	-0.054	-0.079	-0.026	-0.123	-0.022	-0.068	-0.033	-0.007	0.005	-0.05	-0.038	-0.053
RRM2	0.13	0.16	-0.147	-0.04	-0.171	0.106	0.007	-0.115	-0.056	0.052	-0.057	-0.164	0.194	-0.03	-0.029	-0.056	0.057	-0.064	-0.08	-0.191	-0.077	-0.127	-0.086	-0.049	-0.076	-0.035	-0.003	-0.112	-0.026	-0.086
RFC3	0.238	0.031	-0.058	-0.115	-0.171	0.107	-0.017	-0.074	-0.035	0.073	-0.06	-0.166	0.039	0.008	0.017	-0.111	0.108	-0.045	-0.086	-0.142	-0.065	-0.135	-0.063	-0.075	-0.061	-0.029	0.023	-0.08	-0.029	-0.09
NTHL1	0.137	-0.106	-0.088	-0.037	-0.034	0.108	-0.016	-0.042	0.001	0.043	-0.017	0.017	-0.13	0.014	0.053	0.043	-0.065	-0.048	0.03	-0.055	-0.002	0.068	-0.042	-0.004	0.002	-0.003	-0.008	-0.011	-0.028	-0.046
NEIL2	0.084	-0.17	0.013	-0.038	-0.048	0.108	0.099	0.01	0.006	0.009	0.044	-0.032	-0.192	0.027	0.05	-0.044	0.016	0.041	0.014	0.043	0.03	-0.023	0.051	-0.029	-0.004	0.042	0.004	0.004	-0.024	-0.002
CDC25A	0.236	0.089	-0.097	-0.136	-0.202	0.108	0.044	-0.106	-0.05	0.085	-0.057	-0.16	0.135	-0.016	0.008	-0.09	0.034	-0.054	-0.086	-0.162	-0.085	-0.147	-0.068	-0.094	-0.065	-0.032	0.012	-0.103	-0.04	-0.101
NUDT18	-0.083	0.029	-0.078	0.011	0.023	0.109	0.027	0.029	0.031	0.037	-1E-04	0.039	-0.016	0.022	0.005	-0.003	-0.06	0.02	0.032	0.093	0.036	0.004	0.016	-0.019	0.011	0.033	-0.014	0.035	-0.01	0.016
CDC25C	0.194	0.112	-0.102	-0.114	-0.175	0.109	-4E-04	-0.099	-0.053	0.065	-0.05	-0.159	0.131	-0.008	-3E-04	-0.049	0.055	-0.073	-0.079	-0.218	-0.064	-0.103	-0.088	-0.045	-0.081	-0.041	0.004	-0.115	-0.008	-0.073
FANCA	0.089	0.099	-0.123	-0.036	-0.119	0.11	4E-04	-0.081	-0.023	0.075	-0.064	-0.089	0.138	-0.006	-0.028	-0.031	0.061	-0.08	-0.046	-0.138	-0.065	-0.069	-0.07	-0.051	-0.059	-0.013	0.014	-0.069	-0.04	-0.085
HMGB2	0.178	0.143	-0.036	-0.143	-0.154	0.112	-0.065	-0.045	-0.028	0.053	-0.056	-0.145	0.148	0.001	0.013	-0.112	0.052	-0.046	-0.06	-0.169	-0.054	-0.136	-0.067	-0.076	-0.056	-0.027	0.029	-0.075	-0.003	-0.078
CDK7	0.071	0.093	-0.044	-0.081	-0.048	0.117	-0.082	-0.046	0.015	0.06	-0.038	-0.071	0.07	0.004	-0.005	-0.029	-4E-04	-0.022	-0.055	-0.011	-0.016	-0.052	-0.072	-0.071	-0.019	0.016	-0.007	0.008	-0.029	-0.014
NSMCE4A	0.167	0.017	-0.084	-0.116	-0.071	0.122	-0.148	-0.045	-0.028	0.049	-0.069	-0.042	0.002	0.017	0.047	-0.013	0.078	-0.049	-0.016	-0.102	-0.014	-0.01	-0.072	-0.009	-0.028	0.012	0.012	-0.038	-0.015	-0.042
H2AFX	0.169	0.073	-0.107	-0.074	-0.161	0.132	9E-04	-0.087	-0.039	0.098	-0.059	-0.128	0.103	0.005	0.016	-0.076	-0.014	-0.049	-0.067	-0.127	-0.062	-0.12	-0.055	-0.079	-0.065	-0.02	0.015	-0.09	-0.034	-0.088
TP53	0.169	0.036	-0.054	-0.104	-0.097	0.136	0.007	-0.031	0.005	0.066	-0.032	-0.063	-0.003	0.016	0.014	-0.121	-0.037	-0.048	-0.045	0.002	-0.011	-0.091	-0.004	-0.111	-0.026	0.026	0.012	-0.015	-0.045	-0.024
HMGB1	0.305	0.04	-0.093	-0.21	-0.125	0.142	-0.081	-0.047	-0.038	0.07	-0.064	-0.112	0.045	0.013	0.046	-0.107	0.077	-0.072	-0.067	-0.116	-0.029	-0.105	-0.052	-0.113	-0.045	-0.006	0.011	-0.05	-0.053	-0.072
NUDT15	0.13	0.054	-0.067	-0.076	-0.093	0.146	-0.04	-0.068	-0.041	0.078	-0.075	-0.114	0.079	0.002	-0.009	-0.079	0.031	-0.037	-0.082	-0.066	-0.041	-0.126	-0.078	-0.071	-0.037	0.003	0.012	-0.038	-0.052	-0.054
