# Supplementary material for: Correlation-based and feature-driven mutation signature analyses to identify genetic features associated with DNA mutagenic processes in cancer genomes
Source: Genomics Inform. 2021 Dec 31;19(4):e40. doi: 10.5808/gi.21047 (PMC8752981; doi:10.5808/gi.21047)
Supplement: Supplemental Table 4. — Correlation table of DNA damage and repair gene methylation and mutation signature levels [file gi-21047suppl8.pdf]

Supplementary Table 4. Correlation table of DNA damage and repair gene methylation and mutation signature levels

DDR gene	Sig1	Sig2	Sig3	Sig4	Sig5	Sig6	Sig7	Sig8	Sig9	Sig10	Sig11	Sig12	Sig13	Sig14	Sig15	Sig16	Sig17	Sig18	Sig19	Sig20	Sig21	Sig22	Sig23	Sig24	Sig25	Sig26	Sig27	Sig28	Sig29	Sig30
ALKBH3	0.044	-0.055	-0.102	-0.04	-0.06	0.304	-0.056	-0.038	0.001	0.093	-0.028	-0.031	-0.076	0.052	0.021	-0.051	0.015	-0.036	-0.024	0.022	-0.005	-0.022	-0.036	-0.056	-0.036	0.043	-0.022	-6E-04	-0.048	-0.033
MLH1	0.114	-0.029	-0.07	-0.109	-0.039	0.234	-0.058	-0.013	-0.01	0.063	-0.03	-0.03	-0.067	0.023	0.056	-0.053	0.008	-0.005	-0.037	0.009	-0.002	-0.037	-0.038	-0.105	-0.016	0.036	-0.003	0.016	-0.044	-0.024
MPLKIP	0.202	-0.028	-0.204	-0.07	-0.113	0.225	-0.067	-0.065	-0.015	0.011	-0.059	-0.082	-0.034	-0.014	0.081	-0.087	0.21	-0.011	-0.055	-0.084	-0.01	-0.077	-0.056	-0.097	-0.055	0.007	-0.024	-0.012	-0.047	-0.075
EXO5	0.281	-0.089	-0.164	-0.114	-0.088	0.213	-0.067	-0.041	-0.011	-0.014	-0.031	-0.05	-0.1	0.016	0.04	-0.068	0.047	-0.033	-0.034	-0.027	0.014	-0.05	-0.025	-0.082	-0.031	0.023	-0.017	-0.019	-0.058	-0.016
HLTF	0.101	-0.081	-0.097	-0.006	-0.053	0.2	-0.043	-0.037	-0.009	-0.016	-0.028	-0.029	-0.089	-0.007	0.047	-0.038	0.021	-0.007	-0.021	-0.033	0.026	-0.032	-0.027	-0.028	-0.02	0.024	-0.019	0.016	-0.02	-0.014
NSMCE3	0.037	0.024	-0.082	-0.014	-0.052	0.141	-0.021	-0.026	-0.014	-0.005	-0.03	-0.035	-0.007	0.017	0.014	-0.03	0.069	0.002	-0.016	-0.032	-0.021	-0.032	-0.028	-0.038	-0.019	0.007	-0.012	0.014	-0.017	-0.011
UBE2A	0.076	0.072	0.047	-0.093	-0.1	0.138	-0.038	-0.037	-0.002	0.087	-0.021	-0.059	0.042	0.04	-0.016	-0.13	-0.075	-0.023	-0.049	-0.033	-0.005	-0.065	-0.028	-0.047	-0.025	1E-04	-5E-04	-0.024	0.005	-5E-04
PPP4R4	-0.024	-0.012	-0.088	0.102	-0.041	0.117	-0.026	-0.018	0.021	-0.006	-0.035	-0.033	-0.006	-0.008	0.017	-0.033	0.033	-0.008	-0.034	-0.006	-0.011	-0.02	-0.012	-0.013	-0.025	0.031	-0.015	0.041	-0.003	-0.024
APEX2	0.066	0.095	0.012	-0.088	-0.085	0.113	-0.017	-0.026	0.006	0.084	-0.01	-0.054	0.066	0.039	-0.011	-0.124	-0.079	-0.019	-0.055	-0.024	-0.007	-0.056	-0.02	-0.035	-0.02	-0.011	-0.009	-0.025	5E-04	-0.006
ATRX	0.048	0.083	0.033	-0.08	-0.082	0.111	-0.017	-0.026	0.004	0.079	-0.008	-0.044	0.054	0.042	-0.004	-0.12	-0.081	-0.018	-0.047	-0.023	-0.003	-0.052	-0.012	-0.033	-0.021	-0.003	-0.012	-0.024	0.015	0.003
POLE4	0.083	-0.07	-0.086	0.02	-0.04	0.11	0.016	-0.015	-0.01	-0.022	#####	-0.018	-0.076	0.033	0.023	-0.025	-0.016	3E-04	-0.024	-0.005	-0.005	-0.004	0.009	-0.03	-0.003	0.005	-0.003	0.022	-0.027	-0.018
POLA1	0.037	0.092	0.018	-0.079	-0.075	0.11	-0.011	-0.026	-0.005	0.083	-0.015	-0.043	0.074	0.045	-0.01	-0.114	-0.078	-0.009	-0.051	-0.012	-0.002	-0.052	-0.018	-0.034	-0.022	-0.004	-0.009	-0.016	0.007	-0.006
CDC25B	0.088	0.016	-0.1	-0.022	-0.047	0.108	-0.035	-0.031	-0.025	-0.005	-0.034	-0.019	-0.008	-0.024	0.014	-0.038	0.055	-0.014	-0.027	-0.046	0.008	-0.032	-0.025	-0.045	-0.026	0.019	-0.016	0.015	-0.032	-0.004
RRM1	-0.037	0.008	-0.111	0.066	0.005	0.108	6E-04	-0.021	-0.008	0.026	-0.013	-0.004	0.032	9E-04	-0.004	0.019	-0.011	0.011	-0.031	0.011	-0.004	-0.031	-0.02	-0.026	-0.012	0.015	-0.031	0.013	-0.023	-0.03
CETN2	0.049	0.07	0.079	-0.085	-0.084	0.107	-0.027	-0.033	0.004	0.076	-0.009	-0.058	0.04	0.049	-0.01	-0.128	-0.083	-0.01	-0.041	-0.034	-0.008	-0.058	-0.007	-0.029	-0.022	-0.007	0.008	-0.031	0.016	0.009
RAD23B	-0.023	0.114	-0.066	-0.049	-0.019	0.085	0.03	-0.015	-0.001	0.051	0.007	-0.021	0.08	0.011	-0.004	0.023	0.003	-0.01	-0.034	-0.041	0.001	-0.017	-0.024	-0.045	-0.003	-0.027	-0.019	-0.008	-0.01	-0.031
MORF4L1	-0.041	0.083	-0.095	-0.026	0.029	0.076	0.004	-0.003	-0.002	0.068	0.004	0.02	0.076	-0.005	0.018	0.043	-0.109	-0.015	-0.025	0.024	0.024	0.032	-0.008	-0.017	#####	0.014	-0.021	0.025	-0.018	-0.018
WRN	-0.054	0.074	-0.112	0.026	-0.002	0.075	0.08	-0.007	-0.006	0.029	0.004	-0.024	0.033	-0.01	0.02	-0.003	0.025	0.024	-0.025	-1E-03	-0.008	-0.035	-0.013	-0.03	-0.022	0.012	-0.034	0.002	0.002	-0.024
SETMAR	0.005	0.007	-0.09	0.037	-0.001	0.074	-0.034	-0.006	0.014	0.033	-0.003	-0.007	-0.006	-0.01	0.036	-0.038	0.016	0.012	-0.023	0.001	0.026	-0.01	-0.022	-5E-04	-0.026	0.018	-0.026	0.017	-0.008	-0.009
BARD1	-0.027	0.045	-0.073	-0.089	0.061	0.073	0.039	-0.015	0.007	0.041	0.021	0.017	0.001	0.025	0.044	0.062	-0.037	-0.004	0.004	-0.002	0.051	0.059	0.021	-0.02	0.005	0.007	-0.022	0.014	-0.025	0.007
HERC2	0.124	0.027	-0.04	-0.023	-0.057	0.073	-0.104	-0.057	-0.05	0.035	-0.039	-0.093	0.036	4E-04	5E-04	-0.041	0.038	-0.043	-0.028	-0.088	-0.018	-0.096	-0.067	-0.057	-0.055	-0.018	0.016	-0.04	-0.025	-0.015
LIG4	-0.022	-0.008	-0.017	0.046	-0.024	0.063	-0.013	-0.014	-0.016	-0.022	-0.009	-0.004	0.004	-0.008	-0.005	-0.015	-0.02	-0.006	9E-04	0.003	-0.008	0.011	-0.003	0.015	-0.011	-0.011	-0.007	-0.012	0.002	0.016
CHEK1	-0.038	-0.023	-0.073	0.106	-0.017	0.057	-0.028	-0.022	-0.018	0.019	-0.005	0.002	0.002	0.003	0.014	-0.012	0.019	-0.004	-0.032	0.005	0.016	-0.015	-0.028	0.008	-0.013	-0.012	-0.026	0.042	0.003	-0.008
SPRTN	-0.012	0.022	-0.034	-0.061	0.052	0.056	0.066	0.043	0.042	2E-04	0.045	0.048	-0.038	0.036	0.018	-3E-04	-0.136	0.013	0.034	0.074	0.048	6E-04	0.049	-0.04	0.017	0.026	0.005	0.042	0.014	0.053
XRCC2	0.082	-0.117	-0.043	-0.157	0.076	0.048	0.017	0.035	0.022	0.018	0.045	0.069	-0.121	-9E-04	0.051	0.065	0.035	0.009	0.038	0.036	0.037	0.054	0.031	-0.027	0.026	0.038	0.011	0.056	0.015	0.034
RFC3	-0.044	0.017	-0.036	0.123	-0.029	0.045	-0.034	-0.007	-0.022	0.004	-0.03	-0.002	-0.004	0.003	0.001	-0.035	-0.033	0.014	-0.044	0.033	-0.01	-0.016	-0.007	0.011	-0.022	-0.002	-0.027	0.011	-0.005	-0.014
RPA2	-0.049	-0.018	-0.005	0.142	-0.035	0.043	-0.025	-0.005	-0.016	-0.028	0.002	-0.039	-0.027	0.003	-0.022	-0.01	-0.023	-0.013	-0.009	-0.013	-0.02	-0.011	-0.011	0.031	-0.012	0.019	-0.013	-0.002	-0.011	-0.006
SMUG1	-0.015	-0.017	-0.052	0.089	-0.047	0.041	-0.032	-0.001	-0.025	0.081	-0.035	-0.014	-0.018	0.016	0.003	-0.035	-0.041	-0.009	-0.008	0.024	-0.014	-0.01	-0.015	0.042	0.004	-0.004	-0.011	-0.003	0.017	-0.032
NABP2	-0.024	-0.016	-0.044	0.068	0.004	0.039	-0.022	0.011	-0.011	-0.013	0.008	0.039	-0.015	-0.005	-0.005	-0.016	-0.01	-0.01	0.027	0.009	0.019	0.003	-0.013	0.015	-0.005	-2E-04	-0.022	4E-04	-0.024	0.009
RDM1	-0.004	-0.063	-0.063	0.029	0.017	0.038	0.058	-0.014	0.002	-0.012	0.029	-0.001	-0.062	0.002	0.067	0.011	-0.023	0.01	0.007	-0.007	0.017	0.03	0.019	0.015	-0.008	0.024	-0.022	0.019	0.008	-0.014
DCLRE1A	0.016	0.057	-0.013	-0.126	0.063	0.035	0.048	0.019	0.018	0.046	0.015	0.027	0.021	0.021	-0.002	0.022	-0.102	0.003	-0.012	0.019	0.032	-0.008	0.011	-0.056	-0.003	0.016	0.004	0.027	0.02	0.025
NSMCE2	-0.011	0.016	-0.123	0.132	-0.021	0.035	-0.042	-0.033	-0.022	0.002	-0.028	-0.017	0.037	0.004	-0.019	-0.011	0.002	0.015	-0.042	0.023	-0.005	-0.026	-0.04	0.013	-0.026	0.011	-0.04	0.048	-0.031	-0.043
FANCF	-0.023	0.053	-0.058	0.06	-0.019	0.031	-0.004	-0.02	-0.021	0.025	-0.012	-0.031	0.035	0.011	-0.022	-0.016	-0.028	-0.012	-0.029	0.007	0.017	-0.015	-0.013	0.004	-0.016	0.04	-0.011	0.002	-0.019	-0.021
ERCC3	0.043	0.008	-0.094	0.042	-0.035	0.031	-0.036	-0.013	-0.017	0.017	-0.017	-0.015	-0.011	4E-04	0.021	-0.03	0.025	-0.002	-0.018	0.003	-0.009	-0.029	-0.028	-0.011	-0.019	0.024	-0.023	0.053	-1E-04	0.008
PER1	-0.061	0.129	-0.019	-0.037	-0.017	0.027	0.026	-0.04	-0.023	0.034	-0.01	-0.019	0.158	-0.001	-0.006	-0.021	-0.051	-0.008	-0.036	-0.012	-0.019	-0.032	-0.017	0.014	-0.021	0.027	-0.02	-0.011	-0.014	0.01
PPP4R1	-0.114	-0.024	-0.047	0.278	-0.039	0.025	-0.038	-0.033	-0.036	0.008	-0.021	-0.017	0.009	#####	-0.044	-0.04	-0.049	0.014	-0.046	0.048	0.002	-0.046	-0.017	0.06	-0.01	-0.011	0.021	-0.01	-0.017	-0.013
OGG1	-0.19	0.102	-0.164	0.169	0.023	0.024	0.039	-0.026	0.009	0.004	-0.024	0.033	0.153	-0.008	-0.028	0.041	0.058	-0.001	-0.021	0.05	0.002	0.01	-0.024	0.002	0.005	0.004	-0.012	0.045	-0.017	-0.029
REV1	-0.013	0.013	-0.175	0.143	-0.003	0.023	-0.013	-0.033	-0.028	0.011	-0.037	8E-04	0.036	0.011	-0.02	0.015	0.015	-0.008	-0.054	0.041	9E-04	0.01	-0.035	0.008	-0.016	0.015	-0.065	0.05	-0.041	-0.051
RAD51D	0.056	-0.054	-0.078	0.04	-0.021	0.022	-0.042	-0.011	-0.002	0.051	-0.029	-0.014	-0.047	4E-04	0.019	0.007	-0.004	0.019	-0.033	0.013	-0.006	-0.012	-0.006	-0.006	-0.005	-0.003	-0.021	0.047	0.006	-0.015
RAD51C	-0.015	-0.007	-0.1	-0.027	0.085	0.022	-0.032	0.017	0.018	0.038	-0.003	0.067	-0.008	0.009	0.031	0.064	-0.004	0.002	-0.003	0.026	0.005	0.06	7E-04	0.002	0.01	0.044	-0.018	0.042	-0.012	0.034
FANCE	-0.057	0.067	-0.063	-0.026	0.037	0.02	-0.003	-0.004	-0.004	-0.024	0.016	0.035	0.055	-0.008	-0.007	0.087	0.007	0.004	0.025	0.007	0.01	0.04	0.002	0.008	-0.006	0.034	-0.02	0.027	0.002	0.006
ERCC1	-0.042	-0.011	-0.038	0.098	-0.018	0.018	-0.011	0.007	-0.006	-0.028	0.007	-0.024	-0.015	-0.016	0.036	0.002	0.027	4E-04	-0.013	-0.006	-0.002	0.006	0.021	0.018	-0.009	0.003	-0.033	-8E-04	0.016	-0.007
SLX4	-0.015	-0.009	-0.054	0.094	-0.013	0.018	-0.014	-0.025	-0.016	-0.008	-0.019	-0.015	-0.001	-0.003	-0.014	-0.006	-0.005	-0.013	-0.021	-0.005	0.014	-0.002	-0.015	0.036	-0.011	0.034	-0.029	-0.003	-0.017	-0.011
RFC2	-0.026	-0.024	-0.039	0.127	-0.034	0.017	-0.026	-0.015	-0.025	-0.023	-0.002	-0.016	-0.029	0.002	-0.004	-0.025	0.018	0.014	-0.03	-0.003	-0.014	-0.01	-0.006	0.034	0.015	-0.019	-0.023	0.062	0.02	-0.004
RIF1	0.008	-0.029	-0.002	0.098	-0.016	0.017	-0.077	-0.023	-0.012	0.029	-0.043	-0.015	-0.002	0.005	-0.048	-0.057	-0.058	0.005	-0.032	0.021	0.006	-0.033	-0.03	0.044	-0.014	0.011	0.002	-0.003	-0.03	-0.004
FEN1	-0.05	0.01	-0.078	0.157	-0.018	0.016	-0.034	-0.029	-0.029	-0.001	-0.024	-0.018	0.02	-0.004	-0.017	0.002	-0.022	-0.005	-0.035	0.01	-0.003	0.006	-0.025	0.025	-0.009	0.064	-0.026	0.001	-0.008	-0.032
YWHAG	-0.08	-0.031	-0.002	0.216	-0.055	0.016	-0.044	-0.036	-0.026	-0.008	-0.02	-0.031	-0.018	0.008	-0.031	-0.037	-0.012	0.012	-0.047	0.045	0.024	-0.016	-0.019	0.03	-0.014	0.009	-0.035	0.01	-0.01	-0.016
RAD23A	-0.058	-0.012	-0.016	0.169	-0.037	0.016	-0.035	-0.029	-0.021	-0.012	-0.026	-0.025	-0.007	-0.006	-0.02	-0.032	-0.022	-0.007	-0.032	-0.004	0.012	-0.015	-0.02	0.057	0.006	-0.001	-0.02	-0.01	-0.023	-0.012
RAD9A	-0.021	-0.003	-0.019	0.067	-0.025	0.015	-0.013	-0.015	-0.011	0.002	-0.018	0.008	8E-04	5E-04	-0.01	-0.017	-5E-04	0.005	0.012	0.018	0.009	-0.006	-0.009	0.007	-0.016	0.002	-0.035	-4E-04	-0.027	-0.018
SOX4	-0.095	-0.008	-0.046	0.154	0.004	0.015	-0.025	-0.018	0.003	0.001	0.004	0.021	-0.003	-0.003	-0.015	0.016	-0.018	0.005	0.017	0.012	0.026	3E-04	0.005	0.034	-0.012	0.005	-0.027	0.005	-8E-04	0.011
ASCC3	-0.077	-0.003	-0.098	0.238	-0.028	0.014	-0.032	-0.026	-0.044	-0.001	-0.034	-0.018	0.031	0.013	-0.032	-0.027	-0.032	0.007	-0.046	0.043	-0.013	-0.012	-0.034	0.026	-0.01	-0.001	-0.029	0.031	-0.027	-0.01
FANCG	0.014	0.047	-0.157	-0.029	0.064	0.013	-0.045	0.018	0.029	0.017	0.017	0.066	0.014	0.009	0.039	0.074	0.009	-0.022	0.016	0.027	0.042	0.067	0.008	-0.025	0.014	0.027	-0.015	0.048	-0.023	0.009
POLE	-0.058	0.008	-0.059	0.208	-0.016	0.013	-0.069	-0.037	-0.035	0.019	-0.045	-0.019	0.033	0.014	-0.049	-0.038	-0.06	-0.005	-0.041	0.005	-8E-04	-0.028	-0.047	0.058	-0.021	-0.007	-0.012	-0.004	-0.035	-0.014
YWHAE	8E-04	-0.019	-0.056	0.128	-0.017	0.012	-0.045	-0.011	-0.014	0.004	-0.043	0.002	-0.005	-0.009	-0.026	-0.015	-0.045	-0.012	-0.024	0.021	-0.006	-0.008	-0.025	0.019	-0.023	-0.011	-0.014	0.004	-0.024	-0.014
DMC1	0.131	-0.037	-0.078	-0.052	-0.006	0.011	0.039	-0.009	-0.027	-0.005	0.034	-0.028	-0.034	0.018	0.039	-0.024	-0.096	0.004	-0.005	-0.026	0.032	-0.027	0.031	-0.026	-0.015	0.026	0.007	-0.022	-0.01	-0.007
UBE2N	-0.057	-5E-04	-0.051	0.171	-0.049	0.011	-9E-04	-0.013	-0.015	-0.034	0.017	-0.043	-0.019	-0.003	0.005	-0.028	0.042	-0.008	-0.018	-0.002	-0.009	-0.015	-0.023	0.04	0.005	-0.017	-0.029	-0.011	0.014	-0.021
MSH6	0.024	-0.052	0.034	0.061	-0.036	0.009	-0.029	-0.002	-0.011	-0.019	-0.005	-0.021	-0.053	-0.009	0.002	-0.037	-0.017	-0.005	-0.022	0.022	0.008	-0.017	-0.005	0.007	-0.014	-0.001	-0.015	0.002	0.006	0.006
RECQL	0.028	-0.008	-0.07	0.062	-0.028	0.008	-0.04	-0.032	-0.011	-0.007	-0.007	-0.016	-0.012	0.007	-0.012	-0.005	0.056	0.018	-0.013	-0.011	-0.016	-0.022	-0.031	0.005	-0.014	-0.015	-0.019	0.094	-0.011	-0.011
CDC25A	-0.116	-0.012	-0.133	0.303	-0.02	0.006	-0.009	-0.023	-0.028	-0.021	-0.011	-0.008	-0.012	-4E-04	-0.014	0.003	0.009	0.014	-0.042	0.038	-0.013	0.016	-0.024	0.05	0.001	-0.011	-0.042	0.016	-0.02	-0.036
MPG	-0.054	-0.023	0.099	-0.052	0.094	0.006	-0.056	0.017	0.015	0.045	0.027	0.089	-0.037	0.029	-0.03	0.051	-0.114	-0.007	0.012	0.098	0.031	0.034	0.02	-0.014	0.032	0.049	0.001	0.053	-0.006	0.05
UNG	-0.049	-0.013	-0.07	0.145	-0.033	0.006	-0.026	-0.025	-0.02	-0.015	-0.007	-0.004	-0.021	0.008	-0.018	-0.002	0.09	4E-04	-0.031	0.005	-0.014	-0.008	-0.031	0.026	-0.012	-0.002	-0.023	0.158	-0.018	0.015
TCEA1	-0.032	0.033	-0.076	0.119	-0.037	0.004	0.009	0.005	-0.015	-0.029	0.012	-0.03	0.015	0.011	0.017	-0.028	0.03	-0.006	-0.021	-0.015	-0.006	-0.026	-0.016	0.024	-8E-04	-0.012	-0.045	-0.005	-0.012	-0.019
PCNA	0.01	0.003	0.015	0.01	-0.029	0.004	0.01	-0.013	-0.001	-0.015	0.024	-0.016	-0.009	-0.004	0.005	-0.017	0.015	-0.002	-0.002	-0.015	-2E-04	-0.011	-0.002	0.006	-0.009	-0.008	-0.016	-0.015	0.002	-0.013
CHEK2	0.037	-0.044	0.041	-0.031	-0.026	0.003	-0.012	-0.02	0.005	-0.023	-0.007	-0.019	-0.051	-0.009	0.009	0.03	0.059	-0.018	0.003	-0.041	0.009	0.072	0.021	0.005	0.002	-0.012	-0.021	-0.017	-0.013	0.023
CUL3	-0.059	0.003	-0.015	0.123	-0.013	0.003	-0.001	-0.005	-0.012	-0.021	0.026	-0.025	0.007	-0.01	-0.013	-0.011	-0.02	0.013	-0.038	0.019	-0.009	-0.007	-0.008	0.016	-0.012	-0.007	-0.016	-0.004	-0.006	0.012
XRCC5	-0.035	0.025	-0.033	0.049	-0.033	0.003	-0.01	9E-04	-0.003	-0.018	0.009	-0.032	0.015	-0.016	0.006	0.08	0.006	-0.021	-0.014	-0.022	-0.007	0.115	-0.013	0.01	-0.002	0.006	-0.028	-0.009	-0.013	-0.013
POLN	0.021	0.042	-0.009	-0.095	0.03	0.002	-0.006	0.005	0.027	0.031	0.005	0.05	0.045	0.011	0.007	0.005	-0.02	0.004	0.009	0.011	0.023	-0.007	-0.01	-0.036	0.003	0.01	0.012	0.022	0.01	-0.007
GADD45G	-0.027	#####	-0.084	0.107	-0.006	0.002	0.022	-0.024	-0.009	-0.015	-0.025	-0.008	0.01	0.016	-0.025	-0.007	0.018	0.025	0.002	0.012	-0.02	-0.014	0.003	0.009	-0.025	-0.003	-0.022	0.041	0.002	-0.022
RAD17	-0.078	0.023	-0.04	0.199	-0.021	0.002	-0.042	-0.015	-0.011	0.004	-0.026	-0.003	0.021	0.006	-0.034	-0.066	-0.058	0.021	-0.009	0.039	-0.012	-0.009	-0.028	0.046	-0.018	0.002	-0.019	0.021	-0.012	6E-04
TP53	-0.056	0.017	-0.054	0.134	-0.021	5E-04	-0.017	0.017	-0.024	-0.005	0.019	-0.029	0.027	0.007	0.007	-0.036	-0.037	0.008	-0.039	0.033	-0.012	-0.004	-0.012	0.042	0.006	-4E-04	-0.028	-1E-03	-0.008	-0.017
TTK	-0.107	-0.021	-0.026	0.259	-0.033	3E-04	-0.025	-0.025	-0.031	-0.028	-0.026	-0.03	-0.012	0.001	-0.031	-0.019	-0.023	-0.007	-0.034	0.012	-0.011	0.005	-0.009	0.068	-0.005	-0.012	-0.022	0.024	-0.012	-0.01
SMARCC1	-0.062	0.013	-0.075	0.202	-0.041	3E-04	-0.021	-0.023	-0.029	-0.016	-0.013	-0.026	0.006	-0.004	-0.005	-0.04	-0.004	-0.014	-0.033	0.016	-0.011	-0.003	-0.022	0.053	-0.008	-3E-04	-0.039	-0.004	-0.006	-0.02
H2AFX	-0.075	0.002	-0.021	0.238	-0.061	-0.001	-0.035	-0.04	-0.032	-0.021	-0.024	-0.036	-0.008	-0.001	-0.026	-0.046	-0.023	-0.006	-0.027	-0.004	-0.011	-0.02	-0.024	0.057	-0.03	-0.022	-0.018	-0.016	-0.017	0.004
WEE1	-0.063	-0.089	-0.038	0.033	0.102	-0.002	-0.023	0.009	-0.015	-0.042	0.028	0.045	-0.085	-0.01	0.066	0.129	-0.017	0.024	0.037	0.026	0.026	0.107	0.045	0.051	0.013	0.022	-0.011	0.02	0.012	0.043
POLD4	-0.119	0.167	-0.12	0.089	-0.028	-0.002	0.084	-0.034	-0.018	0.067	-0.008	-0.027	0.201	-0.013	-0.031	0.022	-0.058	-0.012	-0.057	0.001	-0.014	-0.045	-0.027	-0.017	-0.014	0.023	-0.025	-0.012	-0.048	-0.039
SMC5	-0.041	0.002	-0.011	0.024	-0.007	-0.002	0.037	-0.002	0.005	-0.037	0.043	-0.008	-0.011	-0.004	0.02	0.031	0.064	-0.01	0.026	-0.036	-0.01	0.03	-0.002	0.015	0.015	-0.009	-0.016	-0.017	0.024	-0.009
FANCC	-0.083	0.018	-0.034	0.2	-0.042	-0.002	-0.017	-0.034	-0.016	-0.016	-0.018	-0.038	0.008	-0.001	-0.033	-0.008	-0.021	-0.011	-0.03	0.002	0.008	0.013	-0.019	0.042	-0.002	-0.008	-0.019	0.002	-0.005	-0.002
BRIP1	-0.036	0.023	-0.001	0.098	-0.017	-0.003	-0.024	-0.003	-0.024	0.008	0.01	-0.009	0.005	-0.008	-0.006	-0.049	-0.04	-0.008	-0.037	0.005	0.006	-0.024	-0.003	0.031	-0.018	-1E-04	0.053	0.009	0.021	0.009
POLG	-0.03	0.007	-0.044	0.141	-0.025	-0.003	-0.02	-0.006	-0.004	-0.031	0.008	-0.023	-0.012	-0.009	0.005	-0.013	-0.01	-0.034	-0.016	-0.014	-9E-04	0.007	-0.001	0.047	-0.014	-0.007	-0.042	-0.012	5E-04	-0.019
XRCC6	-0.034	-0.012	0.057	0.036	-0.024	-0.003	-0.015	-0.013	-0.003	-0.023	0.006	-0.004	-0.021	0.027	-0.021	-0.016	0.018	-0.005	-0.011	0.032	0.007	0.01	0.014	0.023	-0.01	-0.014	-0.006	5E-04	0.003	0.042
PMS2	-0.075	-0.002	-0.021	0.154	-0.011	-0.003	-0.017	-0.02	-0.022	-0.013	-0.003	0.008	0.002	-0.004	-0.016	-0.025	-0.017	-0.013	-0.025	-0.005	-0.013	-0.018	-0.016	0.066	-0.001	0.047	-0.014	-0.002	-0.002	-0.005
REV3L	-0.052	-0.008	-0.037	0.232	-0.059	-0.004	-0.066	-0.034	-0.026	-0.006	-0.02	-0.053	-0.009	0.006	0.006	-0.048	-0.028	-0.022	-0.041	-0.008	-0.006	-0.016	-0.029	0.055	-0.013	-0.013	-0.024	-0.007	0.004	-0.006
RAD54L	-0.033	-0.002	-0.006	0.003	0.002	-0.006	0.082	-0.012	-0.008	-0.01	-0.009	0.05	-0.008	-0.007	-3E-04	0.01	-0.009	-0.008	0.063	-0.013	#####	0.003	0.027	0.006	-0.009	-0.006	-0.007	-0.008	-0.009	0.016
POLD2	-0.079	-0.007	-0.07	0.26	-0.03	-0.006	-0.031	-0.032	-0.026	-0.014	-0.029	-0.024	8E-04	-6E-04	-0.016	-0.031	-0.007	-0.011	-0.045	0.014	-0.023	-0.007	-0.031	0.044	-0.016	-0.003	-0.051	0.02	-0.011	-0.028
TOPBP1	-0.084	0.011	-0.076	0.189	-0.034	-0.006	-0.007	-0.014	-0.023	0.007	-0.022	-0.029	0.014	0.03	-0.003	-0.009	-0.033	-0.002	-0.037	0.01	0.034	0.01	-0.014	0.054	-0.004	0.005	-0.032	0.007	0.013	-0.013
RAD51B	-0.043	-0.027	0.04	0.07	-0.005	-0.007	-0.032	-9E-04	-0.002	-0.036	0.015	-0.02	-0.036	-0.006	0.007	0.011	0.02	-0.028	6E-04	-0.034	0.028	0.036	0.017	0.063	-0.008	-0.017	-0.028	0.023	0.016	0.01
NEIL3	-0.004	-0.03	-0.043	0.073	-0.007	-0.007	-0.024	-0.017	-0.019	-0.01	-0.021	0.017	-0.02	-0.005	-0.013	0.003	0.011	0.013	-0.005	0.013	0.007	0.036	-0.023	0.019	0.004	-0.002	-0.022	0.055	-0.013	0.007
YWHAB	-0.09	-0.001	-0.049	0.273	-0.05	-0.007	-0.037	-0.008	-0.029	-1E-03	-0.02	-0.023	0.001	0.013	-0.038	-0.041	-0.05	-9E-04	-0.031	0.022	0.005	-0.012	-0.018	0.053	-0.009	-0.015	-0.039	0.006	-0.015	-0.023
MSH2	-0.06	-0.021	-0.081	0.223	-0.036	-0.008	-0.029	-0.023	-0.022	-0.028	-0.023	-0.022	-0.015	-1E-03	0.02	-0.002	-0.025	-0.005	-0.046	0.032	0.044	0.01	-0.013	0.049	0.008	-0.011	-0.035	-0.004	-0.021	-0.025
RMI2	-0.034	0.006	-0.01	0.072	-0.036	-0.008	0.016	-0.013	-9E-04	-0.032	0.034	-0.026	-0.006	-0.015	0.026	-0.014	0.068	-0.004	0.006	-0.018	-0.013	-0.014	-0.01	0.019	0.01	-0.02	-0.02	-0.011	0.002	-0.007
EXO1	-0.091	-0.006	-0.061	0.242	-0.052	-0.008	-0.008	-0.014	-0.017	-0.043	-0.005	-0.042	-0.005	-0.004	0.004	-0.021	0.011	-0.005	-0.011	-0.006	-0.021	-0.007	-0.024	0.072	-0.002	-0.017	-0.031	-0.005	0.007	-0.022
DCLRE1C	0.037	-0.094	-0.088	0.039	0.076	-0.008	-0.051	-0.03	-0.016	-0.024	5E-04	0.069	-0.095	-0.007	0.02	0.112	-0.034	-0.023	0.039	-0.012	0.031	0.105	-0.004	0.029	0.004	0.018	-0.018	-0.002	-0.01	0.01
TOP3B	-0.031	-0.02	0.068	0.09	-0.024	-0.009	-0.046	-0.007	-0.024	-0.019	-0.008	-0.03	-0.031	0.001	-0.012	-0.034	-0.024	-0.024	0.006	-0.016	-0.002	0.006	-0.007	0.041	0.004	-0.003	-0.018	-0.012	0.003	0.031
RTEL1	-0.026	0.08	-0.091	0.111	-0.034	-0.01	-0.014	-0.005	-0.006	0.009	-0.025	-0.008	0.088	-0.009	-0.02	-0.045	-0.036	-0.01	-0.033	0.011	#####	-0.027	-0.011	0.017	-0.017	0.002	-0.039	0.011	-0.022	-0.041
GADD45A	-0.108	-0.024	1E-03	0.243	-0.043	-0.01	-0.028	-0.035	-0.005	-0.015	-0.019	-0.025	-0.015	0.01	-0.038	-0.019	-0.002	0.003	-0.038	0.013	-0.005	-0.006	0.002	0.055	-0.008	-0.01	-0.014	-0.007	-0.006	-0.02
RAD9B	-0.063	0.01	-0.032	0.18	-0.048	-0.01	-0.008	-0.006	-0.014	-0.019	-0.016	-0.02	0.008	-0.006	-0.032	-0.027	-0.021	-0.005	-0.017	-0.001	-0.011	-0.01	-0.018	0.042	-0.012	0.011	-0.021	-4E-04	#####	-0.011
HMGB2	-0.111	0.032	-0.117	0.301	-0.036	-0.011	-0.013	-0.016	-0.026	-0.023	-0.039	-0.027	0.028	-0.005	-0.037	-0.023	-0.029	0.002	-0.038	0.017	-0.002	-0.004	-0.039	0.077	-0.025	-0.02	-0.048	-0.01	-0.013	-0.045
NHEJ1	-0.029	0.006	0.118	0.004	-0.028	-0.011	-0.002	-0.013	-0.007	-0.01	-0.003	-0.018	-0.002	-0.007	-0.018	-0.022	-0.003	0.001	-0.001	-0.016	-0.009	-0.014	0.007	0.019	-0.017	-0.004	-0.01	-0.012	-0.007	#####
MUS81	-0.038	0.015	-0.113	0.147	-0.015	-0.011	0.008	-0.032	-0.02	-0.011	-0.008	-0.013	0.038	-0.012	-0.008	0.009	-0.005	-0.007	-0.005	0.02	-0.016	0.014	-0.028	0.025	-0.014	-0.003	-0.037	0.007	-0.013	-0.041
NSMCE4A	-0.024	0.012	-0.013	0.09	-0.024	-0.012	-0.007	-0.009	-0.001	-0.007	-0.004	-0.019	0.01	0.002	-0.014	-0.021	-0.023	-0.013	-0.022	0.001	-5E-04	-0.006	-0.007	0.027	-0.013	-0.005	-0.018	-0.012	0.003	-0.006
MLH3	-0.031	-0.04	-0.018	0.038	-0.009	-0.012	0.14	-0.022	-0.018	-0.017	0.051	-0.034	-0.021	-0.012	0.056	-0.008	-0.034	0.06	-0.008	0.013	0.008	-0.019	0.037	-0.005	-0.016	-0.003	-0.01	0.011	-0.018	-0.032
LIG1	-0.07	-0.03	0.086	0.124	-0.017	-0.012	-0.028	-0.016	-0.012	-0.018	-0.027	-0.027	-0.033	-0.003	-0.019	-0.028	-0.012	-0.005	-0.003	-0.007	0.005	0.005	-0.007	0.045	-0.016	-0.005	-0.01	-0.012	-0.013	0.029
FAAP24	-0.075	0.033	-0.146	0.234	-0.013	-0.013	-0.003	-0.026	-0.029	-0.017	-0.026	-0.013	0.051	-0.006	-0.039	-0.022	-0.011	0.016	-0.044	0.043	-0.004	-0.008	-0.008	0.043	-0.023	0.006	-0.051	0.033	-0.012	-0.046
PARP2	-0.095	0.034	-0.068	0.208	-0.017	-0.013	-0.027	-0.022	-0.006	5E-04	-0.024	0.005	0.034	6E-04	-0.026	-0.018	-0.033	0.002	-0.047	0.045	0.004	-0.006	-0.017	0.047	-0.007	0.002	-0.047	0.012	-0.004	-0.023
CDK7	-0.073	0.03	-0.063	0.181	0.008	-0.014	-0.017	-0.03	-0.031	-0.003	-0.016	-0.006	0.028	-0.014	-0.017	0.003	-0.028	0.011	-0.014	0.012	-0.02	-0.018	-0.022	0.029	-0.013	-0.016	-0.025	-0.006	-0.028	-0.014
PTEN	-0.074	-0.018	-0.082	0.1	-0.026	-0.014	0.316	-0.017	-0.02	-0.012	0.094	-0.017	-0.017	-0.009	-0.03	-0.02	-0.022	0.008	-0.023	0.021	-0.008	-0.016	0.017	0.004	-0.008	-0.009	-0.013	-0.002	-0.025	-0.028
TREX1	-0.081	0.022	-0.103	0.248	-0.047	-0.014	-0.007	-0.04	-0.033	-0.004	-0.033	-0.016	0.024	-0.009	-0.025	0.002	-0.029	-0.015	-0.037	0.019	-0.01	0.006	-0.028	0.058	-0.016	0.001	-0.044	0.018	-0.017	-0.035
DNTT	0.084	-0.075	0.14	-0.093	0.013	-0.014	-0.028	0.058	0.026	0.028	0.011	0.026	-0.102	0.021	0.018	-0.104	-0.089	0.002	0.014	0.108	0.029	-0.05	0.045	-0.054	0.044	0.033	0.001	0.048	-0.007	0.031
UBE2B	-0.059	-0.009	0.028	0.099	-0.022	-0.015	-0.005	-0.009	-0.016	-0.025	-0.011	-0.023	-4E-04	-0.01	-0.015	-0.009	0.024	-0.009	-0.017	-0.015	-0.011	0.007	-0.007	0.03	0.004	0.003	-0.01	-0.016	-0.006	0.023
MAD2L2	0.073	-0.013	-0.115	-0.01	0.011	-0.015	-0.022	-0.015	0.026	0.001	-0.003	0.015	0.024	0.011	0.06	-0.006	0.001	0.019	-0.015	0.028	0.023	-0.009	0.011	-0.053	0.011	0.035	-0.015	0.053	-0.042	-0.024
IDH1	-0.128	0.02	-0.053	0.242	-0.016	-0.015	-0.002	-0.009	-0.025	-0.031	-0.015	-0.028	0.022	-0.012	-0.026	-0.02	-0.033	0.01	-0.007	0.027	0.008	0.012	-0.015	0.055	#####	-0.006	-0.027	0.007	-0.002	-0.005
PPP4R2	-0.107	0.003	0.04	0.166	0.004	-0.016	-0.042	-0.023	-0.03	-0.006	-0.045	-0.011	0.045	-0.012	-0.064	0.013	-0.055	0.009	-0.015	0.022	-0.006	-0.045	-0.027	0.051	-0.019	-0.003	6E-04	0.001	-0.011	-0.013
RNF4	0.051	-0.049	-0.023	-0.003	0.01	-0.017	0.064	0.016	0.01	0.018	0.033	-0.004	-0.03	0.013	0.005	-0.022	-0.057	0.041	-2E-04	0.045	-0.008	-0.032	0.004	-0.033	0.007	0.005	-0.026	0.016	-0.022	-0.015
TP53BP1	0.002	0.064	-0.018	-0.033	-0.003	-0.017	0.027	-0.002	0.021	-0.028	0.044	-0.006	0.032	-0.008	0.022	0.006	0.061	-0.023	0.005	-0.032	-0.017	0.012	-0.003	-0.036	0.016	-0.01	-0.016	-0.019	0.005	-0.023
MDC1	-0.124	-0.012	-0.07	0.309	-0.035	-0.017	-0.041	-0.033	-0.037	-0.017	-0.016	-0.029	-0.002	-0.001	-0.028	-0.006	-0.021	-0.013	-0.041	0.015	-0.007	0.018	-0.031	0.085	-0.012	-0.003	-0.039	0.008	#####	-0.015
POLH	-0.111	0.025	-0.148	0.303	-0.028	-0.018	-0.013	-0.039	-0.016	-0.005	-0.025	-0.02	0.027	-0.008	-0.04	-0.016	-0.009	0.008	-0.032	0.012	-0.008	-0.001	-0.038	0.08	-0.03	0.011	-0.041	0.009	-0.017	-0.032
RNF8	-0.092	-0.012	-0.02	0.254	-0.049	-0.018	-0.033	-0.023	-0.031	-0.021	-0.029	-0.03	-0.007	-0.01	-0.037	-0.028	-0.008	-0.004	-0.034	-0.004	-0.017	-0.006	-0.023	0.08	-0.016	-0.023	-0.023	0.02	-0.009	-0.016
TDP1	-0.111	0.004	-0.085	0.295	-0.038	-0.019	-0.027	-0.024	-0.022	-0.013	-0.014	-0.018	0.016	-0.004	-0.015	-0.034	-0.036	0.02	-0.035	0.025	-0.006	-0.018	-0.041	0.063	-0.018	-0.011	-0.025	-0.012	-0.015	-0.03
BRCA2	-0.057	0.03	-0.032	0.177	-0.023	-0.02	-0.019	-0.024	-0.017	-0.005	-0.021	-0.024	0.022	-0.011	-0.036	-0.03	-0.028	-0.003	-0.022	0.002	-0.006	-0.018	-0.028	0.029	-0.011	-0.014	-0.027	0.006	-0.01	-0.02
CUL5	-0.016	0.07	-0.096	0.045	-0.007	-0.02	0.021	-0.03	0.013	-0.01	-0.003	-0.009	0.065	-0.015	0.017	0.039	0.021	-0.003	-0.015	-0.002	0.003	0.02	-0.003	-0.019	0.012	0.003	-5E-04	-0.013	-0.044	-0.039
HELQ	-0.095	0.001	-0.03	0.263	-0.037	-0.02	-0.023	-0.021	-0.031	-9E-04	-0.04	-0.031	-0.006	0.008	-0.023	-0.05	-0.051	0.015	-0.041	0.034	-0.007	-0.032	-0.022	0.043	-0.012	-0.012	-0.018	-0.004	-0.015	-0.011
UBE2T	-0.046	-0.015	0.023	0.037	0.013	-0.02	0.013	-0.002	0.016	-0.029	0.009	-0.007	-0.012	-0.005	0.021	0.01	0.034	-6E-04	0.012	-0.023	-0.007	0.011	0.01	0.011	-0.002	0.009	-0.015	-0.008	0.016	0.04
RAD54B	-0.059	-0.002	-0.035	0.123	-0.013	-0.02	-0.014	0.009	-0.01	-0.02	0.002	0.006	-0.008	-1E-03	0.004	0.051	-0.014	0.002	0.004	0.007	-0.008	0.016	0.017	0.021	0.009	-0.015	-0.013	0.002	-0.007	0.013
RAD50	-0.097	0.028	-0.082	0.268	-0.05	-0.02	9E-04	-0.008	-0.027	-0.029	-0.013	-0.03	0.033	-0.018	-0.035	-0.024	-0.02	-0.01	-0.033	0.008	-0.018	-0.019	-0.021	0.06	-0.021	-0.012	-0.038	-0.008	-0.017	-0.045
APEX1	-0.083	0.025	-0.061	0.227	-0.024	-0.021	-0.044	-0.022	-0.017	-0.012	-0.039	-0.022	0.032	0.004	-0.024	-0.002	-0.042	-0.013	-0.05	0.022	-0.023	6E-04	-0.031	0.054	-0.021	0.004	-0.035	-1E-03	-0.002	-0.016
FANCA	-0.06	0.081	-0.03	-0.039	0.049	-0.021	0.043	0.042	0.004	0.017	0.046	0.059	0.061	0.03	-0.004	0.039	-0.164	0.012	0.045	0.054	0.017	0.026	0.027	0.005	0.019	0.014	0.005	0.031	0.032	0.04
MGMT	0.198	-0.133	-0.069	0.027	-0.025	-0.021	-0.058	-0.013	0.022	-0.015	0.025	-0.018	-0.114	0.005	0.026	-0.089	0.001	0.042	0.001	0.035	4E-04	-0.066	0.028	-0.067	0.009	0.003	-0.016	0.028	-0.029	0.004
RNF168	-0.045	0.01	-0.019	0.122	-0.03	-0.021	-0.011	-0.011	-0.012	-0.005	-0.01	-0.014	0.013	0.005	-0.006	-0.011	-0.022	0.002	-0.023	#####	0.002	-0.006	-0.02	0.023	-0.01	0.011	-0.024	0.01	0.007	-0.016
RBX1	0.018	-0.006	0.111	-0.021	-0.019	-0.022	-0.025	-0.003	-0.004	-0.03	0.002	-0.037	-0.027	-0.005	-0.001	-0.016	0.011	-0.006	-0.015	-0.035	0.004	0.008	0.04	0.013	-0.012	-0.002	-0.02	-0.02	0.007	0.013
XRCC4	-0.142	-0.031	0.022	0.325	-0.051	-0.022	-0.043	-0.022	-0.018	-0.024	-0.025	-0.031	-0.022	-0.01	-0.043	-0.036	-0.048	4E-04	-0.028	0.013	-0.007	-0.01	-0.011	0.085	-0.025	-0.018	-0.022	0.002	-0.002	-0.005
XRCC1	-0.099	0.034	-0.057	0.167	-0.003	-0.022	-0.016	0.001	-0.009	-0.025	-0.013	0.003	0.029	-0.004	-0.017	0.001	-0.013	-0.011	-0.015	0.02	-0.001	0.056	0.007	0.056	-0.001	0.004	-0.038	0.017	0.017	-0.006
ATR	-0.1	0.02	-0.049	0.223	-0.039	-0.022	-0.007	-0.009	-0.028	0.01	-0.013	-0.028	0.032	0.036	-0.04	-0.045	-0.017	0.015	-0.048	0.021	-0.009	-0.031	-0.012	0.042	-0.018	-0.027	-0.028	0.006	0.013	-0.021
CCNH	-0.097	6E-04	0.037	0.211	-0.028	-0.022	-0.036	-0.006	-0.016	-0.022	-0.018	-0.037	-0.009	-0.002	-0.03	-0.038	-0.047	0.009	-0.02	0.007	-0.008	-0.013	-0.023	0.056	-0.015	-0.009	-0.013	-0.004	0.002	-0.011
PRPF19	-0.082	0.003	-0.073	0.228	-0.032	-0.022	-0.03	-0.038	-0.024	-0.018	-0.031	-0.01	0.017	-0.005	-0.029	0.019	0.004	-0.012	-0.031	0.011	-0.005	-0.007	-0.032	0.047	-0.02	0.01	-0.034	0.05	-0.008	-0.009
NEIL1	0.051	-0.083	0.049	-0.061	0.025	-0.022	0.041	0.042	-0.005	-0.018	0.052	-0.032	-0.058	0.009	0.021	0.02	-0.072	0.033	0.019	-0.034	0.005	-0.016	0.026	0.011	0.002	-0.011	0.017	-0.009	0.041	0.016
DDB2	0.006	-0.044	-0.029	0.086	-0.02	-0.022	-0.028	-0.007	-0.015	-0.02	-0.005	-0.013	-0.029	-0.006	0.067	-0.002	-0.027	0.038	-0.019	0.012	0.051	-0.016	0.004	0.004	-0.01	0.008	-0.017	-6E-04	0.001	-0.02
GEN1	-0.116	-3E-04	-0.036	0.255	-0.04	-0.022	-0.031	-0.009	-0.033	-0.026	-0.017	-0.01	0.004	0.004	-0.019	-0.016	-0.031	0.01	-0.024	0.024	-0.011	7E-04	-0.028	0.051	-0.01	0.019	-0.026	0.016	0.007	-0.014
NUDT15	-0.056	-0.03	-0.078	0.298	-0.047	-0.023	-0.027	-0.041	-0.013	-0.011	-0.036	-0.051	-0.023	-0.008	-0.035	-0.041	-0.024	-0.005	-0.059	0.018	-0.024	-0.03	-0.02	0.052	-0.01	-0.008	-0.029	-0.01	-0.012	-0.019

ERCC6	0.026	-0.065	-0.028	0.038	0.007	-0.023	-0.014	0.037	0.011	-0.002	0.039	-0.015	-0.058	0.034	0.037	0.002	-0.006	0.026	-0.008	-0.004	0.007	-9E-04	0.021	0.01	0.001	-3E-04	-0.013	0.032	0.017	0.015
PARP4	-0.039	0.015	-0.128	0.217	-0.035	-0.023	-0.017	-0.053	-0.025	0.007	-0.023	-0.014	0.021	-0.009	-0.024	-0.009	-0.012	-0.012	-0.068	0.036	-1E-03	0.009	-0.033	0.055	-0.008	3E-04	-0.039	-5E-04	-0.038	-0.043
PLK3	-0.097	0.03	-0.067	0.239	-0.037	-0.023	-0.038	-0.004	-0.019	0.006	-0.03	-0.034	0.031	-0.004	-0.039	-0.023	-0.037	0.004	-0.032	0.013	-4E-04	0.035	-0.029	0.067	-0.006	2E-04	-0.032	0.02	-0.007	-0.033
PNKP	-0.019	-0.049	-0.071	0.075	0.019	-0.023	-0.023	0.015	-0.024	-0.033	0.005	0.03	-0.037	-0.013	0.023	0.063	-0.011	0.008	0.047	0.008	0.018	0.08	0.021	0.018	0.02	0.011	-0.023	0.012	0.014	2E-04
RFC1	-0.05	0.011	-0.023	0.128	-0.029	-0.024	-0.003	-0.007	-0.008	-0.011	-0.014	-0.013	0.011	-0.006	-0.022	-0.016	0.004	-0.008	-0.024	-0.004	-0.006	#####	-0.013	0.049	-0.012	-0.011	-0.019	-0.003	0.004	-0.017
SMARCA4	-0.083	-0.012	-0.062	0.249	-0.047	-0.024	-0.026	-0.018	-0.023	-0.008	-0.033	-0.019	0.007	-1E-03	-0.045	-0.025	-0.04	0.016	-0.003	0.015	8E-04	-0.012	-0.028	0.075	-0.008	-0.013	-0.024	0.012	-0.006	-0.026
APLF	-0.094	0.003	-0.034	0.239	-0.029	-0.024	-0.028	-0.02	-0.021	-0.009	-0.028	-0.011	0.008	-0.005	-0.043	-0.043	-0.039	-0.009	-0.028	0.029	-0.002	-0.023	-0.013	0.061	-0.012	-0.003	-0.015	0.025	0.005	0.016
PMS1	-0.105	0.021	-0.134	0.302	-0.025	-0.026	-0.029	-0.03	-0.035	-0.015	-0.035	-0.018	0.026	-0.004	-0.02	-0.004	-0.034	0.006	-0.032	0.025	-0.009	-0.005	-0.024	0.069	-0.015	-1E-03	-0.042	0.011	-0.006	-0.033
POLI	-0.045	0.025	-0.028	0.119	-0.029	-0.026	-0.013	-0.011	-0.005	-0.007	-0.012	-0.018	0.036	-0.011	-0.019	0.005	-0.021	-0.004	-0.02	-0.011	-0.003	-1E-03	-0.019	0.016	-0.004	-0.004	-0.027	0.001	-0.002	-0.024
HES1	-0.151	0.022	-0.071	0.303	-0.034	-0.026	-0.001	-0.014	-0.03	-0.024	-0.024	-0.034	0.036	-0.007	-0.037	-0.026	-0.027	0.021	-0.035	0.008	0.007	-0.022	-0.015	0.069	0.009	-0.009	0.007	-0.002	-0.011	-0.03
MNAT1	-0.008	-0.048	0.104	0.069	-0.008	-0.026	-0.033	0.007	-0.013	-0.036	2E-04	-0.039	-0.052	0.005	-0.017	-0.025	-0.024	0.008	-0.006	-0.025	-0.007	-0.011	0.013	0.029	0.005	-0.015	-0.004	-0.003	0.007	0.005
RRM2B	-0.059	-0.018	0.016	0.173	-0.03	-0.026	-0.022	-0.03	-0.027	-0.017	-0.014	-0.031	-0.022	0.01	-0.028	-0.032	-0.035	-0.002	-0.018	0.012	0.021	-0.003	-0.004	0.049	0.006	-0.014	-0.018	-0.003	0.014	0.007
DUT	-0.036	-0.012	0.073	0.071	-0.022	-0.026	-0.033	0.004	-0.005	-0.044	-0.007	-0.028	0.005	-0.012	-0.024	0.004	0.004	-0.021	-0.003	-0.035	-0.021	0.042	0.001	0.036	-0.009	-0.01	-0.023	-0.005	0.009	-0.007
CDC5L	-0.079	0.022	-0.065	0.209	-0.005	-0.026	-0.016	-0.003	-0.006	-0.046	-0.006	-0.03	0.004	0.015	-0.021	-0.01	-0.024	0.009	-0.014	0.01	-0.018	-0.003	-0.007	0.043	0.005	-0.013	-0.025	0.009	-0.006	0.007
NEIL2	-0.053	0.003	-0.097	0.163	-0.008	-0.027	-0.013	-0.026	-0.014	-0.008	-0.017	-0.024	0.016	0.007	-0.03	-0.002	0.078	0.007	-0.028	0.022	-0.012	0.003	-0.035	0.034	-0.025	-0.012	-0.026	0.102	-0.004	-0.024
POLB	-0.109	-0.019	0.038	0.174	-0.011	-0.027	-0.013	-0.02	-0.022	-0.027	0.009	-0.007	-0.019	0.013	-0.018	0.006	-0.006	-0.009	-0.021	-0.004	-0.018	0.025	4E-04	0.051	0.015	-0.004	-0.025	0.026	0.025	0.013
RFC4	-0.091	0.045	-0.138	0.277	-0.017	-0.027	-0.032	-0.036	-0.034	-0.021	-0.046	-0.018	0.053	-0.016	-0.01	1E-03	-0.039	-0.005	-0.058	0.03	-0.02	-0.008	-0.042	0.06	-0.013	0.007	-0.05	#####	-0.03	-0.039
RAD18	-0.16	0.031	-0.113	0.357	-0.03	-0.027	-0.021	-0.028	-0.031	-0.019	-0.034	-0.014	0.037	-0.018	-0.038	-0.017	-0.013	0.007	-0.052	0.046	-0.007	-0.009	-0.012	0.079	-0.017	0.004	-0.055	0.013	-0.032	-0.035
HMGB1	-0.106	-2E-04	-0.062	0.336	-0.05	-0.028	-0.039	-0.028	-0.037	-0.024	-0.03	-0.056	0.005	-0.006	-0.048	-0.052	-0.026	-0.006	-0.056	0.011	-0.008	-0.018	-0.025	0.073	-0.013	-0.019	-0.031	-0.003	-0.008	-0.016
NTHL1	-0.097	0.006	-0.079	0.263	-0.018	-0.028	-0.004	-0.04	-0.039	-0.008	0.023	-0.026	0.009	-0.011	-0.022	-0.018	-0.028	0.004	-0.045	0.027	-0.005	-0.029	-0.018	0.058	-0.011	-0.02	-0.047	0.004	-0.015	0.003
FANCL	-0.047	0.01	-0.059	0.184	-0.022	-0.028	-0.03	-0.033	-0.018	-0.005	-0.023	-0.018	0.027	-0.013	-0.028	-0.038	-7E-04	-0.011	-0.021	0.016	-0.011	-0.015	-0.034	0.029	-0.009	-0.005	-0.013	0.041	-0.015	-0.012
CDC25C	-0.1	0.012	-0.07	0.26	-0.028	-0.028	-0.03	-0.008	-0.015	-0.009	-0.021	-0.013	0.011	-0.007	-0.021	-0.028	-0.05	0.004	-0.037	0.028	0.002	-0.013	-0.019	0.066	-0.009	-0.003	-0.028	0.011	0.008	-0.006
GTF2H4	-0.079	0.007	-0.058	0.243	-0.041	-0.028	-0.011	-0.029	-0.022	-0.019	-0.014	-0.024	0.015	-0.005	-0.041	-0.031	-0.018	-0.002	-0.022	0.014	-0.01	-0.01	-0.019	0.055	-0.006	-0.007	-0.025	-6E-04	-0.012	-0.034
NBN	-0.101	0.029	0.011	0.2	-0.066	-0.028	-0.01	-0.003	-0.017	-0.004	-0.005	-0.038	0.014	0.003	-0.006	-0.049	-0.032	-0.016	-0.024	-0.005	2E-04	-0.01	0.006	0.066	-0.014	-0.016	-0.026	0.023	0.031	0.003
MRPL40	-0.108	0.042	-0.06	0.246	-0.043	-0.029	-0.032	-0.027	-0.033	-0.008	-0.047	-0.023	0.052	-0.01	-0.037	-0.004	-0.034	0.007	-0.044	-0.002	-0.021	-0.009	-0.035	0.064	-0.015	-0.014	-0.028	-0.008	-0.011	-0.023
NFATC2IP	-0.057	-0.009	-0.046	0.239	-0.039	-0.029	-0.04	-0.033	-0.03	-0.019	-0.019	-0.038	-0.005	-9E-04	-0.034	-0.042	-0.02	0.002	-0.021	0.007	-3E-04	-0.017	-0.028	0.056	-0.019	-0.014	-0.017	-0.007	1E-03	-0.002
XPC	-0.073	0.053	-0.051	0.12	-0.026	-0.03	0.064	-0.009	-0.019	-0.027	0.022	-0.037	0.028	-0.005	-0.007	-0.003	0.023	-0.008	-0.004	-0.017	-0.005	-0.018	0.007	0.025	0.012	-0.016	-0.029	0.008	0.025	-0.023
TDP2	-0.037	-0.003	-0.05	0.165	-0.037	-0.03	0.013	-0.022	-0.02	-0.018	-0.001	-0.028	-0.012	0.009	-0.025	-0.016	0.019	0.006	-0.026	0.007	-0.009	-0.012	-0.021	0.022	-0.014	-0.011	-0.026	0.042	-0.009	-0.028
FANCD2	-0.124	0.012	-0.035	0.256	-0.02	-0.03	-0.013	-0.025	-0.024	-0.029	-0.028	-0.028	0.011	-0.004	-0.03	-0.013	0.002	0.007	-0.029	0.016	-0.014	-0.01	-0.022	0.057	-0.013	-0.01	-0.029	0.021	-0.004	-0.013
ERCC2	-0.087	0.034	-0.071	0.092	0.052	-0.03	-0.013	-2E-04	0.008	-0.02	0.018	0.015	0.038	0.003	-0.004	0.009	0.016	0.025	0.052	0.024	0.01	0.012	-0.021	0.023	0.005	-0.01	-0.022	0.084	0.019	0.024
UIMC1	-0.078	-0.008	-0.06	0.238	-0.045	-0.03	-0.018	-0.005	-0.029	-0.019	-0.023	-0.041	-0.001	0.001	-0.009	-0.031	-0.013	0.012	-0.042	0.001	-0.007	-0.01	-0.018	0.063	-0.01	-0.022	-0.022	-0.003	0.031	-0.02
TYMS	-0.028	-0.002	-0.018	0.041	0.007	-0.031	-0.01	0.013	-0.018	-0.03	0.015	-0.015	-0.001	-0.004	0.054	0.042	-0.023	0.031	0.005	-0.004	0.013	0.017	-0.009	0.017	-0.005	0.006	-0.006	-0.017	0.026	0.012
POLD3	-0.096	0.003	-0.026	0.247	-0.036	-0.031	-0.028	-0.016	-0.022	-0.016	-0.02	-0.033	0.003	-0.002	-0.037	-0.021	-0.027	0.011	-0.028	0.027	-0.006	-0.016	-0.027	0.056	-0.013	-0.019	-0.023	0.009	-0.004	-0.017
PPP4C	0.004	-0.134	-0.037	0.003	0.075	-0.031	0.051	0.024	0.031	-0.037	0.036	0.087	-0.144	0.002	0.064	0.067	0.008	0.012	0.033	0.056	0.033	0.1	0.028	0.033	0.036	0.027	-0.014	0.064	0.026	0.045
DDB1	-0.087	0.02	-0.018	0.218	-0.045	-0.032	-0.026	-0.014	-0.01	-0.012	-0.012	-0.025	0.01	0.002	-0.027	-0.028	-0.029	-0.015	-0.02	0.017	0.007	-0.016	-0.027	0.041	0.003	-0.004	-0.023	0.013	0.009	-0.007
SMARCAD.	-0.088	0.004	-0.036	0.206	-0.017	-0.032	-0.019	-0.02	-0.018	-0.014	-0.01	-0.022	0.007	-0.003	0.003	-0.012	-0.028	-0.003	-0.02	0.013	-0.009	0.007	-0.019	0.056	7E-04	-0.012	-0.018	-0.003	-3E-04	-0.015
BCAS2	-0.099	0.022	-0.02	0.202	-0.03	-0.032	-0.012	-0.024	-0.028	-0.011	0.01	-0.022	0.027	-0.01	-0.031	-0.007	-0.028	-4E-04	-0.034	0.015	-0.006	-0.006	-0.025	0.056	-0.007	-0.006	-0.032	0.013	-0.011	-0.01
USP1	-0.082	0.011	-0.086	0.259	-0.043	-0.033	-0.022	-0.006	-0.021	-0.021	-0.031	-0.027	0.022	0.011	-0.033	-0.017	-0.036	0.012	-0.049	0.041	-0.014	-0.022	-0.023	0.06	-0.005	-0.009	-0.03	-4E-04	0.008	-0.028
ERCC5	-0.123	-2E-04	-0.059	0.293	-0.034	-0.034	-0.006	-0.02	-0.02	-0.026	-0.03	-0.04	0.012	-0.019	-0.027	-0.031	-0.013	0.009	-0.029	0.028	-0.012	-0.009	-0.013	0.061	0.003	-0.009	-0.029	0.002	#####	-0.021
ERCC4	-0.075	-0.006	0.043	0.15	-0.03	-0.035	-0.025	-0.019	-0.023	-0.02	-8E-04	-0.032	0.001	-0.008	-0.018	-0.011	-0.013	-0.013	-0.015	-0.016	-0.013	0.005	-0.005	0.064	-0.012	-0.006	-0.021	1E-03	0.025	-0.003
PARPBP	-0.102	0.005	-0.044	0.258	-0.023	-0.036	-0.041	-0.023	-0.02	-0.018	-0.02	-0.025	0.016	0.001	-0.011	-0.026	-0.054	-0.005	-0.039	0.009	-0.003	0.014	-0.016	0.071	9E-04	0.005	-0.027	-9E-04	0.001	-0.019
KAT5	-0.093	0.005	-0.07	0.264	-0.034	-0.036	-0.026	-0.03	-0.03	-0.016	-0.013	-0.031	0.011	-0.007	-0.026	-0.025	-0.014	0.015	-0.039	0.032	-0.012	-0.018	-0.031	0.06	-0.01	-0.011	-0.039	0.016	-0.004	-0.023
RECQL4	-0.044	0.009	-0.017	0.124	-0.045	-0.036	-0.016	-0.019	-7E-04	-0.003	0.017	-0.035	0.031	0.006	-0.025	-0.036	0.01	0.002	0.012	0.007	-0.003	-0.027	-0.004	0.029	-0.014	0.003	-0.008	0.034	-0.012	0.02
HUS1	-0.122	0.015	-0.041	0.296	-0.057	-0.036	-0.019	-0.029	-0.025	-0.019	-0.026	-0.043	0.007	0.015	-0.029	-0.02	-0.034	-0.017	-0.034	0.005	-0.006	-0.01	-0.021	0.073	0.002	-0.006	-0.011	-0.007	6E-04	-0.016
CHAF1A	-0.13	-0.017	0.027	0.258	-0.021	-0.036	-0.022	-0.031	-0.029	-0.039	-0.011	-0.043	-0.01	0.005	-0.032	-0.011	-0.01	0.009	-0.034	-0.002	-0.003	0.027	-0.006	0.069	-0.001	-0.013	-0.019	-0.014	-0.01	0.004
XPA	-0.13	0.002	-0.09	0.335	-0.038	-0.036	0.009	-0.032	-0.043	-0.028	-0.023	-0.039	0.02	-0.005	-0.038	-0.008	-0.037	6E-04	-0.046	0.017	-0.013	-0.004	-0.03	0.08	-0.01	-0.008	-0.039	-0.013	-0.005	-0.029
PARP3	-0.079	-0.012	-0.032	0.201	0.001	-0.037	-0.027	-0.018	-0.017	-0.011	-0.016	-0.029	-0.013	0.004	-0.029	0.019	-0.014	-0.019	-0.02	-0.003	-0.011	0.05	-0.016	0.056	-0.002	-0.014	-0.016	-0.004	0.001	-0.001
LIG3	-0.111	2E-04	-0.065	0.319	-0.041	-0.037	-0.033	-0.024	-0.035	-0.02	-0.029	-0.036	-3E-04	-0.004	-0.042	-0.024	-0.032	-0.002	-0.034	0.034	-0.022	-0.01	-0.035	0.069	-0.021	-0.005	-0.02	0.002	-0.004	-0.019
PARP1	-0.044	-0.028	-0.059	0.186	-0.031	-0.037	-0.015	-0.006	-0.007	-0.026	0.008	-0.022	-0.024	0.002	0.054	0.008	-0.029	0.014	-0.017	0.011	0.002	-0.011	0.004	0.035	-0.007	-0.005	-0.025	0.022	-0.003	-0.02
SWSAP1	-0.093	0.009	-0.074	0.243	-0.032	-0.038	-0.004	0.017	-0.025	-0.021	-0.02	-0.032	0.011	-0.011	0.003	-0.008	-0.012	0.038	-0.035	0.009	-0.021	-0.009	-0.026	0.051	-0.019	-0.01	-0.04	1E-04	-6E-04	-0.041
RAD51	-0.125	-0.004	-0.013	0.279	-0.039	-0.041	-0.028	-0.021	-0.025	-0.02	-0.032	-0.027	0.008	-0.004	-0.036	-0.025	-0.024	0.009	-0.024	0.003	-0.013	-0.009	-0.024	0.069	0.001	-0.016	-0.014	-0.001	0.013	-0.001
FAN1	-0.14	0.041	-0.073	0.13	-0.008	-0.041	0.215	-0.013	0.009	-0.045	0.066	-0.006	0.069	-0.001	-0.002	-0.021	-0.002	0.016	0.015	0.004	-2E-04	-0.006	0.039	0.013	0.003	-0.002	-0.021	-0.003	-0.014	-0.027
RMI1	-0.094	0.007	0.054	0.127	-0.011	-0.045	0.015	-0.012	-0.015	-0.038	0.014	-0.024	0.008	-0.007	-0.016	0.018	-6E-04	-0.017	-0.015	-0.028	-0.009	0.013	0.004	0.05	-0.002	-0.01	-0.018	-0.01	0.011	0.016
GTF2H3	-0.118	-0.044	0.035	0.063	0.125	-0.046	-0.102	0.044	0.045	-0.007	0.017	0.096	-0.038	0.012	0.01	0.071	-0.059	0.015	0.045	0.081	0.017	0.09	0.005	0.064	0.029	0.012	0.012	0.066	0.036	0.052
POLD1	0.088	-0.081	-0.02	-0.012	0.021	-0.046	-0.039	0.026	0.026	#####	0.023	0.033	-0.084	0.031	0.024	-0.012	-0.068	0.018	0.012	0.037	0.029	0.005	0.04	0.011	0.02	0.004	0.003	0.017	0.027	0.049
ATM	-0.127	0.03	-0.057	0.284	-0.031	-0.048	-0.022	-0.016	-0.017	-0.005	-0.012	-0.032	0.027	-0.004	-0.044	-0.011	-0.028	-0.019	-0.038	-0.006	-0.02	0.018	-0.022	0.092	-0.015	-0.02	-0.037	-0.015	0.001	-0.02
BLM	-0.161	0.012	-0.062	0.359	-0.038	-0.049	-0.02	-0.034	-0.037	-0.026	-0.028	-0.041	0.019	-0.002	-0.053	-0.009	-0.024	-0.002	-0.04	0.013	-0.015	0.004	-0.032	0.09	-0.012	-0.011	-0.035	0.005	0.01	-0.018
FAAP20	-0.057	-0.002	-0.02	0.238	-0.049	-0.05	-0.029	0.003	-0.031	-0.017	-0.017	-0.033	-0.004	0.009	-0.038	-0.042	-0.036	-0.016	-0.03	-0.002	0.015	-0.026	-0.018	0.058	-0.006	-0.02	-0.019	1E-04	0.001	-0.008
RRM2	0.018	-0.037	0.033	-0.112	0.079	-0.05	0.047	0.084	0.003	-0.017	0.051	0.081	-0.066	0.012	0.037	0.031	-0.114	0.021	0.059	0.057	0.041	0.076	0.073	0.012	0.047	0.014	-0.002	0.038	0.043	0.076
SMC6	-0.091	-0.015	-0.032	0.041	0.099	-0.051	0.004	0.041	0.033	-0.047	0.043	0.051	-0.032	0.001	0.037	0.061	0.006	0.022	0.065	0.071	0.028	0.082	0.022	0.028	0.073	0.015	-0.029	0.077	0.014	0.02
RFC5	-0.145	0.008	-0.028	0.345	-0.049	-0.051	-0.024	-0.027	-0.035	-0.023	-0.026	-0.04	0.016	-0.003	-0.055	-0.039	-0.048	1E-04	-0.022	0.013	-0.027	9E-04	-0.032	0.085	-0.021	-0.019	-0.015	-0.013	0.008	-0.013
RECQL5	0.042	-0.059	0.038	-0.04	0.062	-0.059	-0.006	0.041	-0.008	-0.017	0.004	0.05	-0.05	0.011	0.012	0.015	-0.069	0.018	0.013	0.044	-0.005	0.032	0.016	-0.011	0.008	-0.003	0.019	0.021	0.014	0.023
EME1	0.052	0.033	-0.014	0.019	-0.023	-0.061	-0.025	-0.011	-0.014	-0.023	0.024	-0.015	0.037	0.009	0.002	-0.043	0.001	0.004	-0.037	-0.035	-0.013	-0.018	-5E-04	-5E-04	-0.014	-0.003	-0.018	-0.007	0.002	-0.004
MMS19	-0.035	0.017	0.008	0.114	-0.024	-0.061	0.023	-0.005	-0.02	-0.045	0.011	-0.036	0.001	0.001	-0.013	-0.005	-0.001	-0.022	-9E-04	-0.011	-0.015	0.025	0.038	0.02	0.001	-0.009	-0.028	-0.012	0.01	6E-04
RBBP8	-0.099	0.219	-0.04	0.036	-0.014	-0.062	0.035	-0.041	-0.025	0.008	-0.031	-0.022	0.259	-0.005	-0.062	-0.015	-0.038	-0.016	-0.001	-0.019	-0.026	-0.03	-0.032	-0.02	-0.003	0.005	-0.014	-0.018	-0.015	-0.016
XRCC3	0.09	-0.076	-0.049	-0.039	0.09	-0.073	-0.064	0.065	0.034	-0.038	0.037	0.053	-0.105	0.004	0.044	0.023	-0.025	0.035	0.041	0.002	0.047	0.043	0.051	0.006	0.019	0.019	-0.016	0.052	0.031	0.078
UBE2V2	-0.032	-0.013	0.003	0.02	0.09	-0.078	-0.033	0.084	0.066	-0.033	0.038	0.071	-0.032	0.032	0.022	-0.029	-0.04	0.041	0.043	0.125	0.047	-0.008	0.036	-0.017	0.02	0.025	0.002	0.085	0.005	0.061
MSH3	-0.095	-0.012	0.079	0.033	0.081	-0.08	0.052	0.059	0.012	-0.003	0.027	0.073	-0.007	0.029	-0.051	0.044	-0.196	0.012	0.03	0.081	0.008	0.024	0.048	0.04	0.021	0.003	0.013	0.047	0.03	0.062
SPO11	-0.103	-0.151	0.204	0.043	0.079	-0.081	0.048	0.089	0.038	-0.031	0.069	0.052	-0.16	0.05	-0.001	-0.006	-0.136	0.046	0.059	0.111	0.027	0.019	0.097	0.074	0.045	0.019	0.044	0.064	0.063	0.076
POLL	-0.13	0.028	0.087	0.04	0.091	-0.1	0.022	0.052	0.033	0.002	0.053	0.074	0.006	0.037	-0.008	0.068	-0.226	0.015	0.049	0.078	0.012	0.065	0.069	0.055	0.048	0.004	0.028	0.037	0.042	0.041
AEN	-0.113	0.018	-0.031	0.034	0.115	-0.117	0.117	0.028	0.039	-0.04	0.07	0.059	0.037	-0.004	-0.008	0.083	-0.052	0.029	0.044	0.057	0.02	0.03	0.047	0.019	0.036	0.023	-0.012	0.068	0.002	0.03
BRCA1	-0.062	-0.037	0.103	0.022	0.074	-0.151	0.037	0.063	0.046	-0.081	0.06	0.054	-0.036	0.007	0.021	0.046	-0.115	0.037	0.08	0.029	0.011	0.056	0.065	0.044	0.057	0.011	0.013	0.028	0.044	0.053
